# Proteomics Portrait of Archival Lesions of Chronic Pancreatitis

**DOI:** 10.1371/journal.pone.0027574

**Published:** 2011-11-23

**Authors:** Sheng Pan, Ru Chen, Tyler Stevens, Mary P. Bronner, Damon May, Yasuko Tamura, Martin W. McIntosh, Teresa A. Brentnall

**Affiliations:** 1 Department of Medicine, University of Washington, Seattle, Washington, United States of America; 2 Digestive Disease Institute, Cleveland Clinic Foundation, Cleveland, Ohio, United States of America; 3 Department of Anatomic Pathology, University of Utah, Salt Lake City, Utah, United States of America; 4 Fred Hutchinson Cancer Research Center, Molecular Diagnostics Program, Seattle, Washington, United States of America; Deutsches Krebsforschungszentrum, Germany

## Abstract

Chronic pancreatitis is a chronic inflammatory disorder of the pancreas. The etiology is multi-fold, but all lead to progressive scarring and loss of pancreatic function. Early diagnosis is difficult; and the understanding of the molecular events that underlie this progressive disease is limited. In this study, we investigated differential proteins associated with mild and severe chronic pancreatitis in comparison with normal pancreas and pancreatic cancer. Paraffin-embedded formalin-fixed tissues from five well-characterized specimens each of normal pancreas (NL), mild chronic pancreatitis (MCP), severe chronic pancreatitis (SCP) and pancreatic ductal adenocarcinoma (PDAC) were subjected to proteomic analysis using a “label-free” comparative approach. Our results show that the numbers of differential proteins increase substantially with the disease severity, from mild to severe chronic pancreatitis, while the number of dysregulated proteins is highest in pancreatic adenocarcinoma. Important functional groups and biological processes associated with chronic pancreatitis and cancer include acinar cell secretory proteins, pancreatic fibrosis/stellate cell activation, glycoproteins, and inflammatory proteins. Three differential proteins were selected for verification by immunohistochemistry, including collagen 14A1, lumican and versican. Further canonical pathway analysis revealed that acute phase response signal, prothrombin activation pathway, and pancreatic fibrosis/pancreatic stellate cell activation pathway were the most significant pathways involved in chronic pancreatitis, while pathways relating to metabolism were the most significant pathways in pancreatic adenocarcinoma. Our study reveals a group of differentially expressed proteins and the related pathways that may shed light on the pathogenesis of chronic pancreatitis and the common molecular events associated with chronic pancreatitis and pancreatic adenocarcinoma.

## Introduction

Chronic pancreatitis, a destructive fibroinflammatory disease of the pancreas, can present with a variety of symptoms. There are several causes of chronic pancreatitis, including toxic, obstructive, and inherited, but all lead to progressive scarring and loss of pancreatic function. Advanced fibrosis may lead to exocrine and endocrine failure, resulting in abdominal pain, weight loss, nutritional deficiencies, and brittle diabetes. Chronic pancreatitis presents with non-specific symptoms which can be mild; thus, the disease is significantly under-diagnosed. The prevalence of chronic pancreatitis appears to increase dramatically in the US and Europe during the past decade [1]–[3]. This is an increasingly common disease in the population and yet is difficult to diagnose yielding a very high cost per diagnosis ratio.

There is a limited understanding of the underlying molecular events in chronic pancreatitis. It has previously been shown that patients with chronic pancreatitis have an increased risk of developing pancreatic cancer [4]–[7]; thus it is not surprising that many molecular features presented in pancreatic cancer are also presented in chronic pancreatitis [4]–[9]. Proteins are the essential biological molecules that participate in numerous physiological functions; the changes at the mRNA level may not always directly correlate to the changes at the protein level [10]. Thus, further exploration of the pancreatitis disease-specific protein expression profiles could 1) lead to better diagnostic tests, 2) shed light on the mechanisms that underlie the disease, and 3) provide information regarding the relationship of chronic pancreatitis to pancreatic adenocarcinoma.

The emerging technology of quantitative proteomics provides a powerful tool for the systematic identification of dysregulated proteins associated with specific disease settings, and has been widely applied to investigate an assortment of diseases, including pancreatic cancer and pancreatitis [11]–[15]. Several studies have reported tissue proteomics analysis on pancreatic carcinoma, pancreatic intraepithelial neoplasia, and chronic pancreatitis, providing new insights and hypotheses to further the understanding of disease mechanism and biomarker development [8], [16]–[23]. While most prior studies have used snap-frozen tissue specimens, recently developed protein extraction techniques permit the use of fixed, paraffin-embedded tissue specimens for global proteomics analysis [24]–[29], providing a rich source of pathologically well-characterized specimens for clinical proteomics study.

In this study, we investigate dysregulated proteins in early and late stages of chronic pancreatitis, and compare them with the proteins present in normal pancreas and pancreatic ductal adenocarcinoma. The use of paraffin-embedded, formalin-fixed materials provides the gold-standard for histologic analysis, which in turn further optimizes proteomic correlation.

## Methods

### Patients and formalin-fixed paraffin-embedded pancreatic tissue specimens

**Figure 1:**
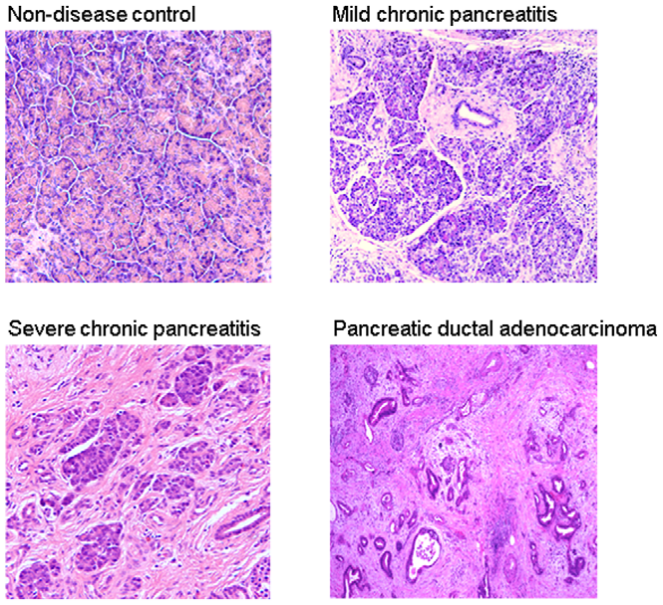
Histology of chronic pancreatitis, pancreatic adenocarcinoma and normal pancreas. a). non-disease pancreas; b). mild chronic pancreatitis; c). severe chronic pancreatitis; d). pancreatic ductal adenocarcinoma.

The formalin-fixed, paraffin-embedded pancreatic tissues from pancreatic ductal adenocarcinoma and chronic pancreatitis patients as well as normal healthy controls were obtained from surgical specimens at the Cleveland Clinic with the approval of the Cleveland Clinic Institutional Review Board and under HIPPA compliant guidelines. The IRB approval was obtained with a waiver of informed consent. Four study groups with five cases in each group were included, including pancreatic ductal adenocarcinoma (PDAC), mild primary chronic pancreatitis (MCP), severe primary chronic pancreatitis (SCP) and normal pancreas controls (NL) (Table S1). The diagnoses for the patients and tissues used for this study were confirmed by an experienced pancreatic pathologist (co-author MPB), through the review of all current and past histologic slides per patient, in addition to the blocks selected for proteomic analysis. The diagnoses were further confirmed by an experienced gastroenterologist specializing in pancreatic disease (co-author TS). Representative pathologic images from each of the four categories are shown in Figure 1. The mild and severe pancreatitis diagnoses were assessed as previously described [30] and based on the system proposed by Ammann and colleagues [31]. Pancreatic parenchyma is composed of grouped lobules of acini (functional units of the exocrine pancreas) emptying into ducts. Chronic pancreatitis is quantified according to the amount of intralobular and perilobular fibrosis within the pancreatic parenchyma. The severity of fibrosis as mild, moderate or severe and its distribution as focal or diffuse within (intralobular) or surrounding (perilobular) tissue is quantified based on a 0 to 12 point score [30], [31]. Mild pancreatitis was defined by scores of 1–4 and severe pancreatitis was defined by scores of 9–12. For this proteomics study, scores in the moderate chronic pancreatitis range of 5–8 were not used in an attempt to avoid overlapping results. Diagnostic tissues were manually circled under microscopic guidance by the pathologist (co-author MPB). Pancreatic adenocarcinomas were selected to contain greater than 50% adenocarcinoma cells.

### Extraction of proteins from paraffin-embedded tissue

Unstained 15 µm thick sections of formalin-fixed, paraffin-embedded tissue were deparaffinized and rehydrated by heating to 60°C for 45 minutes, followed by a series of washes: xylene (twice, 5 minutes each), 100% ethanol (twice, 5 minutes each), 85% ethanol (once, 3 minute), 70% ethanol (once, 3 minute), 50% ethanol (once, 3 minute) and de-ionized water (once, 1 minute). The corresponding H&E slides with the selected and circled diagnostic areas of pancreatic adenocarcinoma, mild chronic pancreatitis and severe chronic pancreatitis tissues were aligned with the parallel tissue profiles on the prepared slides and were manually microdissected. An equivalent of 1 cm×1 cm×15 µm amount of deparaffinized tissue samples were scraped from each slide and transferred into 300 µl lysis solution of 70% 50 mM ammonium bicarbonate and 30% acetonitrile, using sterile blades and needle tips. The lysates were then incubated at 90°C for 30 minutes followed by 60°C for 120 minutes to rehydrate the proteins and hydrolyze crosslinks. The samples were sonicated for 2 minutes followed by 1 minute incubation on ice. The sonication and ice incubation process was repeated twice more. The homogenized samples were incubated at 60°C for 1 hr, followed by the second sonication steps.

### Sample preparation for proteomics analysis

The protein lysates were digested with sequencing-grade trypsin (Promega, Madison, WI) at a trypsin to protein ratio of 1∶50 at 37°C for 18 hours. The digested samples were centrifuged at 1500×g for 10 minutes and the supernatants were collected. The protein digests were reduced with 20 mM dithiothreitol (DTT) at 60°C for 60 minutes then incubated with 25 mM iodoacetamide at room temperature in the dark for 30 minutes for cysteine alkylation. The samples were then purified with C18 columns (UltraMicroSpin Column/Vydac C18 silica, The Nest Group, Inc., Southborough, MA), dried down and stored at −20°C until mass spectrometric analysis.

### LC MS/MS analysis

An LTQ-Orbitrap hybrid mass spectrometer (Thermo Fisher Scientific, Waltham, MA) coupled with a nano-flow HPLC (Eksigent Technologies, Dublin, CA) was used in this study. A 2 µg sample of each case was injected for the mass spectrometric analysis. The samples were first loaded onto a 1.5 cm trap column (IntegraFrit 100 µm, New Objective, Woburn, MA) packed with Magic C18AQ resin (5 µm, 200 Å particles; Michrom Bioresources, Auburn, CA) with Buffer A (water with 0.1% formic acid) at a flow rate of 3 uL/minute. The peptide samples were then separated by an 27 cm analytical column (PicoFrit 75 µm, New Objective) packed with Magic C18AQ resin (5 µm, 100 Å particles; Michrom Bioresources) followed by mass spectrometric analysis. A 90-minute non-linear LC gradient was used as follows: 5% to 7% Buffer B (acetonitrile with 0.1% formic acid) versus Buffer A over 2 minutes, then to 35% over 90 minutes, then to 50% over 1 minute, hold at 50% for 9 minutes, change to 95% over 1 minute, hold at 95% for 5 minutes, drop to 5% over 1 minute and recondition at 5%. The flow rate for the peptide separation was 300 nL/minute. For MS analysis, a spray voltage of 2.25 kV was applied to the nanospray tip. The mass spectrometry experiment was performed using data-dependent acquisition with a m/z range of 400–1800, consisting of a full MS scan in the Orbitrap followed by up to 5 MS/MS spectra acquisitions in the linear ion trap using collision induced dissociation (CID). Other mass spectrometer parameters include: isolation width 2 m/z, target value 1e4, collision energy 35%, max injection time 100 ms. Lower abundance peptide ions were interrogated using dynamic exclusion (exclusion time 45 second, exclusion mass width −0.55 m/z low to 1.55 m/z high). Charge state screen was used, allowing for MS/MS of any ions with identifiable charge states +2, +3, and +4 and higher.

### Proteomics data analysis

The mass spectrometric data collected were processed using the proteomics data processing pipeline recently described [32], including the use of an Accurate Mass and Time (AMT) database for peptide and protein identification and an ion intensity based label-free approach for quantification.

#### Peptide and protein identification from tandem MS data

Raw machine output files from all MS runs were converted to mzXML files and searched with X!Tandem [33] configured with the k-score scoring algorithm [34], against version 3.69 of the human International Protein Index (IPI) database. The search parameters were as follows: enzyme, trypsin; maximum missed cleavages, 1; fixed modification, carboxamidomethylation on cysteine; potential modification, oxidization on methionine; parent monositopic mass error, 2.5 Da. Peptide identifications were assigned probability by PeptideProphet [35], and all identifications assigned probability <0.95 (estimated false discovery rate: 0.006–0.007) were discarded. The msInspect/AMT tools [36] were used to create an Accurate Mass and Time (AMT) database containing all identified peptides that were observed in at least two sample runs independently; these filtration steps ensure that AMT matching occurs with a minimum of background noise due to false identifications.

#### LC-MS feature detection, identification and peptide array creation

The LC-MS peptide features in each run were located by msInspect [37] using the default settings, and identified by matching to the AMT database. Match probabilities were calculated based on mass and RT error [38], and only matches with probability ≥0.95 were retained. Feature intensities within each sample run were normalized based on ion intensity distribution as previously described [32], [37], [39] to eliminate the effect of differences between machine runs. LC-MS data from the two replicate runs of each sample were combined into a single dataset per sample and assembled into a “peptide array” [37], in which the rows represent the peptide features and the columns describe peptide intensity. Features observed in only one replicate were retained as-is; features observed in both replicates were collapsed into a single feature and assigned the geometric mean intensity of the two constituent features.

#### Peptide abundance comparison and protein ratio quantification

All peptide identifications across the 5 samples of each disease group (PDAC, MCP and SCP) were assembled into a single pepXML file [40] with a quantitative “ratio” (where available) representing the ratio of geometric mean abundance in the disease samples to the mean abundance of matching peptides in the normal pancreas samples. Peptides observed in one of the compared groups, but not the other, were assigned a ratio of 0 or infinity accordingly, but were removed from the current analysis. For each of the two group comparisons (PDAC∶NL, MCP∶NL and SCP∶NL) a t-test compared the peptide ion intensity ratio for each peptide that was observed in at least two patient samples in each group and generated a t-statistic. For each of the two group-comparison pepXML files (PDAC∶NL, MCP∶NL and SCP∶NL), ProteinProphet [41] was used to infer the protein identifications from the corresponding identified peptides. Protein ratios were calculated using the geometric mean of all associated peptide ratios. For each protein having two or more peptides with t-scores, we performed an overrepresentation analysis, comparing the t-statistics from all of the protein's peptides with the t-statistics from all other peptide t-statistics observed using a Wilcox test. The p-values calculated from this analysis represented the probability of observing the observed data if the protein was not differentially abundant between groups. Finally, to correct for multiple testing error, q-values [42] were calculated from those p-values.

### Enrichment and pathway analysis

The enrichment analysis was performed using The Database for Annotation, Visualization and Integrated Discovery (DAVID ) v6.7 [42]. The gene symbols of differential proteins were uploaded for functional annotation analysis. Canonical pathway analysis was performed using ingenuity pathways Analysis (Ingenuity Systems, www.ingenuity.com). It identifies the pathways from the Ingenuity Pathway Analysis library of canonical pathways that are most significant to the data set. The whole data set were imported into Ingenuity and 2-fold change was used as the cutoff value for focusing on genes and/or proteins.

### Immunohistochemical (IHC) analysis

All 20 cases that were used for proteomics analysis were analyzed by IHC. Paraffin-embedded, formalin-fixed tissue blocks were sectioned at 5 µm onto charged slides and deparaffinized, processed for antigen retrieval in EDTA buffer (PH 8.0) and microwaving for 18 minutes, followed by primary antibody incubation at room temperature. The primary antibody dilutions for lumican (Novusbio), versican (Sigma Aldrich), and collagen alpha-1(XIV) chain (Sigma Aldrich) were 1∶50, 1∶200, and 1∶5, respectively. Biotinylated secondary antibody was used at 1∶500 dilution and 3,3′- Diaminobenzidine (DAB) was used as chromagen.

## Results

### Proteomics pipeline and quantitative analysis

**Figure 2:**
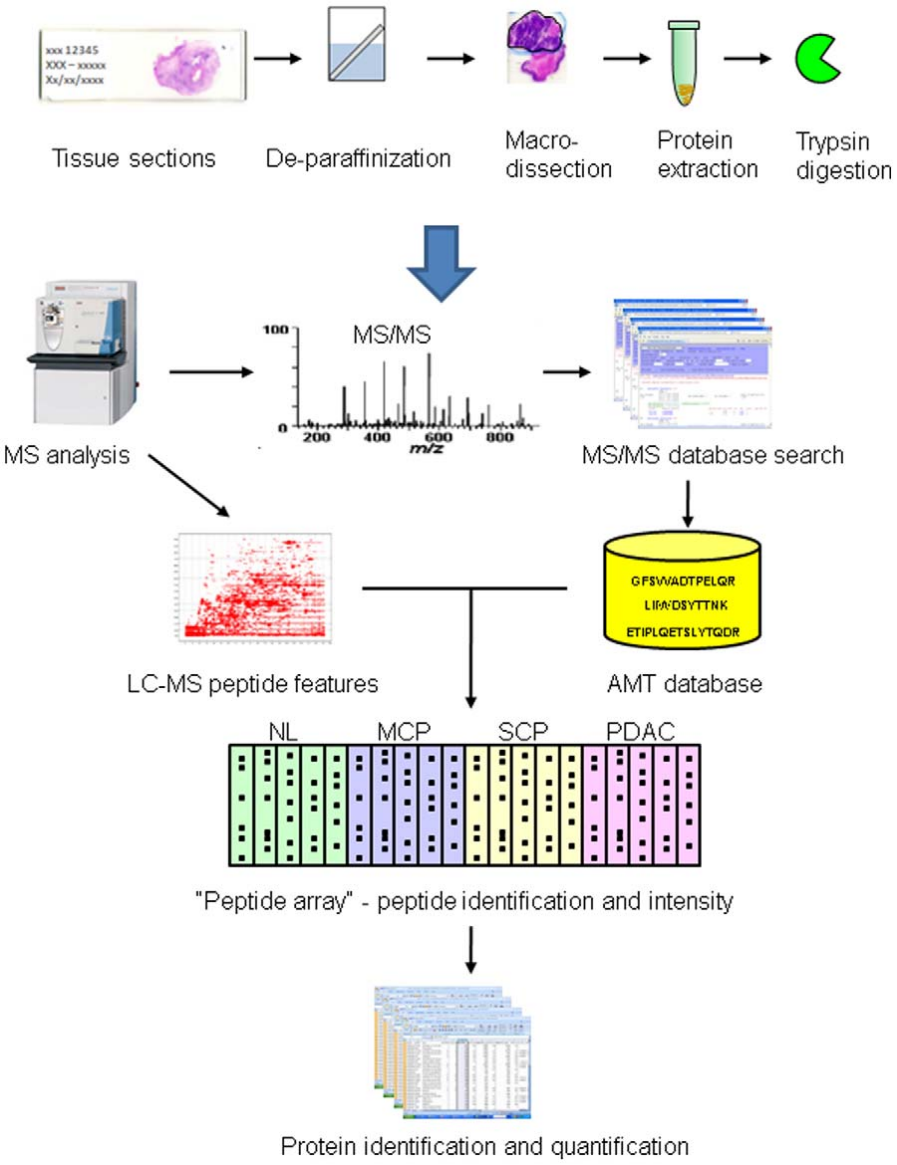
The workflow of the label-free quantitative proteomics pipeline for analyzing dissected formalin-fixed, paraffin-embedded tissue specimens.

As schematically illustrated in Figure 2, the proteomics workflow for this study consists of two major components: extraction of proteins from formalin-fixed paraffin-embedded tissue and a label-free quantitative analytical pipeline [32] for identification and quantification of proteins in diseased and control samples. Four categories of tissue specimens were analyzed: mild chronic pancreatitis (MCP), severe chronic pancreatitis (SCP), pancreatic ductal adenocarcinoma (PDAC) and normal control (NL) pancreatic tissue. In each group, 5 pathologically and clinically well-characterized samples from each group were included for the analysis. The unique peptide sequences identified by MS/MS database search in each of the original 40 runs (duplicate analysis on each samples) from all 20 samples with assigned probability ≥0.95 by PeptideProphet were used to construct the AMT database. The AMT database created from these runs contained 3,360 unique peptides observed independently in at least two different runs.

For each sample, LC-MS peptide ions were located and assigned peptide identifications using AMT database matching, and this information was combined across replicate sample runs. The msInspect located an average of 18,974 ions per run; and AMT matching identified an average of 1,620 of these ions per run with probability ≥0.95, representing 1,484 unique peptides per run. Sample replicate runs were combined; each sample contained an average of 1,881 unique sequences with high-probability AMT matches (range: 1,426–2,184).

Peptide identification and intensity information from all samples were assembled into a single “peptide array” [32], and peptide abundance ratios between the disease (MCP, SCP and PDAC) and NL groups were calculated using the geometric mean peptide intensity value from all samples in each group. The protein identifications were inferred from peptide identifications. For quantification, the ratio of a protein was obtained based on the quantification of its corresponding peptides. Where possible, q-values were calculated as described previously [32] for each protein describing the probability of observing the observed peptide information for that protein under the null hypothesis that the protein was not differentially expressed. The proteomics identification information is summarized in Table 1.

**Table 1:** Summary of proteomics identification in comparison of MCP to NL, SCP to NL and PDAC to NL.

Comparison groups	Protein identification	Quantified proteins	Peptides used for t-statistics (observed in ≥2 samples/group)	Proteins with q-value calculated
Mild chronic pancreatitis:Normal	924	799	2010	378
Severe chronic pancreatitis:Normal	953	755	1702	340
Pancreatic adenocarcinoma:Normal	996	697	1403	290

**Figure 3:**
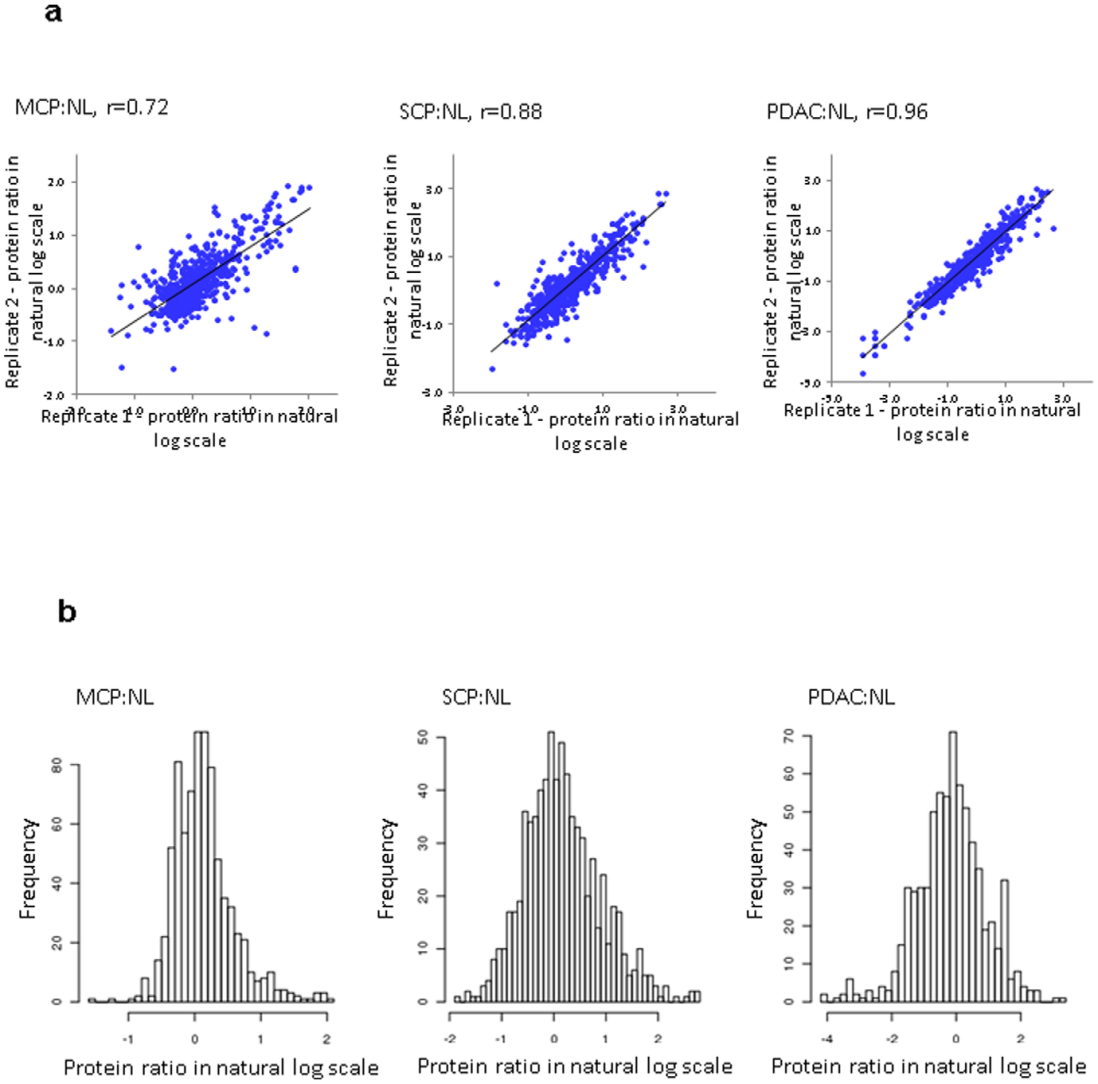
Quantitative proteomics results. a). evaluation of the reproducibility between the duplicates at protein level; b). histogram of protein ratio distribution in natural log scale.

The samples were analyzed in a random order using the same reversed-phase analytical column, LC gradient and instrument parameters. To evaluate the technical reproducibility, all the 20 samples analyzed were combined to assess the correlation of peptide intensity between two replicate runs. The ion intensity of the peptides in each analysis were plotted versus each other. A high average correlation (Spearman r = 0.96) of the peptide intensities between the replicates was observed (Figure S1), indicating good technical reproducibility. It is of note that the correlations of peptide ion intensity relationship and the protein ratio relationship should not be compared directly since they reflect very different measurement scales as previously discussed [32]. At the protein level, the variation between replicates contains the effects of variability from the entire analytic pipeline, including instrument variation, peptide intensity assessment variability, peptide identification variability, as well as computational variation that could vary from experiment to experiment (e.g., protein-level inference). Between the two replicate analyses, the correlation coefficient (Spearman r) of the replicate protein log ratio for MCP∶NL, SCP∶NL and PDAC∶NL are 0.72, 0.88, and 0.96, respectively, as shown in Figure 3a. Further, for each comparison, both replicate datasets are combined to obtain the protein quantification data, providing additional improvement on robustness for identifying the proteins that are differentially associated with the diseases. The protein identification and quantification information is provided in Table S2.

### Differential proteins in mild pancreatitis, severe pancreatitis and pancreatic adenocarcinoma

**Figure 4:**
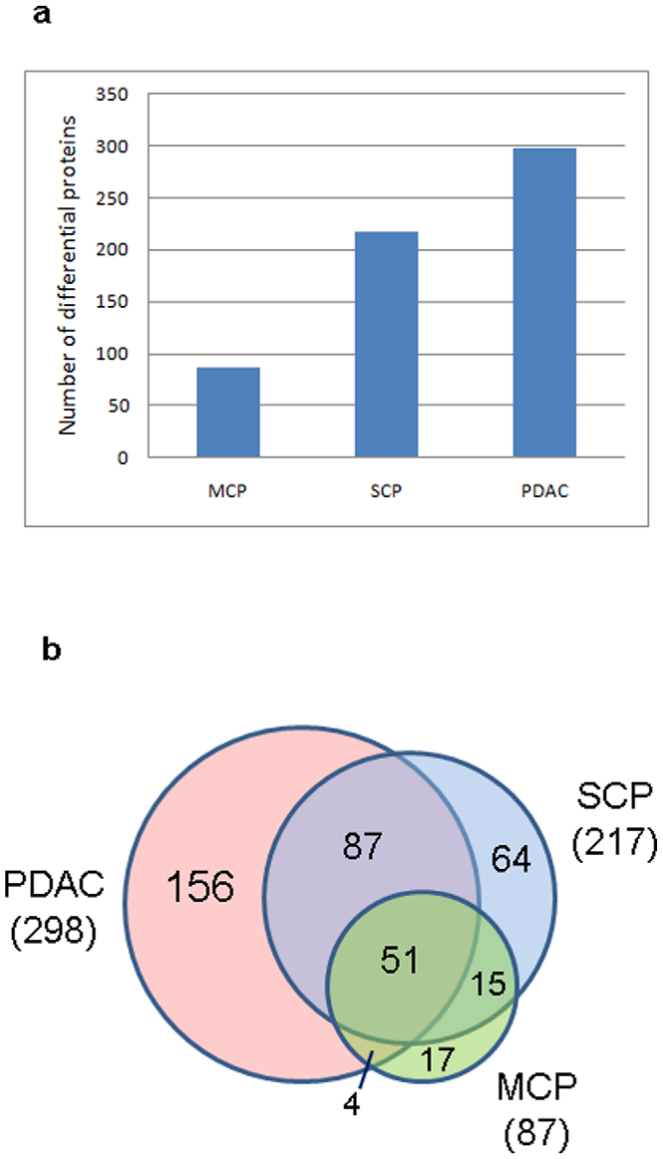
The differentially expressed proteins in MCP, SCP and PDAC. a). the number of differential proteins in MCP, SCP and PDAC using normal pancreas as a comparison. b). the overlap of the differential proteins with concurrent abundant changes in MCP, SCP and PDAC.

The comparative analysis between the disease groups and the normal control group was achieved using AMT and intensity based label-free approach as described above. To evaluate the variations within a group, the distribution of coefficient of variance (CV) of intensities of each peptide that were detected in at least two samples in each group is provided in Figure S2. For NL, MCP, SCP and PDAC, the medians are 0.36, 0.40, 0.35 and 0.37, respectively; and there is a trend toward tighter CVs as intensity increases. Figure 3b shows the distribution of protein ratios in MCP∶NL, SCP∶NL and PDAC∶NL. The proteins with a 2-fold or more change in diseases states (MCP, SCP or PDAC) versus NL are listed in Table S3. The number of the differential proteins with 2-fold or more abundant change in MCP, SCP and PDAC increased substantially as the disease severity increases, from 87 to 217 and to 298, respectively (Figure 4a). Further examination of the differential proteins indicated that some of the proteins are concurrently expressed in two or all disease categories. Figure 4b shows the comparison of the differential proteins with concurrent abundance change between each category. There are significant portions of differential proteins in MCP (51 proteins, 58%) and SCP (138 proteins, 63%) that are concurrently expressed in PDAC, consistent with our previous study [8]. Not surprisingly, the majority of the differential proteins in MCP (66 proteins, 75%) are concurrently expressed in SCP. Among these proteins, 15 proteins are exclusively expressed in both MCP and SCP, but not in PDAC (Table S3). Our current study has expanded our previous discovery and confirmed many of the differential proteins that were previously identified in pancreatic adenocarcinoma, PanIN and chronic pancreatitis from our previous proteomic studies using snap frozen tissues, [8], [17], [22] as indicated in Table S3.

### Functional clustering of differential proteins

Further analysis of the differential proteins in each category using the DAVID bioinformatics software [42] reveals the enriched biologic clusters in each disease group. For the over-expressed proteins in all 3 disease categories (MCP, SCP and PDAC), the most significant biological processes involved are extracellular matrix organization and structure organization. In addition to that, in SCP the functional enrichment analysis reveals the up-regulation of biological processes related to cell adhesion (23 proteins), response to wounding (28 proteins) and defense response (25 proteins), two processes that are fundamental to the pathogenesis of inflammation. In comparison, significant enrichment of inflammatory related processes is not observed in MCP, in which collagen fibril organization (7 proteins) is the main process that is up-regulated. In PDAC, the enrichment of up-regulation of actin filament-based process (16 proteins), cytoskeleton organization (19 proteins) and response to wounding (25 proteins) is observed.

### A proteomics view of pathophysiologic abnormalities of pancreas associated with chronic pancreatitis

#### Acinar cell secretory and related proteins – digestive enzymes and inhibitors

**Figure 5:**
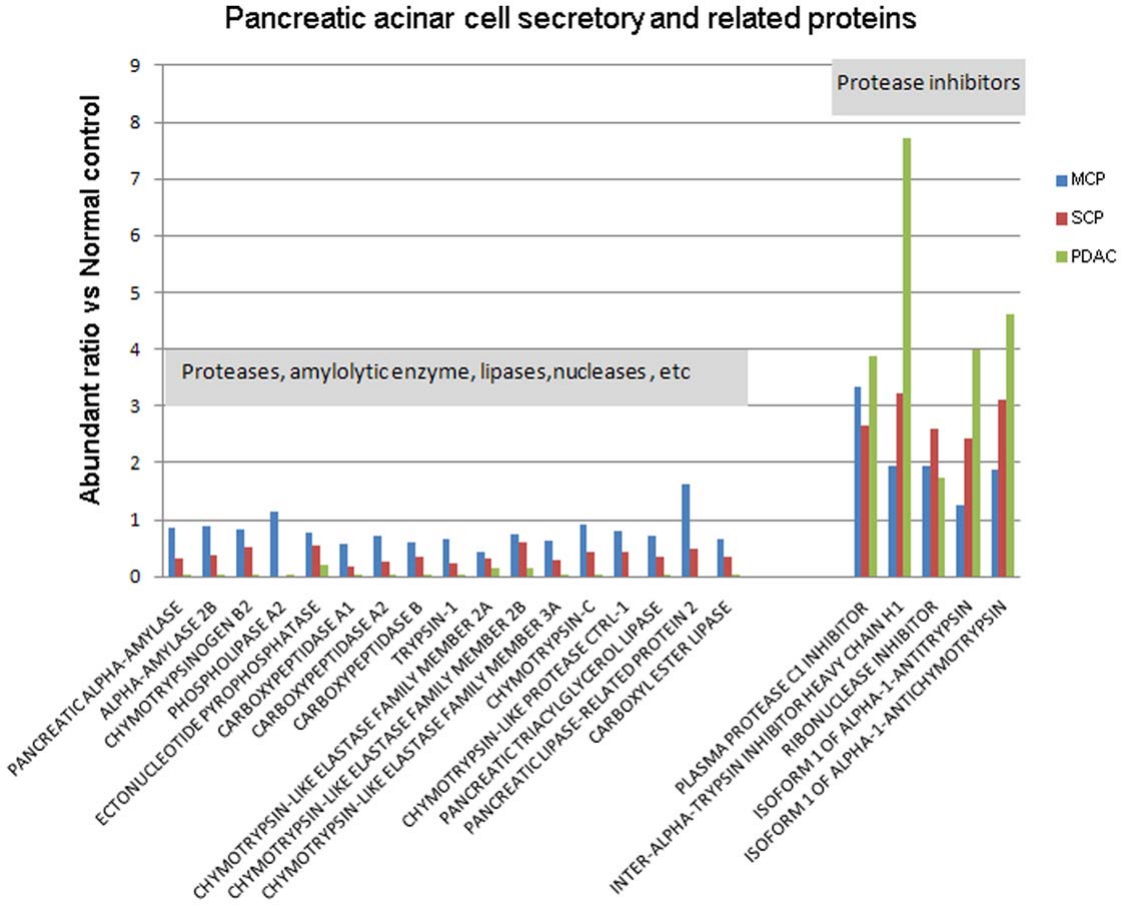
The expression of pancreatic acinar cell secretory enzymes and the related inhibitors.

Among the differential proteins identified, we observe a decrease in abundance across several enzymes that are secreted by pancreas acinar cells in SCP and PDAC compared to normal controls. These digestive enzymes include proteases, amylolytic enzymes and lipases, such as trypsin-1 (PRSS1), chymotrypsins (CTRC, CTRL, CTRB2), phospholipase A2 (PLA2G1B), ectonucleotide pyrophosphatase (ENPP1), carboxypeptidases (chronic pancreatitisA1, chronic pancreatitisA2, chronic pancreatitisB1), chymotrypsin-liked elastases (CELA2A, CELA2B, CELA3A), pancreatic triacylglycerol lipase (PnormalIP), pancreatic lipase-related protein 2 (PnormalIPRP2) and carboxyl ester lipase (CEL). The abundance changes of these proteins are illustrated in Figure 5. Using 0.5 as an abundance ratio cut-off, many of these enzymes are down-regulated (<0.5 fold) in SCP and PDAC, but have less significant changes in MCP. Another interesting group of proteins that are related to acinar cells are protease inhibitors. This group of proteins, including trypsin inhibitor (SERPINA1, ITIH1), chymotrypsin inhibitor (SERPINA3), plasma protease C1 inhibitor (SERPING1) and ribonuclease inhibitor (RNH1), all elevate significantly in the pancreatic tissue of MCP, SCP and PDAC (Figure 5), implying a possible involvement of protective mechanisms in the disease states.

#### Differential proteins associated with pancreatic fibrosis

**Figure 6:**
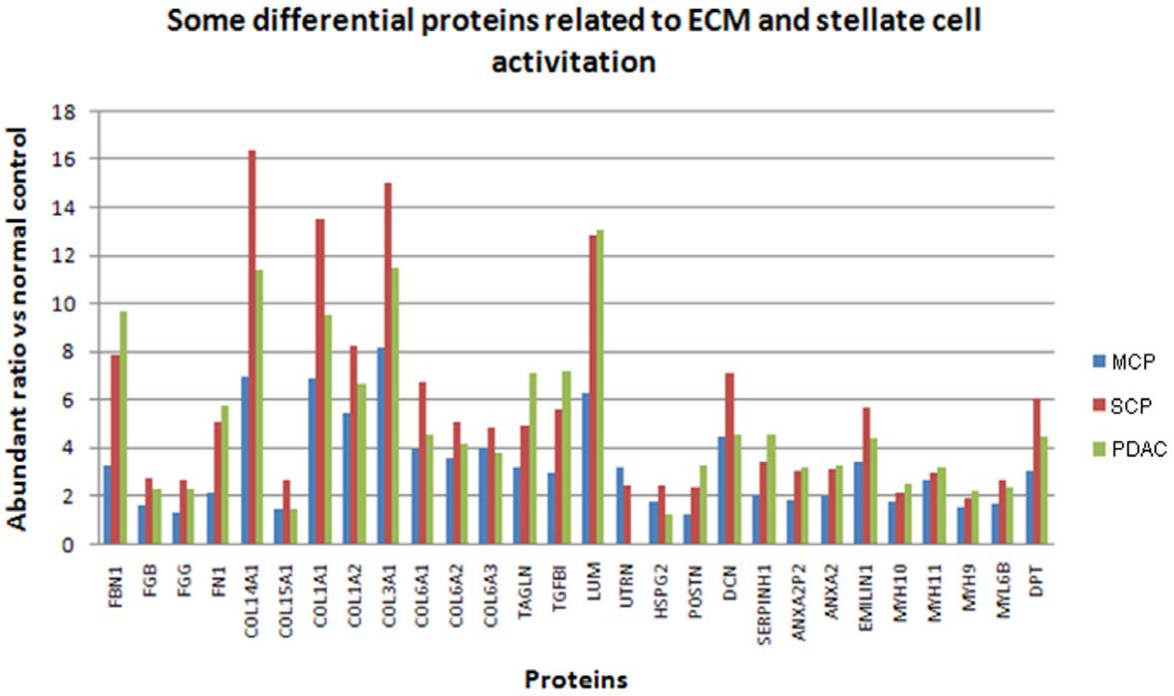
The expression of some of the differential proteins related to ECM and stellate cell activation.

Fibrosis is one of the fundamental histological abnormalities observed in chronic pancreatitis and pancreatic adenocarcinoma. One of the most enriched groups of the over-expressed proteins in MCP, SCP and PDAC includes those involved in extracellular matrix (ECM) structure and organization, which regulates fibrosis. Figure 6 shows the relative abundance change of some of the differential proteins that are related to ECM and stellate cell activation. Some of these proteins, for example, periostin (POSTN), are involved in cell mobility and neovascularization for promoting angiogenesis [43]. In SCP and PDAC, most of these proteins show more than 2-fold change in abundance compared to normal tissue. Some of these proteins are also over-expressed (>2-fold) in MCP, including fibrillin-1 (FBN1), a group of collagens (COL14A1, COL15A1, COL1A1, COL1A2, COL3A1, COL6A1, COL6A2, COL6A3), transgelin (TAGLN), transforming growth factor-beta-induced protein IG-H3 (TGFBI), lumican (LUM), utrophin (UTRN), decorin (DCN), emilin-1 (EMILIN1), myosin-11 (MYH11) and dermatopontin (DPT). As observed in Figure 6, many of these proteins similarly increase in abundance in chronic pancreatitis and pancreatic adenocarcinoma. However, their specific roles involving in the two different disease mechanisms remain to be defined.

#### Glycoproteins

Membrane proteins and secreted proteins are frequently glycoproteins. In addition, glycoproteins are important components of the ECM. Collagen, proteoglycans and other ECM specialized glycoproteins such as fibrillin, fibronectin and laminin all contain various degrees of glycosylation [44]. In our study, glycoproteins, especially those with N-linked glycosylation sites, are significantly enriched among the over-expressed proteins in MCP (40%), SCP (45%) and PDAC (40%). These glycoproteins are indicated in Table S3.

#### Inflammatory proteins

**Figure 7:**
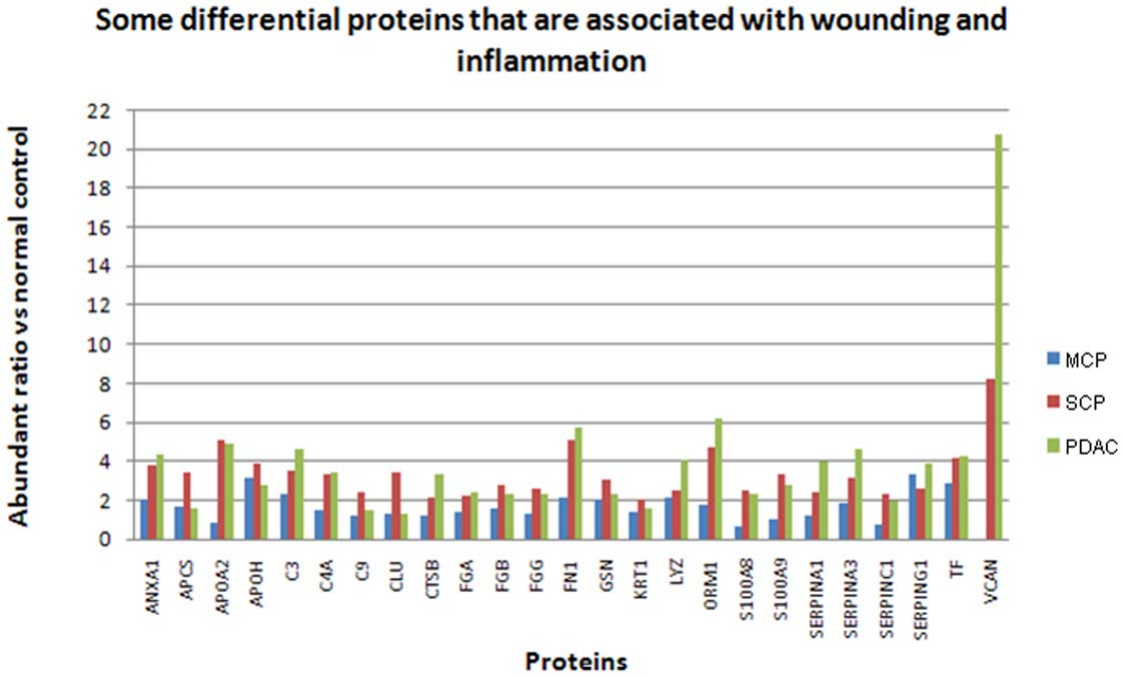
The expression of some of the differential proteins associated with wounding response and inflammation.

Inflammatory proteins and proteins that respond to wounding are particular enriched among the over-expressed proteins in SCP (28 proteins) and PDAC (25 proteins). As shown in Figure 7, many of these proteins were elevated more than 2-fold in abundance in SCP and PDAC compared to controls, but less so for MCP. In addition, a group of collagens and myosins, which are also associated with inflammation, tissue remodeling and reorganization, are over-expressed in SCP and PDAC. It is of note that some classic inflammatory markers such as TNF alpha, IFN gamma, IL-6, IL-8 were not directly detected in our samples, probably due to the low abundance of these cytokines in the pancreas tissue.

### IHC analysis of lumican, versican and Col14A1

**Figure 8:**
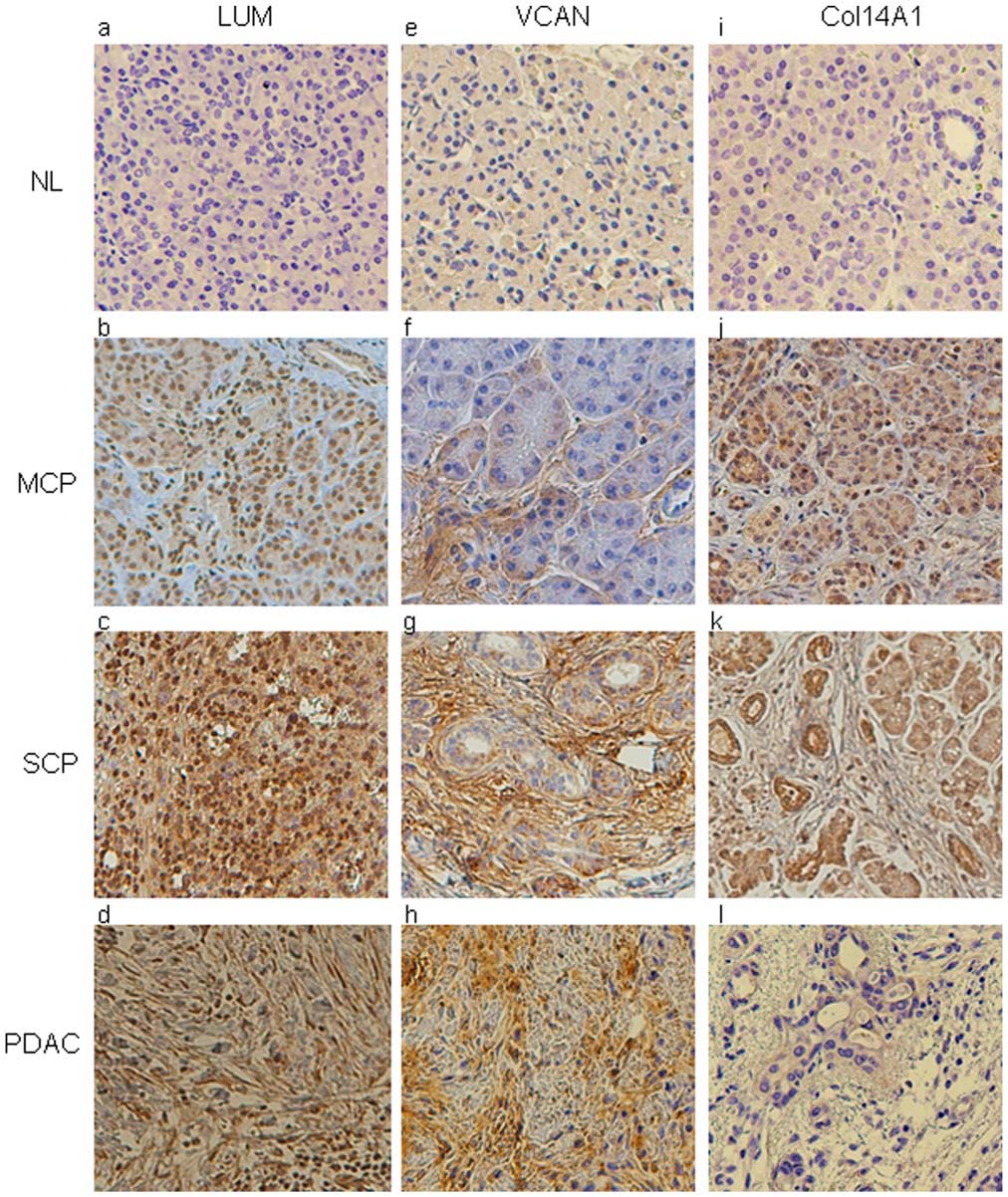
Immunohistochemical confirmation of proteomic findings in chronic pancreatitis and pancreatic adenocarcinoma. Lumican (a–d) not seen in NL (a), but is overexpressed in acinar cells of MCP (b), SCP (c), and only in the stroma of PDAC (d). Versican (e–h) is mainly over-expressed in the stroma of MCP (f), SCP (g), and PDAC (h). Col14A1 (i–l) is over-expressed in acinar and ductal cells of MCP (j), and SCP (k), but not in PDAC (l).

Three up-regulated proteins were confirmed using immunohistochemistry (IHC), as shown in Figure 8. These proteins were selected based on: 1) their high ratio in diseases versus normal; 2) their ≥2 ratio of SCP/MCP. Lumican (LUM), a proteoglycan that interacts with collagen and helps define the extracellular matrix architecture is strongly over-expressed in MCP, SCP and PDAC by 6.3 fold, 12.8 fold and 13.0 fold respectively by proteomics analysis. The up-regulation of lumican (Figure 8a–d) is detected in the acinar cells of chronic pancreatitis (both MCP and SCP) and PDAC. In addition, expression of lumican is increased in the extracellular matrix (ECM) of SCP and PDAC, but only mildly increased in MCP. Versican is a proteoglycan in the ECM that mediates cell proliferation, adhesion and migration. It is strongly over-expressed in SCP and PDAC by 8.2 fold and 20.7 fold respectively by proteomic analysis. Using IHC to identify the affected cell type in tissue sections, we found that versican is markedly increased in the ECM of SCP and PDAC (Figure 8e–h) and to a lesser extent in MCP compared to normal pancreas. In addition, increased expression of versican is observed in the acinar cells of MCP and SCP. Another ECM glycoprotein, collagen alpha-1(XIV) chain (Col14A1), is increased in MCP, SCP, and PDAC by 6.9, 16.4 and 11.4 fold respectively, by proteomics analysis. Consistent with the proteomic results, in IHC, Col14A1 is highly expressed in both the epithelial cells and acinar cells of MCP and SCP (Figure 8i–l). Although Col14A1 is a secreted protein, surprisingly, there is no staining of this protein in the ECM of chronic pancreatitis and pancreatic adenocarcinoma. One possible explanation could be that the Col14A1 protein undergoes further modification when secreted or is lost in the processes of tissue fixation of the IHC protocol.

### Canonical pathway analysis

Ingenuity Pathways Analysis was used to further explore the well-defined canonical pathways involved in MCP, SCP and PDAC. The top three canonical pathways up-regulated in MCP, include (1) pancreatic fibrosis/pancreatic stellate cell activation (p = 2.05E-04); (2) intrinsic prothrombin activation (p = 2.98E-04); and (3) acute phase response signaling (p = 7.15E-04). The top three canonical pathways in SCP were (1) acute phase response signaling (p = 4.59E-13); (2) intrinsic prothrombin activation pathway (p = 3.97E-08); and (3) extrinsic prothrombin activation pathway (p = 3.17E-05). Although the pancreatic fibrosis/stellate cell activation pathway was not ranked in the top three pathways in SCP, all of the 5 proteins involved in this pathway in MCP were also up-regulated in SCP. Comparing to MCP, acute phase response signaling is more prominent in SCP with 18 up-regulated proteins involved in this pathway. In contrast, only 5 proteins in this pathway were up-regulated in mild chronic pancreatitis. In PDAC, the top three canonical pathways were (1) citrate cycle (p = 1.17E-08), (2) propanoate metabolism (p = 5.82E-08), and (3) acute phase response signaling (p = 3.06E-07). Two of these pathways were related to metabolism, which is different from the top pathways in MCP and SCP.

## Discussion

The molecular events underlying the pathogenesis of chronic pancreatitis remains an area of great interest and uncertainty. In addition, the relationship between chronic pancreatitis, a known risk factor for pancreatic adenocarcinoma is a model system to studying the paradigm of how chronic inflammation leads to neoplastic progression. Here, we present a quantitative global proteomics study to investigate the tissue proteome alterations associated with mild and severe chronic pancreatitis and compare those findings to the proteomic profiles of normal pancreas and pancreatic cancer. The development of protein extraction techniques for fixed tissues opens a new avenue for clinical proteomics study by providing a rich source of pathologically well-characterized specimens. Using clinically well-defined formalin-fixed, paraffin-embedded pancreatic tissues, we have uncovered the concerted molecular activities and pathways that correlate with histological inflammation and fibrosis.

The analysis of the dissected histologically confirmed tissue allows the identification of differential proteins that are not only presented in ductal cells or acinar cells but also in the host microenvironment, reflecting a more complete picture of what could be underlying the pathogenesis of chronic pancreatitis. We chose a label-free pipeline to analyze the formalin-fixed, paraffin-embedded samples using high resolution Orbitrap mass spectrometer. The comparison of the replicates at both peptide and protein level indicates that the label-free pipeline is adequate for quantitative proteomics analysis in this study to identify systematic differences in protein profiles across experimental conditions.

Both chronic pancreatitis and pancreatic adenocarcinoma cause damage to the pancreas, including chronic inflammation, fibrosis, and progressive destruction of both exocrine and eventually endocrine tissue [45]. While some of these parenchymal changes can be observed histologically, the disease and its progression may induce a more profound disturbance at the molecular level. At the proteome level, we have previously found some commonality of dysregulated proteins that are shared between chronic pancreatitis and pancreatic adenocarcinoma [8]. These observations are expanded in the current study.

Using normal pancreatic tissue as a comparison, the number of differential proteins increases substantially from mild to severe chronic pancreatitis, and even more in pancreatic adenocarcinoma, possibly reflecting the changes in the proteome of pancreatic tissue with progressive severity and/or complexity. Further analysis of the differential proteins (discussed below) implied that such changes in pancreas tissue proteome might be in part associated with the changes in acinar cell abundance and increased level of fibrosis in MCP, SCP, and PDAC, as observed in histology. In addition, the presence of cancerous cells in PDAC might induce additional proteome perturbations comparing to normal pancreas and chronic pancreatitis. The significant overlap of the differential proteins between chronic pancreatitis and pancreatic adenocarcinoma suggests that chronic pancreatitis and pancreatic adenocarcinoma share many molecular features at the protein level. Alternatively they may reflect that most pancreatic adenocarcinoma patients also have chronic pancreatitis as either a primary or secondary process.

Functional clustering analysis of the differential proteins revealed that extracellular matrix organization is the most significant and common biological process among chronic pancreatitis and pancreatic adenocarcinoma. In addition, for SCP, a significant up-regulation of proteins associated with cell adhesion, response to wounding and defense response was observed. As a comparison, the significant up-regulated proteins observed in PDAC include those that play a role in actin filament-based processes, cytoskeleton organization and response to wounding. Actin filaments are a major component of the cytoskeleton. In addition to its functions in structural framework for cell shape and motile events, recent studies has shown that the cytoskeleton also plays critical roles in the regulation of various cellular processes relating to proliferation, contact inhibition, anchorage-independent cell growth, and apoptosis [46]. Indeed, cytoskeleton remodeling is an important process in cancer invasion and metastasis. It is not surprising that the proteins relating to the cytoskeleton are enriched in the pancreatic adenocarcinoma tissue. Response to wounding was enriched in both severe chronic pancreatitis and pancreatic adenocarcinoma. The enrichment of proteins involved in wounding response in SCP is consistent with the complex molecular events occurring in wound healing, including proliferation of fibroblasts, accumulation of EMC, and release of matrix metalloproteinases, growth factors and cytokines. The finding of enriched proteins related to the wounding response is not surprising in pancreatic cancer: it has long been known that tumors are like wounded tissues that do not heal.

The proteomic data evidenced that digestive enzymes were significantly decreased in SCP and PDAC tissue lesions. While the increase of pancreatic enzymes in blood, such as amylase and lipase, may be a signal of acute pancreatic injury, the significant reduction of enzymes that are secreted from acinar cells in chronic pancreatitis tissue may reflect the possible destruction and loss of acinar cell mass. The fact that amylase and lipase are generally not elevated in the blood of patients with chronic pancreatitis or pancreatic cancer would clinically fit with the proteomic findings in the tissues. Previous studies have suggested that intrapancreatic activation of trypsinogen contributes to pancreatitis and is, in part, mediated by the activity of cathepsin B (CTSB) [47]. Indeed, the abundance of this protein is associated with the severity of pancreatitis, significantly over-expressed (>2-fold) in SCP and PDAC, but not in MCP compared to normal pancreas. It is also important to note that while most of the digestive enzymes were down-regulated in severe chronic pancreatitis and pancreatic adenocarconoma, tryptase (TPSAB1) was up-regaulated in chronic pancreatitis and adenocarinoma (Table S3). Tryptase is the most abundant serine protease selectively concentrated in the secretory granules of mast cells [48] – an inflammatory cell that also involves in chronic pancreatitis [49]. The up-regulation of tryptase is consistent with a previous observation at gene level using DNA arrays [50], and may imply the increase of mast cells in chronic pancreatitis and adenocainoma.

On the other hand, enzyme inhibitors are up-regulated in these conditions, potentially reflecting protective mechanisms associated with severe chronic pancreatitis and carcinoma. It may be worthy of mentioning that a well-studied protease inhibitor, pancreatic secretory trypsin inhibitor (Serine protease inhibitor Kazal-type 1, SPINK1), whose gene mutation was frequently associated with chronic pancreatitis and pancreatic adenocarcinoma by a number of studies [51], [52], was not detected in any pancreatic tissue in our current investigation. However, this protein was indeed found up-regulated in the pancreatic juice [53] and plasma [54] of pancreatic cancer patients in our previous studies.

A significant number of proteins that are involved in the ECM structure and organization were observed up-regulated in chronic pancreatitis and pancreatic adenocarcinoma. It is known that persistent activation of pancreatic stellate cells that secret ECM promotes fibrosis [55]. Thus, the proteomic results are well correlated with the histological observation of the fibrosis promoted by pancreatic stellate cell activation. As an important component of ECM, a large number of glycoproteins were observed up-regulated in mild and severe chronic pancreatitis as well as pancreatic adenocarcinoma, implicating their role in the ECM changes that occur in these diseases and the possible pathologic implication of glycosylation. The increase in proteins associated with ECM and stellate cell activation and the presence of a large number of up-regulated tissue glycoproteins supports the concept that pancreatic fibrosis occurs in mild and severe chronic pancreatitis and cancer.

Proteins involved in wounding response and inflammation are also over-expressed in SCP and PDAC. Canonical pathway analysis also indicated that the acute phase response signaling pathway was ranked as a top canonical pathway in MCP, SCP and PDAC, suggesting the importance of this pathway in these disease processes. In addition, prothrombin activation pathway, and pancreatic fibrosis/pancreatic stellate cell activation pathway were the most significant pathways involved in chronic pancreatitis, while pathways relating to metabolism were the most significant pathway in pancreatic adenocarcinoma.

Thrombosis has long been observed as a common complication in chronic pancreatitis [56], [57], resulted from inflammatory, pseudocysts, pancreatic fibrosis and other factors. The observation of remarkable prothrombin activation in SCP may imply a possible cyclical injury process, causing further damage to the vessels that perfuse the tissue as disease progresses, if further validated. On the other hand, antithrombin III – an inhibitor of thrombin, was 2-fold increased in SCP, probably suggesting a negative feedback mechanism.

The IHC analysis of lumican, versican and Col14A1 confirmed their up-regulation in chronic pancreatitis and pancreatic adenocarcinoma, and provided additional insights on how they behave at cellular level in association with the diseases. It is of note that previous studies also observed the strong presence of lumican and versican in cancer cells [58], [59]. Lumican plays a pivotal role in cell migration and proliferation, is localized in alpha cells of islets and stromal tissues of a normal pancreas. The synthesis of this protein by cancer cell may suggest its role in pancreatic adenocarcinoma cell growth, in addition to the inflammatory process. Other studies of lumican in pancreatic adenocarcinoma suggests an increased risk for invasion and poor prognosis for patients who have strong staining in the cancer-associated stromal cells [60].

While the biological implication for many of the dysregulated proteins discovered in this study still remains unclear and warrants further investigation to unravel their role in the pathogenesis of chronic pancreatitis and pancreatic cancer, our work has revealed new molecular insights and hypothesis for better understanding mechanisms that underlie pancreatitis and pancreatic adenocarcinoma. Moreover, the differential proteins identified could serve as potential candidates for biomarker development to improve the diagnosis and treatment of pancreatic diseases.

## Supporting Information

Figure S1 **Scatter plot of the normalized peptide intensities (in natural log transformation) between the duplicate runs.**
(PDF)

Figure S2 **These charts describe the peptide ion intensity variation between samples within each sample group: normal control, mild chronic pancreatitis, severe chronic pancreatitis and pancreatic adenocarcinoma.** Each plot is a histogram of the coefficients of variation (CVs) (in natural log scale) of peptide intensity for all peptide ions that appear in at least two of the five samples in the group.
(PDF)

Table S1 **The patient demographic information.**
(PDF)

Table S2 **Summary of protein identification and quantification.**
(PDF)

Table S3 **The proteins with ≥2-fold change in abundance in mild chronic pancreatitis (MCP), severe chronic pancreatitis (SCP) and pancreatic ductal adenocarcinoma (PDAC) compared to normal pancreas (NL).**
(PDF)
