# Supplementary material for: Proteomics Portrait of Archival Lesions of Chronic Pancreatitis
Source: PLoS One. 2011 Nov 23;6(11):e27574. doi: 10.1371/journal.pone.0027574 (PMC3223181; doi:10.1371/journal.pone.0027574)

Supplemental Figure 1. Scatter plot of the normalized peptide intensities (in natural log transformation) between the duplicate runs.

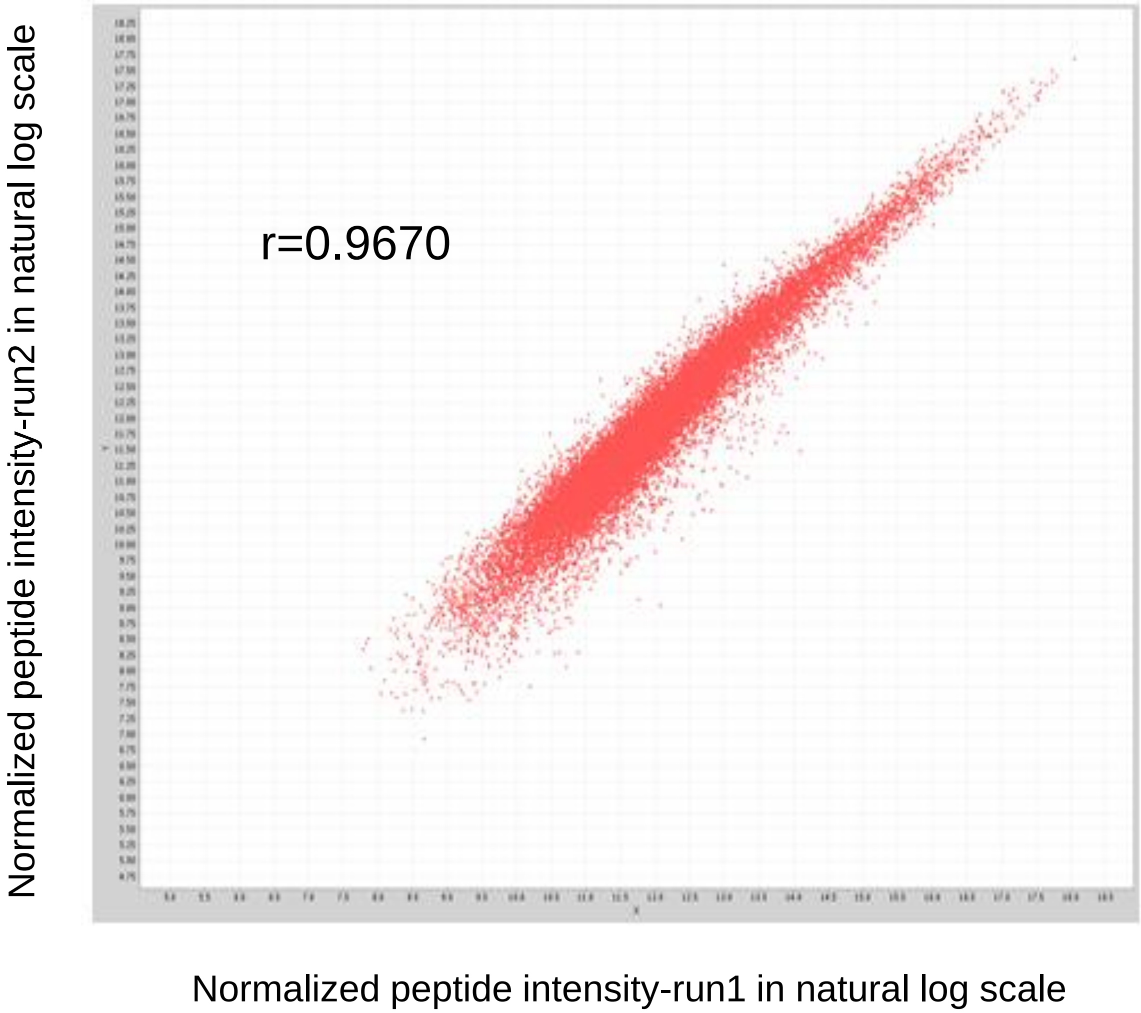

Supplement: Figure S1 — Scatter plot of the normalized peptide intensities (in natural log transformation) between the duplicate runs. (PDF) [file pone.0027574.s001.pdf]
