# Supplementary material for: Proteomics Portrait of Archival Lesions of Chronic Pancreatitis
Source: PLoS One. 2011 Nov 23;6(11):e27574. doi: 10.1371/journal.pone.0027574 (PMC3223181; doi:10.1371/journal.pone.0027574)

Supplemental Figure 2. These charts describe the peptide ion intensity variation between samples within each sample group: normal control, mild chronic pancreatitis, severe chronic pancreatitis and pancreatic adenocarcinoma. Each plot is a histogram of the coefficients of variation (CVs) (in natural log scale) of peptide intensity for all peptide ions that appear in at least two of the five samples in the group.

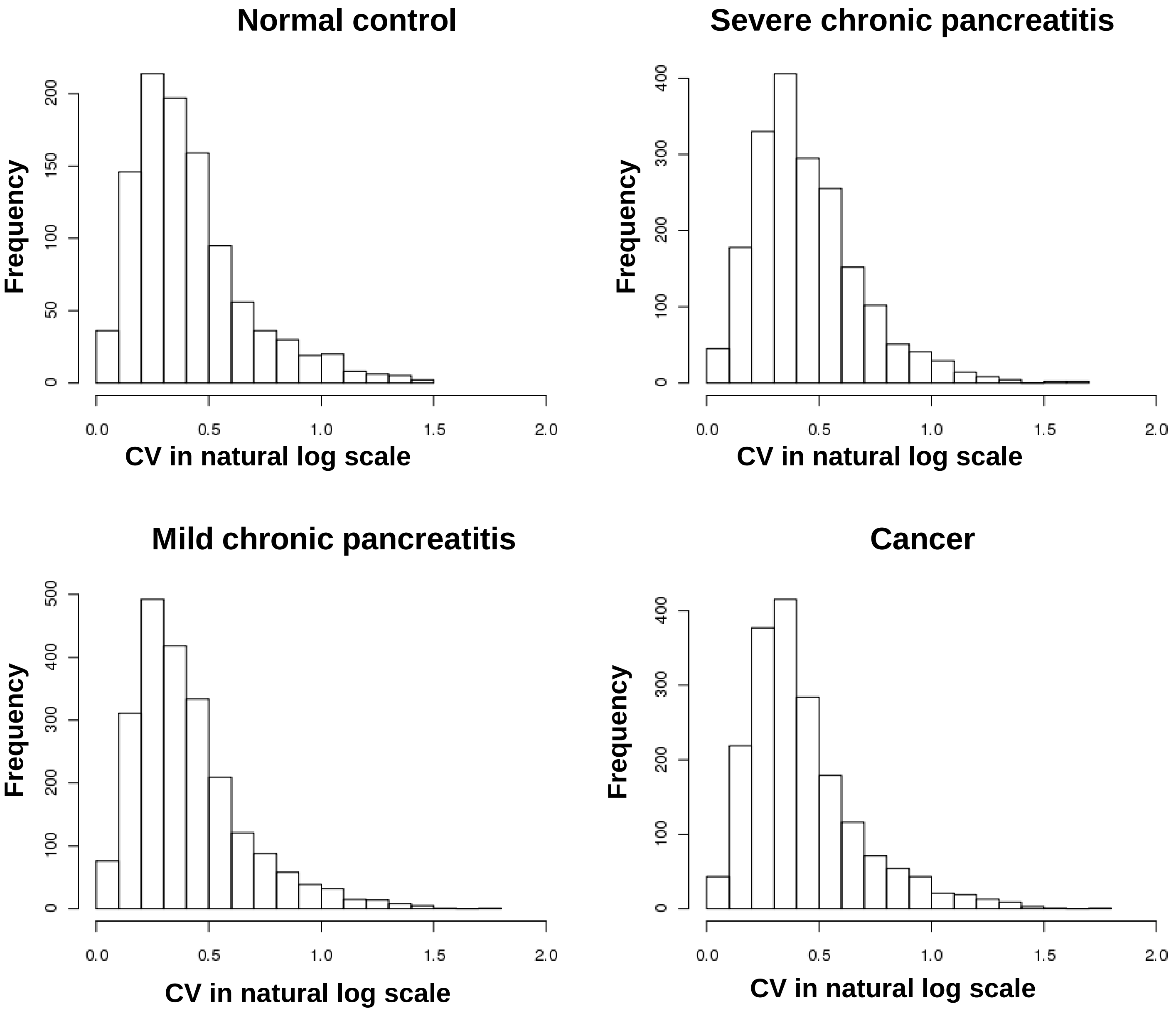

Supplement: Figure S2 — These charts describe the peptide ion intensity variation between samples within each sample group: normal control, mild chronic pancreatitis, severe chronic pancreatitis and pancreatic adenocarcinoma. Each plot is a histogram of the coefficients of variation (CVs) (in natural log scale) of peptide intensity for all peptide ions that appear in at least two of the five samples in the group. (PDF) [file pone.0027574.s002.pdf]
