# Supplementary material for: Proteomics Portrait of Archival Lesions of Chronic Pancreatitis
Source: PLoS One. 2011 Nov 23;6(11):e27574. doi: 10.1371/journal.pone.0027574 (PMC3223181; doi:10.1371/journal.pone.0027574)
Supplement: Table S1 — The patient demographic information. (PDF) [file pone.0027574.s003.pdf]

Supplemental Table 1 - The patient demographic information

Normal Control			Mild pancreatitis 1°			Severe pancreatitis 1°			Pancreatic adenocarcinoma		
**age**	**gender**	**Ethnicity**	**age**	**gender**	**Ethnicity**	**age**	**gender**	**Ethnicity**	**age**	**gender**	**Ethnicity**
77	M	white	69	M	white	55	F	white	45	F	white
69	F	white	59	M	hispanic	46	F	white	69	F	white
57	M	white	49	F	hispanic	77	M	white	62	F	white
50	F	white	39	M	white	67	F	white	67	M	white
43	F	african american	74	F	white	61	M	white	60	M	white
