# Supplementary material for: Proteomics Portrait of Archival Lesions of Chronic Pancreatitis
Source: PLoS One. 2011 Nov 23;6(11):e27574. doi: 10.1371/journal.pone.0027574 (PMC3223181; doi:10.1371/journal.pone.0027574)
Supplement: Table S2 — Summary of protein identification and quantification. (PDF) [file pone.0027574.s004.pdf]

Supplemental table 2. Summary of protein identification and quantification

protein IPI #	gene symbol	Protein	MCP/NL ratio	MCP/NL stdev	MCP/NL total peptide	MCP/NL q value	SCP/NL ratio	SCP/NL stdev	SCP/NL total peptide	SCP/NL q value	PDAC/NL ratio	PDAC/NL stdev	PDAC/NL total peptide	PDAC/NL q value
IPI00000015;IPI00012341;IPI00012345;IPI00215879;IPI00413654;IPI00556297;IPI00908618;IPI00915825	SFRS4;SFRS5;SFRS6	SPLICING FACTOR, ARGININE/SERINE-RICH 4.;"ISOFORM SRP40-1 OF SPLICING FACTOR, ARGININE/SERINE-RICH 5.";"ISOFORM SRP55-1 OF SPLICING FACTOR, ARGININE/SERINE-RICH 6.";"ISOFORM SRP55-3 OF SPLICING FACTOR, ARGININE/SERINE-RICH 6.";"ISOFORM SRP40-4 OF SPLICING FACTOR, ARGININE/SERINE-RICH 5.";ARGININE/SERINE-RICH SPLICING FACTOR 6 VARIANT (FRAGMENT).;"CDNA FLJ50118, HIGHLY SIMILAR TO SPLICING FACTOR, ARGININE/SERINE-RICH 4.";CS0DF038YO05 VARIANT (FRAGMENT).	**1.05**	0.00	1		**1.31**	0.00	1		**0.62**	0.00	1	
IPI00000105;IPI00910019	MVP	MAJOR VAULT PROTEIN.;"CDNA FLJ53437, HIGHLY SIMILAR TO MAJOR VAULT PROTEIN."	**1.53**	0.52	6		**2.49**	0.75	7		**2.04**	0.63	11	
IPI00000155;IPI00644210;IPI00647134;IPI00647648;IPI00910934	GLYR1	ISOFORM 1 OF PUTATIVE OXIDOREDUCTASE GLYR1.;ISOFORM 4 OF PUTATIVE OXIDOREDUCTASE GLYR1.;ISOFORM 3 OF PUTATIVE OXIDOREDUCTASE GLYR1.;ISOFORM 2 OF PUTATIVE OXIDOREDUCTASE GLYR1.;ISOFORM 5 OF PUTATIVE OXIDOREDUCTASE GLYR1.			1		**1.05**	0.00	1				1	
IPI00000335	HINT2	HISTIDINE TRIAD NUCLEOTIDE-BINDING PROTEIN 2.	**1.02**	0.00	1		**0.64**	0.00	1				1	
IPI00000477;IPI00005161;IPI00927156	ARPC2	PNAS-139.;ACTIN-RELATED PROTEIN 2/3 COMPLEX SUBUNIT 2.;19 KDA PROTEIN.							1				1	
IPI00000494	RPL5	60S RIBOSOMAL PROTEIN L5.	**0.66**	0.10	3	0.112	**0.37**	0.00	3		**0.40**	0.00	3	
IPI00000513;IPI00025861;IPI00744889;IPI00911003	CDH1	CADHERIN 1, TYPE 1, E-CADHERIN (EPITHELIAL), ISOFORM CRA_C.;CADHERIN-1.;E-CADHERIN.;"CDNA FLJ60313, HIGHLY SIMILAR TO EPITHELIAL-CADHERIN."	**0.94**	0.00	1		**1.04**	0.00	1		**0.64**	0.00	1	
IPI00000581;IPI00939174	OTUB1	CDNA FLJ56307, HIGHLY SIMILAR TO UBIQUITIN THIOESTERASE PROTEIN OTUB1.;ISOFORM 1 OF UBIQUITIN THIOESTERASE OTUB1.			1				1				1	
IPI00000690;IPI00157908	AIFM1	ISOFORM 1 OF APOPTOSIS-INDUCING FACTOR 1, MITOCHONDRIAL.;"ISOFORM 3 OF APOPTOSIS-INDUCING FACTOR 1, MITOCHONDRIAL."	**1.17**	0.09	5	0.405	**0.95**	0.20	5				5	
IPI00000695;IPI00305551	GNA14;GNA11	GUANINE NUCLEOTIDE-BINDING PROTEIN SUBUNIT ALPHA-14.;GUANINE NUCLEOTIDE-BINDING PROTEIN SUBUNIT ALPHA-11.			1				1					
IPI00000760;IPI00645821;IPI00646524;IPI00647695;IPI00797462	DDAH2	N(G),N(G)-DIMETHYLARGININE DIMETHYLAMINOHYDROLASE 2.;DIMETHYLARGININE DIMETHYLAMINOHYDROLASE 2.			1				2				1	
IPI00000787;IPI00170899;IPI00796431;IPI00893267;IPI00893456;IPI00894395	PSMB9	ISOFORM LMP2.L OF PROTEASOME SUBUNIT BETA TYPE-9.;ISOFORM LMP2.S OF PROTEASOME SUBUNIT BETA TYPE-9.;"PROTEASOME (PROSOME, MACROPAIN) SUBUNIT, BETA TYPE, 9.";PROTEASOME SUBUNIT BETA TYPE.	**0.72**	0.00	1		**1.65**	0.00	1		**0.93**	0.00	1	
IPI00000816	YWHAE	14-3-3 PROTEIN EPSILON.	**0.96**	0.11	13	0.185	**0.82**	0.08	13	0.354	**0.71**	0.10	14	0.626
IPI00000861;IPI00386803;IPI00883946;IPI00909481;IPI00909678;IPI00909939;IPI00952738	LASP1	ISOFORM 1 OF LIM AND SH3 DOMAIN PROTEIN 1.;ISOFORM 2 OF LIM AND SH3 DOMAIN PROTEIN 1.;19 KDA PROTEIN.;"CDNA FLJ58065, HIGHLY SIMILAR TO LIM AND SH3 DOMAIN PROTEIN 1.";"CDNA FLJ51834, HIGHLY SIMILAR TO LIM AND SH3 DOMAIN PROTEIN 1.";"CDNA FLJ52195, HIGHLY SIMILAR TO LIM AND SH3 DOMAIN PROTEIN 1."	**0.97**	0.00	1		**1.56**	0.00	1		**1.31**	0.00	1	
IPI00000874;IPI00640741;IPI00641244	PRDX1	PEROXIREDOXIN-1.;19 KDA PROTEIN.;11 KDA PROTEIN.	**0.97**	0.11	7	0.507	**0.80**	0.17	7	0.394	**0.45**	0.13	7	0.242
IPI00000875;IPI00937615	EEF1G;TUT1	CDNA FLJ56389, HIGHLY SIMILAR TO ELONGATION FACTOR 1-GAMMA.;ELONGATION FACTOR 1-GAMMA.	**1.05**	0.16	10	0.202	**0.61**	0.11	9	0.081	**0.42**	0.09	10	0.125
IPI00000877;IPI00922838	HYOU1	HYPOXIA UP-REGULATED PROTEIN 1.;"CDNA FLJ56074, HIGHLY SIMILAR TO 150 KDA OXYGEN-REGULATED PROTEIN."	**1.13**	0.14	18	0.507	**0.73**	0.14	18	0.090	**0.22**	0.02	18	0.054
IPI00000948;IPI00927538	TBL2	TRANSDUCIN BETA-LIKE PROTEIN 2.;"CDNA FLJ55528, HIGHLY SIMILAR TO TRANSDUCIN BETA-LIKE 2 PROTEIN."	**0.80**	0.00	3		**0.94**	0.00	3		**0.84**	0.00	3	
IPI00000982;IPI00795899	PPY	PANCREATIC PROHORMONE.;11 KDA PROTEIN.			1								1	
IPI00001159	GCN1L1	TRANSLATIONAL ACTIVATOR GCN1.	**0.85**	0.00	2		**0.80**	0.00	2				2	
IPI00001466;IPI00854834;IPI00917887;IPI00940934	EML4	ECHINODERM MICROTUBULE-ASSOCIATED PROTEIN-LIKE 4.;ECHINODERM MICROTUBULE ASSOCIATED PROTEIN LIKE 4 ISOFORM B.;PUTATIVE UNCHARACTERIZED PROTEIN EML4.;109 KDA PROTEIN.	**1.15**	0.00	2				2				2	
IPI00001508	INS;INS-IGF2	INSULIN.	**1.06**	0.09	3		**2.20**	0.38	3	0.373	**0.51**	0.03	3	
IPI00001539;IPI00873829	ACAA2	3-KETOACYL-COA THIOLASE, MITOCHONDRIAL.;PUTATIVE UNCHARACTERIZED PROTEIN ACAA2 (FRAGMENT).	**1.34**	0.24	2		**0.54**	0.00	2		**0.70**	0.00	2	
IPI00001624	GAST	GASTRIN.			1									
IPI00001639	KPNB1	IMPORTIN SUBUNIT BETA-1.	**1.36**	0.15	4	0.219	**1.61**	0.20	4	0.266	**1.13**	0.18	5	0.329
IPI00001661;IPI00747309;IPI00787306;IPI00925317;IPI00925570;IPI00925791;IPI00926548;IPI00927389	RCC1;RCC1;SNHG3-RCC1	REGULATOR OF CHROMOSOME CONDENSATION 1 ISOFORM A.;REGULATOR OF CHROMOSOME CONDENSATION.;REGULATOR OF CHROMOSOME CONDENSATION 1 ISOFORM B.;29 KDA PROTEIN.;PROTEIN.;30 KDA PROTEIN.;24 KDA PROTEIN.	**1.19**	0.00	1		**1.34**	0.00	1		**1.36**	0.00	1	
IPI00001699;IPI00221360;IPI00221362	PYCARD	ISOFORM 1 OF APOPTOSIS-ASSOCIATED SPECK-LIKE PROTEIN CONTAINING A CARD.;ISOFORM 2 OF APOPTOSIS-ASSOCIATED SPECK-LIKE PROTEIN CONTAINING A CARD.;ISOFORM 3 OF APOPTOSIS-ASSOCIATED SPECK-LIKE PROTEIN CONTAINING A CARD.			1				1				1	
IPI00001734;IPI00219478;IPI00930609;IPI00941072	C8orf62;PSAT1;C8orf62;PSAT1	PHOSPHOSERINE AMINOTRANSFERASE.;ISOFORM 2 OF PHOSPHOSERINE AMINOTRANSFERASE.;ISOFORM 1 OF PHOSPHOSERINE AMINOTRANSFERASE.;45 KDA PROTEIN.	**0.73**	0.15	3	0.185	**0.67**	0.03	3		**0.27**	0.00	3	
IPI00002243;IPI00339373;IPI00855794	GGT5	ISOFORM 1 OF GAMMA-GLUTAMYLTRANSFERASE 5.;ISOFORM 2 OF GAMMA-GLUTAMYLTRANSFERASE 5.;GAMMA-GLUTAMYLTRANSFERASE 5 ISOFORM A.	**2.23**	0.00	3		**5.01**	0.00	3		**1.59**	0.00	2	
IPI00002280	PCSK1N	PROSAAS.	**2.59**	0.00	2		**5.94**	0.43	3		**2.45**	0.00	2	
IPI00002406;IPI00554618	BCAM	BASAL CELL ADHESION MOLECULE.;BASAL CELL ADHESION MOLECULE ISOFORM 2 PRECURSOR.	**1.08**	0.00	1		**0.78**	0.00	2				1	
IPI00002412;IPI00514424;IPI00514996;IPI00908718	PPT1	PALMITOYL-PROTEIN THIOESTERASE 1.;PALMITOYL-PROTEIN THIOESTERASE 1 ISOFORM 2 PRECURSOR.	**1.68**	0.00	1				1				1	
IPI00002459	ANXA6	ANNEXIN VI ISOFORM 2.	**1.36**	0.10	9	0.028	**1.97**	0.18	9	0.003	**1.28**	0.09	11	0.300
IPI00002460;IPI00021771;IPI00953297	ANXA7	PUTATIVE UNCHARACTERIZED PROTEIN ANXA7.;ISOFORM 2 OF ANNEXIN A7.	**0.95**	0.00	1		**1.40**	0.00	1		**1.11**	0.00	1	
IPI00002491;IPI00010554;IPI00010555;IPI00064591;IPI00186452;IPI00306791;IPI00334908;IPI00383430;IPI00426075;IPI00454950;IPI00454951;IPI00513917;IPI00514261;IPI00514576;IPI00646306;IPI00654848;IPI00922621	SORBS1	ISOFORM 9 OF SORBIN AND SH3 DOMAIN-CONTAINING PROTEIN 1.;ISOFORM 3 OF SORBIN AND SH3 DOMAIN-CONTAINING PROTEIN 1.;ISOFORM 2 OF SORBIN AND SH3 DOMAIN-CONTAINING PROTEIN 1.;ISOFORM 8 OF SORBIN AND SH3 DOMAIN-CONTAINING PROTEIN 1.;ISOFORM 10 OF SORBIN AND SH3 DOMAIN-CONTAINING PROTEIN 1.;SORBIN AND SH3 DOMAIN-CONTAINING PROTEIN 1.;ISOFORM 6 OF SORBIN AND SH3 DOMAIN-CONTAINING PROTEIN 1.;ISOFORM 7 OF SORBIN AND SH3 DOMAIN-CONTAINING PROTEIN 1.;PUTATIVE UNCHARACTERIZED PROTEIN DKFZP686N08224.;ISOFORM 4 OF SORBIN AND SH3 DOMAIN-CONTAINING PROTEIN 1.;ISOFORM 5 OF SORBIN AND SH3 DOMAIN-CONTAINING PROTEIN 1.;SORBIN AND SH3 DOMAIN CONTAINING 1 ISOFORM 4.;SORBIN AND SH3 DOMAIN CONTAINING 1.;PUTATIVE UNCHARACTERIZED PROTEIN SORBS1.;SORBIN AND SH3 DOMAIN CONTAINING 1 ISOFORM 1.;"CDNA FLJ60072, HIGHLY SIMILAR TO HOMO SAPIENS SORBIN AND SH3 DOMAIN CONTAINING 1 (SORBS1), TRANSCRIPT VARIANT 6, MRNA.";"CDNA FLJ54732, MODERATELY SIMILAR TO SORBIN AND SH3 DOMAIN-CONTAINING PROTEIN 1."							1					
IPI00002496;IPI00030920	GMPPB	ISOFORM 2 OF MANNOSE-1-PHOSPHATE GUANYLTRANSFERASE BETA.;ISOFORM 1 OF MANNOSE-1-PHOSPHATE GUANYLTRANSFERASE BETA.			1				1				1	
IPI00002535	FKBP2	FK506-BINDING PROTEIN 2.	**0.60**	0.00	2		**0.55**	0.00	2		**0.21**	0.00	2	
IPI00002790;IPI00555723	SEL1L	ISOFORM 1 OF PROTEIN SEL-1 HOMOLOG 1.;ISOFORM 2 OF PROTEIN SEL-1 HOMOLOG 1.	**0.62**	0.06	4				4				4	
IPI00003031;IPI00442223;IPI00551022	ISOC2	ISOFORM 2 OF ISOCHORISMATASE DOMAIN-CONTAINING PROTEIN 2, MITOCHONDRIAL.;ISOCHORISMATASE DOMAIN CONTAINING 2 ISOFORM 3.;"ISOFORM 1 OF ISOCHORISMATASE DOMAIN-CONTAINING PROTEIN 2, MITOCHONDRIAL."			1									
IPI00003348;IPI00026268;IPI00029143;IPI00386304;IPI00639998;IPI00640462;IPI00640949;IPI00790302;IPI00790896;IPI00797969;IPI00925530	GNB2;GNB1;GNB3	GUANINE NUCLEOTIDE-BINDING PROTEIN G(I)/G(S)/G(T) SUBUNIT BETA-2.;GUANINE NUCLEOTIDE-BINDING PROTEIN G(I)/G(S)/G(T) SUBUNIT BETA-1.;GUANINE NUCLEOTIDE-BINDING PROTEIN G(I)/G(S)/G(T) SUBUNIT BETA-3.;GNB3 PROTEIN.;"GUANINE NUCLEOTIDE BINDING PROTEIN (G PROTEIN), BETA POLYPEPTIDE 1.";28 KDA PROTEIN.;26 KDA PROTEIN.;17 KDA PROTEIN.;32 KDA PROTEIN.	**1.16**	0.00	1		**1.57**	0.00	1	0.159			1	
IPI00003362	HSPA5	HSPA5 PROTEIN.	**0.75**	0.10	36	0.000	**0.46**	0.10	34	0.000	**0.21**	0.03	34	0.000
IPI00003377;IPI00010204;IPI00744364;IPI00843996;IPI00916448;IPI00939530	SFRS7;SFRS3	ISOFORM 1 OF SPLICING FACTOR, ARGININE/SERINE-RICH 7.;"SPLICING FACTOR, ARGININE/SERINE-RICH 3.";"CDNA FLJ59182, HIGHLY SIMILAR TO SPLICING FACTOR, ARGININE/SERINE-RICH 7.";"SPLICING FACTOR, ARGININE/SERINE-RICH 3, ISOFORM CRA_A.";27 KDA PROTEIN.;"SPLICING FACTOR, ARGININE/SERINE-RICH 7, 35KDA, ISOFORM CRA_A."	**1.25**	0.03	2	0.497	**1.19**	0.05	2		**1.05**	0.12	2	
IPI00003411	PTGIS	PROSTACYCLIN SYNTHASE.			1				1				4	
IPI00003482	DECR1	2,4-DIENOYL-COA REDUCTASE, MITOCHONDRIAL.	**1.04**	0.04	2				2				1	
IPI00003527;IPI00794755;IPI00902652	SLC9A3R1	NA(+)/H(+) EXCHANGE REGULATORY COFACTOR NHE-RF1.;26 KDA PROTEIN.;"CDNA FLJ37148 FIS, CLONE BRACE2025333, HIGHLY SIMILAR TO HOMO SAPIENS NA+/H+ EXCHANGE REGULATORY CO-FACTOR (NHERF) MRNA."			1									

IPI00003799;IPI00644697	HEBP2	ISOFORM 2 OF HEME-BINDING PROTEIN 2.;HEBP2 PROTEIN (FRAGMENT).	**0.82**	0.00	1		**0.91**	0.00	1		**0.87**	0.00	1	
IPI00003815;IPI00793767;IPI00794402;IPI00796541	ARHGDIA	RHO GDP-DISSOCIATION INHIBITOR 1.;22 KDA PROTEIN.;28 KDA PROTEIN.;26 KDA PROTEIN.	**1.04**	0.03	3		**1.06**	0.00	3		**0.87**	0.04	3	
IPI00003817;IPI00791712;IPI00792537;IPI00792758	ARHGDIB	RHO GDP-DISSOCIATION INHIBITOR 2.;13 KDA PROTEIN.;21 KDA PROTEIN.	**1.29**	0.00	1				1		**2.20**	0.00	1	
IPI00003865;IPI00037070;IPI00939595	HSPA8	ISOFORM 1 OF HEAT SHOCK COGNATE 71 KDA PROTEIN.;54 KDA PROTEIN.;ISOFORM 2 OF HEAT SHOCK COGNATE 71 KDA PROTEIN.	**0.99**	0.09	19	0.347	**0.96**	0.18	20	0.463	**0.69**	0.18	20	0.258
IPI00003881;IPI00908896	HNRNPF;HNRNPH1	HETEROGENEOUS NUCLEAR RIBONUCLEOPROTEIN F.;"CDNA FLJ54533, HIGHLY SIMILAR TO HETEROGENEOUS NUCLEAR RIBONUCLEOPROTEIN H."	**1.15**	0.04	3	0.442	**1.08**	0.04	2	0.578	**0.77**	0.00	3	0.655
IPI00003918	RPL4	60S RIBOSOMAL PROTEIN L4.	**0.67**	0.07	10	0.057	**0.53**	0.12	10	0.073	**0.23**	0.03	9	0.069
IPI00003935;IPI00152785;IPI00166293;IPI00220403;IPI00515061	HIST2H2BE;HIST1H2BO;HIST3H2BB;HIST1H2BB;HIST1H2BJ	HISTONE H2B TYPE 2-E.;HISTONE H2B TYPE 1-O.;HISTONE H2B TYPE 3-B.;HISTONE H2B TYPE 1-B.;HISTONE H2B TYPE 1-J.	**1.10**	0.17	5	0.506	**1.17**	0.28	5	0.610	**0.79**	0.20	5	0.623
IPI00003949;IPI00376844;IPI00874051	UBE2N;UBE2NL	UBIQUITIN-CONJUGATING ENZYME E2 N.;PUTATIVE UBIQUITIN-CONJUGATING ENZYME E2 N-LIKE.;UBIQUITIN CARRIER PROTEIN (FRAGMENT).			1				1				2	
IPI00003951;IPI00377045;IPI00743994;IPI00790353;IPI00790908;IPI00895830	LAMA3	ISOFORM 1 OF LAMININ SUBUNIT ALPHA-3.;LAMININ ALPHA-3 CHAIN VARIANT 1.;LAMININ ALPHA 3 SUBUNIT ISOFORM 3.;ISOFORM 2 OF LAMININ SUBUNIT ALPHA-3.;LAMININ ALPHA 3 SUBUNIT ISOFORM 2.;LAMININ ALPHA 3 SUBUNIT ISOFORM 4.											1	
IPI00003968	NDUFA9	NADH DEHYDROGENASE [UBIQUINONE] 1 ALPHA SUBCOMPLEX SUBUNIT 9, MITOCHONDRIAL.	**1.32**	0.10	3		**1.01**	0.00	2		**0.36**	0.00	2	
IPI00004358	PYGB	GLYCOGEN PHOSPHORYLASE, BRAIN FORM.	**1.59**	0.01	3		**1.33**	0.04	5		**2.15**	0.71	14	0.638
IPI00004450;IPI00061178;IPI00167369;IPI00304692;IPI00552938;IPI00643486	RBMXL2;RBMXL1;RBMXL3;RBMX;RBMXL1;RBMX	RNA-BINDING MOTIF PROTEIN, X-LINKED-LIKE-2.;"RNA BINDING MOTIF PROTEIN, X-LINKED-LIKE 1.";"RNA-BINDING MOTIF PROTEIN, X-LINKED-LIKE-3.";HETEROGENEOUS NUCLEAR RIBONUCLEOPROTEIN G.;"RNA BINDING MOTIF PROTEIN, X-LINKED ISOFORM 2."			1				2				1	
IPI00004457	AOC3	MEMBRANE PRIMARY AMINE OXIDASE.							3					
IPI00004902;IPI00556451	ETFB	ISOFORM 1 OF ELECTRON TRANSFER FLAVOPROTEIN SUBUNIT BETA.;ISOFORM 2 OF ELECTRON TRANSFER FLAVOPROTEIN SUBUNIT BETA.	**1.11**	0.13	2	0.507	**0.64**	0.12	2	0.477	**0.70**	0.04	2	
IPI00005040;IPI00513827;IPI00895801	ACADM	MEDIUM-CHAIN SPECIFIC ACYL-COA DEHYDROGENASE, MITOCHONDRIAL.;PUTATIVE UNCHARACTERIZED PROTEIN DKFZP686M24262.;MEDIUM-CHAIN ACYL-COA DEHYDROGENASE ISOFORM B PRECURSOR.	**0.78**	0.07	4	0.312	**0.73**	0.05	4	0.310	**0.35**	0.00	4	
IPI00005171;IPI00464948;IPI00645572;IPI00908657;IPI00909061;IPI00922761;IPI00936417	HLA-DRA	HLA CLASS II HISTOCOMPATIBILITY ANTIGEN, DR ALPHA CHAIN.;"MAJOR HISTOCOMPATIBILITY COMPLEX, CLASS II, DR ALPHA PRECURSOR.";"MAJOR HISTOCOMPATIBILITY COMPLEX, CLASS II, DR ALPHA.";"CDNA FLJ52115, HIGHLY SIMILAR TO HLA CLASS II HISTOCOMPATIBILITY ANTIGEN, DRALPHA CHAIN.";"CDNA FLJ51117, HIGHLY SIMILAR TO HLA CLASS II HISTOCOMPATIBILITY ANTIGEN, DRALPHA CHAIN.";"CDNA FLJ57318, HIGHLY SIMILAR TO HLA CLASS II HISTOCOMPATIBILITY ANTIGEN, DRALPHA CHAIN.";"CDNA FLJ51113, HIGHLY SIMILAR TO HLA CLASS II HISTOCOMPATIBILITY ANTIGEN, DRALPHA CHAIN."			1								1	
IPI00005186;IPI00103082;IPI00472169;IPI00472604;IPI00640057;IPI00643602;IPI00643649;IPI00645418;IPI00645451;IPI00746667;IPI00796775;IPI00815678;IPI00816410;IPI00852890;IPI00853042;IPI00871639;IPI00877774;IPI00892984;IPI00893069;IPI00893098;IPI00893146;IPI00893284;IPI00893423;IPI00893809;IPI00894111;IPI00894198;IPI00894266;IPI00922386;IPI00926215;IPI00929265;IPI00930425;IPI00930511;IPI00938353;IPI00939139;IPI00939177;IPI00940612;IPI00941129;IPI00941555;IPI00941950;IPI00943082;IPI00943138;IPI00944601;IPI00947197;IPI00954011;IPI00954107;IPI00954108;IPI00954207;IPI00954582	LOC100133583;HLA-DPB1;HLA-DRB5;LOC100290966;LOC100294468;LOC649783;LOC100133811;LOC100294036;LOC100133661;LOC100294276;HLA-DRB1;HLA-DRB4;HLA-DRB3;HLA-DQB3;LOC100294115;LOC100294278;LOC100294191;LOC100293977;HLA-DQB1	SIMILAR TO MAJOR HISTOCOMPATIBILITY COMPLEX, CLASS II, DR BETA 5 ISOFORM 2.;"HLA CLASS II HISTOCOMPATIBILITY ANTIGEN, DP(W4) BETA CHAIN.";"HLA CLASS II HISTOCOMPATIBILITY ANTIGEN, DRB1-15 BETA CHAIN.";"HLA CLASS II HISTOCOMPATIBILITY ANTIGEN, DP(W2) BETA CHAIN.";"MAJOR HISTOCOMPATIBILITY COMPLEX, CLASS II, DP BETA 1.";MHC CLASS II ANTIGEN.;"HLA CLASS II HISTOCOMPATIBILITY ANTIGEN, DRB1-11 BETA CHAIN.";28 KDA PROTEIN.;MHC CLASS II (FRAGMENT).;CELL SURFACE GLYCOPROTEIN (FRAGMENT).;"CDNA FLJ56572, HIGHLY SIMILAR TO HLA CLASS II HISTOCOMPATIBILITY ANTIGEN, DQ(1)BETA CHAIN.";"CDNA FLJ59004, HIGHLY SIMILAR TO HLA CLASS II HISTOCOMPATIBILITY ANTIGEN, DQ(1)BETA CHAIN.";30 KDA PROTEIN.;"HLA CLASS II HISTOCOMPATIBILITY ANTIGEN, SB BETA CHAIN.";MAJOR HISTOCOMPATIBILITY COMPLEX CLASS II DP BETA 1.;MHC CLASS II ANTIGEN (FRAGMENT).;"HLA CLASS II HISTOCOMPATIBILITY ANTIGEN, DR-1 BETA CHAIN.";HLA-DR3 (FRAGMENT).;"SIMILAR TO MAJOR HISTOCOMPATIBILITY COMPLEX, CLASS II, DR BETA 5.";"MAJOR HISTOCOMPATIBILITY COMPLEX, CLASS II, DQ BETA 1.";"MAJOR HISTOCOMPATIBILITY COMPLEX, CLASS II, DP BETA 1 (FRAGMENT).";27 KDA PROTEIN.;29 KDA PROTEIN.;"SIMILAR TO MAJOR HISTOCOMPATIBILITY COMPLEX, CLASS II, DR BETA 3 ISOFORM 1.";"HLA CLASS II HISTOCOMPATIBILITY ANTIGEN, DQ(1) BETA CHAIN."	**0.93**	0.00	1		**1.53**	0.00	1		**2.09**	0.00	1	
IPI00005198	ILF2	INTERLEUKIN ENHANCER-BINDING FACTOR 2.	**1.35**	0.00	3		**1.75**	0.00	1		**1.09**	0.00	2	
IPI00005202	PGRMC2	MEMBRANE-ASSOCIATED PROGESTERONE RECEPTOR COMPONENT 2.	**1.00**	0.00	1				1				1	
IPI00005563;IPI00910801	TINAGL1	ISOFORM 1 OF TUBULOINTERSTITIAL NEPHRITIS ANTIGEN-LIKE.;"CDNA FLJ55020, HIGHLY SIMILAR TO TUBULOINTERSTITIAL NEPHRITIS ANTIGEN- LIKE."	**2.22**	0.22	2	0.252	**2.28**	0.27	2	0.450	**0.97**	0.00	1	
IPI00005578	EHD4	EH DOMAIN-CONTAINING PROTEIN 4.	**1.21**	0.00	1		**1.13**	0.00	1		**0.79**	0.00	1	0.549
IPI00005614;IPI00328230;IPI00333015	SPTBN1	ISOFORM LONG OF SPECTRIN BETA CHAIN, BRAIN 1.;"ISOFORM SHORT OF SPECTRIN BETA CHAIN, BRAIN 1.";"ISOFORM 2 OF SPECTRIN BETA CHAIN, BRAIN 1."	**1.65**	0.16	8		**1.85**	0.13	10		**1.33**	0.24	9	0.421
IPI00005668;IPI00029733;IPI00289524;IPI00291483;IPI00455167;IPI00514814;IPI00910990	AKR1C2;AKR1C1;AKR1C4;AKR1C3	ALDO-KETO REDUCTASE FAMILY 1 MEMBER C2.;ALDO-KETO REDUCTASE FAMILY 1 MEMBER C1.;ALDO-KETO REDUCTASE FAMILY 1 MEMBER C4.;ALDO-KETO REDUCTASE FAMILY 1 MEMBER C3.;PUTATIVE UNCHARACTERIZED PROTEIN AKR1C2.;"ALDO-KETO REDUCTASE FAMILY 1, MEMBER C1 (DIHYDRODIOL DEHYDROGENASE 1.";"CDNA FLJ52680, HIGHLY SIMILAR TO ALDO-KETO REDUCTASE FAMILY 1 MEMBER C2."			1				1				1	
IPI00005705;IPI00027423;IPI00218187;IPI00218236;IPI00550451;IPI00892668;IPI00894274;IPI00894333;IPI00902512;IPI00930380	PPP1CC;PPP1CA;PPP1CB	ISOFORM GAMMA-1 OF SERINE/THREONINE-PROTEIN PHOSPHATASE PP1-GAMMA CATALYTIC SUBUNIT.;"PROTEIN PHOSPHATASE 1, CATALYTIC SUBUNIT, ALPHA ISOFORM 3.";ISOFORM GAMMA-2 OF SERINE/THREONINE-PROTEIN PHOSPHATASE PP1-GAMMA CATALYTIC SUBUNIT.;SERINE/THREONINE-PROTEIN PHOSPHATASE PP1-BETA CATALYTIC SUBUNIT.;SERINE/THREONINE-PROTEIN PHOSPHATASE PP1-ALPHA CATALYTIC SUBUNIT.;16 KDA PROTEIN.;SERINE/THREONINE PROTEIN PHOSPHATASE.;14 KDA PROTEIN.;20 KDA PROTEIN	**1.59**	0.00	2		**1.33**	0.00	2		**0.97**	0.00	3	
IPI00005707	MRC2	C-TYPE MANNOSE RECEPTOR 2.			1				1				1	
IPI00005737;IPI00399142;IPI00641719	SURF4	ISOFORM 1 OF SURFEIT LOCUS PROTEIN 4.;SURFEIT 4.	**0.76**	0.00	2		**0.64**	0.05	2		**0.25**	0.00	2	
IPI00005923	PNLIPRP1	ISOFORM 1 OF PANCREATIC LIPASE-RELATED PROTEIN 1.	**1.32**	0.27	9	0.441	**0.69**	0.09	8				8	
IPI00005924	PNLIPRP2	PANCREATIC LIPASE-RELATED PROTEIN 2.	**1.62**	0.22	7	0.432	**0.49**	0.11	6				6	
IPI00005969;IPI00873484	CAPZA1	F-ACTIN-CAPPING PROTEIN SUBUNIT ALPHA-1.;PUTATIVE UNCHARACTERIZED PROTEIN CAPZA1 (FRAGMENT).	**1.17**	0.08	2	0.506	**1.29**	0.15	2	0.578	**1.35**	0.09	2	0.549
IPI00005978;IPI00385786;IPI00477842;IPI00746575;IPI00796848;IPI00856075;IPI00894320;IPI00902967	SFRS2;SFRS2B	SPLICING FACTOR, ARGININE/SERINE-RICH 2.;"CDNA FLJ35170 FIS, CLONE PLACE6012942, HIGHLY SIMILAR TO SPLICING FACTOR, ARGININE/SERINE-RICH 2.";"ISOFORM 1 OF SPLICING FACTOR, ARGININE/SERINE-RICH 2B.";"CDNA FLJ39750 FIS, CLONE SMINT2017736, MODERATELY SIMILAR TO SPLICING FACTOR, ARGININE/SERINE-RICH 2.";"CDNA FLJ52570, HIGHLY SIMILAR TO SPLICING FACTOR, ARGININE/SERINE-RICH 2.";"ISOFORM 2 OF SPLICING FACTOR, ARGININE/SERINE-RICH 2B.";PUTATIVE UNCHARACTERIZED PROTEIN ENSP00000381889.;"CDNA FLJ44468 FIS, CLONE UTERU2026025, MODERATELY SIMILAR TO SPLICING FACTOR, ARGININE/SERINE-RICH 2."	**1.25**	0.00	1		**1.23**	0.00	1		**1.16**	0.00	1	
IPI00006034;IPI00921911	CRIP2	CYSTEINE-RICH PROTEIN 2.;"CDNA FLJ56946, HIGHLY SIMILAR TO CYSTEINE-RICH PROTEIN 2."			1				1				1	
IPI00006092	PMM2	PHOSPHOMANNOMUTASE 2.	**1.07**	0.00	1				1				1	
IPI00006114;IPI00790473	SERPINF1	PIGMENT EPITHELIUM-DERIVED FACTOR.;12 KDA PROTEIN.							1				2	
IPI00006196;IPI00292771;IPI00872028	NUMA1	ISOFORM 2 OF NUCLEAR MITOTIC APPARATUS PROTEIN 1.;ISOFORM 1 OF NUCLEAR MITOTIC APPARATUS PROTEIN 1.;NUMA1 VARIANT PROTEIN (FRAGMENT).	**1.65**	0.00	3		**1.04**	0.01	2		**0.97**	0.00	3	
IPI00006205	SLC33A1	ACETYL-COENZYME A TRANSPORTER 1.	**0.57**	0.00	1				1				1	
IPI00006211;IPI00170692;IPI00374657;IPI00642826;IPI00748221;IPI00929577	VAPB;VAPA;ABHD2	ISOFORM 1 OF VESICLE-ASSOCIATED MEMBRANE PROTEIN-ASSOCIATED PROTEIN B/C.;VESICLE-ASSOCIATED MEMBRANE PROTEIN-ASSOCIATED PROTEIN A.;VESICLE-ASSOCIATED MEMBRANE PROTEIN-ASSOCIATED PROTEIN A ISOFORM 1.;14 KDA PROTEIN.;ISOFORM 2 OF VESICLE-ASSOCIATED MEMBRANE PROTEIN-ASSOCIATED PROTEIN B/C.;SIMILAR TO VESICLE-ASSOCIATED MEMBRANE PROTEIN-ASSOCIATED PROTEIN A.	**0.73**	0.00	1		**0.56**	0.00	1		**0.55**	0.00	1	
IPI00006451;IPI00936987	NSF	VESICLE-FUSING ATPASE.;"CDNA FLJ58682, HIGHLY SIMILAR TO VESICLE-FUSING ATPASE."	**0.56**	0.00	1				1				1	
IPI00006482;IPI00414005;IPI00646182	ATP1A1	ISOFORM LONG OF SODIUM/POTASSIUM-TRANSPORTING ATPASE SUBUNIT ALPHA-1.;ISOFORM SHORT OF SODIUM/POTASSIUM-TRANSPORTING ATPASE SUBUNIT ALPHA-1.;NA+/K+ -ATPASE ALPHA 1 SUBUNIT ISOFORM C.	**1.17**	0.08	4	0.347	**1.16**	0.00	4	0.568	**0.55**	0.04	4	
IPI00006579;IPI00645361	COX4I1	CYTOCHROME C OXIDASE SUBUNIT 4 ISOFORM 1, MITOCHONDRIAL.;COX4I1 PROTEIN.	**0.62**	0.00	2		**0.23**	0.00	2		**0.55**	0.00	2	

IPI00006601	CHGB	SECRETOGRANIN-1.	**2.88**	0.00	2		**2.60**	0.83	2				2	
IPI00006663	ALDH2	ALDEHYDE DEHYDROGENASE, MITOCHONDRIAL.	**1.15**	0.12	10	0.347	**1.09**	0.10	11	0.545	**0.65**	0.12	9	0.171
IPI00006690	EPX	EOSINOPHIL PEROXIDASE.			1				3				6	
IPI00006865	SEC22B	VESICLE-TRAFFICKING PROTEIN SEC22B.	**1.01**	0.05	4	0.412	**0.89**	0.19	5		**0.19**	0.00	4	
IPI00006980;IPI00792100	C14orf166	UPF0568 PROTEIN C14ORF166.;CLE.	**0.84**	0.00	2		**0.57**	0.00	2				2	
IPI00007047	S100A8	PROTEIN S100-A8.	**0.64**	0.00	1		**2.55**	0.00	2		**2.33**	0.00	2	
IPI00007061;IPI00791523	GOLT1B	VESICLE TRANSPORT PROTEIN GOT1B.;7 KDA PROTEIN.	**0.74**	0.00	2		**0.51**	0.00	2		**0.40**	0.00	2	
IPI00007068;IPI00028091;IPI00892652;IPI00908396;IPI00936769	ACTR3B;ACTR3;LOC100290215	ISOFORM 1 OF ACTIN-RELATED PROTEIN 3B.;ACTIN-RELATED PROTEIN 3.;ISOFORM 2 OF ACTIN-RELATED PROTEIN 3B.;"CDNA FLJ52216, HIGHLY SIMILAR TO ACTIN-LIKE PROTEIN 3.";HYPOTHETICAL PROTEIN XP_002346994.	**1.12**	0.00	1		**1.47**	0.00	1	0.556	**1.25**	0.00	1	0.420
IPI00007074	YARS	TYROSYL-TRNA SYNTHETASE, CYTOPLASMIC.	**0.89**	0.02	3	0.282	**0.62**	0.00	3		**0.37**	0.00	3	
IPI00007084;IPI00930710	SLC25A13	CALCIUM-BINDING MITOCHONDRIAL CARRIER PROTEIN ARALAR2.;"SOLUTE CARRIER FAMILY 25, MEMBER 13 ISOFORM 1."			1				1				1	
IPI00007102;IPI00032575;IPI00745272;IPI00909336;IPI00922891	GLOD4	UNCHARACTERIZED PROTEIN C17ORF25.;ISOFORM 1 OF GLYOXALASE DOMAIN-CONTAINING PROTEIN 4.;ISOFORM 2 OF GLYOXALASE DOMAIN-CONTAINING PROTEIN 4.;CDNA FLJ59092.;CDNA FLJ55095.	**0.83**	0.00	2		**1.73**	0.00	1		**0.62**	0.00	1	
IPI00007144;IPI00027270;IPI00433834;IPI00789514;IPI00939745	RPL26L1;RPL26	60S RIBOSOMAL PROTEIN L26-LIKE 1.;60S RIBOSOMAL PROTEIN L26.;PUTATIVE UNCHARACTERIZED PROTEIN RPL26.;13 KDA PROTEIN.;17 KDA PROTEIN.	**0.77**	0.04	3		**1.31**	0.00	3				3	
IPI00007188	SLC25A5	ADP/ATP TRANSLOCASE 2.	**0.75**	0.07	7	0.072	**0.63**	0.12	7		**0.53**	0.03	7	0.223
IPI00007242	LGALS2	GALECTIN-2.	**1.13**	0.00	2				1				1	
IPI00007244;IPI00236554;IPI00236556	MPO	ISOFORM H17 OF MYELOPEROXIDASE.;ISOFORM H14 OF MYELOPEROXIDASE.;ISOFORM H7 OF MYELOPEROXIDASE.	**1.97**	0.25	3		**3.36**	0.39	5	0.311	**2.00**	0.27	3	0.570
IPI00007247;IPI00387103;IPI00792361;IPI00921904;IPI00945117;IPI00945862;IPI00946409;IPI00946553;IPI00946876;IPI00947502	PCCB	PROPIONYL-COA CARBOXYLASE BETA CHAIN, MITOCHONDRIAL.;"CDNA FLJ50917, HIGHLY SIMILAR TO PROPIONYL-COA CARBOXYLASE BETA CHAIN, MITOCHONDRIAL.";PUTATIVE UNCHARACTERIZED PROTEIN DKFZP451E113.;"PROPIONYL COENZYME A CARBOXYLASE, BETA POLYPEPTIDE, ISOFORM CRA_C.";"PROPIONYL COENZYME A CARBOXYLASE, BETA POLYPEPTIDE VARIANT.";"CDNA FLJ53115, HIGHLY SIMILAR TO PROPIONYL-COA CARBOXYLASE BETA CHAIN, MITOCHONDRIAL.";56 KDA PROTEIN.;61 KDA PROTEIN.;62 KDA PROTEIN.	**1.19**	0.12	2				2				2	
IPI00007423;IPI00025849;IPI00449263;IPI00647001;IPI00759824;IPI00940656	ANP32B;LOC723972;ANP32A	ISOFORM 1 OF ACIDIC LEUCINE-RICH NUCLEAR PHOSPHOPROTEIN 32 FAMILY MEMBER B.;ACIDIC LEUCINE-RICH NUCLEAR PHOSPHOPROTEIN 32 FAMILY MEMBER A.;ANP32A PROTEIN (FRAGMENT).;"ACIDIC (LEUCINE-RICH) NUCLEAR PHOSPHOPROTEIN 32 FAMILY, MEMBER B.";ISOFORM 2 OF ACIDIC LEUCINE-RICH NUCLEAR PHOSPHOPROTEIN 32 FAMILY MEMBER B.;PUTATIVE UNCHARACTERIZED PROTEIN ANP32A.	**1.41**	0.00	1		**1.11**	0.00	1		**1.87**	0.00	2	
IPI00007426;IPI00908696;IPI00909717;IPI00945943;IPI00946450;IPI00946721	ARL6IP5	PRA1 FAMILY PROTEIN 3.;"CDNA FLJ52128, HIGHLY SIMILAR TO PRA1 FAMILY PROTEIN 3.";"CDNA FLJ51702, MODERATELY SIMILAR TO PRA1 FAMILY PROTEIN 3.";10 KDA PROTEIN.;7 KDA PROTEIN.							1				1	
IPI00007427;IPI00893607;IPI00942032;IPI00952571	AGR2	AGR2.;PUTATIVE UNCHARACTERIZED PROTEIN AGR2.;ANTERIOR GRADIENT PROTEIN 2 HOMOLOG.	**3.94**	1.17	7		**1.24**	0.00	2		**4.04**	0.31	5	
IPI00007611	ATP5O	ATP SYNTHASE SUBUNIT O, MITOCHONDRIAL.	**1.10**	0.07	4	0.419	**0.80**	0.09	3	0.276	**0.40**	0.00	3	0.125
IPI00007750;IPI00794663	TUBA4A	TUBULIN ALPHA-4A CHAIN.;"TUBULIN, ALPHA 1 (TESTIS SPECIFIC), ISOFORM CRA_A."	**1.05**	0.08	13	0.375	**1.10**	0.14	13	0.536	**0.88**	0.09	13	0.557
IPI00007752;IPI00908605;IPI00930130	TUBB2C	TUBULIN BETA-2C CHAIN.;"CDNA FLJ59940, HIGHLY SIMILAR TO TUBULIN BETA-2C CHAIN.";"CDNA FLJ11352 FIS, CLONE HEMBA1000020, HIGHLY SIMILAR TO TUBULIN BETA- 2C CHAIN."	**0.87**	0.05	4		**0.64**	0.05	4		**0.42**	0.03	5	
IPI00007764;IPI00384857;IPI00917313	HN1	ISOFORM 1 OF HEMATOLOGICAL AND NEUROLOGICAL EXPRESSED 1 PROTEIN.;ISOFORM 2 OF HEMATOLOGICAL AND NEUROLOGICAL EXPRESSED 1 PROTEIN.;PUTATIVE UNCHARACTERIZED PROTEIN HN1.											1	
IPI00007765	HSPA9	STRESS-70 PROTEIN, MITOCHONDRIAL.	**1.00**	0.04	9	0.309	**0.73**	0.12	9	0.244	**0.76**	0.20	10	0.198
IPI00007926	C6orf108	DEOXYRIBONUCLEOSIDE 5'-MONOPHOSPHATE N-GLYCOSIDASE.	**0.82**	0.00	2		**0.38**	0.00	2		**0.23**	0.00	2	
IPI00007935;IPI00553102;IPI00555593	PDLIM5	PDZ AND LIM DOMAIN PROTEIN 5.;PDZ AND LIM DOMAIN 5 ISOFORM B.;ENIGMA HOMOLOG (FRAGMENT).	**1.24**	0.00	1		**2.10**	0.00	1		**1.75**	0.00	1	
IPI00007960;IPI00218585;IPI00410241;IPI00641231;IPI00910262	POSTN	ISOFORM 1 OF PERIOSTIN.;ISOFORM 2 OF PERIOSTIN.;"PERIOSTIN, OSTEOBLAST SPECIFIC FACTOR.";ISOFORM 4 OF PERIOSTIN.	**1.21**	0.41	8		**2.38**	0.72	18		**3.26**	1.61	25	
IPI00008037;IPI00296333;IPI00401990;IPI00411387;IPI00657816;IPI00657883;IPI00657924;IPI00658107;IPI00854706;IPI00874262;IPI00879209;IPI00939479;IPI00940097	ACSL5;ACSL6	ISOFORM 1 OF LONG-CHAIN-FATTY-ACID--COA LIGASE 5.;ISOFORM 3 OF LONG-CHAIN-FATTY-ACID--COA LIGASE 6.;ISOFORM 2 OF LONG-CHAIN-FATTY-ACID--COA LIGASE 5.;"CDNA FLJ52792, HIGHLY SIMILAR TO LONG-CHAIN-FATTY-ACID--COA LIGASE 5.";ISOFORM 2 OF LONG-CHAIN-FATTY-ACID--COA LIGASE 6.;ISOFORM 1 OF LONG-CHAIN-FATTY-ACID--COA LIGASE 6.;41 KDA PROTEIN.;ISOFORM 8 OF LONG-CHAIN-FATTY-ACID--COA LIGASE 6.;ISOFORM 3 OF LONG-CHAIN-FATTY-ACID--COA LIGASE 5.;79 KDA PROTEIN.;ISOFORM 6 OF LONG-CHAIN-FATTY-ACID--COA LIGASE 6.;ISOFORM 4 OF LONG-CHAIN-FATTY-ACID--COA LIGASE 6.;ISOFORM 5 OF LONG-CHAIN-FATTY-ACID--COA LIGASE 6.			1								1	
IPI00008219	RAD23A	UV EXCISION REPAIR PROTEIN RAD23 HOMOLOG A.	**1.25**	0.00	1		**1.37**	0.00	1				1	
IPI00008223;IPI00642549	RAD23B	UV EXCISION REPAIR PROTEIN RAD23 HOMOLOG B.;"CDNA FLJ56531, HIGHLY SIMILAR TO UV EXCISION REPAIR PROTEIN RAD23 HOMOLOG B."	**1.26**	0.06	2		**0.98**	0.09	2		**0.83**	0.02	2	
IPI00008240;IPI00910687	MARS	METHIONYL-TRNA SYNTHETASE, CYTOPLASMIC.;"CDNA FLJ53309, HIGHLY SIMILAR TO METHIONYL-TRNA SYNTHETASE."			1				1				1	
IPI00008274	CAP1	ISOFORM 1 OF ADENYLYL CYCLASE-ASSOCIATED PROTEIN 1.	**0.94**	0.13	4	0.347	**1.66**	0.36	4	0.319	**1.64**	0.35	4	0.206
IPI00008433	RPS5	40S RIBOSOMAL PROTEIN S5.	**0.69**	0.00	2		**0.34**	0.00	2		**0.23**	0.00	2	
IPI00008438;IPI00749512	RPS10	40S RIBOSOMAL PROTEIN S10.;RIBOSOMAL PROTEIN S10 VARIANT (FRAGMENT).	**0.73**	0.00	2	0.110	**0.38**	0.02	2	0.067	**0.27**	0.00	2	0.103
IPI00008454	DNAJB11	DNAJ HOMOLOG SUBFAMILY B MEMBER 11.			1				1				1	
IPI00008485	ACO1	CYTOPLASMIC ACONITATE HYDRATASE.	**0.81**	0.06	5	0.152	**0.79**	0.07	4		**0.23**	0.00	4	
IPI00008527	RPLP1	60S ACIDIC RIBOSOMAL PROTEIN P1.	**0.60**	0.05	2		**0.40**	0.00	2		**0.17**	0.00	2	
IPI00008529	RPLP2	60S ACIDIC RIBOSOMAL PROTEIN P2.	**0.81**	0.05	10	0.078	**0.71**	0.07	10	0.171	**0.36**	0.08	10	
IPI00008530;IPI00556485;IPI00791448	RPLP0	60S ACIDIC RIBOSOMAL PROTEIN P0.;RPLP0 PROTEIN.;"CDNA FLJ51469, HIGHLY SIMILAR TO 60S ACIDIC RIBOSOMAL PROTEIN P0."	**0.75**	0.05	5		**0.55**	0.03	6		**0.31**	0.03	6	
IPI00008552	GLRX3	GLUTAREDOXIN-3.											1	
IPI00008575;IPI00082310;IPI00385834;IPI00479209	KHDRBS1	ISOFORM 1 OF KH DOMAIN-CONTAINING, RNA-BINDING, SIGNAL TRANSDUCTION- ASSOCIATED PROTEIN 1.;"ISOFORM 3 OF KH DOMAIN-CONTAINING, RNA-BINDING, SIGNAL TRANSDUCTION-ASSOCIATED PROTEIN 1.";"ISOFORM 2 OF KH DOMAIN-CONTAINING, RNA-BINDING, SIGNAL TRANSDUCTION- ASSOCIATED PROTEIN 1.";"CDNA FLJ54590, HIGHLY SIMILAR TO KH DOMAIN-CONTAINING, RNA-BINDING, SIGNALTRANSDUCTION-ASSOCIATED PROTEIN 1."	**0.78**	0.00	1		**1.45**	0.00	1		**1.17**	0.00	1	
IPI00008708;IPI00642046	RSL1D1	RIBOSOMAL L1 DOMAIN-CONTAINING PROTEIN 1.;RSL1D1 PROTEIN.							1				1	
IPI00008730;IPI00044168	SCGN	SECRETAGOGIN.;SETAGIN.	**3.30**	0.00	1		**2.46**	0.00	3		**0.59**	0.00	2	
IPI00008868;IPI00020356;IPI00789977;IPI00887765	MAP1B;MAP1A	MICROTUBULE-ASSOCIATED PROTEIN 1B.;331 KDA PROTEIN.;MICROTUBULE-ASSOCIATED PROTEIN 1A.			1				1				1	
IPI00008934;IPI00945760	HMGCS2	HYDROXYMETHYLGLUTARYL-COA SYNTHASE, MITOCHONDRIAL.;HYDROXYMETHYLGLUTARYL-COA SYNTHASE 2 ISOFORM 2 PRECURSOR.			2				2				2	
IPI00008982;IPI00218547	ALDH18A1	ISOFORM LONG OF DELTA-1-PYRROLINE-5-CARBOXYLATE SYNTHETASE.;ISOFORM SHORT OF DELTA-1-PYRROLINE-5-CARBOXYLATE SYNTHETASE.	**2.05**	0.00	1				1		**0.76**	0.00	1	
IPI00009027	REG1A	LITHOSTATHINE-1-ALPHA.	**0.72**	0.02	4		**0.99**	0.10	4		**0.33**	0.00	4	
IPI00009028	CLEC3B	TETRANECTIN.							1				1	
IPI00009030;IPI00216172;IPI00739827;IPI00922445	LAMP2	ISOFORM LAMP-2A OF LYSOSOME-ASSOCIATED MEMBRANE GLYCOPROTEIN 2.;LYSOSOMAL-ASSOCIATED MEMBRANE PROTEIN 2 ISOFORM C PRECURSOR.;ISOFORM LAMP-2B OF LYSOSOME-ASSOCIATED MEMBRANE GLYCOPROTEIN 2.;"CDNA FLJ52540, HIGHLY SIMILAR TO LYSOSOME-ASSOCIATED MEMBRANE GLYCOPROTEIN 2."	**1.13**	0.00	1		**1.00**	0.00	1		**1.04**	0.00	1	
IPI00009032;IPI00916802;IPI00916922;IPI00917205;IPI00917386	SSB	LUPUS LA PROTEIN.;SS-B/LA PROTEIN.;21 KDA PROTEIN.;SJOGREN SYNDROME ANTIGEN B.			1				1				2	
IPI00009123;IPI00922708	NUCB2	ISOFORM 1 OF NUCLEOBINDIN-2.;ISOFORM 2 OF NUCLEOBINDIN-2.	**0.52**	0.03	3		**0.31**	0.00	3		**0.10**	0.00	3	
IPI00009235;IPI00945960;IPI00946150	SSR3	TRANSLOCON-ASSOCIATED PROTEIN SUBUNIT GAMMA.;20 KDA PROTEIN.;"CDNA FLJ52061, HIGHLY SIMILAR TO TRANSLOCON-ASSOCIATED PROTEIN SUBUNIT GAMMA."	**0.69**	0.00	2		**0.97**	0.00	2		**0.94**	0.00	2	
IPI00009236;IPI00759683;IPI00852906;IPI00853146;IPI00876941	CAV1	ISOFORM ALPHA OF CAVEOLIN-1.;ISOFORM BETA OF CAVEOLIN-1.;16 KDA PROTEIN.;CAVEOLIN.;CAVEOLIN (FRAGMENT).			1				1					
IPI00009315	ACBD3	GOLGI RESIDENT PROTEIN GCP60.			2				2				2	
IPI00009329	UTRN	UTROPHIN.	**3.20**	0.00	1		**2.44**	0.00	2				1	
IPI00009342	IQGAP1	RAS GTPASE-ACTIVATING-LIKE PROTEIN IQGAP1.	**1.11**	0.12	7	0.348	**1.17**	0.19	7	0.390	**1.16**	0.16	8	0.649

IPI00009407	DAD1	DOLICHYL-DIPHOSPHOOLIGOSACCHARIDE--PROTEIN GLYCOSYLTRANSFERASE SUBUNIT DAD1.	**0.98**	0.00	1		**0.73**	0.00	1		**0.39**	0.00	1	
IPI00009532	ABAT	CDNA FLJ56034, HIGHLY SIMILAR TO 4-AMINOBUTYRATE AMINOTRANSFERASE, MITOCHONDRIAL.	**0.74**	0.13	8	0.070	**0.41**	0.11	8	0.064	**0.09**	0.00	8	
IPI00009634;IPI00790715	SQRDL	SULFIDE:QUINONE OXIDOREDUCTASE, MITOCHONDRIAL.;25 KDA PROTEIN.			1		**1.71**	0.00	1		**2.51**	0.00	1	
IPI00009750	LGALS4	GALECTIN-4.	**2.88**	0.00	2		**2.64**	0.00	2		**2.11**	0.00	2	
IPI00009771;IPI00879819	LMNB2	LAMIN-B2.	**1.67**	0.10	5	0.177	**1.82**	0.44	5	0.208	**1.27**	0.26	7	0.399
IPI00009802;IPI00215628;IPI00215631	VCAN	ISOFORM V0 OF VERSICAN CORE PROTEIN.;ISOFORM V1 OF VERSICAN CORE PROTEIN.;ISOFORM VINT OF VERSICAN CORE PROTEIN.			2		**8.20**	0.00	3		**20.75**	0.00	9	
IPI00009823	CPA1	CARBOXYPEPTIDASE A1.	**0.58**	0.09	33	0.000	**0.19**	0.04	33	0.000	**0.04**	0.01	32	0.010
IPI00009826	CPB1	CARBOXYPEPTIDASE B.	**0.61**	0.07	31	0.000	**0.35**	0.08	31	0.000	**0.04**	0.01	29	0.060
IPI00009829	CPA3	MAST CELL CARBOXYPEPTIDASE A.	**0.89**	0.00	1		**2.58**	0.00	2				1	
IPI00009841;IPI00065554;IPI00293254;IPI00872855;IPI00878484;IPI00879242;IPI00879259;IPI00953325	EWSR1	EWING SARCOMA BREAKPOINT REGION 1 ISOFORM 1.;EWING SARCOMA BREAKPOINT REGION 1 ISOFORM 4.;ISOFORM EWS-B OF RNA-BINDING PROTEIN EWS.;ISOFORM EWS OF RNA-BINDING PROTEIN EWS.;PROTEIN.;EWING SARCOMA BREAKPOINT REGION 1.;EWING SARCOMA BREAKPOINT REGION 1 ISOFORM 3.	**2.12**	0.00	1				1				1	
IPI00009865	KRT10	KERATIN, TYPE I CYTOSKELETAL 10.	**1.36**	0.08	4	0.347	**1.86**	0.31	8	0.610	**1.73**	0.50	7	0.591
IPI00009867;IPI00796776	KRT5	KERATIN, TYPE II CYTOSKELETAL 5.;"CDNA FLJ54081, HIGHLY SIMILAR TO KERATIN, TYPE II CYTOSKELETAL 5."				0.516	**1.41**	0.14	4	0.463				0.638
IPI00009885;IPI00789642;IPI00791084;IPI00793091;IPI00794245	FKBP11	FK506-BINDING PROTEIN 11.;FK506 BINDING PROTEIN 11 ISOFORM 3 PRECURSOR.;16 KDA PROTEIN.;10 KDA PROTEIN.;15 KDA PROTEIN.	**0.74**	0.09	5	0.214	**0.56**	0.01	5	0.175	**0.10**	0.00	5	
IPI00009896	EPHX1	EPOXIDE HYDROLASE 1.	**0.82**	0.10	9	0.112	**0.49**	0.10	9	0.082	**0.11**	0.01	9	0.087
IPI00009904	PDIA4	PROTEIN DISULFIDE-ISOMERASE A4.	**0.88**	0.13	22	0.044	**0.80**	0.10	21	0.202	**0.20**	0.03	21	0.097
IPI00009950	LMAN2	VESICULAR INTEGRAL-MEMBRANE PROTEIN VIP36.	**1.03**	0.00	1		**0.78**	0.00	1				1	
IPI00009960;IPI00470829;IPI00554469;IPI00655644;IPI00910079;IPI00917497;IPI00925553;IPI00926611;IPI00940432	IMMT	ISOFORM 1 OF MITOCHONDRIAL INNER MEMBRANE PROTEIN.;ISOFORM 3 OF MITOCHONDRIAL INNER MEMBRANE PROTEIN.;ISOFORM 2 OF MITOCHONDRIAL INNER MEMBRANE PROTEIN.;PUTATIVE UNCHARACTERIZED PROTEIN IMMT.;"CDNA FLJ59418, HIGHLY SIMILAR TO MITOCHONDRIAL INNER MEMBRANE PROTEIN.";IMMT PROTEIN.;PROTEIN.;79 KDA PROTEIN.;"CDNA, FLJ92546, HIGHLY SIMILAR TO HOMO SAPIENS INNER MEMBRANE PROTEIN, MITOCHONDRIAL (MITOFILIN) (IMMT), MRNA."			2				1					
IPI00010105;IPI00375391	EIF6	EUKARYOTIC TRANSLATION INITIATION FACTOR 6.;EUKARYOTIC TRANSLATION INITIATION FACTOR 6 ISOFORM C.	**1.02**	0.00	1				1		**0.99**	0.00	1	
IPI00010133;IPI00642725	CORO1A	CORONIN-1A.;40 KDA PROTEIN.			2		**2.71**	0.00	2		**1.65**	0.00	1	
IPI00010157;IPI00916229	MAT2A	S-ADENOSYLMETHIONINE SYNTHETASE ISOFORM TYPE-2.;S-ADENOSYLMETHIONINE SYNTHETASE.	**0.61**	0.00	1		**0.56**	0.00	1				1	
IPI00010180;IPI00514878;IPI00607693;IPI00607801;IPI00939157;IPI00940556	CES1	ISOFORM 1 OF LIVER CARBOXYLESTERASE 1.;PUTATIVE UNCHARACTERIZED PROTEIN ENSP00000355193.;CARBOXYLESTERASE 1 ISOFORM C PRECURSOR.;ISOFORM 2 OF LIVER CARBOXYLESTERASE 1.;24 KDA PROTEIN.;28 KDA PROTEIN.	**0.77**	0.00	1		**1.23**	0.00	1		**0.56**	0.00	1	
IPI00010270;IPI00877663;IPI00878066;IPI00879948	RAC2	RAS-RELATED C3 BOTULINUM TOXIN SUBSTRATE 2.;"RAS-RELATED C3 BOTULINUM TOXIN SUBSTRATE 2 (RHO FAMILY, SMALL GTP BINDING PROTEIN RAC2), ISOFORM CRA_C."							1				1	
IPI00010274;IPI00219931;IPI00382751;IPI00419942;IPI00472739;IPI00915376;IPI00930446;IPI00941399;IPI00943162;IPI00946599	TPSAB1;TPSB2;TPSAB1;TPSB2	ISOFORM 1 OF TRYPTASE ALPHA-1.;ISOFORM 2 OF TRYPTASE ALPHA-1.;MAST CELL TRYPTASE BETA III.;TRYPTASE BETA-2.;ISOFORM 2 OF TRYPTASE BETA-1.;TRYPTASE BETA 2 PRECURSOR.;TPSAB1 PROTEIN.;TPSAB1 PROTEIN (FRAGMENT).;26 KDA PROTEIN.;TPSB2 PROTEIN.	**3.29**	0.31	4	0.177	**3.72**	0.17	4	0.090	**1.91**	0.38	4	0.655
IPI00010341;IPI00847535	PRG2	BONE MARROW PROTEOGLYCAN.;SIMILAR TO SOLUTE CARRIER FAMILY 43 MEMBER 3.			1				2				4	
IPI00010402;IPI00514669;IPI00746352	SH3BGRL3	PUTATIVE UNCHARACTERIZED PROTEIN.;SH3 DOMAIN BINDING GLUTAMIC ACID-RICH PROTEIN LIKE 3.;SH3 DOMAIN-BINDING GLUTAMIC ACID-RICH-LIKE PROTEIN 3.	**1.61**	0.00	1		**0.86**	0.00	2		**2.24**	0.00	2	
IPI00010414	PDLIM1	PDZ AND LIM DOMAIN PROTEIN 1.			1				1				2	
IPI00010418;IPI00743335;IPI00829992	MYO1C	ISOFORM 2 OF MYOSIN-IC.;ISOFORM 1 OF MYOSIN-IC.;ISOFORM 3 OF MYOSIN-IC.	**1.20**	0.14	7	0.309	**1.55**	0.09	8	0.266	**0.97**	0.14	6	0.712
IPI00010471	LCP1	PLASTIN-2.	**0.98**	0.15	7	0.323	**2.18**	0.41	9	0.536	**1.48**	0.48	13	0.282
IPI00010697;IPI00216221;IPI00216222;IPI00216223;IPI00216224;IPI00216225;IPI00921401	ITGA6	ISOFORM ALPHA-6X1X2B OF INTEGRIN ALPHA-6.;ISOFORM ALPHA-6X1A OF INTEGRIN ALPHA-6.;ISOFORM ALPHA-6X1B OF INTEGRIN ALPHA-6.;ISOFORM ALPHA-6X2A OF INTEGRIN ALPHA-6.;ISOFORM ALPHA-6X2B OF INTEGRIN ALPHA-6.;ISOFORM ALPHA-6X1X2A OF INTEGRIN ALPHA-6.;ISOFORM 9 OF INTEGRIN ALPHA-6.	**1.50**	0.00	1				1		**1.86**	0.00	2	
IPI00010720;IPI00940257	CCT5	T-COMPLEX PROTEIN 1 SUBUNIT EPSILON.;"CDNA FLJ52361, HIGHLY SIMILAR TO T-COMPLEX PROTEIN 1 SUBUNIT EPSILON."	**1.34**	0.08	3	0.127	**1.22**	0.10	4		**0.49**	0.03	3	0.099
IPI00010740	SFPQ	ISOFORM LONG OF SPLICING FACTOR, PROLINE- AND GLUTAMINE-RICH.	**1.52**	0.00	2		**1.58**	0.00	2	0.515	**0.95**	0.00	2	
IPI00010779;IPI00910322	TPM4	ISOFORM 1 OF TROPOMYOSIN ALPHA-4 CHAIN.;"CDNA FLJ52936, WEAKLY SIMILAR TO TROPOMYOSIN ALPHA-4 CHAIN."	**2.22**	0.41	11		**3.47**	0.64	10		**4.06**	0.74	9	
IPI00010790;IPI00643384;IPI00903084	BGN	BIGLYCAN.;PUTATIVE UNCHARACTERIZED PROTEIN BGN.;"CDNA FLJ35704 FIS, CLONE SPLEN2020183, HIGHLY SIMILAR TO BIGLYCAN."									**7.87**	3.39	8	
IPI00010796	P4HB	PROTEIN DISULFIDE-ISOMERASE.	**0.75**	0.13	40	0.001	**0.45**	0.11	40	0.001	**0.12**	0.02	40	0.001
IPI00010800	NES	NESTIN.			1				1					
IPI00010810;IPI00895865	ETFA	ELECTRON TRANSFER FLAVOPROTEIN SUBUNIT ALPHA, MITOCHONDRIAL.;"ELECTRON TRANSFER FLAVOPROTEIN, ALPHA POLYPEPTIDE ISOFORM B."	**1.11**	0.03	3	0.216	**0.62**	0.00	2		**0.78**	0.08	4	
IPI00010845;IPI00908766	NDUFS8	NADH DEHYDROGENASE [UBIQUINONE] IRON-SULFUR PROTEIN 8, MITOCHONDRIAL.;"CDNA FLJ57061, HIGHLY SIMILAR TO NADH-UBIQUINONE OXIDOREDUCTASE 23 KDA SUBUNIT, MITOCHONDRIAL."	**1.04**	0.00	1		**1.10**	0.00	1				1	
IPI00010896	CLIC1	CHLORIDE INTRACELLULAR CHANNEL PROTEIN 1.	**1.31**	0.00	2		**1.79**	0.00	2		**1.59**	0.00	3	
IPI00011062;IPI00397498;IPI00889534	CPS1	ISOFORM 1 OF CARBAMOYL-PHOSPHATE SYNTHASE [AMMONIA], MITOCHONDRIAL.;"ISOFORM 2 OF CARBAMOYL-PHOSPHATE SYNTHASE [AMMONIA], MITOCHONDRIAL.";CARBAMOYL-PHOSPHATE SYNTHETASE 1 ISOFORM A PRECURSOR.			3									
IPI00011107;IPI00953980	IDH2	ISOCITRATE DEHYDROGENASE [NADP], MITOCHONDRIAL.	**0.86**	0.04	7		**0.75**	0.09	7		**0.27**	0.01	6	
IPI00011200	PHGDH	D-3-PHOSPHOGLYCERATE DEHYDROGENASE.	**0.69**	0.07	10	0.026	**0.34**	0.06	10	0.002	**0.16**	0.04	10	0.087
IPI00011229	CTSD	CATHEPSIN D.	**0.78**	0.27	5	0.177	**1.70**	0.23	4	0.206	**0.86**	0.28	4	0.604
IPI00011253	RPS3	40S RIBOSOMAL PROTEIN S3.	**0.87**	0.09	6	0.051	**0.54**	0.07	6	0.082	**0.27**	0.01	6	0.176
IPI00011261	C8G	COMPLEMENT COMPONENT C8 GAMMA CHAIN.							1				1	
IPI00011274;IPI00045498;IPI00845282	HNRPDL	ISOFORM 1 OF HETEROGENEOUS NUCLEAR RIBONUCLEOPROTEIN D-LIKE.;ISOFORM 3 OF HETEROGENEOUS NUCLEAR RIBONUCLEOPROTEIN D-LIKE.;ISOFORM 2 OF HETEROGENEOUS NUCLEAR RIBONUCLEOPROTEIN D-LIKE.									**0.63**	0.00	3	
IPI00011285;IPI00910547	CAPN1	CALPAIN-1 CATALYTIC SUBUNIT.;"CDNA FLJ53224, HIGHLY SIMILAR TO CALPAIN-1 CATALYTIC SUBUNIT."	**1.27**	0.22	2		**1.78**	0.25	2		**1.45**	0.00	3	
IPI00011416	ECH1	DELTA(3,5)-DELTA(2,4)-DIENOYL-COA ISOMERASE, MITOCHONDRIAL.	**1.11**	0.05	3	0.399	**0.86**	0.05	3				3	
IPI00011454;IPI00383581;IPI00441414	GANAB	ISOFORM 2 OF NEUTRAL ALPHA-GLUCOSIDASE AB.;"CDNA FLJ61290, HIGHLY SIMILAR TO NEUTRAL ALPHA-GLUCOSIDASE AB.";ISOFORM 3 OF NEUTRAL ALPHA-GLUCOSIDASE AB.	**1.12**	0.18	9	0.399	**0.85**	0.11	10	0.269	**0.65**	0.10	9	0.296
IPI00011571;IPI00845275;IPI00878546	PDIA2	PROTEIN DISULFIDE-ISOMERASE A2.;"PROTEIN DISULFIDE ISOMERASE FAMILY A, MEMBER 2 VARIANT.";"PROTEIN DISULFIDE ISOMERASE FAMILY A, MEMBER 2."	**0.74**	0.15	26	0.002	**0.45**	0.13	25	0.039	**0.07**	0.00	26	
IPI00011694;IPI00945846;IPI00946754	PRSS1	TRYPSIN-1.;28 KDA PROTEIN.;PROTEASE SERINE 1.	**0.66**	0.12	10	0.074	**0.25**	0.06	10	0.087	**0.03**	0.00	10	0.153
IPI00011695;IPI00815665;IPI00954434	PRSS2;PRSS1	PROTEASE SERINE 2 ISOFORM B.;PRSS1 PROTEIN.	**0.72**	0.08	13	0.088	**0.32**	0.07	13	0.075	**0.06**	0.02	13	0.099
IPI00011876;IPI00903024;IPI00942294	MTAP	CDNA FLJ59758, HIGHLY SIMILAR TO S-METHYL-5-THIOADENOSINE PHOSPHORYLASE.;16 KDA PROTEIN.;S-METHYL-5'-THIOADENOSINE PHOSPHORYLASE.	**1.55**	0.18	2		**1.80**	0.00	2		**2.57**	0.00	2	
IPI00011913	HNRNPA0	HETEROGENEOUS NUCLEAR RIBONUCLEOPROTEIN A0.	**1.07**	0.00	1		**1.25**	0.00	1				1	
IPI00011916;IPI00893242;IPI00893993;IPI00940113	AIMP2;STAG3L1;STAG3L2;AIMP2;STAG3L3;STAG3L1;STAG3L2;STAG3L3	AMINOACYL TRNA SYNTHETASE COMPLEX-INTERACTING MULTIFUNCTIONAL PROTEIN 2.;PUTATIVE UNCHARACTERIZED PROTEIN STAG3L3.;28 KDA PROTEIN.;"CDNA, FLJ93510, HIGHLY SIMILAR TO HOMO SAPIENS JTV1 GENE (JTV1), MRNA."	**1.34**	0.00	1		**0.96**	0.00	1		**0.24**	0.00	1	
IPI00011937;IPI00640466;IPI00938009	PRDX4;LOC100292021	PEROXIREDOXIN-4.;PUTATIVE UNCHARACTERIZED PROTEIN PRDX4.;SIMILAR TO THIOREDOXIN PEROXIDASE.	**0.67**	0.14	3		**0.43**	0.00	3		**0.07**	0.00	3	0.048
IPI00012007;IPI00935818	AHCY	ADENOSYLHOMOCYSTEINASE.;ADENOSYLHOMOCYSTEINASE ISOFORM 2.	**1.07**	0.08	3		**1.10**	0.00	3	0.275	**0.69**	0.00	3	0.268
IPI00012011;IPI00909841	CFL1	COFILIN-1.;"CDNA FLJ51435, MODERATELY SIMILAR TO COFILIN-1."	**1.36**	0.21	4		**1.66**	0.33	4		**1.80**	0.38	4	
IPI00012048;IPI00375531;IPI00791971	NME1	ISOFORM 1 OF NUCLEOSIDE DIPHOSPHATE KINASE A.;ISOFORM 2 OF NUCLEOSIDE DIPHOSPHATE KINASE A.;9 KDA PROTEIN.	**0.97**	0.08	2		**1.01**	0.00	2				2	

IPI00012066;IPI00216689;IPI00470509;IPI00788837;IPI00790627;IPI00791223;IPI00796337;IPI00900327	PCBP2	POLY(RC) BINDING PROTEIN 2 ISOFORM B.;POLY(RC)-BINDING PROTEIN 2.;POLY(RC) BINDING PROTEIN 2 ISOFORM C.;"CDNA FLJ54752, HIGHLY SIMILAR TO POLY(RC)-BINDING PROTEIN 2.";POLY(RC) BINDING PROTEIN 2 ISOFORM E.;POLY(RC) BINDING PROTEIN 2 ISOFORM F.;POLY(RC) BINDING PROTEIN 2 ISOFORM A.;"CDNA FLJ58339, HIGHLY SIMILAR TO POLY(RC)-BINDING PROTEIN 2."	**0.91**	0.00	1		**0.88**	0.00	1		**0.57**	0.00	1	
IPI00012074;IPI00644055;IPI00856037;IPI00856038;IPI00910614;IPI00941649	HNRNPR	HETEROGENEOUS NUCLEAR RIBONUCLEOPROTEIN R.;HETEROGENEOUS NUCLEAR RIBONUCLEOPROTEIN R ISOFORM 1.;HETEROGENEOUS NUCLEAR RIBONUCLEOPROTEIN R ISOFORM 3.;HETEROGENEOUS NUCLEAR RIBONUCLEOPROTEIN-R2.;HETEROGENEOUS NUCLEAR RIBONUCLEOPROTEIN R ISOFORM 4.;71 KDA PROTEIN.	**0.87**	0.21	2		**1.00**	0.06	2	0.557	**0.64**	0.10	2	0.382
IPI00012079;IPI00798302;IPI00908588;IPI00910983;IPI00942600	EIF4B	EUKARYOTIC TRANSLATION INITIATION FACTOR 4B.;"CDNA FLJ59405, HIGHLY SIMILAR TO EUKARYOTIC TRANSLATION INITIATION FACTOR 4B.";"CDNA FLJ59206, HIGHLY SIMILAR TO EUKARYOTIC TRANSLATION INITIATION FACTOR 4B.";"CDNA FLJ59402, HIGHLY SIMILAR TO EUKARYOTIC TRANSLATION INITIATION FACTOR 4B.";70 KDA PROTEIN.	**1.42**	0.00	1				1		**0.30**	0.00	1	
IPI00012119	DCN	ISOFORM A OF DECORIN.	**4.46**	0.23	6		**7.13**	0.17	7		**4.52**	0.33	8	
IPI00012268;IPI00793806;IPI00926258;IPI00926410	PSMD2	26S PROTEASOME NON-ATPASE REGULATORY SUBUNIT 2.;15 KDA PROTEIN.;86 KDA PROTEIN.;"CDNA FLJ55045, HIGHLY SIMILAR TO 26S PROTEASOME NON-ATPASE REGULATORY SUBUNIT 2."	**2.29**	0.00	1				1				2	
IPI00012493;IPI00794659	RPS20	40S RIBOSOMAL PROTEIN S20.;RIBOSOMAL PROTEIN S20 ISOFORM 1.				0.195	**0.52**	0.10	4	0.129	**0.21**	0.00	4	0.102
IPI00012503;IPI00219825;IPI00744835;IPI00873020;IPI00873201	PSAP	ISOFORM SAP-MU-0 OF PROACTIVATOR POLYPEPTIDE.;PROSAPOSIN.;ISOFORM SAP-MU-9 OF PROACTIVATOR POLYPEPTIDE.;58 KDA PROTEIN.;ISOFORM SAP-MU-6 OF PROACTIVATOR POLYPEPTIDE.							1				2	
IPI00012726;IPI00555747;IPI00642904;IPI00642944	PABPC4	ISOFORM 1 OF POLYADENYLATE-BINDING PROTEIN 4.;ISOFORM 2 OF POLYADENYLATE-BINDING PROTEIN 4.;"POLY A BINDING PROTEIN, CYTOPLASMIC 4 ISOFORM 1.";"POLY(A) BINDING PROTEIN, CYTOPLASMIC 4 (INDUCIBLE FORM), ISOFORM CRA_E."	**0.68**	0.02	2	0.149	**0.67**	0.06	2	0.394	**0.33**	0.01	2	
IPI00012750;IPI00903251	RPS25	40S RIBOSOMAL PROTEIN S25.;14 KDA PROTEIN.	**0.62**	0.00	1		**0.33**	0.00	1		**0.21**	0.00	1	
IPI00012772;IPI00797230	RPL8	60S RIBOSOMAL PROTEIN L8.;32 KDA PROTEIN.	**0.86**	0.07	6	0.177	**0.48**	0.01	6	0.076	**0.28**	0.01	6	0.099
IPI00012795	EIF3I	EUKARYOTIC TRANSLATION INITIATION FACTOR 3 SUBUNIT I.	**0.70**	0.00	1		**0.75**	0.00	1		**0.48**	0.00	1	
IPI00013004;IPI00216320;IPI00418202;IPI00791490	PDXK	ISOFORM 1 OF PYRIDOXAL KINASE.;ISOFORM 2 OF PYRIDOXAL KINASE.;PUTATIVE UNCHARACTERIZED PROTEIN PDXK.	**1.40**	0.01	2		**1.60**	0.04	3		**0.95**	0.04	2	
IPI00013068;IPI00796161	EIF3E	EUKARYOTIC TRANSLATION INITIATION FACTOR 3 SUBUNIT E.;20 KDA PROTEIN.							1				1	
IPI00013079	EMILIN1	EMILIN-1.	**3.41**	0.59	13	0.066	**5.71**	1.03	16	0.045	**4.39**	0.81	14	0.065
IPI00013296	RPS18;RPS18P9	40S RIBOSOMAL PROTEIN S18.	**1.14**	0.20	6	0.507	**0.98**	0.18	6	0.294			6	
IPI00013415;IPI00893703	RPS7	40S RIBOSOMAL PROTEIN S7.;PUTATIVE UNCHARACTERIZED PROTEIN RPS7.	**0.78**	0.08	3	0.285	**0.56**	0.20	3	0.299	**0.26**	0.05	3	
IPI00013452	EPRS	BIFUNCTIONAL AMINOACYL-TRNA SYNTHETASE.	**1.13**	0.00	4		**0.83**	0.00	3		**0.56**	0.00	3	
IPI00013468;IPI00514701;IPI00644108	BUB3	MITOTIC CHECKPOINT PROTEIN BUB3.;BUDDING UNINHIBITED BY BENZIMIDAZOLES 3 ISOFORM B.;32 KDA PROTEIN.	**0.78**	0.00	1		**0.78**	0.00	1		**0.64**	0.00	1	
IPI00013475;IPI00930715	TUBB2A	TUBULIN BETA-2A CHAIN.;"CDNA, FLJ93005, HIGHLY SIMILAR TO HOMO SAPIENS TUBULIN, BETA POLYPEPTIDE (TUBB), MRNA."	**1.17**	0.09	12	0.141	**1.22**	0.22	12	0.578	**1.00**	0.15	12	0.671
IPI00013508;IPI00759776;IPI00909239;IPI00921118	ACTN1	ALPHA-ACTININ-1.;"ACTININ, ALPHA 1 ISOFORM A.";"ACTININ, ALPHA 1 ISOFORM C.";ACTININ ALPHA 1 ISOFORM 3.	**1.69**	0.38	17	0.026	**1.66**	0.44	17	0.186	**2.05**	0.57	24	0.102
IPI00013808	ACTN4	ALPHA-ACTININ-4.	**1.20**	0.15	20	0.184	**1.21**	0.17	20	0.516	**1.25**	0.18	24	0.273
IPI00013847;IPI00792890	UQCRC1	CYTOCHROME B-C1 COMPLEX SUBUNIT 1, MITOCHONDRIAL.;"CDNA FLJ52292, HIGHLY SIMILAR TO UBIQUINOL-CYTOCHROME-C REDUCTASE COMPLEX COREPROTEIN I, MITOCHONDRIAL."	**1.06**	0.03	3	0.281	**0.92**	0.00	3		**0.43**	0.04	3	
IPI00013860	HIBADH	3-HYDROXYISOBUTYRATE DEHYDROGENASE, MITOCHONDRIAL.			1				1				1	
IPI00013877;IPI00216492;IPI00216493	HNRNPH3	ISOFORM 1 OF HETEROGENEOUS NUCLEAR RIBONUCLEOPROTEIN H3.;ISOFORM 2 OF HETEROGENEOUS NUCLEAR RIBONUCLEOPROTEIN H3.;ISOFORM 3 OF HETEROGENEOUS NUCLEAR RIBONUCLEOPROTEIN H3.	**0.99**	0.02	2	0.433	**1.06**	0.12	2	0.611	**0.78**	0.02	2	
IPI00013881;IPI00026230;IPI00479191;IPI00940955	HNRNPH1;RPL36AP37;HNRNPH2	HETEROGENEOUS NUCLEAR RIBONUCLEOPROTEIN H.;HETEROGENEOUS NUCLEAR RIBONUCLEOPROTEIN H2.;51 KDA PROTEIN.;"CDNA FLJ57964, HIGHLY SIMILAR TO HETEROGENEOUS NUCLEAR RIBONUCLEOPROTEIN H'."	**1.15**	0.04	3	0.426	**1.13**	0.00	1	0.603	**0.79**	0.00	2	0.516
IPI00013894	STIP1	STRESS-INDUCED-PHOSPHOPROTEIN 1.	**1.09**	0.07	3	0.358	**0.99**	0.00	3		**0.92**	0.13	3	0.591
IPI00013895	S100A11	PROTEIN S100-A11.			2				1				3	
IPI00013917	RPS12	40S RIBOSOMAL PROTEIN S12.			1				1				1	
IPI00013933;IPI00217182	DSP	ISOFORM DPI OF DESMOPLAKIN.;ISOFORM DPII OF DESMOPLAKIN.	**1.03**	0.00	1		**0.88**	0.00	1		**0.55**	0.00	1	
IPI00013955;IPI00218163;IPI00218164;IPI00218165;IPI00218168;IPI00218169;IPI00385172;IPI00385173;IPI00385178;IPI00607560;IPI00607569;IPI00607673;IPI00641632;IPI00646925;IPI00852677;IPI00855849;IPI00855927;IPI00856027;IPI00876878;IPI00889553;IPI00889566;IPI00889579;IPI00902840;IPI00909818;IPI00914966;IPI00916285;IPI00939413;IPI00940944;IPI00942342	MUC1	ISOFORM 1 OF MUCIN-1.;ISOFORM 2 OF MUCIN-1.;ISOFORM 3 OF MUCIN-1.;ISOFORM 4 OF MUCIN-1.;ISOFORM 6 OF MUCIN-1.;ISOFORM 7 OF MUCIN-1.;MUC1 ISOFORM M3.;MUCIN 1 ISOFORM 3 PRECURSOR.;MUC1 ISOFORM J24.;MUC1 ISOFORM M4.;MUCIN 1 ISOFORM 2 PRECURSOR.;ISOFORM 8 OF MUCIN-1.;MUCIN 1 ISOFORM 7 PRECURSOR.;MUCIN SHORT VARIANT E2.;MUC1 ISOFORM T11.;MUC1 ISOFORM M15.;MUC1 ISOFORM M8.;MUC1 ISOFORM M9.;MUC1 ISOFORM S-LSP.;"MUCIN 1, CELL SURFACE ASSOCIATED.";ISOFORM 9 OF MUCIN-1.;MUC1 ISOFORM M10.;ISOFORM 10 OF MUCIN-1.;"CDNA FLJ60927, HIGHLY SIMILAR TO MUCIN-1.";MUC1 ISOFORM J13.;MUC1 ISOFORM Z-LSP.;"HCG1996357, ISOFORM CRA_G.";MUCIN 1 ISOFORM 1 PRECURSOR.;60 KDA PROTEIN.			1				1		**1.77**	0.00	1	
IPI00013976;IPI00853454	LAMB1	LAMININ SUBUNIT BETA-1.;200 KDA PROTEIN.	**1.06**	0.06	4		**1.36**	0.06	5		**0.99**	0.10	4	0.627
IPI00014230	C1QBP	COMPLEMENT COMPONENT 1 Q SUBCOMPONENT-BINDING PROTEIN, MITOCHONDRIAL.	**1.00**	0.02	2		**6.13**	0.00	2		**1.21**	0.00	2	
IPI00014238;IPI00307092;IPI00445108	KARS	LYSYL-TRNA SYNTHETASE.;LYSYL-TRNA SYNTHETASE ISOFORM 1.;"CDNA FLJ44621 FIS, CLONE BRACE2016896, HIGHLY SIMILAR TO LYSYL-TRNA SYNTHETASE."			1				1				1	
IPI00014263	EIF4H	ISOFORM LONG OF EUKARYOTIC TRANSLATION INITIATION FACTOR 4H.	**0.56**	0.00	1		**0.74**	0.00	1				1	
IPI00014361;IPI00791788;IPI00909958	TSTA3	GDP-L-FUCOSE SYNTHETASE.;"CDNA FLJ39433 FIS, CLONE PROST2004142, HIGHLY SIMILAR TO GDP-L-FUCOSE SYNTHETASE.";"CDNA FLJ57685, HIGHLY SIMILAR TO GDP-L-FUCOSE SYNTHETASE."	**0.47**	0.00	1		**1.41**	0.00	1		**0.52**	0.00	1	
IPI00014375	ENPEP	GLUTAMYL AMINOPEPTIDASE.			1				1					
IPI00014424	EEF1A2	ELONGATION FACTOR 1-ALPHA 2.	**0.85**	0.12	11	0.074	**0.63**	0.11	11	0.071	**0.59**	0.14	11	0.071
IPI00014516;IPI00218694;IPI00218695;IPI00218696;IPI00333771;IPI00816770;IPI00925125	CALD1	ISOFORM 1 OF CALDESMON.;ISOFORM 2 OF CALDESMON.;ISOFORM 3 OF CALDESMON.;"CDNA FLJ53193, HIGHLY SIMILAR TO HOMO SAPIENS CALDESMON 1 (CALD1), TRANSCRIPT VARIANT 5, MRNA.";ISOFORM 5 OF CALDESMON.;PUTATIVE UNCHARACTERIZED PROTEIN DKFZP779D0769.;"CDNA FLJ76646, HIGHLY SIMILAR TO HOMO SAPIENS CALDESMON 1 (CALD1), TRANSCRIPT VARIANT 3, MRNA."	**2.73**	0.55	8	0.075	**4.73**	0.67	8	0.060	**6.99**	0.87	10	0.014
IPI00014625	CLCA1	CALCIUM-ACTIVATED CHLORIDE CHANNEL REGULATOR 1.			2									
IPI00015018	PPA1	INORGANIC PYROPHOSPHATASE.	**1.03**	0.00	1		**0.77**	0.00	1		**0.85**	0.00	1	
IPI00015148;IPI00019345;IPI00796349;IPI00816182;IPI00877120;IPI00908754;IPI00942831	RAP1B;RAP1A	RAS-RELATED PROTEIN RAP-1B.;RAS-RELATED PROTEIN RAP-1A.;18 KDA PROTEIN.;PNAS-140 (FRAGMENT).;"RAP1A, MEMBER OF RAS ONCOGENE FAMILY.";"CDNA FLJ50714, MODERATELY SIMILAR TO RAS-RELATED PROTEIN RAP-1B.";"CDNA FLJ50711, MODERATELY SIMILAR TO RAS-RELATED PROTEIN RAP-1B."	**1.11**	0.00	1	0.392	**1.52**	0.00	1	0.342				0.655
IPI00015262;IPI00398735;IPI00910593	CNN2	CALPONIN-2.;CALPONIN 2 ISOFORM B.;"CDNA FLJ52765, HIGHLY SIMILAR TO CALPONIN-2."							1				1	
IPI00015736;IPI00383579;IPI00791004	UBA5	UBIQUITIN-LIKE MODIFIER-ACTIVATING ENZYME 5.;UBIQUITIN-ACTIVATING ENZYME 5 ISOFORM 2.			1				1				1	
IPI00015842	RCN1	RETICULOCALBIN-1.							1				1	
IPI00015911;IPI00909143;IPI00926319	DLD	DIHYDROLIPOYL DEHYDROGENASE, MITOCHONDRIAL.;DIHYDROLIPOYL DEHYDROGENASE.;"CDNA FLJ56112, HIGHLY SIMILAR TO DIHYDROLIPOYL DEHYDROGENASE, MITOCHONDRIAL."	**0.94**	0.00	1		**0.71**	0.00	1		**0.57**	0.00	1	
IPI00016077;IPI00925073;IPI00925757;IPI00926531;IPI00926675	GBAS	PROTEIN NIPSNAP HOMOLOG 2.;10 KDA PROTEIN.;29 KDA PROTEIN.;PROTEIN.;8 KDA PROTEIN.	**0.84**	0.00	1		**0.72**	0.00	1		**0.33**	0.00	1	
IPI00016112;IPI00791587;IPI00952632	PXDN	ISOFORM 1 OF PEROXIDASIN HOMOLOG.;ISOFORM 2 OF PEROXIDASIN HOMOLOG.							1				1	
IPI00016342;IPI00947210	RAB7A	RAS-RELATED PROTEIN RAB-7A.;13 KDA PROTEIN.	**1.30**	0.13	4		**1.22**	0.03	4		**1.06**	0.07	3	
IPI00016346;IPI00796241;IPI00902466	PROSC	PROLINE SYNTHETASE CO-TRANSCRIBED BACTERIAL HOMOLOG PROTEIN.;14 KDA PROTEIN.;"CDNA FLJ11861 FIS, CLONE HEMBA1006885, HIGHLY SIMILAR TO PROLINE SYNTHETASE CO-TRANSCRIBED BACTERIAL HOMOLOG PROTEIN."	**0.77**	0.00	1		**0.80**	0.00	1	0.552			1	
IPI00016513	RAB10	RAS-RELATED PROTEIN RAB-10.	**1.29**	0.09	3		**1.15**	0.00	3		**1.38**	0.16	3	0.299
IPI00016610	PCBP1	POLY(RC)-BINDING PROTEIN 1.	**1.03**	0.11	4	0.371	**1.18**	0.03	4	0.545	**0.57**	0.11	4	0.433

IPI00016786;IPI00909484;IPI00910473	CDC42	ISOFORM 2 OF CELL DIVISION CONTROL PROTEIN 42 HOMOLOG.;"CDNA FLJ54776, HIGHLY SIMILAR TO CELL DIVISION CONTROL PROTEIN 42 HOMOLOG.";"CDNA FLJ55107, HIGHLY SIMILAR TO CELL DIVISION CONTROL PROTEIN 42 HOMOLOG."	**1.10**	0.00	2		**1.29**	0.00	2		**0.65**	0.02	2	
IPI00016801	GLUD1	GLUTAMATE DEHYDROGENASE 1, MITOCHONDRIAL.	**1.24**	0.16	5	0.337	**0.84**	0.04	5		**0.77**	0.09	5	
IPI00016832;IPI00472442;IPI00871889	PSMA1	ISOFORM SHORT OF PROTEASOME SUBUNIT ALPHA TYPE-1.;ISOFORM LONG OF PROTEASOME SUBUNIT ALPHA TYPE-1.;PROTEASOME SUBUNIT ALPHA TYPE.			1				1				1	
IPI00016862;IPI00759575;IPI00953236;IPI00953628;IPI00953696	GSR	ISOFORM MITOCHONDRIAL OF GLUTATHIONE REDUCTASE, MITOCHONDRIAL.;"ISOFORM CYTOPLASMIC OF GLUTATHIONE REDUCTASE, MITOCHONDRIAL."	**1.08**	0.08	2		**1.43**	0.00	2				2	
IPI00016910;IPI00646839;IPI00873286;IPI00910003;IPI00922360;IPI00939953	EIF3CL;EIF3C	EUKARYOTIC TRANSLATION INITIATION FACTOR 3 SUBUNIT C.;PUTATIVE UNCHARACTERIZED PROTEIN EIF3CL.;"CDNA FLJ55750, HIGHLY SIMILAR TO EUKARYOTIC TRANSLATION INITIATION FACTOR 3 SUBUNIT 8.";"CDNA FLJ53378, HIGHLY SIMILAR TO EUKARYOTIC TRANSLATION INITIATION FACTOR 3 SUBUNIT 8.";"CDNA FLJ54488, HIGHLY SIMILAR TO EUKARYOTIC TRANSLATION INITIATION FACTOR 3 SUBUNIT 8.";85 KDA PROTEIN.	**1.43**	0.00	1	0.469	**0.43**	0.00	1		**0.83**	0.00	1	0.535
IPI00017256;IPI00377066;IPI00478433;IPI00847168	RSU1	RAS SUPPRESSOR PROTEIN 1.;RAS SUPPRESSOR PROTEIN 1 ISOFORM 2.;RAS SUPPRESSOR PROTEIN 1 VARIANT 5.;RSU1 PROTEIN (FRAGMENT).			1				1				1	
IPI00017283	IARS2	ISOLEUCYL-TRNA SYNTHETASE, MITOCHONDRIAL.	**0.99**	0.00	1		**1.49**	0.00	1				1	
IPI00017292;IPI00219893;IPI00927799	CTNNB1	ISOFORM 1 OF CATENIN BETA-1.;"CDNA FLJ59565, HIGHLY SIMILAR TO BETA-CATENIN.";"CDNA FLJ57878, HIGHLY SIMILAR TO BETA-CATENIN."	**1.21**	0.15	3	0.339	**0.81**	0.10	3				3	
IPI00017297;IPI00789551	MATR3	MATRIN-3.;PUTATIVE UNCHARACTERIZED PROTEIN MATR3.	**1.26**	0.06	4	0.226	**1.15**	0.11	5		**0.71**	0.04	5	
IPI00017334	PHB	PROHIBITIN.	**1.02**	0.12	5	0.353	**0.78**	0.04	4	0.324	**0.52**	0.04	4	0.319
IPI00017367;IPI00903145	RDX	RADIXIN, ISOFORM CRA_A.;RADIXIN.				0.214	**1.75**	0.08	3	0.311				0.353
IPI00017376	SEC23B	PROTEIN TRANSPORT PROTEIN SEC23B.	**0.77**	0.10	3		**0.93**	0.00	3				3	
IPI00017448	LOC100291837;RPS21	40S RIBOSOMAL PROTEIN S21.			1		**0.63**	0.00	1				1	
IPI00017469;IPI00909276	SPR	SEPIAPTERIN REDUCTASE.;"CDNA FLJ56734, MODERATELY SIMILAR TO SEPIAPTERIN REDUCTASE."	**0.77**	0.05	2		**0.69**	0.16	2		**0.36**	0.00	3	
IPI00017510	MT-CO2;COX2	CYTOCHROME C OXIDASE SUBUNIT 2.	**1.09**	0.00	1	0.336	**0.47**	0.00	1	0.323	**0.34**	0.00	1	0.296
IPI00017526	S100P	PROTEIN S100-P.			1								2	
IPI00017704	COTL1	COACTOSIN-LIKE PROTEIN.	**0.47**	0.00	2		**2.03**	0.00	1		**1.40**	0.00	2	
IPI00017726;IPI00336094	HSD17B10	ISOFORM 1 OF 3-HYDROXYACYL-COA DEHYDROGENASE TYPE-2.;ISOFORM 2 OF 3-HYDROXYACYL-COA DEHYDROGENASE TYPE-2.	**0.79**	0.08	6	0.129	**0.64**	0.11	6				6	
IPI00017763;IPI00788840;IPI00789437;IPI00798071;IPI00941463	NAP1L4	CDNA FLJ59403, HIGHLY SIMILAR TO NUCLEOSOME ASSEMBLY PROTEIN 1-LIKE 4.;20 KDA PROTEIN.;32 KDA PROTEIN.;PUTATIVE UNCHARACTERIZED PROTEIN NAP1L4.;NUCLEOSOME ASSEMBLY PROTEIN 1-LIKE 4.	**0.80**	0.00	1		**1.02**	0.00	1		**0.30**	0.00	1	
IPI00017767	MGST2	MICROSOMAL GLUTATHIONE S-TRANSFERASE 2.	**0.87**	0.00	1		**1.07**	0.00	1		**0.88**	0.00	1	
IPI00017855;IPI00790739	ACO2	ACONITATE HYDRATASE, MITOCHONDRIAL.;"ACONITASE 2, MITOCHONDRIAL."	**0.91**	0.05	8	0.246	**1.04**	0.20	8		**0.44**	0.03	8	
IPI00017895;IPI00719611	GPD2	ISOFORM 1 OF GLYCEROL-3-PHOSPHATE DEHYDROGENASE, MITOCHONDRIAL.;"ISOFORM 2 OF GLYCEROL-3-PHOSPHATE DEHYDROGENASE, MITOCHONDRIAL."			1				1				1	
IPI00018140;IPI00402182;IPI00402183;IPI00402184;IPI00402185;IPI00930205	SYNCRIP	ISOFORM 1 OF HETEROGENEOUS NUCLEAR RIBONUCLEOPROTEIN Q.;ISOFORM 2 OF HETEROGENEOUS NUCLEAR RIBONUCLEOPROTEIN Q.;ISOFORM 3 OF HETEROGENEOUS NUCLEAR RIBONUCLEOPROTEIN Q.;ISOFORM 4 OF HETEROGENEOUS NUCLEAR RIBONUCLEOPROTEIN Q.;"SYNAPTOTAGMIN BINDING, CYTOPLASMIC RNA INTERACTING PROTEIN ISOFORM 2.";"SYNAPTOTAGMIN BINDING, CYTOPLASMIC RNA INTERACTING PROTEIN ISOFORM 5."	**1.08**	0.02	2	0.469	**0.81**	0.02	2	0.310	**0.54**	0.03	2	0.114
IPI00018146	YWHAQ	14-3-3 PROTEIN THETA.	**0.82**	0.05	7	0.184	**0.97**	0.12	7	0.578	**1.25**	0.12	8	0.680
IPI00018206;IPI00910267	GOT2	ASPARTATE AMINOTRANSFERASE, MITOCHONDRIAL.;ASPARTATE AMINOTRANSFERASE.	**0.81**	0.06	4	0.205	**0.68**	0.04	4	0.155	**0.53**	0.04	4	0.327
IPI00018219	TGFBI	TRANSFORMING GROWTH FACTOR-BETA-INDUCED PROTEIN IG-H3.	**2.94**	0.00	4		**5.63**	0.00	4		**7.15**	0.00	5	
IPI00018246;IPI00220663;IPI00220665;IPI00220667;IPI00903226	HK1	ISOFORM 1 OF HEXOKINASE-1.;ISOFORM 2 OF HEXOKINASE-1.;ISOFORM 3 OF HEXOKINASE-1.;ISOFORM 4 OF HEXOKINASE-1.;"CDNA FLJ46359 FIS, CLONE TESTI4049786, HIGHLY SIMILAR TO HEXOKINASE-1."	**2.02**	0.00	1		**2.17**	0.00	1		**2.51**	0.00	2	
IPI00018398	PSMC3	26S PROTEASE REGULATORY SUBUNIT 6A.	**0.62**	0.00	1				1		**0.73**	0.00	1	
IPI00018415;IPI00902914	TM9SF2	TRANSMEMBRANE 9 SUPERFAMILY MEMBER 2.;"CDNA FLJ36188 FIS, CLONE TESTI2027179, HIGHLY SIMILAR TO TRANSMEMBRANE 9 SUPERFAMILY PROTEIN MEMBER 2."	**0.78**	0.00	1		**0.20**	0.00	1		**0.30**	0.00	1	
IPI00018452;IPI00852917;IPI00894409;IPI00917016	CPNE1	COPINE-1.;COPINE I.;COPINE I ISOFORM B.;59 KDA PROTEIN.	**1.01**	0.11	3	0.469	**1.51**	0.00	2	0.578	**1.08**	0.00	1	0.668
IPI00018465;IPI00952607	CCT7	T-COMPLEX PROTEIN 1 SUBUNIT ETA.			1									
IPI00018534;IPI00020101;IPI00152906;IPI00303133;IPI00329665;IPI00419833;IPI00477495;IPI00554798;IPI00646240;IPI00794461;IPI00815755;IPI00930174;IPI00930570	HIST1H2BL;HIST1H2BC;HIST1H2BE;HIST1H2BI;HIST1H2BG;HIST1H2BF;HIST1H2BD;HIST1H2BH;HIST2H2BF;HIST1H2BK;H2BFS;HIST1H2BM;HIST1H2BN	HISTONE H2B TYPE 1-L.;"HISTONE CLUSTER 1, H2BG.";HISTONE H2B TYPE 1-D.;HISTONE H2B TYPE 1-H.;HISTONE H2B TYPE 2-F.;HISTONE H2B TYPE 1-K.;HISTONE H2B TYPE F-S.;HISTONE H2B TYPE 1-M.;HISTONE H2B.;HISTONE H2B TYPE 1-N.;HIST1H2BC PROTEIN.;"HISTONE CLUSTER 2, H2BF ISOFORM B."	**0.92**	0.15	5	0.354	**0.92**	0.12	5	0.545	**0.51**	0.06	5	0.340
IPI00018553;IPI00872452	CTRC	CHYMOTRYPSIN-C.;PUTATIVE UNCHARACTERIZED PROTEIN CTRC.	**0.92**	0.23	13	0.186	**0.44**	0.09	13	0.067	**0.03**	0.00	13	
IPI00018755;IPI00022796;IPI00419258;IPI00644653;IPI00645948;IPI00742929;IPI00872915;IPI00879051;IPI00903004;IPI00943040	HMGB1L10;HMGB1L1;HMGB1	HIGH MOBILITY GROUP PROTEIN 1-LIKE 10.;PUTATIVE HIGH MOBILITY GROUP PROTEIN B1-LIKE 1.;HIGH MOBILITY GROUP PROTEIN B1.;"CDNA FLJ56337, HIGHLY SIMILAR TO HIGH MOBILITY GROUP PROTEIN B1.";HIGH-MOBILITY GROUP BOX 1.;SIMILAR TO HIGH MOBILITY GROUP PROTEIN B1.;19 KDA PROTEIN.;15 KDA PROTEIN.;"CDNA FLJ32558 FIS, CLONE SPLEN1000143, HIGHLY SIMILAR TO HIGH MOBILITY GROUP PROTEIN B1.";18 KDA PROTEIN.	**0.81**	0.00	2		**3.04**	0.00	2		**0.96**	0.00	2	
IPI00018769;IPI00642455	THBS2	THROMBOSPONDIN-2.;THROMBOSPONDIN 2.							1				3	
IPI00018931;IPI00943562	VPS35;LOC100133770	VACUOLAR PROTEIN SORTING-ASSOCIATED PROTEIN 35.;SIMILAR TO VACUOLAR PROTEIN SORTING 35.			1				1		**1.39**	0.00	1	
IPI00019038	LYZ	LYSOZYME C.	**2.11**	0.00	1		**2.53**	0.00	1		**4.08**	0.00	1	
IPI00019359	KRT9	KERATIN, TYPE I CYTOSKELETAL 9.	**1.02**	0.18	3		**2.60**	0.00	5		**3.88**	0.00	5	
IPI00019385;IPI00646864;IPI00647421	SSR4	TRANSLOCON-ASSOCIATED PROTEIN SUBUNIT DELTA PRECURSOR.;PROTEIN.;PUTATIVE UNCHARACTERIZED PROTEIN SSR4.	**0.55**	0.03	4	0.069	**0.29**	0.04	4	0.082	**0.11**	0.00	4	0.103
IPI00019449	RNASE2	NON-SECRETORY RIBONUCLEASE.			1				1				1	
IPI00019463;IPI00385404;IPI00807398	EIF2AK2	INTERFERON-INDUCED, DOUBLE-STRANDED RNA-ACTIVATED PROTEIN KINASE.;EUKARYOTIC TRANSLATION INITIATION FACTOR 2-ALPHA KINASE 2 ISOFORM B.;EIF2AK2 PROTEIN (FRAGMENT).											1	
IPI00019502	MYH9	ISOFORM 1 OF MYOSIN-9.	**1.54**	0.17	42	0.001	**1.88**	0.27	45	0.001	**2.22**	0.35	54	0.000
IPI00019568	F2	PROTHROMBIN (FRAGMENT).							2				2	
IPI00019580	PLG	PLASMINOGEN.									**1.64**	0.11	3	
IPI00019599;IPI00019600;IPI00030962;IPI00397947;IPI00447356;IPI00472498;IPI00514724;IPI00789287;IPI00797889;IPI00890717	UBE2V1;UBE2V2;TMEM189-UBE2V1	ISOFORM 1 OF UBIQUITIN-CONJUGATING ENZYME E2 VARIANT 1.;UBIQUITIN-CONJUGATING ENZYME E2 VARIANT 2.;ISOFORM 5 OF UBIQUITIN-CONJUGATING ENZYME E2 VARIANT 1.;TMEM189-UBE2V1 READTHROUGH TRANSCRIPT.;ISOFORM 2 OF UBIQUITIN-CONJUGATING ENZYME E2 VARIANT 1.;ISOFORM 3 OF UBIQUITIN-CONJUGATING ENZYME E2 VARIANT 1.;UBIQUITIN-CONJUGATING ENZYME E2 VARIANT 1.;12 KDA PROTEIN.;16 KDA PROTEIN.;42 KDA PROTEIN.	**1.14**	0.00	1		**0.67**	0.00	1		**0.69**	0.00	1	
IPI00019755	GSTO1	GLUTATHIONE S-TRANSFERASE OMEGA-1.	**2.02**	0.00	1		**2.22**	0.00	1		**1.33**	0.00	3	
IPI00019770	FAU	UBIQUITIN-LIKE PROTEIN FUBI AND RIBOSOMAL PROTEIN S30 PRECURSOR.	**0.61**	0.00	2		**0.31**	0.00	2		**0.19**	0.00	2	
IPI00019901;IPI00220158;IPI00253279;IPI00376220	ADD1	ISOFORM 1 OF ALPHA-ADDUCIN.;ISOFORM 3 OF ALPHA-ADDUCIN.;ADDUCIN 1.;ADDUCIN 1 (ALPHA) ISOFORM D.	**1.36**	0.00	1		**1.14**	0.00	1		**0.75**	0.00	1	
IPI00019912;IPI00847995;IPI00909582	HSD17B4	PEROXISOMAL MULTIFUNCTIONAL ENZYME TYPE 2.;"CDNA FLJ61268, HIGHLY SIMILAR TO PEROXISOMAL MULTIFUNCTIONAL ENZYME TYPE 2.";"CDNA FLJ55431, HIGHLY SIMILAR TO PEROXISOMAL MULTIFUNCTIONAL ENZYME TYPE 2."	**0.78**	0.00	2				2		**0.84**	0.00	2	
IPI00020005	ARSE	CDNA FLJ50239, HIGHLY SIMILAR TO ARYLSULFATASE E.	**0.74**	0.00	1				1				1	
IPI00020017	C10orf116	ADIPOSE MOST ABUNDANT GENE TRANSCRIPT 2 PROTEIN.							1					
IPI00020042;IPI00216770;IPI00738042	PSMC4;LOC652826	ISOFORM 1 OF 26S PROTEASE REGULATORY SUBUNIT 6B.;ISOFORM 2 OF 26S PROTEASE REGULATORY SUBUNIT 6B.;SIMILAR TO PROTEASOME 26S ATPASE SUBUNIT 4.			1				1		**0.30**	0.00	1	
IPI00020194;IPI00221354;IPI00260715;IPI00294426;IPI00428056;IPI00645208;IPI00854677;IPI00909890;IPI00941083	TAF15;FUS	ISOFORM SHORT OF TATA-BINDING PROTEIN-ASSOCIATED FACTOR 2N.;ISOFORM SHORT OF RNA-BINDING PROTEIN FUS.;FUS-LIKE PROTEIN (FRAGMENT).;ISOFORM LONG OF TATA-BINDING PROTEIN-ASSOCIATED FACTOR 2N.;BBF2H7/FUS PROTEIN (FRAGMENT).;53 KDA PROTEIN.;PUTATIVE UNCHARACTERIZED PROTEIN FUS.;"CDNA FLJ58049, HIGHLY SIMILAR TO RNA-BINDING PROTEIN FUS."	**1.55**	0.00	1		**2.22**	0.00	2		**1.34**	0.00	1	
IPI00020418	RRAS	RAS-RELATED PROTEIN R-RAS.											1	

IPI00020436;IPI00429190;IPI00795676;IPI00930720;IPI00941638	RAB11B;RAB11A	RAS-RELATED PROTEIN RAB-11B.;"CDNA FLJ61136, HIGHLY SIMILAR TO RAS-RELATED PROTEIN RAB-11A.";22 KDA PROTEIN.;"CDNA FLJ61134, HIGHLY SIMILAR TO RAS-RELATED PROTEIN RAB-11B.";RAS-RELATED PROTEIN RAB-11A.							1				1	
IPI00020501;IPI00024870;IPI00743857;IPI00744256;IPI00873982	MYH11	MYOSIN-11.;SMOOTH MUSCLE MYOSIN HEAVY CHAIN 11 ISOFORM SM2A.;SMOOTH MUSCLE MYOSIN HEAVY CHAIN 11 ISOFORM SM1B.;SMOOTH MUSCLE MYOSIN HEAVY CHAIN 11 ISOFORM SM2B.;MYOSIN HEAVY CHAIN 11 SMOOTH MUSCLE ISOFORM.	**2.63**	0.64	42	0.040	**2.98**	0.84	35	0.015	**3.16**	0.73	45	0.090
IPI00020557	LRP1	PROLOW-DENSITY LIPOPROTEIN RECEPTOR-RELATED PROTEIN 1.							1				1	
IPI00020567	ARHGAP1	RHO GTPASE-ACTIVATING PROTEIN 1.	**0.76**	0.02	3		**1.38**	0.00	4		**1.32**	0.01	5	
IPI00020599	CALR	CALRETICULIN.	**0.98**	0.14	10	0.376	**0.84**	0.10	11	0.260	**0.46**	0.00	10	
IPI00020850;IPI00184845;IPI00332511;IPI00376972;IPI00376973;IPI00376974;IPI00442821;IPI00470920;IPI00472385;IPI00798041;IPI00944548;IPI00944557	PPP2R2B;PPP2R2D;PPP2R2A	ISOFORM 1 OF SERINE/THREONINE-PROTEIN PHOSPHATASE 2A 55 KDA REGULATORY SUBUNIT B BETA ISOFORM.;UNCHARACTERIZED HEMATOPOIETIC STEM/PROGENITOR CELLS PROTEIN MDS026.;SERINE/THREONINE-PROTEIN PHOSPHATASE 2A 55 KDA REGULATORY SUBUNIT B ALPHA ISOFORM.;ISOFORM 2 OF SERINE/THREONINE-PROTEIN PHOSPHATASE 2A 55 KDA REGULATORY SUBUNIT B BETA ISOFORM.;ISOFORM 5 OF SERINE/THREONINE-PROTEIN PHOSPHATASE 2A 55 KDA REGULATORY SUBUNIT B BETA ISOFORM.;"BETA ISOFORM OF REGULATORY SUBUNIT B55, PROTEIN PHOSPHATASE 2 ISOFORM D.";"CDNA FLJ26613 FIS, CLONE MPB05565, HIGHLY SIMILAR TO SERINE/THREONINE PROTEIN PHOSPHATASE 2A, 55 KDA REGULATORY SUBUNIT B, ALPHA ISOFORM.";SERINE/THREONINE-PROTEIN PHOSPHATASE 2A 55 KDA REGULATORY SUBUNIT B DELTA ISOFORM.;"PROTEIN PHOSPHATASE 2, REGULATORY SUBUNIT B, DELTA ISOFORM 2.";"CDNA FLJ58665, HIGHLY SIMILAR TO SERINE/THREONINE-PROTEIN PHOSPHATASE 2A 55 KDA REGULATORY SUBUNIT B ALPHA ISOFORM.";ISOFORM 4 OF SERINE/THREONINE-PROTEIN PHOSPHATASE 2A 55 KDA REGULATORY SUBUNIT B BETA ISOFORM.;ISOFORM 3 OF SERINE/THREONINE-PROTEIN PHOSPHATASE 2A 55 KDA REGULATORY SUBUNIT B BETA ISOFORM.	**0.85**	0.00	1		**0.79**	0.00	1		**0.53**	0.00	1	
IPI00020956;IPI00514127;IPI00514330;IPI00922032	HDGF	HEPATOMA-DERIVED GROWTH FACTOR.;HEPATOMA-DERIVED GROWTH FACTOR ISOFORM B.;HEPATOMA-DERIVED GROWTH FACTOR ISOFORM C.;"CDNA FLJ55039, MODERATELY SIMILAR TO HEPATOMA-DERIVED GROWTH FACTOR."	**1.09**	0.00	1				1				1	
IPI00020984;IPI00941747	CANX	CDNA FLJ55574, HIGHLY SIMILAR TO CALNEXIN.;CALNEXIN.	**1.23**	0.16	18	0.469	**0.85**	0.17	19	0.139	**0.51**	0.12	18	0.043
IPI00020986	LUM	LUMICAN.	**6.29**	1.09	12	0.028	**12.82**	1.75	12	0.015	**13.05**	2.06	12	0.014
IPI00020987	PRELP	PROLARGIN.	**7.14**	0.00	3		**10.81**	0.00	4	0.202	**29.40**	0.00	5	
IPI00020996;IPI00925635	IGFALS	INSULIN-LIKE GROWTH FACTOR-BINDING PROTEIN COMPLEX ACID LABILE CHAIN.;"INSULIN-LIKE GROWTH FACTOR BINDING PROTEIN, ACID LABILE SUBUNIT ISOFORM 1 PRECURSOR."											1	
IPI00021033	COL3A1	ISOFORM 1 OF COLLAGEN ALPHA-1(III) CHAIN.	**8.17**	0.45	2		**14.99**	1.68	2		**11.44**	1.28	2	
IPI00021048;IPI00216268;IPI00216269;IPI00385318;IPI00645867	MYOF	ISOFORM 1 OF MYOFERLIN.;ISOFORM 2 OF MYOFERLIN.;ISOFORM 3 OF MYOFERLIN.;ISOFORM 5 OF MYOFERLIN.;ISOFORM 6 OF MYOFERLIN.							1				1	
IPI00021263	YWHAZ	14-3-3 PROTEIN ZETA/DELTA.	**1.29**	0.22	12	0.449	**1.28**	0.17	12	0.547	**2.02**	0.42	15	0.302
IPI00021264	CNN1	CALPONIN-1.									**4.31**	0.00	3	
IPI00021290;IPI00394838;IPI00935456;IPI00939422	ACLY	ATP-CITRATE SYNTHASE.;"CDNA FLJ56442, HIGHLY SIMILAR TO ATP-CITRATE SYNTHASE.";"CDNA FLJ55447, HIGHLY SIMILAR TO ATP-CITRATE SYNTHASE.";ATP CITRATE LYASE ISOFORM 2.	**1.50**	0.22	3		**1.35**	0.27	3				3	
IPI00021302	SUSD2	SUSHI DOMAIN-CONTAINING PROTEIN 2.							1				1	
IPI00021304	KRT2	KERATIN, TYPE II CYTOSKELETAL 2 EPIDERMAL.	**1.11**	0.03	6	0.452	**1.94**	0.37	6	0.333	**1.69**	0.43	7	0.535
IPI00021347;IPI00909773;IPI00909975	UBE2L3	UBIQUITIN-CONJUGATING ENZYME E2 L3.;UBIQUITIN CARRIER PROTEIN.	**0.92**	0.00	1		**0.98**	0.00	1		**0.73**	0.00	1	
IPI00021405;IPI00216953;IPI00910241	LMNA	ISOFORM A OF LAMIN-A/C.;ISOFORM ADELTA10 OF LAMIN-A/C.;"CDNA FLJ56081, HIGHLY SIMILAR TO LAMIN-A/C."	**1.86**	0.21	23		**2.34**	0.33	21		**1.90**	0.42	23	
IPI00021439;IPI00021440;IPI00894365;IPI00894498;IPI00930226	ACTB;ACTG1;ACTB;ACTG1	ACTIN, CYTOPLASMIC 1.;"ACTIN, CYTOPLASMIC 2.";"CDNA FLJ52842, HIGHLY SIMILAR TO ACTIN, CYTOPLASMIC 1.";BETA ACTIN VARIANT (FRAGMENT).;"CDNA FLJ57283, HIGHLY SIMILAR TO ACTIN, CYTOPLASMIC 2."	**1.83**	0.24	21	0.001	**2.40**	0.29	20	0.004	**2.42**	0.35	21	0.001
IPI00021447	AMY2B	ALPHA-AMYLASE 2B.	**0.90**	0.20	30	0.186	**0.38**	0.14	31	0.000	**0.03**	0.01	30	
IPI00021727	C4BPA	C4B-BINDING PROTEIN ALPHA CHAIN.											1	
IPI00021728;IPI00176637	EIF2S2;EIF2S2;LOC728350	EUKARYOTIC TRANSLATION INITIATION FACTOR 2 SUBUNIT 2.;EUKARYOTIC TRANSLATION INITIATION FACTOR 2 SUBUNIT 2-LIKE PROTEIN.	**0.94**	0.00	1				1		**0.44**	0.00	1	
IPI00021766;IPI00298289;IPI00478442	RTN4	ISOFORM 1 OF RETICULON-4.;ISOFORM 2 OF RETICULON-4.;ISOFORM 5 OF RETICULON-4.	**1.73**	0.00	1		**2.90**	0.00	1		**2.21**	0.00	1	
IPI00021772	MAT1A	S-ADENOSYLMETHIONINE SYNTHETASE ISOFORM TYPE-1.	**0.72**	0.00	1		**0.71**	0.00	1				1	
IPI00021792;IPI00029251	PLA2G1B	PHOSPHOLIPASE A2.;PLA2G1B PROTEIN.	**1.15**	0.11	2				2		**0.04**	0.00	2	
IPI00021812	AHNAK	NEUROBLAST DIFFERENTIATION-ASSOCIATED PROTEIN AHNAK.	**2.67**	0.38	12	0.028	**4.39**	0.65	14	0.013	**2.82**	0.37	12	0.015
IPI00021828	CSTB	CYSTATIN-B.											1	
IPI00021831	PRKAR1A	CAMP-DEPENDENT PROTEIN KINASE TYPE I-ALPHA REGULATORY SUBUNIT.			1		**0.76**	0.00	1				1	
IPI00021840;IPI00640929;IPI00643522	RPS6	40S RIBOSOMAL PROTEIN S6.;RIBOSOMAL PROTEIN S6.;"RIBOSOMAL PROTEIN S6, ISOFORM CRA_A."	**1.23**	0.00	1				1				1	
IPI00021841	APOA1	APOLIPOPROTEIN A-I.	**1.22**	0.20	12	0.391	**2.70**	0.68	12	0.025	**1.38**	0.42	12	0.510
IPI00021842;IPI00878953;IPI00879368;IPI00879456	APOE	APOLIPOPROTEIN E.;MRNA FOR APOLIPOPROTEIN E.;25 KDA PROTEIN.							2				1	
IPI00021854	APOA2	APOLIPOPROTEIN A-II.	**0.89**	0.10	2		**5.08**	0.00	2		**4.93**	0.51	2	
IPI00021857;IPI00657670	APOC3	APOLIPOPROTEIN C-III.;APOLIPOPROTEIN C-III VARIANT 1.			2				2				2	
IPI00021885;IPI00029717	FGA	ISOFORM 1 OF FIBRINOGEN ALPHA CHAIN.;ISOFORM 2 OF FIBRINOGEN ALPHA CHAIN.	**1.41**	0.41	17	0.469	**2.27**	0.55	15	0.283	**2.42**	0.47	17	0.092
IPI00021888;IPI00910147	SLC39A7	ZINC TRANSPORTER SLC39A7.;"CDNA FLJ52943, HIGHLY SIMILAR TO ZINC TRANSPORTER SLC39A7."	**1.01**	0.00	1				1				1	
IPI00021891;IPI00219713;IPI00877703;IPI00877792	FGG	ISOFORM GAMMA-B OF FIBRINOGEN GAMMA CHAIN.;ISOFORM GAMMA-A OF FIBRINOGEN GAMMA CHAIN.;52 KDA PROTEIN.;50 KDA PROTEIN.	**1.32**	0.35	11	0.510	**2.65**	0.81	11	0.296	**2.30**	0.36	11	0.225
IPI00021926;IPI00178056;IPI00910829;IPI00926977	PSMC6	26S PROTEASE REGULATORY SUBUNIT S10B.;44 KDA PROTEIN.;"CDNA FLJ57570, HIGHLY SIMILAR TO 26S PROTEASE REGULATORY SUBUNIT S10B.";PROTEASOME 26S ATPASE SUBUNIT 6.			1									
IPI00022018;IPI00641116;IPI00790317;IPI00796864	DPM1	DOLICHOL-PHOSPHATE MANNOSYLTRANSFERASE.;"DOLICHYL-PHOSPHATE MANNOSYLTRANSFERASE POLYPEPTIDE 1, CATALYTIC SUBUNIT."			1				1				1	
IPI00022143;IPI00746655;IPI00902463	ESYT1	ISOFORM 1 OF EXTENDED SYNAPTOTAGMIN-1.;ISOFORM 2 OF EXTENDED SYNAPTOTAGMIN-1.;"CDNA FLJ46898 FIS, CLONE UTERU3022168, HIGHLY SIMILAR TO PROTEIN FAM62A."	**2.11**	0.00	2		**1.62**	0.00	2		**1.34**	0.00	2	
IPI00022200;IPI00220701	COL6A3	ISOFORM 1 OF COLLAGEN ALPHA-3(VI) CHAIN.;ISOFORM 2 OF COLLAGEN ALPHA-3(VI) CHAIN.	**4.04**	0.62	92	0.000	**4.82**	0.75	91	0.000	**3.82**	0.83	87	0.000
IPI00022202;IPI00215777;IPI00788624;IPI00790115;IPI00791315;IPI00793177	SLC25A3	ISOFORM A OF PHOSPHATE CARRIER PROTEIN, MITOCHONDRIAL.;"ISOFORM B OF PHOSPHATE CARRIER PROTEIN, MITOCHONDRIAL.";31 KDA PROTEIN.;"CDNA FLJ90278 FIS, CLONE NT2RP1000325, HIGHLY SIMILAR TO PHOSPHATE CARRIER PROTEIN, MITOCHONDRIALPRECURSOR.";21 KDA PROTEIN.;24 KDA PROTEIN.	**1.02**	0.00	1		**0.82**	0.00	1		**0.44**	0.00	1	
IPI00022213;IPI00383359;IPI00607684;IPI00792533;IPI00910787	PGC	GASTRICSIN.;PGC PROTEIN.;PROGASTRICSIN.;"CDNA FLJ58861, HIGHLY SIMILAR TO GASTRICSIN."	**3.14**	0.00	1				1				1	
IPI00022228	HDLBP	VIGILIN.	**0.70**	0.05	5	0.163	**0.72**	0.03	4				6	
IPI00022229	APOB	APOLIPOPROTEIN B-100.	**0.78**	0.00	3		**0.82**	0.00	5		**1.54**	0.00	6	
IPI00022232	CLPS	COLIPASE.			1		**1.84**	0.00	1				1	
IPI00022246	AZU1	AZUROCIDIN.			1		**1.27**	0.00	1		**1.10**	0.00	1	
IPI00022255	OLFM4	OLFACTOMEDIN-4.							1				4	
IPI00022300;IPI00910039	METTL7A	METHYLTRANSFERASE-LIKE PROTEIN 7A.;"CDNA FLJ52156, MODERATELY SIMILAR TO MUS MUSCULUS METHYLTRANSFERASE LIKE 7A (METTL7A), MRNA."	**1.26**	0.00	1		**0.87**	0.00	1		**0.59**	0.00	1	
IPI00022314;IPI00847322;IPI00911015	SOD2	SUPEROXIDE DISMUTASE [MN], MITOCHONDRIAL.;MANGANESE SUPEROXIDE DISMUTASE ISOFORM A PRECURSOR.;SUPEROXIDE DISMUTASE.	**1.28**	0.07	3	0.376	**1.38**	0.12	3	0.552	**0.55**	0.00	3	
IPI00022348;IPI00220453;IPI00291097;IPI00303999;IPI00304000;IPI00332110;IPI00382504;IPI00395707;IPI00395893;IPI00744329;IPI00876842;IPI00908329;IPI00922350;IPI00922504;IPI00940182	PML	ISOFORM PML-1 OF PROBABLE TRANSCRIPTION FACTOR PML.;ISOFORM PML-X OF PROBABLE TRANSCRIPTION FACTOR PML.;PROMYELOCYTIC LEUKEMIA PROTEIN ISOFORM 11.;ISOFORM PML-2 OF PROBABLE TRANSCRIPTION FACTOR PML.;ISOFORM PML-3 OF PROBABLE TRANSCRIPTION FACTOR PML.;PROMYELOCYTIC LEUKEMIA PROTEIN ISOFORM 9.;TRIPARTITE MOTIF PROTEIN TRIM19 DELTA.;PROMYELOCYTIC LEUKEMIA PROTEIN ISOFORM 7.;PROMYELOCYTIC LEUKEMIA PROTEIN ISOFORM 8.;PML PROTEIN.;PML-RAR PROTEIN.;"CDNA FLJ58284, MODERATELY SIMILAR TO PROBABLE TRANSCRIPTION FACTOR PML.";ISOFORM PML-4 OF PROBABLE TRANSCRIPTION FACTOR PML.;ISOFORM PML-5 OF PROBABLE TRANSCRIPTION FACTOR PML.;ISOFORM PML-3B OF PROBABLE TRANSCRIPTION FACTOR PML.			1				1				1	
IPI00022371	HRG	HISTIDINE-RICH GLYCOPROTEIN.	**1.87**	0.25	2		**3.13**	0.75	6		**2.19**	0.39	4	
IPI00022391	APCS	SERUM AMYLOID P-COMPONENT.	**1.68**	0.06	3	0.344	**3.40**	0.82	3	0.173	**1.60**	0.21	3	0.496
IPI00022395	C9	COMPLEMENT COMPONENT C9.	**1.18**	0.00	1		**2.41**	0.00	2		**1.48**	0.00	1	

IPI00022418;IPI00339223;IPI00339225;IPI00339228;IPI00414283;IPI00855777	FN1	ISOFORM 1 OF FIBRONECTIN.;ISOFORM 3 OF FIBRONECTIN.;ISOFORM 5 OF FIBRONECTIN.;ISOFORM 8 OF FIBRONECTIN.;ISOFORM 9 OF FIBRONECTIN.;ISOFORM 14 OF FIBRONECTIN.	**2.16**	0.26	16	0.177	**5.08**	0.89	28	0.003	**5.75**	1.10	29	0.039
IPI00022426;IPI00922298	AMBP	PROTEIN AMBP.;"CDNA FLJ51445, HIGHLY SIMILAR TO AMBP PROTEIN."	**1.44**	0.00	1						**1.87**	0.00	2	
IPI00022429;IPI00884926	ORM1	ALPHA-1-ACID GLYCOPROTEIN 1.;OROSOMUCOID 1 PRECURSOR.	**1.79**	0.19	6	0.395	**4.74**	0.42	6	0.076	**6.19**	0.57	8	0.374
IPI00022432;IPI00855916	TTR	TRANSTHYRETIN.	**1.68**	0.24	12	0.262	**3.22**	0.53	13	0.040	**1.52**	0.28	12	0.526
IPI00022433;IPI00908768	HSPB6	HEAT SHOCK PROTEIN BETA-6.;"CDNA FLJ51906, HIGHLY SIMILAR TO HEAT-SHOCK PROTEIN BETA-6."	**1.67**	0.00	2		**2.65**	0.00	2		**1.28**	0.00	2	
IPI00022434	ALB	PUTATIVE UNCHARACTERIZED PROTEIN ALB.	**1.87**	0.67	42	0.262	**3.80**	1.02	40	0.000	**4.36**	0.91	42	0.004
IPI00022463	TF	SEROTRANSFERRIN.	**2.86**	1.12	6	0.202	**4.13**	0.37	7	0.033	**4.27**	1.51	7	0.099
IPI00022488	HPX	HEMOPEXIN.			2				2				2	
IPI00022624	GPRC5A	RETINOIC ACID-INDUCED PROTEIN 3.			1								1	
IPI00022774	VCP	TRANSITIONAL ENDOPLASMIC RETICULUM ATPASE.	**0.91**	0.11	15	0.285	**0.88**	0.06	15	0.152	**0.54**	0.05	15	0.054
IPI00022792;IPI00793751	MFAP4	MICROFIBRIL-ASSOCIATED GLYCOPROTEIN 4.;"CDNA FLJ50064, HIGHLY SIMILAR TO MICROFIBRIL-ASSOCIATED GLYCOPROTEIN 4."	**1.74**	0.00	1		**3.01**	0.00	1		**1.79**	0.00	1	
IPI00022793	HADHB	TRIFUNCTIONAL ENZYME SUBUNIT BETA, MITOCHONDRIAL.	**0.97**	0.02	3	0.285	**0.73**	0.15	2	0.578	**0.86**	0.00	3	
IPI00022822;IPI00414694;IPI00479309;IPI00783931;IPI00828004	COL18A1	ISOFORM 2 OF COLLAGEN ALPHA-1(XVIII) CHAIN.;ISOFORM 3 OF COLLAGEN ALPHA-1(XVIII) CHAIN.;PUTATIVE UNCHARACTERIZED PROTEIN COL18A1.;ISOFORM 1 OF COLLAGEN ALPHA-1(XVIII) CHAIN.;MULTI-FUNCTIONAL PROTEIN MFP.	**1.43**	0.10	2	0.184	**1.56**	0.16	2	0.260	**0.72**	0.02	2	0.671
IPI00022830;IPI00100197;IPI00376904;IPI00397571	NSFL1C	ISOFORM 2 OF NSFL1 COFACTOR P47.;ISOFORM 1 OF NSFL1 COFACTOR P47.;P47 PROTEIN ISOFORM C.;ISOFORM 3 OF NSFL1 COFACTOR P47.			1				1		**1.00**	0.00	1	
IPI00022892;IPI00555577	THY1	THY-1 MEMBRANE GLYCOPROTEIN.;THY-1 CELL SURFACE ANTIGEN VARIANT (FRAGMENT).	**4.70**	0.00	1		**3.31**	0.00	2		**2.75**	0.00	2	
IPI00022895;IPI00644018;IPI00646799	A1BG	ALPHA-1B-GLYCOPROTEIN.;41 KDA PROTEIN.;"CDNA FLJ31323 FIS, CLONE LIVER2000037, HIGHLY SIMILAR TO ALPHA-1B- GLYCOPROTEIN."											2	
IPI00022977;IPI00908811	CKB	CREATINE KINASE B-TYPE.;"CDNA FLJ52237, HIGHLY SIMILAR TO CREATINE KINASE B-TYPE."			3				2				1	
IPI00023001;IPI00945585	FAM162A	UPF0389 PROTEIN FAM162A.;17 KDA PROTEIN.	**0.60**	0.00	1		**0.54**	0.00	1				1	
IPI00023048;IPI00642971;IPI00789435	EEF1D	ISOFORM 1 OF ELONGATION FACTOR 1-DELTA.;ISOFORM 2 OF ELONGATION FACTOR 1-DELTA.;EUKARYOTIC TRANSLATION ELONGATION FACTOR 1 DELTA ISOFORM 3.	**1.71**	0.00	3				3		**0.93**	0.00	4	
IPI00023122;IPI00023560;IPI00030835;IPI00060308;IPI00478185;IPI00655883	PDLIM7	ISOFORM 1 OF PDZ AND LIM DOMAIN PROTEIN 7.;ISOFORM 2 OF PDZ AND LIM DOMAIN PROTEIN 7.;ISOFORM 3 OF PDZ AND LIM DOMAIN PROTEIN 7.;ISOFORM 6 OF PDZ AND LIM DOMAIN PROTEIN 7.;ISOFORM 4 OF PDZ AND LIM DOMAIN PROTEIN 7.;ISOFORM 5 OF PDZ AND LIM DOMAIN PROTEIN 7.			1				2				2	
IPI00023191;IPI00879576;IPI00879680;IPI00903336;IPI00910932;IPI00913835;IPI00941588;IPI00942537	TOM1	CDNA FLJ54710, HIGHLY SIMILAR TO TARGET OF MYB PROTEIN 1.;TARGET OF MYB1.;TARGET OF MYB1 ISOFORM 3.;"TARGET OF MYB1 (CHICKEN), ISOFORM CRA_A.";"CDNA FLJ54716, HIGHLY SIMILAR TO TARGET OF MYB PROTEIN 1.";TARGET OF MYB PROTEIN 1.;TARGET OF MYB1 ISOFORM 2.;TOM1.	**1.18**	0.00	1		**1.44**	0.00	1				1	
IPI00023591	PURA	TRANSCRIPTIONAL ACTIVATOR PROTEIN PUR-ALPHA.	**1.05**	0.06	3	0.469	**1.31**	0.10	4	0.498	**0.57**	0.00	3	
IPI00023598;IPI00926685	TUBB4	TUBULIN BETA-4 CHAIN.;"CDNA FLJ53341, HIGHLY SIMILAR TO TUBULIN BETA-4 CHAIN."									**0.66**	0.13	12	
IPI00023635;IPI00555726;IPI00639910	IMPA2	ISOFORM 1 OF INOSITOL MONOPHOSPHATASE 2.;ISOFORM 2 OF INOSITOL MONOPHOSPHATASE 2.;INOSINE MONOPHOSPHATASE 2.	**0.68**	0.00	1				1				1	
IPI00023673	LGALS3BP	GALECTIN-3-BINDING PROTEIN.											1	
IPI00023728	GGH	GAMMA-GLUTAMYL HYDROLASE.											2	
IPI00023748;IPI00797126;IPI00797259;IPI00909970	NACA	NASCENT POLYPEPTIDE-ASSOCIATED COMPLEX SUBUNIT ALPHA.;NASCENT POLYPEPTIDE-ASSOCIATED COMPLEX ALPHA SUBUNIT ISOFORM A.;PUTATIVE UNCHARACTERIZED PROTEIN NACA.;"CDNA FLJ53704, MODERATELY SIMILAR TO NASCENT POLYPEPTIDE-ASSOCIATED COMPLEX SUBUNIT ALPHA."	**1.24**	0.00	2		**0.47**	0.17	2		**0.27**	0.00	2	
IPI00023824;IPI00465038;IPI00789451;IPI00927720	FBLN2	CDNA FLJ56912, HIGHLY SIMILAR TO FIBULIN-2.;FIBULIN 2 ISOFORM A PRECURSOR.;PUTATIVE UNCHARACTERIZED PROTEIN DKFZP586A1519.;FIBULIN-2.			1				1				2	
IPI00023860;IPI00789029;IPI00797545;IPI00902909	NAP1L1	NUCLEOSOME ASSEMBLY PROTEIN 1-LIKE 1.;"CDNA FLJ30458 FIS, CLONE BRACE2009421, HIGHLY SIMILAR TO NUCLEOSOME ASSEMBLY PROTEIN 1-LIKE 1.";"CDNA FLJ60539, HIGHLY SIMILAR TO NUCLEOSOME ASSEMBLY PROTEIN 1-LIKE 1.";"NUCLEOSOME ASSEMBLY PROTEIN 1-LIKE 1, ISOFORM CRA_C."	**1.28**	0.00	1		**1.54**	0.00	2		**1.05**	0.00	2	
IPI00023919;IPI00555749;IPI00745502	PSMC5	26S PROTEASE REGULATORY SUBUNIT 8.;PROTEASOME 26S ATPASE SUBUNIT 5 VARIANT (FRAGMENT).;"PROTEASOME (PROSOME, MACROPAIN) 26S SUBUNIT, ATPASE, 5, ISOFORM CRA_B."			1				1				1	
IPI00024067;IPI00455383	CLTC	ISOFORM 1 OF CLATHRIN HEAVY CHAIN 1.;ISOFORM 2 OF CLATHRIN HEAVY CHAIN 1.	**1.01**	0.14	17	0.410	**1.17**	0.13	19	0.578	**1.02**	0.13	19	0.432
IPI00024095;IPI00745868	ANXA3	ANNEXIN A3.;PUTATIVE UNCHARACTERIZED PROTEIN ANXA3 (FRAGMENT).	**0.88**	0.05	5	0.469	**1.30**	0.09	5	0.311	**1.84**	0.38	7	0.563
IPI00024145;IPI00216026;IPI00455531;IPI00737171;IPI00902560;IPI00917420	VDAC2;VDAC2;LOC729317;LOC729317	ISOFORM 2 OF VOLTAGE-DEPENDENT ANION-SELECTIVE CHANNEL PROTEIN 2.;ISOFORM 3 OF VOLTAGE-DEPENDENT ANION-SELECTIVE CHANNEL PROTEIN 2.;PUTATIVE UNCHARACTERIZED PROTEIN ENSP00000367226.;SIMILAR TO VOLTAGE-DEPENDENT ANION CHANNEL 2.;ISOFORM 1 OF VOLTAGE-DEPENDENT ANION-SELECTIVE CHANNEL PROTEIN 2.;30 KDA PROTEIN.	**0.88**	0.05	3	0.231	**0.85**	0.10	3	0.618	**0.89**	0.02	3	
IPI00024284	HSPG2	BASEMENT MEMBRANE-SPECIFIC HEPARAN SULFATE PROTEOGLYCAN CORE PROTEIN.	**1.77**	0.34	26	0.000	**2.46**	0.32	31	0.001	**1.21**	0.16	25	0.185
IPI00024403	CPNE3	COPINE-3.			1				1				1	
IPI00024466;IPI00619903	UGGT1	ISOFORM 1 OF UDP-GLUCOSE:GLYCOPROTEIN GLUCOSYLTRANSFERASE 1.;ISOFORM 2 OF UDP-GLUCOSE:GLYCOPROTEIN GLUCOSYLTRANSFERASE 1.	**0.93**	0.00	3	0.285			3				3	
IPI00024580;IPI00789136;IPI00792499;IPI00945529	MCCC1	METHYLCROTONOYL-COA CARBOXYLASE SUBUNIT ALPHA, MITOCHONDRIAL.;"CDNA FLJ53328, HIGHLY SIMILAR TO METHYLCROTONOYL-COA CARBOXYLASE SUBUNIT ALPHA, MITOCHONDRIAL.";PUTATIVE UNCHARACTERIZED PROTEIN DKFZP686B20267.;"CDNA FLJ54709, HIGHLY SIMILAR TO METHYLCROTONOYL-COA CARBOXYLASE SUBUNIT ALPHA, MITOCHONDRIAL."	**0.98**	0.00	2				2				2	
IPI00024661	SEC24C	PROTEIN TRANSPORT PROTEIN SEC24C.			1		**2.52**	0.00	1				1	
IPI00024664;IPI00375145	USP5	ISOFORM LONG OF UBIQUITIN CARBOXYL-TERMINAL HYDROLASE 5.;ISOFORM SHORT OF UBIQUITIN CARBOXYL-TERMINAL HYDROLASE 5.			1								1	
IPI00024689;IPI00428490;IPI00909619;IPI00909780;IPI00915781;IPI00915988;IPI00917259	AQP1;FAM188B	AQUAPORIN-1.;AQUAPORIN 1 SPLICE VARIANT 2.;"CDNA FLJ50602, HIGHLY SIMILAR TO AQUAPORIN-1.";"CDNA FLJ53773, HIGHLY SIMILAR TO AQUAPORIN-1.";23 KDA PROTEIN.;"CDNA FLJ50708, HIGHLY SIMILAR TO AQUAPORIN-1.";"CDNA FLJ51453, HIGHLY SIMILAR TO AQUAPORIN-1."	**0.90**	0.00	1		**0.41**	0.00	1				1	
IPI00024896;IPI00396138;IPI00651719	PBLD	PHENAZINE BIOSYNTHESIS-LIKE DOMAIN-CONTAINING PROTEIN.;28 KDA PROTEIN.;MAWD BINDING PROTEIN ISOFORM B.	**0.79**	0.24	2				2				1	
IPI00024911	ERP29	ENDOPLASMIC RETICULUM PROTEIN ERP29.	**0.78**	0.00	1	0.180	**0.62**	0.03	3		**0.27**	0.00	1	
IPI00024915;IPI00759663	PRDX5	ISOFORM MITOCHONDRIAL OF PEROXIREDOXIN-5, MITOCHONDRIAL.;"ISOFORM CYTOPLASMIC+PEROXISOMAL OF PEROXIREDOXIN-5, MITOCHONDRIAL."	**1.10**	0.14	7	0.312	**0.83**	0.04	7	0.323	**0.82**	0.22	7	0.343
IPI00024919;IPI00374151	PRDX3	THIOREDOXIN-DEPENDENT PEROXIDE REDUCTASE, MITOCHONDRIAL.;PEROXIREDOXIN 3 ISOFORM B.	**0.90**	0.02	3	0.322	**0.66**	0.06	3	0.261	**0.40**	0.00	3	0.207
IPI00024933	RPL12	ISOFORM 1 OF 60S RIBOSOMAL PROTEIN L12.	**1.20**	0.16	3	0.477	**0.97**	0.06	3	0.529	**0.52**	0.03	3	
IPI00024990	ALDH6A1	METHYLMALONATE-SEMIALDEHYDE DEHYDROGENASE [ACYLATING], MITOCHONDRIAL.	**1.11**	0.11	3	0.366	**0.37**	0.03	3				3	
IPI00024993	ECHS1	ENOYL-COA HYDRATASE, MITOCHONDRIAL.	**1.09**	0.00	1		**1.55**	0.00	1		**0.74**	0.00	2	
IPI00025084	CAPNS1	CALPAIN SMALL SUBUNIT 1.			2				2				2	
IPI00025091;IPI00748962	RPS11	40S RIBOSOMAL PROTEIN S11.;SIMILAR TO 40S RIBOSOMAL PROTEIN S11.	**0.60**	0.00	1						**0.22**	0.00	1	
IPI00025252	PDIA3	PROTEIN DISULFIDE-ISOMERASE A3.	**0.98**	0.12	21	0.261	**0.76**	0.16	20	0.049	**0.45**	0.05	20	0.003
IPI00025273;IPI00384624	GART	ISOFORM LONG OF TRIFUNCTIONAL PURINE BIOSYNTHETIC PROTEIN ADENOSINE-3.;GARS-AIRS-GART (FRAGMENT).	**1.07**	0.00	1				1		**0.38**	0.00	1	
IPI00025277;IPI00930049;IPI00930510	PDCD6;AHRR	PROGRAMMED CELL DEATH PROTEIN 6.;"HCG1985580, ISOFORM CRA_F.";PROGRAMMED CELL DEATH 6 VARIANT (FRAGMENT).	**1.04**	0.00	1		**1.51**	0.00	1		**0.66**	0.00	1	
IPI00025318	SH3BGRL	SH3 DOMAIN-BINDING GLUTAMIC ACID-RICH-LIKE PROTEIN.	**1.09**	0.00	3		**1.89**	0.00	1		**1.67**	0.00	1	
IPI00025329	RPL19	60S RIBOSOMAL PROTEIN L19.	**1.27**	0.29	3	0.245	**1.18**	0.35	3	0.175	**0.84**	0.37	3	
IPI00025366;IPI00383539;IPI00791211;IPI00791780;IPI00792509;IPI00793839;IPI00795080;IPI00795682	CS	CITRATE SYNTHASE, MITOCHONDRIAL.;CITRATE SYNTHASE.;12 KDA PROTEIN.;16 KDA PROTEIN.;7 KDA PROTEIN.;48 KDA PROTEIN.;9 KDA PROTEIN.	**0.93**	0.00	1		**0.95**	0.00	1		**0.69**	0.00	1	
IPI00025427	RNASE3	EOSINOPHIL CATIONIC PROTEIN.			1		**0.99**	0.00	1		**1.29**	0.00	1	
IPI00025447;IPI00940393	EEF1A1	ELONGATION FACTOR 1-ALPHA.	**0.76**	0.12	12	0.028	**0.57**	0.10	12	0.040	**0.48**	0.07	12	0.054
IPI00025465	OGN	CDNA FLJ59205, HIGHLY SIMILAR TO MIMECAN.	**6.08**	0.30	7	0.051	**6.41**	0.29	7	0.086	**3.16**	0.18	8	0.171
IPI00025476	AMY2A;AMY1C;AMY1A;AMY1B	PANCREATIC ALPHA-AMYLASE.	**0.86**	0.15	32	0.204	**0.32**	0.12	33	0.000	**0.02**	0.00	32	0.067
IPI00025512	HSPB1	HEAT SHOCK PROTEIN BETA-1.	**1.35**	0.13	13	0.324	**1.39**	0.10	13	0.345	**0.84**	0.16	13	0.526

IPI00025796	NDUFS3	NADH DEHYDROGENASE [UBIQUINONE] IRON-SULFUR PROTEIN 3, MITOCHONDRIAL.			2				1				2	
IPI00025874	RPN1	DOLICHYL-DIPHOSPHOOLIGOSACCHARIDE--PROTEIN GLYCOSYLTRANSFERASE SUBUNIT 1 PRECURSOR.	**0.77**	0.07	16	0.017	**0.56**	0.09	16	0.010	**0.23**	0.03	17	0.040
IPI00026059;IPI00216858	SNTB1	ISOFORM 1 OF BETA-1-SYNTROPHIN.;ISOFORM 2 OF BETA-1-SYNTROPHIN.	**1.15**	0.00	1				1				1	
IPI00026087;IPI00186338	BANF1;LOC645870	BARRIER-TO-AUTOINTEGRATION FACTOR.;SIMILAR TO BARRIER TO AUTOINTEGRATION FACTOR 1.	**1.56**	0.00	1		**1.15**	0.00	1		**1.10**	0.00	1	
IPI00026154;IPI00792916;IPI00943074	PRKCSH	CDNA FLJ59211, HIGHLY SIMILAR TO GLUCOSIDASE 2 SUBUNIT BETA.;PROTEIN KINASE C SUBSTRATE 80K-H ISOFORM 2.;GLUCOSIDASE 2 SUBUNIT BETA.	**0.97**	0.11	3	0.328	**0.85**	0.12	3	0.323	**0.61**	0.12	3	0.353
IPI00026182;IPI00852796;IPI00853571;IPI00873113;IPI00952611	CAPZA2	F-ACTIN-CAPPING PROTEIN SUBUNIT ALPHA-2.;20 KDA PROTEIN.;"CDNA FLJ51827, HIGHLY SIMILAR TO F-ACTIN CAPPING PROTEIN ALPHA-2 SUBUNIT.";PUTATIVE UNCHARACTERIZED PROTEIN CAPZA2.	**1.19**	0.07	2	0.469	**1.01**	0.01	2	0.578	**1.16**	0.00	2	
IPI00026185;IPI00218782;IPI00642256;IPI00646047;IPI00647677;IPI00903103	CAPZB	ISOFORM 1 OF F-ACTIN-CAPPING PROTEIN SUBUNIT BETA.;"CDNA FLJ60094, HIGHLY SIMILAR TO F-ACTIN CAPPING PROTEIN SUBUNIT BETA.";ISOFORM 2 OF F-ACTIN-CAPPING PROTEIN SUBUNIT BETA.;"CDNA FLJ43095 FIS, CLONE CORDB2000541, HIGHLY SIMILAR TO F-ACTIN CAPPING PROTEIN SUBUNIT BETA.";"CAPPING PROTEIN (ACTIN FILAMENT) MUSCLE Z-LINE, BETA."											1	
IPI00026202;IPI00847774;IPI00872093;IPI00909746	RPL18A	60S RIBOSOMAL PROTEIN L18A.;"CDNA FLJ51501, HIGHLY SIMILAR TO 60S RIBOSOMAL PROTEIN L18A.";21 KDA PROTEIN.;"CDNA FLJ51502, HIGHLY SIMILAR TO 60S RIBOSOMAL PROTEIN L18A."	**0.87**	0.00	1	0.141	**0.64**	0.00	1	0.092	**0.53**	0.00	1	
IPI00026216	NPEPPS	PUROMYCIN-SENSITIVE AMINOPEPTIDASE.	**1.45**	0.14	2		**1.55**	0.00	2		**1.37**	0.21	2	
IPI00026260;IPI00029091;IPI00604590;IPI00790856;IPI00795292	NME1;NME2;NME1-NME2;NME2	ISOFORM 1 OF NUCLEOSIDE DIPHOSPHATE KINASE B.;PUTATIVE NUCLEOSIDE DIPHOSPHATE KINASE.;NUCLEOSIDE DIPHOSPHATE KINASE.;9 KDA PROTEIN.;ISOFORM 3 OF NUCLEOSIDE DIPHOSPHATE KINASE B.	**1.25**	0.00	1		**1.10**	0.00	1		**0.84**	0.00	1	
IPI00026271	RPS14	40S RIBOSOMAL PROTEIN S14.	**0.72**	0.06	4	0.076	**0.27**	0.01	4	0.055	**0.20**	0.01	4	0.087
IPI00026272;IPI00031562;IPI00081836;IPI00216456;IPI00220855;IPI00255316;IPI00291764;IPI00552873;IPI00930144	HIST1H2AD;HIST1H2AI;HIST1H2AG;HIST1H2AM;HIST1H2AJ;HIST1H2AE;HIST1H2AB;HIST1H2AK;HIST1H2AL;HIST3H2A;HIST1H2AI;HIST1H2AG;HIST1H2AM;HIST1H2AH;HIST1H2AJ;HIST1H2AK;HIST1H2AL;HIST1H2AC;H2AFJ;HIST1H2AD;HIST1H2AI;HIST1H2AG;HIST1H2AM;HIST1H2AJ;HIST1H2AK;HIST1H2AL	HISTONE H2A TYPE 1-B/E.;HISTONE H2A TYPE 3.;HISTONE H2A TYPE 1-H.;HISTONE H2A TYPE 1-C.;ISOFORM 1 OF HISTONE H2A.J.;HISTONE H2A TYPE 1-D.;HISTONE H2A TYPE 1.;HISTONE H2A TYPE 1-J.;HISTONE H2A.	**1.26**	0.08	7									
IPI00026302;IPI00848331;IPI00856058;IPI00915795;IPI00916315;IPI00916572;IPI00917298	RPL31	60S RIBOSOMAL PROTEIN L31.;RIBOSOMAL PROTEIN L31 ISOFORM 2.;RIBOSOMAL PROTEIN L31 ISOFORM 3.;13 KDA PROTEIN.;PUTATIVE UNCHARACTERIZED PROTEIN RPL31.;PROTEIN.	**0.80**	0.03	3	0.177	**0.80**	0.00	3				3	
IPI00026314;IPI00646773;IPI00647556;IPI00796316	GSN	ISOFORM 1 OF GELSOLIN.;ISOFORM 2 OF GELSOLIN.;GELSOLIN ISOFORM C.;GELSOLIN.	**2.05**	0.09	8	0.051	**3.10**	0.41	9	0.022	**2.33**	0.58	7	0.015
IPI00026530	LMAN1	PROTEIN ERGIC-53.	**0.93**	0.10	5	0.227	**0.47**	0.03	5		**0.26**	0.00	5	
IPI00026570;IPI00872879	COX7A2	CYTOCHROME C OXIDASE POLYPEPTIDE 7A2, MITOCHONDRIAL.;PUTATIVE UNCHARACTERIZED PROTEIN COX7A2.	**0.96**	0.00	1	0.216	**1.07**	0.00	1		**0.46**	0.00	1	
IPI00026665;IPI00796022;IPI00925046;IPI00925203;IPI00925511;IPI00925952	QARS	CDNA FLJ75085, HIGHLY SIMILAR TO HOMO SAPIENS GLUTAMINYL-TRNA SYNTHETASE (QARS), MRNA.;"CDNA FLJ54487, HIGHLY SIMILAR TO GLUTAMINYL-TRNA SYNTHETASE.";GLUTAMINYL-TRNA SYNTHETASE.;29 KDA PROTEIN.;"CDNA FLJ54314, HIGHLY SIMILAR TO GLUTAMINYL-TRNA SYNTHETASE.";"CDNA FLJ54453, HIGHLY SIMILAR TO GLUTAMINYL-TRNA SYNTHETASE."	**1.19**	0.00	1		**0.21**	0.00	1				1	
IPI00026673;IPI00071696;IPI00419894;IPI00472000;IPI00472247;IPI00736943;IPI00741668;IPI00742111;IPI00873332;IPI00927318	CUX1	ISOFORM 1 OF HOMEOBOX PROTEIN CUT-LIKE 1.;PROTEIN CASP.;ISOFORM 3 OF HOMEOBOX PROTEIN CUT-LIKE 1.;163 KDA PROTEIN.;ISOFORM 2 OF HOMEOBOX PROTEIN CUT-LIKE 1.;ISOFORM 5 OF HOMEOBOX PROTEIN CUT-LIKE 1.;ISOFORM 7 OF HOMEOBOX PROTEIN CUT-LIKE 1.;ISOFORM 6 OF HOMEOBOX PROTEIN CUT-LIKE 1.;159 KDA PROTEIN.;"CDNA FLJ43107 FIS, CLONE CTONG2020108, HIGHLY SIMILAR TO PROTEIN CASP."											1	
IPI00026781	FASN	FATTY ACID SYNTHASE.	**0.78**	0.00	2		**0.83**	0.06	3		**0.67**	0.02	2	
IPI00026944;IPI00384542	NID1	ISOFORM 1 OF NIDOGEN-1.;ISOFORM 2 OF NIDOGEN-1.	**1.45**	0.00	3		**1.50**	0.00	3		**0.85**	0.00	3	
IPI00027009;IPI00221111	PACSIN2	ISOFORM 1 OF PROTEIN KINASE C AND CASEIN KINASE SUBSTRATE IN NEURONS PROTEIN 2.;ISOFORM 2 OF PROTEIN KINASE C AND CASEIN KINASE SUBSTRATE IN NEURONS PROTEIN 2.	**1.04**	0.00	1		**0.85**	0.00	1				1	
IPI00027032;IPI00514267;IPI00514798;IPI00644956;IPI00876921	LYPLA2	ACYL-PROTEIN THIOESTERASE 2.;LYSOPHOSPHOLIPASE II.	**1.23**	0.00	2		**0.90**	0.00	1		**0.88**	0.00	1	
IPI00027107	TUFM	TU TRANSLATION ELONGATION FACTOR, MITOCHONDRIAL PRECURSOR.	**1.09**	0.07	8	0.374	**0.70**	0.15	8	0.368	**0.48**	0.09	8	0.187
IPI00027223	IDH1	ISOCITRATE DEHYDROGENASE [NADP] CYTOPLASMIC.	**0.93**	0.09	6	0.180	**0.79**	0.15	6	0.087	**0.70**	0.22	6	0.117
IPI00027230	HSP90B1	ENDOPLASMIN.	**0.87**	0.12	34	0.038	**0.51**	0.12	34	0.001	**0.26**	0.04	34	0.000
IPI00027252	PHB2	PROHIBITIN-2.	**1.11**	0.06	5	0.439	**0.70**	0.09	4	0.411	**0.53**	0.07	5	0.305
IPI00027341;IPI00848090;IPI00908511;IPI00916783	CAPG	MACROPHAGE-CAPPING PROTEIN.;GELSOLIN-LIKE CAPPING PROTEIN.;"CDNA FLJ51488, HIGHLY SIMILAR TO MACROPHAGE CAPPING PROTEIN.";PUTATIVE UNCHARACTERIZED PROTEIN CAPG.	**2.35**	0.00	2		**1.95**	0.40	2		**2.92**	1.38	2	
IPI00027350;IPI00909207	PRDX2	PEROXIREDOXIN-2.;"CDNA FLJ60461, HIGHLY SIMILAR TO PEROXIREDOXIN-2."	**1.35**	0.07	8	0.051	**1.46**	0.13	8	0.323	**0.80**	0.20	8	0.490
IPI00027409	PRTN3	MYELOBLASTIN.			1		**1.65**	0.00	1		**1.64**	0.00	1	
IPI00027412	CEACAM6	CARCINOEMBRYONIC ANTIGEN-RELATED CELL ADHESION MOLECULE 6.											5	
IPI00027442;IPI00910701	AARS	ALANYL-TRNA SYNTHETASE, CYTOPLASMIC.;"CDNA FLJ61339, HIGHLY SIMILAR TO ALANYL-TRNA SYNTHETASE."	**0.79**	0.00	3		**0.80**	0.06	3				2	
IPI00027443;IPI00556365;IPI00556541;IPI00909763;IPI00939491	CARS	CYSTEINYL-TRNA SYNTHETASE ISOFORM C.;"ISOFORM 1 OF CYSTEINYL-TRNA SYNTHETASE, CYTOPLASMIC.";CYSTEINYL-TRNA SYNTHETASE ISOFORM D.;"CDNA FLJ59563, HIGHLY SIMILAR TO CYSTEINYL-TRNA SYNTHETASE.";"ISOFORM 2 OF CYSTEINYL-TRNA SYNTHETASE, CYTOPLASMIC."	**0.81**	0.03	3		**0.95**	0.00	3		**1.04**	0.03	3	
IPI00027444	SERPINB1	LEUKOCYTE ELASTASE INHIBITOR.	**0.82**	0.05	9	0.063	**0.67**	0.08	9	0.058	**0.62**	0.09	9	0.171
IPI00027462;IPI00939362	S100A9	PROTEIN S100-A9.;9 KDA PROTEIN.	**1.07**	0.28	4	0.336	**3.34**	0.43	4	0.319	**2.80**	0.43	5	0.452
IPI00027463	S100A6	PROTEIN S100-A6.	**3.32**	0.00	2		**5.60**	0.00	1		**8.34**	0.00	2	
IPI00027486;IPI00743702;IPI00880101	CEACAM5	CARCINOEMBRYONIC ANTIGEN-RELATED CELL ADHESION MOLECULE 5.;PUTATIVE UNCHARACTERIZED PROTEIN DKFZP781M2392.;PROTEIN.											1	
IPI00027493;IPI00554481;IPI00554611;IPI00554702;IPI00554722;IPI00604710	SLC3A2;LOC442497	ISOFORM 2 OF 4F2 CELL-SURFACE ANTIGEN HEAVY CHAIN.;ISOFORM 4 OF 4F2 CELL-SURFACE ANTIGEN HEAVY CHAIN.;"SOLUTE CARRIER FAMILY 3, MEMBER 2 ISOFORM B.";"SOLUTE CARRIER FAMILY 3, MEMBER 2 ISOFORM D.";ISOFORM 3 OF 4F2 CELL-SURFACE ANTIGEN HEAVY CHAIN.;ISOFORM 1 OF 4F2 CELL-SURFACE ANTIGEN HEAVY CHAIN.											1	
IPI00027497;IPI00908881;IPI00910781	GPI	GLUCOSE-6-PHOSPHATE ISOMERASE.	**1.57**	0.22	7	0.246	**2.08**	0.33	7	0.204	**1.51**	0.28	7	
IPI00027509	MMP9	MATRIX METALLOPROTEINASE-9.							1					
IPI00027701;IPI00909944	ACADS	SHORT-CHAIN SPECIFIC ACYL-COA DEHYDROGENASE, MITOCHONDRIAL.;"CDNA FLJ51323, HIGHLY SIMILAR TO SHORT-CHAIN SPECIFIC ACYL-COA DEHYDROGENASE, MITOCHONDRIAL."			1				1				1	
IPI00027720	PNLIP	PANCREATIC TRIACYLGLYCEROL LIPASE.	**0.71**	0.14	25	0.028	**0.35**	0.12	24	0.066	**0.02**	0.00	23	0.176
IPI00027722;IPI00829925	CELA2A	CHYMOTRYPSIN-LIKE ELASTASE FAMILY MEMBER 2A.;PUTATIVE UNCHARACTERIZED PROTEIN ENSP00000365090.	**0.44**	0.11	9	0.038	**0.33**	0.13	9	0.081	**0.16**	0.11	9	0.353
IPI00027723;IPI00940831	CELA2B	CHYMOTRYPSIN-LIKE ELASTASE FAMILY MEMBER 2B.;33 KDA PROTEIN.	**0.74**	0.12	5	0.214	**0.61**	0.18	5	0.298	**0.16**	0.11	5	0.353
IPI00027827	SOD3	EXTRACELLULAR SUPEROXIDE DISMUTASE [CU-ZN].	**1.21**	0.00	1	0.369	**1.78**	0.00	1	0.260	**1.50**	0.00	2	0.611
IPI00027933	PSMB10	PROTEASOME SUBUNIT BETA TYPE-10.	**0.79**	0.00	1		**1.19**	0.00	1		**0.87**	0.00	1	
IPI00027947;IPI00643847	CTRL	SIMILAR TO CHYMOTRYPSIN-LIKE PROTEASE CTRL-1 PRECURSOR.;CHYMOTRYPSIN-LIKE PROTEASE CTRL-1.	**0.80**	0.10	3	0.366	**0.44**	0.01	2	0.202			2	
IPI00028004	PSMB3	PROTEASOME SUBUNIT BETA TYPE-3.	**0.91**	0.01	3		**0.86**	0.00	3	0.493	**0.56**	0.02	3	
IPI00028031;IPI00937735	ACADVL	CDNA FLJ56425, HIGHLY SIMILAR TO VERY-LONG-CHAIN SPECIFIC ACYL- COADEHYDROGENASE, MITOCHONDRIAL.;"CDNA FLJ55801, HIGHLY SIMILAR TO VERY-LONG-CHAIN SPECIFIC ACYL-COADEHYDROGENASE, MITOCHONDRIAL."	**1.05**	0.12	4	0.383	**0.85**	0.06	4	0.287	**0.42**	0.04	4	0.065
IPI00028055;IPI00911085	TMED10	TRANSMEMBRANE EMP24 DOMAIN-CONTAINING PROTEIN 10.;"CDNA FLJ53754, HIGHLY SIMILAR TO TRANSMEMBRANE EMP24 DOMAIN-CONTAINING PROTEIN 10."	**0.68**	0.00	2				2		**0.24**	0.00	2	
IPI00028064	CTSG	CATHEPSIN G.	**1.85**	0.00	1				1		**2.35**	0.00	2	
IPI00028160;IPI00219877	HMBS	ISOFORM 1 OF PORPHOBILINOGEN DEAMINASE.;ISOFORM 2 OF PORPHOBILINOGEN DEAMINASE.	**1.49**	0.00	1		**1.20**	0.00	1				1	

IPI00028277;IPI00641635;IPI00845477;IPI00845501;IPI00939501;IPI00945698	FTO	ISOFORM 1 OF PROTEIN FTO.;64 KDA PROTEIN.;ISOFORM 2 OF PROTEIN FTO.;ISOFORM 4 OF PROTEIN FTO.;11 KDA PROTEIN.	**0.91**	0.00	2		**0.97**	0.00	2				1	
IPI00028387;IPI00942981	DDRGK1	ISOFORM 1 OF DDRGK DOMAIN-CONTAINING PROTEIN 1.;36 KDA PROTEIN.	**0.85**	0.06	6	0.285	**0.60**	0.05	6	0.235	**0.15**	0.00	6	
IPI00028444	PLIN1	PERILIPIN-1.	**1.10**	0.00	1		**1.09**	0.00	1				1	
IPI00028481	RAB8A	RAS-RELATED PROTEIN RAB-8A.			1		**0.94**	0.00	1				1	
IPI00028520;IPI00221298;IPI00910368	NDUFV1	ISOFORM 1 OF NADH DEHYDROGENASE [UBIQUINONE] FLAVOPROTEIN 1, MITOCHONDRIAL.;"ISOFORM 2 OF NADH DEHYDROGENASE [UBIQUINONE] FLAVOPROTEIN 1, MITOCHONDRIAL.";"CDNA FLJ57201, HIGHLY SIMILAR TO NADH-UBIQUINONE OXIDOREDUCTASE 51 KDA SUBUNIT, MITOCHONDRIAL."	**0.79**	0.05	2	0.285			2				1	
IPI00028635;IPI00383680;IPI00552972	RPN2	DOLICHYL-DIPHOSPHOOLIGOSACCHARIDE--PROTEIN GLYCOSYLTRANSFERASE SUBUNIT 2.;RIBOPHORIN II ISOFORM 2 PRECURSOR.;PUTATIVE UNCHARACTERIZED PROTEIN RPN2.	**0.75**	0.12	11		**0.53**	0.10	11		**0.21**	0.03	11	
IPI00028786;IPI00409566;IPI00409567;IPI00883708;IPI00939688;IPI00939907;IPI00942389	PKD1	ISOFORM 3 OF POLYCYSTIN-1.;ISOFORM 1 OF POLYCYSTIN-1.;ISOFORM 2 OF POLYCYSTIN-1.;392 KDA PROTEIN.;462 KDA PROTEIN.;463 KDA PROTEIN.;461 KDA PROTEIN.	**1.36**	0.13	2		**2.14**	0.23	2		**1.31**	0.19	2	
IPI00028888;IPI00220683;IPI00220684;IPI00220685;IPI00903278;IPI00915340	HNRNPD	ISOFORM 1 OF HETEROGENEOUS NUCLEAR RIBONUCLEOPROTEIN D0.;ISOFORM 2 OF HETEROGENEOUS NUCLEAR RIBONUCLEOPROTEIN D0.;ISOFORM 3 OF HETEROGENEOUS NUCLEAR RIBONUCLEOPROTEIN D0.;ISOFORM 4 OF HETEROGENEOUS NUCLEAR RIBONUCLEOPROTEIN D0.;P37 AUF1.;PUTATIVE UNCHARACTERIZED PROTEIN HNRNPD.	**1.03**	0.12	3		**1.23**	0.01	3	0.578	**0.77**	0.07	3	0.508
IPI00028908;IPI00293033	NID2	NIDOGEN-2.;NID2 PROTEIN.	**1.85**	0.00	1		**2.17**	0.00	1		**1.66**	0.00	1	
IPI00028931	DSG2	DESMOGLEIN-2.											2	
IPI00029012	EIF3A	EUKARYOTIC TRANSLATION INITIATION FACTOR 3 SUBUNIT A.			1		**0.74**	0.00	1				1	
IPI00029039	REG3A	REGENERATING ISLET-DERIVED PROTEIN 3 ALPHA.			1				1				1	
IPI00029046;IPI00798088	MLEC	MALECTIN.;16 KDA PROTEIN.	**0.67**	0.00	1		**0.39**	0.00	1				1	
IPI00029111;IPI00872788	DPYSL3	COLLAPSIN RESPONSE MEDIATOR PROTEIN 4 LONG VARIANT.;DIHYDROPYRIMIDINASE-RELATED PROTEIN 3.	**1.31**	0.00	2		**3.86**	0.00	3		**2.82**	0.00	2	
IPI00029133;IPI00456747;IPI00880198	ATP5F1	ATP SYNTHASE SUBUNIT B, MITOCHONDRIAL.;"ATP SYNTHASE, H+ TRANSPORTING, MITOCHONDRIAL F0 COMPLEX, SUBUNIT B1.";12 KDA PROTEIN.	**1.18**	0.00	1				1				1	
IPI00029260	CD14	MONOCYTE DIFFERENTIATION ANTIGEN CD14.			1				2					
IPI00029264	CYC1	CYTOCHROME C1, HEME PROTEIN, MITOCHONDRIAL.	**0.78**	0.00	2		**0.56**	0.00	2		**0.31**	0.00	2	
IPI00029403;IPI00790243;IPI00946080	SNX4	SORTING NEXIN-4.;20 KDA PROTEIN.;PROTEIN.	**1.42**	0.00	1		**1.75**	0.00	1		**0.78**	0.00	1	
IPI00029468;IPI00908883	ACTR1A	ALPHA-CENTRACTIN.;"CDNA FLJ55002, HIGHLY SIMILAR TO ALPHA-CENTRACTIN."	**1.22**	0.20	2	0.449	**1.11**	0.13	2	0.621	**0.67**	0.18	4	
IPI00029469	ACTR1B	BETA-CENTRACTIN.	**0.90**	0.00	1		**0.73**	0.00	1				1	
IPI00029485;IPI00219114;IPI00555695;IPI00872359;IPI00873712;IPI00914026;IPI00916757;IPI00917166;IPI00917681;IPI00935906	DCTN1	ISOFORM P150 OF DYNACTIN SUBUNIT 1.;ISOFORM P135 OF DYNACTIN SUBUNIT 1.;DYNACTIN 1 ISOFORM 1 VARIANT (FRAGMENT).;PUTATIVE UNCHARACTERIZED PROTEIN DCTN1.;DYNACTIN 1 ISOFORM 4.;137 KDA PROTEIN.;PUTATIVE UNCHARACTERIZED PROTEIN DKFZP686E0752.;139 KDA PROTEIN.;DYNACTIN 1 ISOFORM 3.	**1.17**	0.00	1		**1.77**	0.00	1				1	
IPI00029561;IPI00103509;IPI00894154	NDUFA10	NADH DEHYDROGENASE [UBIQUINONE] 1 ALPHA SUBCOMPLEX SUBUNIT 10, MITOCHONDRIAL.;NADH DEHYDROGENASE UBIQUINONE 1 ALPHA SUBCOMPLEX.;45 KDA PROTEIN.	**1.02**	0.00	2		**0.90**	0.00	2		**0.31**	0.00	2	
IPI00029601;IPI00062884;IPI00792087	CTTN	SRC SUBSTRATE CORTACTIN.;CORTACTIN ISOFORM B.;PUTATIVE UNCHARACTERIZED PROTEIN CTTN.											1	
IPI00029623	PSMA6	PROTEASOME SUBUNIT ALPHA TYPE-6.	**1.19**	0.00	1		**1.39**	0.00	1		**1.11**	0.00	1	
IPI00029658;IPI00220813;IPI00220814;IPI00220815;IPI00893517;IPI00908496;IPI00922935	EFEMP1	ISOFORM 1 OF EGF-CONTAINING FIBULIN-LIKE EXTRACELLULAR MATRIX PROTEIN 1.;ISOFORM 2 OF EGF-CONTAINING FIBULIN-LIKE EXTRACELLULAR MATRIX PROTEIN 1.;ISOFORM 3 OF EGF-CONTAINING FIBULIN-LIKE EXTRACELLULAR MATRIX PROTEIN 1.;ISOFORM 4 OF EGF-CONTAINING FIBULIN-LIKE EXTRACELLULAR MATRIX PROTEIN 1.;PUTATIVE UNCHARACTERIZED PROTEIN EFEMP1.;"CDNA FLJ53670, HIGHLY SIMILAR TO EGF-CONTAINING FIBULIN-LIKE EXTRACELLULARMATRIX PROTEIN 1.";"CDNA FLJ35535 FIS, CLONE SPLEN2002419, HIGHLY SIMILAR TO EGF- CONTAINING FIBULIN-LIKE EXTRACELLULAR MATRIX PROTEIN 1."			1				1				2	
IPI00029715	AOX1	ALDEHYDE OXIDASE.			1				1				1	
IPI00029750;IPI00847986;IPI00903204;IPI00915363;IPI00915463;IPI00942970;IPI00943336	RPS24	ISOFORM 1 OF 40S RIBOSOMAL PROTEIN S24.;ISOFORM 2 OF 40S RIBOSOMAL PROTEIN S24.;15 KDA PROTEIN.;RIBOSOMAL PROTEIN S24 ISOFORM D.;RIBOSOMAL PROTEIN S24 ISOFORM E.;16 KDA PROTEIN.;RIBOSOMAL PROTEIN S24 ISOFORM F.	**0.68**	0.00	2		**0.39**	0.00	2		**0.27**	0.00	2	
IPI00029928;IPI00163202;IPI00177942;IPI00301099;IPI00472548;IPI00513726;IPI00514114;IPI00514196;IPI00514508;IPI00515021;IPI00829923;IPI00902812;IPI00902994;IPI00925620;IPI00926323;IPI00926593;IPI00926794;IPI00926971;IPI00954283	ELN	ELASTIN.;ISOFORM 10 OF ELASTIN.;ELASTIN ISOFORM C PRECURSOR.;"CDNA PSEC0254 FIS, CLONE NT2RP3003474, MODERATELY SIMILAR TO ELASTIN.";ISOFORM 1 OF ELASTIN.;ISOFORM 9 OF ELASTIN.;ISOFORM 3 OF ELASTIN.;ISOFORM 8 OF ELASTIN.;ISOFORM 5 OF ELASTIN.;ISOFORM 4 OF ELASTIN.;ELASTIN ISOFORM E PRECURSOR.;ELASTIN ISOFORM B PRECURSOR.;"CDNA FLJ36819 FIS, CLONE ASTRO2005575, HIGHLY SIMILAR TO ELASTIN.";ISOFORM 2 OF ELASTIN.;61 KDA PROTEIN.;"CDNA FLJ43523 FIS, CLONE PLACE5000282, WEAKLY SIMILAR TO HOMO SAPIENS ELASTIN.";"CDNA FLJ56005, HIGHLY SIMILAR TO ELASTIN.";ELASTIN ISOFORM A PRECURSOR.			1				1				1	
IPI00029997;IPI00643842	PGLS	6-PHOSPHOGLUCONOLACTONASE.;20 KDA PROTEIN.	**1.02**	0.00	1		**1.21**	0.00	1		**0.88**	0.00	1	
IPI00030131;IPI00181409;IPI00216230;IPI00791301;IPI00830089;IPI00873716	TMPO	ISOFORM BETA OF LAMINA-ASSOCIATED POLYPEPTIDE 2, ISOFORMS BETA/GAMMA.;"ISOFORM GAMMA OF LAMINA-ASSOCIATED POLYPEPTIDE 2, ISOFORMS BETA/GAMMA.";"LAMINA-ASSOCIATED POLYPEPTIDE 2, ISOFORM ALPHA.";46 KDA PROTEIN.;THYMOPOIETIN ZETA ISOFORM.;"LAMINA-ASSOCIATED POLYPEPTIDE 2, ISOFORMS BETA/GAMMA VARIANT (FRAGMENT)."	**1.84**	0.00	1				1				1	
IPI00030179;IPI00472171;IPI00794746;IPI00871827;IPI00872387;IPI00953028	RPL7P32;RPL7	60S RIBOSOMAL PROTEIN L7.;PUTATIVE UNCHARACTERIZED PROTEIN ENSP00000260536.;PUTATIVE UNCHARACTERIZED PROTEIN RPL7.;PUTATIVE UNCHARACTERIZED PROTEIN RPL7 (FRAGMENT).;PUTATIVE UNCHARACTERIZED PROTEIN RPL7P23 (FRAGMENT).	**0.79**	0.06	9		**0.71**	0.17	9		**0.25**	0.05	9	0.060
IPI00030205;IPI00384576;IPI00385252;IPI00387115;IPI00387117;IPI00387118;IPI00827510;IPI00827560;IPI00827637;IPI00854644;IPI00916434;IPI00945366;IPI00954551	IGKV3-20;IGKV3D-20;LOC100291682;LOC100290992	IG KAPPA CHAIN V-III REGION HAH.;IG KAPPA CHAIN V-III REGION HIC.;IG KAPPA CHAIN V-III REGION GOL.;IG KAPPA CHAIN V-III REGION SIE.;IG KAPPA CHAIN V-III REGION TI.;IG KAPPA CHAIN V-III REGION WOL.;HRV FAB 026-VL (FRAGMENT).;HRV FAB N27-VL (FRAGMENT).;K LIGHT CHAIN VARIABLE REGION (FRAGMENT).;SIMILAR TO HCG1686089.;ANTI-(ED-B) SCFV (FRAGMENT).;MYOSIN-REACTIVE IMMUNOGLOBULIN LIGHT CHAIN VARIABLE REGION (FRAGMENT).	**3.06**	0.00	1									
IPI00030275;IPI00646055	TRAP1	HEAT SHOCK PROTEIN 75 KDA, MITOCHONDRIAL.;57 KDA PROTEIN.	**0.94**	0.00	1				1		**0.70**	0.00	1	
IPI00030363	ACAT1	ACETYL-COA ACETYLTRANSFERASE, MITOCHONDRIAL.	**0.84**	0.10	9	0.094	**0.53**	0.08	8	0.299	**0.21**	0.05	8	
IPI00030702;IPI00792971;IPI00909577;IPI00921820	IDH3A	ISOFORM 1 OF ISOCITRATE DEHYDROGENASE [NAD] SUBUNIT ALPHA, MITOCHONDRIAL.;"ISOFORM 2 OF ISOCITRATE DEHYDROGENASE [NAD] SUBUNIT ALPHA, MITOCHONDRIAL.";"CDNA FLJ52894, HIGHLY SIMILAR TO ISOCITRATE DEHYDROGENASE.";"CDNA, FLJ78950, HIGHLY SIMILAR TO ISOCITRATE DEHYDROGENASE."			1									
IPI00030706	AHSA1	ACTIVATOR OF 90 KDA HEAT SHOCK PROTEIN ATPASE HOMOLOG 1.	**0.28**	0.00	2		**0.32**	0.00	2		**0.26**	0.00	2	
IPI00030730;IPI00446834;IPI00872071;IPI00896393	SULT1A4;SULT1A3	PHENOL SULFOTRANSFERASE 1A5*1A POSSIBLE ALTERNATIVE SPLICING FORM.;ISOFORM 2 OF SULFOTRANSFERASE 1A3/1A4.;ISOFORM 1 OF SULFOTRANSFERASE 1A3/1A4.;PUTATIVE UNCHARACTERIZED PROTEIN SULT1A4.			1				1				2	
IPI00030781;IPI00218188;IPI00795482	STAT1	ISOFORM ALPHA OF SIGNAL TRANSDUCER AND ACTIVATOR OF TRANSCRIPTION 1-ALPHA/BETA.;ISOFORM BETA OF SIGNAL TRANSDUCER AND ACTIVATOR OF TRANSCRIPTION 1-ALPHA/BETA.;83 KDA PROTEIN.											1	
IPI00030929	MYL9	MYOSIN REGULATORY LIGHT CHAIN 9 ISOFORM B.											2	
IPI00031008;IPI00220211;IPI00220212;IPI00220213;IPI00220214;IPI00220216;IPI00867560	TNC	ISOFORM 1 OF TENASCIN.;ISOFORM 2 OF TENASCIN.;ISOFORM 3 OF TENASCIN.;ISOFORM 4 OF TENASCIN.;ISOFORM 5 OF TENASCIN.;ISOFORM 6 OF TENASCIN.;TNC PROTEIN.											5	
IPI00031107;IPI00414384;IPI00936508	HSDL2	ISOFORM 2 OF HYDROXYSTEROID DEHYDROGENASE-LIKE PROTEIN 2.;ISOFORM 1 OF HYDROXYSTEROID DEHYDROGENASE-LIKE PROTEIN 2.;"CDNA FLJ61200, HIGHLY SIMILAR TO HOMO SAPIENS HYDROXYSTEROID DEHYDROGENASE LIKE 2 (HSDL2), MRNA."	**2.75**	0.00	1		**2.90**	0.00	2				2	
IPI00031121	CPE	CARBOXYPEPTIDASE E PRECURSOR.	**3.85**	0.00	2		**3.01**	0.00	2				1	
IPI00031169;IPI00790570;IPI00794561;IPI00873632	RAB2A	RAS-RELATED PROTEIN RAB-2A.;PROTEIN.;PUTATIVE UNCHARACTERIZED PROTEIN RAB2A.;24 KDA PROTEIN.	**0.96**	0.00	1									
IPI00031420;IPI00954552	UGDH	UDP-GLUCOSE 6-DEHYDROGENASE.	**1.45**	0.23	4		**1.11**	0.04	4		**0.59**	0.00	4	

IPI00031461;IPI00640006;IPI00940148	GDI2	CDNA FLJ60299, HIGHLY SIMILAR TO RAB GDP DISSOCIATION INHIBITOR BETA.;GDP DISSOCIATION INHIBITOR 2 ISOFORM 2.;RAB GDP DISSOCIATION INHIBITOR BETA.	**1.17**	0.09	9	0.339	**1.38**	0.18	8	0.463	**0.82**	0.17	8	0.238
IPI00031522	HADHA	TRIFUNCTIONAL ENZYME SUBUNIT ALPHA, MITOCHONDRIAL.	**1.17**	0.16	9	0.439	**1.01**	0.10	9	0.610	**0.67**	0.11	9	0.444
IPI00031556;IPI00830039	U2AF2	ISOFORM 1 OF SPLICING FACTOR U2AF 65 KDA SUBUNIT.;ISOFORM 2 OF SPLICING FACTOR U2AF 65 KDA SUBUNIT.	**1.18**	0.00	1				1		**1.29**	0.00	1	
IPI00031583;IPI00941161	USO1	PUTATIVE UNCHARACTERIZED PROTEIN DKFZP451D234.;GENERAL VESICULAR TRANSPORT FACTOR P115.	**1.42**	0.00	2				2				2	
IPI00031691	RPL9	60S RIBOSOMAL PROTEIN L9.	**0.72**	0.13	4	0.303	**0.30**	0.00	5		**0.22**	0.00	4	
IPI00031697;IPI00908304	TMEM109	TRANSMEMBRANE PROTEIN 109.;"CDNA FLJ57860, HIGHLY SIMILAR TO TRANSMEMBRANE PROTEIN 109."	**1.32**	0.00	3		**1.26**	0.00	3		**0.64**	0.00	3	
IPI00031812;IPI00450235;IPI00643351	YBX1	NUCLEASE-SENSITIVE ELEMENT-BINDING PROTEIN 1.;NUCLEASE SENSITIVE ELEMENT BINDING PROTEIN-1.;PROTEIN.									**0.51**	0.00	2	
IPI00031820;IPI00909657	FARSA	PHENYLALANYL-TRNA SYNTHETASE ALPHA CHAIN.;"CDNA FLJ50378, HIGHLY SIMILAR TO PHENYLALANYL-TRNA SYNTHETASE ALPHA CHAIN."	**0.73**	0.02	2		**0.48**	0.06	2		**0.23**	0.00	2	
IPI00032003;IPI00552214	EMD	EMERIN.;"EMERIN (EMERY-DREIFUSS MUSCULAR DYSTROPHY), ISOFORM CRA_B."	**1.34**	0.00	1				1		**2.12**	0.00	1	
IPI00032103;IPI00216279;IPI00792191;IPI00953505	GATM	ISOFORM MITOCHONDRIAL OF GLYCINE AMIDINOTRANSFERASE, MITOCHONDRIAL.;"ISOFORM CYTOPLASMIC OF GLYCINE AMIDINOTRANSFERASE, MITOCHONDRIAL.";"CDNA FLJ56372, HIGHLY SIMILAR TO GLYCINE AMIDINOTRANSFERASE, MITOCHONDRIAL."	**0.91**	0.12	13	0.219	**0.55**	0.18	13	0.082	**0.17**	0.07	13	
IPI00032140	SERPINH1	SERPIN H1.	**2.09**	0.33	7	0.172	**3.40**	0.76	7		**4.53**	1.13	8	0.150
IPI00032179;IPI00844156	SERPINC1	ANTITHROMBIN-III.;SERPINC1 PROTEIN.	**0.76**	0.00	1		**2.30**	0.00	1		**2.00**	0.00	2	
IPI00032220	AGT	ANGIOTENSINOGEN.	**1.74**	0.15	2		**1.20**	0.08	2	0.470	**1.36**	0.18	2	0.530
IPI00032258;IPI00643525;IPI00843913;IPI00889723;IPI00892547;IPI00937598	C4A;LOC100292046;LOC100294156	COMPLEMENT C4-A.;PUTATIVE UNCHARACTERIZED PROTEIN C4A.;COMPLEMENT COMPONENT 4A.;C4A PROTEIN.;SIMILAR TO C4A PROTEIN ISOFORM 2.	**1.54**	0.44	7	0.452	**3.36**	0.88	12	0.132	**3.40**	0.43	12	0.150
IPI00032311	LBP	LIPOPOLYSACCHARIDE-BINDING PROTEIN.							1				2	
IPI00032328;IPI00215894;IPI00789376;IPI00924859	KNG1	ISOFORM HMW OF KININOGEN-1.;ISOFORM LMW OF KININOGEN-1.;KNG1 PROTEIN.;44 KDA PROTEIN.	**1.12**	0.00	1		**1.86**	0.00	1		**1.84**	0.00	1	
IPI00032355;IPI00551014;IPI00646054;IPI00909786;IPI00911111;IPI00941189;IPI00941275	PUM1	PUMILIO HOMOLOG 1 (DROSOPHILA), ISOFORM CRA_C.;ISOFORM 1 OF PUMILIO HOMOLOG 1.;"PUMILIO HOMOLOG 1 (DROSOPHILA), ISOFORM CRA_B.";"CDNA FLJ56067, HIGHLY SIMILAR TO PUMILIO HOMOLOG 1.";"CDNA FLJ54549, HIGHLY SIMILAR TO PUMILIO HOMOLOG 1.";ISOFORM 2 OF PUMILIO HOMOLOG 1.;PUMILIO 1 ISOFORM 1.	**0.74**	0.00	1				1				1	
IPI00032460	LSM2	U6 SNRNA-ASSOCIATED SM-LIKE PROTEIN LSM2.			1				1				1	
IPI00032808	RAB3D	RAS-RELATED PROTEIN RAB-3D.	**1.37**	0.00	1		**0.84**	0.00	1				1	
IPI00032826;IPI00218038;IPI00793211;IPI00847961;IPI00892521;IPI00892883;IPI00954445	ST13;FAM10A4	HSC70-INTERACTING PROTEIN.;PROTEIN FAM10A4.;41 KDA PROTEIN.;ST13 PROTEIN.;PROTEIN.;SUPPRESSION OF TUMORIGENICITY 13.	**1.75**	0.00	2		**1.81**	0.00	1		**1.15**	0.00	2	
IPI00032851;IPI00790808;IPI00791413;IPI00792095;IPI00792696	COPZ1	COATOMER SUBUNIT ZETA-1.;7 KDA PROTEIN.;"CDNA FLJ59891, MODERATELY SIMILAR TO COATOMER SUBUNIT ZETA-1.";"CDNA FLJ59844, HIGHLY SIMILAR TO COATOMER SUBUNIT ZETA-1.";13 KDA PROTEIN.	**0.98**	0.00	1				1		**0.76**	0.00	1	
IPI00033025;IPI00642666;IPI00816201;IPI00873656;IPI00884240;IPI00941534	SEPT7;CDC10L	51 KDA PROTEIN.;NOVEL PROTEIN.;ISOFORM 2 OF SEPTIN-7.;"CDNA FLJ52574, HIGHLY SIMILAR TO SEPTIN-7.";SIMILAR TO SEPTIN-7.;ISOFORM 1 OF SEPTIN-7.			2				1				2	
IPI00033030;IPI00470921	ADRM1	PROTEASOMAL UBIQUITIN RECEPTOR ADRM1.;PUTATIVE UNCHARACTERIZED PROTEIN DKFZP686G2045.	**0.84**	0.00	1				1				1	
IPI00033494;IPI00220573;IPI00604523;IPI00719669	MYL12B;MYL12A	MYOSIN REGULATORY LIGHT CHAIN 12B.;MYOSIN REGULATORY LIGHT CHAIN 12A.;MYOSIN REGULATORY LIGHT CHAIN MRCL3 VARIANT.;MYOSIN REGULATORY LIGHT CHAIN.	**1.29**	0.15	4		**1.45**	0.14	4		**1.62**	0.23	5	
IPI00033600;IPI00395771;IPI00759705;IPI00759816;IPI00893539;IPI00893764;IPI00893774;IPI00893943;IPI00894184	PPP1R7	ISOFORM 1 OF PROTEIN PHOSPHATASE 1 REGULATORY SUBUNIT 7.;ISOFORM 2 OF PROTEIN PHOSPHATASE 1 REGULATORY SUBUNIT 7.;ISOFORM 3 OF PROTEIN PHOSPHATASE 1 REGULATORY SUBUNIT 7.;ISOFORM 4 OF PROTEIN PHOSPHATASE 1 REGULATORY SUBUNIT 7.;PROTEIN.;34 KDA PROTEIN.;PUTATIVE UNCHARACTERIZED PROTEIN PPP1R7.;32 KDA PROTEIN.	**0.94**	0.00	2				2				2	
IPI00034308;IPI00807391	SARDH	SARCOSINE DEHYDROGENASE, MITOCHONDRIAL.;SARDH PROTEIN (FRAGMENT).	**0.47**	0.05	5	0.058	**0.59**	0.07	5				5	
IPI00034319;IPI00554556;IPI00554634	CUTA	ISOFORM A OF PROTEIN CUTA.;ISOFORM C OF PROTEIN CUTA.;ISOFORM B OF PROTEIN CUTA.	**0.70**	0.02	2	0.070	**0.74**	0.04	2	0.322	**0.71**	0.00	2	
IPI00037448;IPI00550682	GRHPR	GLYOXYLATE REDUCTASE/HYDROXYPYRUVATE REDUCTASE.;GRHPR PROTEIN (FRAGMENT).	**1.11**	0.03	2				2				2	
IPI00045109;IPI00216730;IPI00219037	HIST1H2AA;HIST2H2AB;H2AFX	HISTONE H2A TYPE 1-A.;HISTONE H2A TYPE 2-B.;HISTONE H2A.X.	**1.11**	0.07	6		**0.96**	0.08	7		**0.67**	0.09	6	
IPI00045396;IPI00789155;IPI00941900;IPI00946092	CALU	ISOFORM 2 OF CALUMENIN.;"CDNA FLJ31776 FIS, CLONE NT2RI2008141, HIGHLY SIMILAR TO CALUMENIN.";ISOFORM 1 OF CALUMENIN.;PROTEIN.			1		**1.15**	0.00	1		**1.11**	0.00	1	
IPI00045839;IPI00163381;IPI00644560;IPI00761114	LEPRE1	ISOFORM 3 OF PROLYL 3-HYDROXYLASE 1.;ISOFORM 1 OF PROLYL 3-HYDROXYLASE 1.;23 KDA PROTEIN.;ISOFORM 2 OF PROLYL 3-HYDROXYLASE 1.							1					
IPI00059366;IPI00304171;IPI00744148	H2AFY	H2A HISTONE FAMILY, MEMBER Y ISOFORM 2.;ISOFORM 2 OF CORE HISTONE MACRO-H2A.1.;ISOFORM 1 OF CORE HISTONE MACRO-H2A.1.							1				1	
IPI00059476	DPEP1	DIPEPTIDASE 1.	**0.57**	0.08	7	0.028	**0.42**	0.05	7	0.082	**0.25**	0.06	7	
IPI00060181	EFHD2	EF-HAND DOMAIN-CONTAINING PROTEIN D2.			1		**0.68**	0.00	1		**1.08**	0.00	1	
IPI00060715	KCTD12	BTB/POZ DOMAIN-CONTAINING PROTEIN KCTD12.	**2.15**	0.20	3		**2.49**	0.13	4		**1.62**	0.03	4	
IPI00061005	ERP27	ENDOPLASMIC RETICULUM RESIDENT PROTEIN ERP27.	**0.92**	0.15	4		**0.49**	0.06	4				4	
IPI00061507;IPI00072044;IPI00761072	C11orf54	ISOFORM 3 OF ESTER HYDROLASE C11ORF54.;ISOFORM 1 OF ESTER HYDROLASE C11ORF54.;ISOFORM 2 OF ESTER HYDROLASE C11ORF54.	**0.98**	0.00	2		**0.96**	0.01	2				2	
IPI00061793;IPI00477577;IPI00747799;IPI00787549;IPI00900281;IPI00921885;IPI00922463;IPI00923523;IPI00924446;IPI00926122;IPI00926362;IPI00927243;IPI00953589;IPI00953656	SORBS2	SORBIN AND SH3 DOMAIN CONTAINING 2 ISOFORM 6.;ISOFORM 1 OF SORBIN AND SH3 DOMAIN-CONTAINING PROTEIN 2.;ISOFORM 5 OF SORBIN AND SH3 DOMAIN-CONTAINING PROTEIN 2.;ISOFORM 2 OF SORBIN AND SH3 DOMAIN-CONTAINING PROTEIN 2.;SORBIN AND SH3 DOMAIN CONTAINING 2 ISOFORM 4.;SORBIN AND SH3 DOMAIN CONTAINING 2 ISOFORM 5.;"CDNA FLJ55213, HIGHLY SIMILAR TO RATTUS NORVEGICUS ARG/ABL-INTERACTING PROTEIN ARGBP2 (ARGBP2), MRNA.";SORBIN AND SH3 DOMAIN CONTAINING 2 ISOFORM 3.;PROTEIN.;"CDNA FLJ32066 FIS, CLONE OCBBF1000091, HIGHLY SIMILAR TO HOMO SAPIENS SORBIN AND SH3 DOMAIN CONTAINING 2 (SORBS2), TRANSCRIPT VARIANT 2, MRNA.";10 KDA PROTEIN.			1				1		**0.55**	0.00	1	
IPI00062266	SCRN2	SECERNIN-2.			1				1				1	
IPI00063130	TMEM205	TRANSMEMBRANE PROTEIN 205.	**1.08**	0.40	2		**0.57**	0.00	2		**0.36**	0.00	2	
IPI00063234;IPI00219774;IPI00924946	PRKAR2A	PROTEIN KINASE, CAMP-DEPENDENT, REGULATORY, TYPE II, ALPHA, ISOFORM CRA_B.;CAMP-DEPENDENT PROTEIN KINASE TYPE II-ALPHA REGULATORY SUBUNIT.;13 KDA PROTEIN.	**0.83**	0.09	3		**0.60**	0.09	3	0.222			3	
IPI00063827;IPI00747859;IPI00910706;IPI00910875	ABHD14B	ISOFORM 1 OF ABHYDROLASE DOMAIN-CONTAINING PROTEIN 14B.;PUTATIVE UNCHARACTERIZED PROTEIN ABHD14B.;"CDNA FLJ52710, HIGHLY SIMILAR TO ABHYDROLASE DOMAIN-CONTAINING PROTEIN 14B.";"CDNA FLJ52723, HIGHLY SIMILAR TO ABHYDROLASE DOMAIN-CONTAINING PROTEIN 14B."			1				1				1	
IPI00069693;IPI00555744;IPI00815843;IPI00925323	RPL14	PUTATIVE UNCHARACTERIZED PROTEIN RPL14P1.;RIBOSOMAL PROTEIN L14 VARIANT.;RPL14 PROTEIN (FRAGMENT).;60S RIBOSOMAL PROTEIN L14.	**0.93**	0.15	4	0.132	**0.72**	0.13	4	0.223	**0.47**	0.10	4	
IPI00072377;IPI00301311;IPI00844040;IPI00896523;IPI00917753	SET	ISOFORM 1 OF PROTEIN SET.;ISOFORM 2 OF PROTEIN SET.;PUTATIVE UNCHARACTERIZED PROTEIN SET.;SET NUCLEAR ONCOGENE.	**0.84**	0.11	3		**0.90**	0.15	3		**1.13**	0.02	3	
IPI00072917;IPI00871621;IPI00946286	COL6A3	COL6A3 PROTEIN.;132 KDA PROTEIN.;ALPHA 3 TYPE VI COLLAGEN ISOFORM 4 PRECURSOR.	**4.24**	0.65	75		**5.50**	1.05	73		**4.07**	0.91	73	
IPI00073454;IPI00220613	COL6A2	ISOFORM 2C2A' OF COLLAGEN ALPHA-2(VI) CHAIN.;ISOFORM 2C2A OF COLLAGEN ALPHA-2(VI) CHAIN.	**3.60**	0.65	17	0.002	**5.06**	0.98	15	0.007	**4.16**	0.71	13	0.003
IPI00073772	FBP1	FRUCTOSE-1,6-BISPHOSPHATASE 1.	**0.81**	0.00	2		**0.54**	0.00	1		**0.67**	0.00	1	
IPI00075248;IPI00386621;IPI00794543;IPI00916600;IPI00916768	CALM3;CALM2;CALM1	CALMODULIN.;CALM3 PROTEIN.;"CDNA FLJ75174, HIGHLY SIMILAR TO HOMO SAPIENS CALMODULIN 1 (PHOSPHORYLASE KINASE, DELTA), MRNA.";22 KDA PROTEIN.;21 KDA PROTEIN.	**2.14**	0.64	4	0.427	**1.61**	0.25	4	0.441	**1.82**	0.38	4	
IPI00095891;IPI00219835;IPI00385033;IPI00514055;IPI00640867;IPI00644936;IPI00646491;IPI00871191	GNAS	ISOFORM XLAS-1 OF GUANINE NUCLEOTIDE-BINDING PROTEIN G(S) SUBUNIT ALPHA ISOFORMS XLAS.;ISOFORM GNAS-2 OF GUANINE NUCLEOTIDE-BINDING PROTEIN G(S) SUBUNIT ALPHA ISOFORMS SHORT.;"MUTANT GUANINE NUCLEOTIDE-BINDING PROTEIN G(S), ALPHA SUBUNIT.";ISOFORM GNAS-1 OF GUANINE NUCLEOTIDE-BINDING PROTEIN G(S) SUBUNIT ALPHA ISOFORMS SHORT.;GNAS COMPLEX LOCUS ISOFORM F.;ISOFORM 3 OF GUANINE NUCLEOTIDE-BINDING PROTEIN G(S) SUBUNIT ALPHA ISOFORMS SHORT.;ISOFORM XLAS-2 OF GUANINE NUCLEOTIDE-BINDING PROTEIN G(S) SUBUNIT ALPHA ISOFORMS XLAS.;ALPHA SUBUNIT OF GSGTP BINDING PROTEIN (FRAGMENT).			1				1				1	
IPI00096066	SUCLG2	SUCCINYL-COA LIGASE [GDP-FORMING] SUBUNIT BETA, MITOCHONDRIAL.	**1.10**	0.13	5	0.511	**0.63**	0.09	5	0.299	**0.39**	0.06	5	

IPI00098902;IPI00829595;IPI00926312;IPI00926925	OGDH	2-OXOGLUTARATE DEHYDROGENASE E1 COMPONENT, MITOCHONDRIAL.;"CDNA FLJ53308, HIGHLY SIMILAR TO 2-OXOGLUTARATE DEHYDROGENASE E1 COMPONENT, MITOCHONDRIAL.";OXOGLUTARATE DEHYDROGENASE ISOFORM 3 PRECURSOR.;OGDH PROTEIN.	**1.33**	0.03	2	0.376	**0.96**	0.04	2		**0.49**	0.07	3	0.482
IPI00099670;IPI00843810	CEL	CARBOXYL ESTER LIPASE PRECURSOR.;BILE SALT-ACTIVATED LIPASE PRECURSOR.	**0.67**	0.09	37	0.000	**0.35**	0.11	35	0.000	**0.03**	0.01	34	0.151
IPI00100154;IPI00167490;IPI00654582	TOLLIP	TOLL-INTERACTING PROTEIN.;"CDNA FLJ39374 FIS, CLONE PEBLM2008576, HIGHLY SIMILAR TO HOMO SAPIENS TOLLIP PROTEIN.";TOLL INTERACTING PROTEIN VARIANT (FRAGMENT).							1				1	
IPI00100160;IPI00604431	CAND1	ISOFORM 1 OF CULLIN-ASSOCIATED NEDD8-DISSOCIATED PROTEIN 1.;ISOFORM 2 OF CULLIN-ASSOCIATED NEDD8-DISSOCIATED PROTEIN 1.	**0.81**	0.04	3	0.214	**0.98**	0.06	3	0.515	**0.63**	0.07	3	0.285
IPI00100656;IPI00644037	TECR	ISOFORM 1 OF TRANS-2,3-ENOYL-COA REDUCTASE.;"CDNA FLJ36768 FIS, CLONE ADIPS1000064, HIGHLY SIMILAR TO SYNAPTIC GLYCOPROTEIN SC2."			1				1				1	
IPI00100980	EHD2	EH DOMAIN-CONTAINING PROTEIN 2.			2				2		**1.03**	0.00	1	
IPI00101037	RCN3	RETICULOCALBIN-3.							2				1	
IPI00101782;IPI00657888	GMPPA	ISOFORM 1 OF MANNOSE-1-PHOSPHATE GUANYLTRANSFERASE ALPHA.;ISOFORM 2 OF MANNOSE-1-PHOSPHATE GUANYLTRANSFERASE ALPHA.	**1.00**	0.17	3		**0.65**	0.00	3				3	
IPI00102165	H2AFJ	ISOFORM 2 OF HISTONE H2A.J.	**1.19**	0.11	7	0.351				0.450				0.598
IPI00102821	MGC29506	ISOFORM 1 OF PROAPOPTOTIC CASPASE ADAPTER PROTEIN.			1								1	
IPI00102936;IPI00168388	SRP68	ISOFORM 2 OF SIGNAL RECOGNITION PARTICLE 68 KDA PROTEIN.;ISOFORM 1 OF SIGNAL RECOGNITION PARTICLE 68 KDA PROTEIN.			1				1				1	
IPI00103467	ALDH1B1	ALDEHYDE DEHYDROGENASE X, MITOCHONDRIAL.	**2.18**	0.00	1		**1.04**	0.00	1	0.618	**0.52**	0.00	2	
IPI00103994;IPI00871954;IPI00939672	LARS	LEUCYL-TRNA SYNTHETASE, CYTOPLASMIC.;"CDNA FLJ58466, HIGHLY SIMILAR TO LEUCYL-TRNA SYNTHETASE, CYTOPLASMIC.";"CDNA FLJ58533, HIGHLY SIMILAR TO LEUCYL-TRNA SYNTHETASE, CYTOPLASMIC."			1				1				1	
IPI00104128;IPI00790799;IPI00873608;IPI00940637	SEC11A	SIGNAL PEPTIDASE COMPLEX CATALYTIC SUBUNIT SEC11A.;16 KDA PROTEIN.;SIGNAL PEPTIDASE COMPLEX SPC-18.;19 KDA PROTEIN.	**0.86**	0.00	2				2				2	
IPI00104341;IPI00909862;IPI00942976	EPHX2	CDNA FLJ59619, HIGHLY SIMILAR TO EPOXIDE HYDROLASE 2.;"CDNA FLJ54951, HIGHLY SIMILAR TO EPOXIDE HYDROLASE 2.";EPOXIDE HYDROLASE 2.	**0.94**	0.00	1				1				1	
IPI00106642	SDF2L1	DIHYDROPYRIMIDINASE-LIKE 2.	**1.79**	0.03	4		**1.95**	0.19	5		**1.41**	0.19	4	
IPI00106668;IPI00303882;IPI00943550	PLIN3	MANNOSE 6 PHOSPHATE RECEPTOR BINDING PROTEIN 1 ISOFORM 3.;ISOFORM B OF MANNOSE-6-PHOSPHATE RECEPTOR-BINDING PROTEIN 1.;MANNOSE 6 PHOSPHATE RECEPTOR BINDING PROTEIN 1 ISOFORM 2.							1				1	
IPI00106687	LXN	LATEXIN.	**4.63**	0.00	1		**8.46**	0.00	1		**7.26**	0.00	1	
IPI00140420;IPI00910438	SND1	STAPHYLOCOCCAL NUCLEASE DOMAIN-CONTAINING PROTEIN 1.;"CDNA FLJ54574, HIGHLY SIMILAR TO STAPHYLOCOCCAL NUCLEASE DOMAIN- CONTAINING PROTEIN 1."	**0.71**	0.06	19	0.004	**0.67**	0.12	19	0.014	**0.25**	0.05	19	0.054
IPI00140827;IPI00299147;IPI00299149;IPI00434968;IPI00745893;IPI00790142;IPI00793687;IPI00798011;IPI00827816;IPI00909492	LOC728825;SUMO3;SUMO2;SUMO4	SMT3 SUPPRESSOR OF MIF TWO 3 HOMOLOG 2 ISOFORM B PRECURSOR.;SMALL UBIQUITIN-RELATED MODIFIER 3.;SMALL UBIQUITIN-RELATED MODIFIER 2.;SMALL UBIQUITIN-RELATED MODIFIER 4.;10 KDA PROTEIN.;"SMT3 SUPPRESSOR OF MIF TWO 3 HOMOLOG 3 (YEAST), ISOFORM CRA_D.";PUTATIVE UNCHARACTERIZED PROTEIN SUMO3.;SMT3 SUPPRESSOR OF MIF TWO 3 HOMOLOG 4 PRECURSOR.;"CDNA FLJ57440, MODERATELY SIMILAR TO SMALL UBIQUITIN-RELATED MODIFIER 3."	**1.32**	0.00	1		**2.02**	0.00	1		**1.86**	0.00	1	
IPI00141318;IPI00604713;IPI00883950;IPI00939370	CKAP4	ISOFORM 1 OF CYTOSKELETON-ASSOCIATED PROTEIN 4.;ISOFORM 2 OF CYTOSKELETON-ASSOCIATED PROTEIN 4.;SIMILAR TO CYTOSKELETON-ASSOCIATED PROTEIN 4 (FRAGMENT).;CKAP4 PROTEIN (FRAGMENT).	**1.45**	0.12	7	0.121	**1.00**	0.06	7	0.619	**1.15**	0.19	8	0.414
IPI00147874	NANS	SIALIC ACID SYNTHASE.	**0.83**	0.00	3		**0.56**	0.00	3		**0.37**	0.00	2	
IPI00156232;IPI00221091	RPS15A	15 KDA PROTEIN.;40S RIBOSOMAL PROTEIN S15A.	**0.82**	0.00	1		**0.50**	0.00	1		**0.25**	0.00	1	
IPI00156689	VAT1	SYNAPTIC VESICLE MEMBRANE PROTEIN VAT-1 HOMOLOG.	**1.97**	0.16	5	0.059	**2.59**	0.52	4	0.167	**0.96**	0.28	4	0.683
IPI00157790	KIAA0368	KIAA0368 PROTEIN.	**2.51**	0.00	1		**1.32**	0.00	1		**0.96**	0.00	1	
IPI00163187;IPI00747810	FSCN1	FASCIN.;FSCN1 PROTEIN (FRAGMENT).			1				1				1	
IPI00165230;IPI00335930	DAZAP1	ISOFORM 1 OF DAZ-ASSOCIATED PROTEIN 1.;ISOFORM 2 OF DAZ-ASSOCIATED PROTEIN 1.	**1.22**	0.00	1		**1.57**	0.00	1				1	
IPI00165261;IPI00794956	SCFD1	SEC1 FAMILY DOMAIN-CONTAINING PROTEIN 1.;PUTATIVE UNCHARACTERIZED PROTEIN SCFD1.	**0.61**	0.00	3		**0.73**	0.00	3		**1.24**	0.00	3	
IPI00165360;IPI00910977	MPST	3-MERCAPTOPYRUVATE SULFURTRANSFERASE.;MERCAPTOPYRUVATE SULFURTRANSFERASE ISOFORM 1.	**1.25**	0.01	3		**1.08**	0.21	2		**0.87**	0.00	2	
IPI00165949;IPI00477831	ERAP1	ISOFORM 2 OF ENDOPLASMIC RETICULUM AMINOPEPTIDASE 1.;ISOFORM 1 OF ENDOPLASMIC RETICULUM AMINOPEPTIDASE 1.	**2.16**	0.00	1				1				1	
IPI00166079	VKORC1L1	VITAMIN K EPOXIDE REDUCTASE COMPLEX SUBUNIT 1-LIKE PROTEIN 1.	**0.69**	0.00	1				1		**0.65**	0.00	1	
IPI00166729;IPI00924948	AZGP1	ALPHA-2-GLYCOPROTEIN 1, ZINC.;26 KDA PROTEIN.											1	
IPI00166768;IPI00218343;IPI00792677;IPI00793930;IPI00930688	TUBA1C;TUBA1B	TUBA1C PROTEIN.;TUBULIN ALPHA-1C CHAIN.;"CDNA FLJ60097, HIGHLY SIMILAR TO TUBULIN ALPHA-UBIQUITOUS CHAIN.";TUBA1B PROTEIN.;TUBULIN ALPHA-1B CHAIN.	**0.99**	0.08	10		**1.33**	0.13	10		**1.12**	0.10	10	
IPI00166865	CISD2	CDGSH IRON SULFUR DOMAIN-CONTAINING PROTEIN 2.											1	
IPI00167072;IPI00170896;IPI00171152;IPI00171691;IPI00171692;IPI00922400;IPI00922889;IPI00925250;IPI00927072	ABHD11	ISOFORM 5 OF ABHYDROLASE DOMAIN-CONTAINING PROTEIN 11.;ISOFORM 3 OF ABHYDROLASE DOMAIN-CONTAINING PROTEIN 11.;ISOFORM 4 OF ABHYDROLASE DOMAIN-CONTAINING PROTEIN 11.;ISOFORM 2 OF ABHYDROLASE DOMAIN-CONTAINING PROTEIN 11.;ISOFORM 1 OF ABHYDROLASE DOMAIN-CONTAINING PROTEIN 11.;ABHYDROLASE DOMAIN CONTAINING 11 7.;ABHYDROLASE DOMAIN CONTAINING 11 ISOFORM 8.;PROTEIN.			1									
IPI00168184;IPI00554737;IPI00798040;IPI00908543;IPI00910732	PPP2R1A	CDNA FLJ34068 FIS, CLONE FCBBF3001918, HIGHLY SIMILAR TO SERINE/THREONINE PROTEIN PHOSPHATASE 2A, 65 KDA REGULATORY SUBUNIT A, ALPHA ISOFORM.;SERINE/THREONINE-PROTEIN PHOSPHATASE 2A 65 KDA REGULATORY SUBUNIT A ALPHA ISOFORM.;"CDNA FLJ33169 FIS, CLONE ADRGL2000384, HIGHLY SIMILAR TO SERINE/THREONINE-PROTEIN PHOSPHATASE 2A 65 KDA REGULATORY SUBUNIT A ALPHA ISOFORM.";"CDNA FLJ56133, HIGHLY SIMILAR TO SERINE/THREONINE-PROTEIN PHOSPHATASE 2A 65 KDA REGULATORY SUBUNIT A ALPHA ISOFORM.";"CDNA FLJ56053, HIGHLY SIMILAR TO SERINE/THREONINE-PROTEIN PHOSPHATASE 2A 65 KDA REGULATORY SUBUNIT A ALPHA ISOFORM."	**1.46**	0.14	3	0.227	**1.41**	0.11	3	0.316	**1.30**	0.08	3	0.387
IPI00169383;IPI00916818	PGK1	PHOSPHOGLYCERATE KINASE 1.;PHOSPHOGLYCERATE KINASE.	**0.96**	0.17	12	0.260	**1.24**	0.12	12	0.335	**1.22**	0.24	12	0.312
IPI00170436;IPI00645667;IPI00657706;IPI00885058	MBOAT7	LEUKOCYTE RECEPTOR CLUSTER (LRC) MEMBER 4.;ISOFORM 3 OF LYSOPHOSPHOLIPID ACYLTRANSFERASE 7.;ISOFORM 1 OF LYSOPHOSPHOLIPID ACYLTRANSFERASE 7.;ISOFORM 2 OF LYSOPHOSPHOLIPID ACYLTRANSFERASE 7.	**1.34**	0.00	1				1				1	
IPI00170935	LRRC47	LEUCINE-RICH REPEAT-CONTAINING PROTEIN 47.	**1.22**	0.00	1		**0.54**	0.00	1				1	
IPI00171438;IPI00395646;IPI00939560;IPI00946258	TXNDC5;MUTED;TXNDC5;MUTED	THIOREDOXIN DOMAIN-CONTAINING PROTEIN 5.;THIOREDOXIN DOMAIN CONTAINING 5 ISOFORM 2.;THIOREDOXIN DOMAIN CONTAINING 5 ISOFORM 3.;PUTATIVE UNCHARACTERIZED PROTEIN DKFZP666I134 (FRAGMENT).	**1.32**	0.00	1	0.481	**1.04**	0.00	1	0.463	**0.69**	0.00	1	0.473
IPI00171903;IPI00383296	HNRNPM	ISOFORM 1 OF HETEROGENEOUS NUCLEAR RIBONUCLEOPROTEIN M.;ISOFORM 2 OF HETEROGENEOUS NUCLEAR RIBONUCLEOPROTEIN M.	**1.25**	0.06	5	0.395	**3.19**	1.17	5	0.469	**1.12**	0.10	6	0.671
IPI00173549;IPI00900276;IPI00900324;IPI00942427	SYNPO2	SYNAPTOPODIN 2 ISOFORM A.;SYNAPTOPODIN 2 ISOFORM C.;SYNAPTOPODIN-2.;118 KDA PROTEIN.			1								1	
IPI00173589;IPI00419919;IPI00796934	RPL29P4;RPL29	17 KDA PROTEIN.;60S RIBOSOMAL PROTEIN L29.;"CDNA FLJ78093, HIGHLY SIMILAR TO HOMO SAPIENS RIBOSOMAL PROTEIN L29 (RPL29), MRNA."	**0.74**	0.00	2		**0.48**	0.00	2		**0.22**	0.00	2	
IPI00176193;IPI00550918	COL14A1	ISOFORM 1 OF COLLAGEN ALPHA-1(XIV) CHAIN.;ISOFORM 2 OF COLLAGEN ALPHA-1(XIV) CHAIN.	**6.95**	1.32	32	0.177	**16.39**	3.13	34	0.082	**11.38**	1.87	37	0.274
IPI00176903;IPI00514023	PTRF	ISOFORM 1 OF POLYMERASE I AND TRANSCRIPT RELEASE FACTOR.;"CDNA FLJ53495, HIGHLY SIMILAR TO POLYMERASE I AND TRANSCRIPT RELEASE FACTOR."	**2.39**	0.00	4		**2.91**	0.01	4		**1.39**	0.14	4	
IPI00177728	CNDP2	ISOFORM 1 OF CYTOSOLIC NON-SPECIFIC DIPEPTIDASE.	**1.17**	0.06	7	0.340	**1.07**	0.10	5	0.459	**0.66**	0.08	5	0.344
IPI00177817;IPI00219078;IPI00747443	ATP2A2	ISOFORM SERCA2A OF SARCOPLASMIC/ENDOPLASMIC RETICULUM CALCIUM ATPASE 2.;ISOFORM SERCA2B OF SARCOPLASMIC/ENDOPLASMIC RETICULUM CALCIUM ATPASE 2.;PUTATIVE UNCHARACTERIZED PROTEIN ATP2A2.	**1.03**	0.17	2		**0.61**	0.00	2		**0.46**	0.00	2	
IPI00178352;IPI00413958	FLNC	ISOFORM 1 OF FILAMIN-C.;ISOFORM 2 OF FILAMIN-C.				0.044				0.030	**4.32**	0.66	11	0.070
IPI00178440	EEF1B2	ELONGATION FACTOR 1-BETA.	**0.93**	0.06	3	0.345	**0.55**	0.07	3	0.272	**0.28**	0.00	3	
IPI00179183;IPI00216580;IPI00385067;IPI00643086	ASB9	ISOFORM 1 OF ANKYRIN REPEAT AND SOCS BOX PROTEIN 9.;ISOFORM 2 OF ANKYRIN REPEAT AND SOCS BOX PROTEIN 9.;ANKYRIN REPEAT AND SOCS BOX-CONTAINING 9.;"ANKYRIN REPEAT AND SOCS BOX-CONTAINING 9, ISOFORM CRA_D."							1					

IPI00179330;IPI00418813;IPI00456429;IPI00719280;IPI00784990;IPI00789107;IPI00789823;IPI00790633;IPI00792712;IPI00793330;IPI00793729;IPI00793810;IPI00794205;IPI00794211;IPI00794925;IPI00795527;IPI00796007;IPI00796600;IPI00797400;IPI00798127;IPI00798155	UBC;UBB;RPS27A;UBA52	UBIQUITIN AND RIBOSOMAL PROTEIN S27A PRECURSOR.;PSEUDOGENE CANDIDATE.;UBIQUITIN AND RIBOSOMAL PROTEIN L40 PRECURSOR.;UBIQUITIN B PRECURSOR.;UBIQUITIN C SPLICE VARIANT.;30 KDA PROTEIN.;16 KDA PROTEIN.;25 KDA PROTEIN.;39 KDA PROTEIN.;12 KDA PROTEIN.;UBC PROTEIN.;19 KDA PROTEIN.;26 KDA PROTEIN.;18 KDA PROTEIN.;21 KDA PROTEIN.;"CDNA FLJ51326, HIGHLY SIMILAR TO HOMO SAPIENS UBIQUITIN B (UBB), MRNA.";UBIQUITIN C.;UBIQUITIN.	**1.26**	0.09	3		**1.37**	0.23	3		**0.97**	0.08	3	
IPI00179589;IPI00924816;IPI00926178	LUZP6;MTPN;MTPN	14 KDA PROTEIN.;MYOTROPHIN.;6 KDA PROTEIN.	**1.24**	0.00	1		**1.64**	0.00	1		**1.67**	0.00	1	
IPI00179709;IPI00180675;IPI00218345;IPI00410402;IPI00784332;IPI00915791;IPI00936821	TUBA3C;TUBA3D;TUBA1A;TUBA3E	ISOFORM 1 OF TUBULIN ALPHA-3C/D CHAIN.;TUBULIN ALPHA-1A CHAIN.;ISOFORM 2 OF TUBULIN ALPHA-3C/D CHAIN.;TUBULIN ALPHA-3E CHAIN.;TUBULIN ALPHA-3C/D CHAIN.;46 KDA PROTEIN.;"CDNA FLJ53743, HIGHLY SIMILAR TO TUBULIN ALPHA-3 CHAIN."	**1.56**	0.38	6		**1.64**	0.43	6		**1.40**	0.26	6	
IPI00179964;IPI00183626;IPI00334175	PTBP1	ISOFORM 1 OF POLYPYRIMIDINE TRACT-BINDING PROTEIN 1.;POLYPYRIMIDINE TRACT-BINDING PROTEIN 1 ISOFORM A.;ISOFORM 2 OF POLYPYRIMIDINE TRACT-BINDING PROTEIN 1.	**1.00**	0.02	6	0.427	**0.91**	0.07	6	0.545	**0.66**	0.02	7	0.486
IPI00180954;IPI00443657;IPI00641579;IPI00646241;IPI00909097;IPI00910502	CIRBP	COLD-INDUCIBLE RNA-BINDING PROTEIN.;"CDNA FLJ46566 FIS, CLONE THYMU3040829, MODERATELY SIMILAR TO COLD- INDUCIBLE RNA-BINDING PROTEIN.";"CDNA FLJ37462 FIS, CLONE BRAWH2011343, HIGHLY SIMILAR TO COLD- INDUCIBLE RNA-BINDING PROTEIN.";18 KDA PROTEIN.;"CDNA FLJ52850, MODERATELY SIMILAR TO COLD-INDUCIBLE RNA-BINDING PROTEIN.";"CDNA FLJ52834, MODERATELY SIMILAR TO COLD-INDUCIBLE RNA-BINDING PROTEIN."	**0.84**	0.08	4	0.506	**1.07**	0.07	4				4	
IPI00180956;IPI00240503;IPI00798387		PUTATIVE UNCHARACTERIZED PROTEIN KRT17P1.;48 KDA PROTEIN.				0.185				0.459	**3.57**	0.13	6	0.473
IPI00182469;IPI00182540;IPI00219725;IPI00219726;IPI00219727;IPI00219728;IPI00219730;IPI00219731;IPI00219732;IPI00219733;IPI00219734;IPI00219735;IPI00219737;IPI00219738;IPI00219739;IPI00219741;IPI00219742;IPI00219868;IPI00219869;IPI00219870;IPI00219872;IPI00219873;IPI00219875;IPI00419482;IPI00845519;IPI00940850;IPI00942869	CTNND1	ISOFORM 1AB OF CATENIN DELTA-1.;106 KDA PROTEIN.;ISOFORM 2AB OF CATENIN DELTA-1.;ISOFORM 2AC OF CATENIN DELTA-1.;ISOFORM 2BC OF CATENIN DELTA-1.;ISOFORM 2A OF CATENIN DELTA-1.;ISOFORM 2B OF CATENIN DELTA-1.;ISOFORM 2C OF CATENIN DELTA-1.;ISOFORM 2 OF CATENIN DELTA-1.;ISOFORM 3ABC OF CATENIN DELTA-1.;ISOFORM 3AB OF CATENIN DELTA-1.;ISOFORM 3AC OF CATENIN DELTA-1.;ISOFORM 3BC OF CATENIN DELTA-1.;ISOFORM 3A OF CATENIN DELTA-1.;ISOFORM 3B OF CATENIN DELTA-1.;ISOFORM 3C OF CATENIN DELTA-1.;ISOFORM 3 OF CATENIN DELTA-1.;ISOFORM 1AC OF CATENIN DELTA-1.;ISOFORM 1BC OF CATENIN DELTA-1.;ISOFORM 1A OF CATENIN DELTA-1.;ISOFORM 1B OF CATENIN DELTA-1.;ISOFORM 1C OF CATENIN DELTA-1.;ISOFORM 2ABC OF CATENIN DELTA-1.;"CATENIN, DELTA 1 ISOFORM 1B.";ISOFORM 1 OF CATENIN DELTA-1.;103 KDA PROTEIN.;ISOFORM 1ABC OF CATENIN DELTA-1.	**1.07**	0.00	1		**1.15**	0.00	1		**0.84**	0.00	1	
IPI00182933;IPI00397860;IPI00871858	CYB5A	ISOFORM 2 OF CYTOCHROME B5.;ISOFORM 1 OF CYTOCHROME B5.;CYTOCHROME B-5 ISOFORM 1 VARIANT.	**1.02**	0.07	7	0.433	**0.57**	0.06	7	0.124			7	
IPI00184311;IPI00478248	ENPP1	ECTONUCLEOTIDE PYROPHOSPHATASE/PHOSPHODIESTERASE FAMILY MEMBER 1.;100 KDA PROTEIN.	**0.79**	0.00	1		**0.54**	0.00	1		**0.21**	0.00	1	
IPI00185374	PSMD12	26S PROTEASOME NON-ATPASE REGULATORY SUBUNIT 12.	**1.59**	0.00	1		**1.18**	0.00	1		**0.80**	0.00	1	
IPI00185919;IPI00411690;IPI00643027	LARP1	ISOFORM 1 OF LA-RELATED PROTEIN 1.;ISOFORM 3 OF LA-RELATED PROTEIN 1.;ISOFORM 2 OF LA-RELATED PROTEIN 1.			1				1				1	
IPI00186290	EEF2	ELONGATION FACTOR 2.	**0.64**	0.09	13	0.004	**0.61**	0.10	12	0.034	**0.26**	0.03	12	0.026
IPI00186460;IPI00936892	COL2A1	ISOFORM 1 OF COLLAGEN ALPHA-1(II) CHAIN.;ISOFORM 2 OF COLLAGEN ALPHA-1(II) CHAIN.			2				2				2	
IPI00215637;IPI00910433	DDX3X	ATP-DEPENDENT RNA HELICASE DDX3X.;"CDNA FLJ60675, HIGHLY SIMILAR TO ATP-DEPENDENT RNA HELICASE DDX3X."	**0.59**	0.00	2	0.186	**1.22**	0.00	2	0.328	**0.52**	0.00	2	0.347
IPI00215719	RPL18	60S RIBOSOMAL PROTEIN L18.	**0.67**	0.01	3	0.116	**0.36**	0.02	3	0.098	**0.23**	0.01	3	0.157
IPI00215743;IPI00856098	RRBP1	ISOFORM 3 OF RIBOSOME-BINDING PROTEIN 1.;P180/RIBOSOME RECEPTOR.	**0.79**	0.13	44	0.000	**0.54**	0.12	44	0.000	**0.19**	0.03	44	0.001
IPI00215746	FABP4	FATTY ACID-BINDING PROTEIN, ADIPOCYTE.			2				2		**0.31**	0.00	2	
IPI00215780	RPS19	40S RIBOSOMAL PROTEIN S19.	**0.77**	0.13	7	0.130	**0.65**	0.17	7	0.235	**0.25**	0.02	7	0.102
IPI00215884;IPI00218591;IPI00218592	SFRS1	ISOFORM ASF-1 OF SPLICING FACTOR, ARGININE/SERINE-RICH 1.;"ISOFORM ASF-2 OF SPLICING FACTOR, ARGININE/SERINE-RICH 1.";"ISOFORM ASF-3 OF SPLICING FACTOR, ARGININE/SERINE-RICH 1."	**1.48**	0.00	3		**1.24**	0.00	2		**1.15**	0.00	2	
IPI00215888	SRP72	SIGNAL RECOGNITION PARTICLE 72 KDA PROTEIN.	**0.78**	0.00	2				2				1	
IPI00215901;IPI00218988;IPI00921960;IPI00922165	AK2	ISOFORM 1 OF ADENYLATE KINASE 2, MITOCHONDRIAL.;"ISOFORM 2 OF ADENYLATE KINASE 2, MITOCHONDRIAL.";"ISOFORM 6 OF ADENYLATE KINASE 2, MITOCHONDRIAL.";"ISOFORM 5 OF ADENYLATE KINASE 2, MITOCHONDRIAL."	**1.12**	0.00	1		**1.05**	0.00	1		**0.45**	0.00	1	
IPI00215911	APEX1	DNA-(APURINIC OR APYRIMIDINIC SITE) LYASE.	**1.52**	0.00	1		**1.65**	0.00	1		**0.91**	0.00	2	
IPI00215914;IPI00215917;IPI00930263	ARF1;ARF3	ADP-RIBOSYLATION FACTOR 1.;ADP-RIBOSYLATION FACTOR 3.;"CDNA FLJ61099, HIGHLY SIMILAR TO ADP-RIBOSYLATION FACTOR 1."	**1.16**	0.04	4	0.222	**1.17**	0.08	4	0.578	**0.89**	0.11	4	0.606
IPI00215918;IPI00792330;IPI00946929	ARF4	ADP-RIBOSYLATION FACTOR 4.;15 KDA PROTEIN.;18 KDA PROTEIN.	**1.03**	0.03	2	0.354	**0.76**	0.20	2	0.303	**0.95**	0.09	2	0.598
IPI00215925	GNMT	GLYCINE N-METHYLTRANSFERASE.	**1.59**	0.04	2	0.417	**0.69**	0.15	2				2	
IPI00215948;IPI00473136;IPI00953077	CTNNA1	ISOFORM 1 OF CATENIN ALPHA-1.;ISOFORM 2 OF CATENIN ALPHA-1.	**0.99**	0.02	2		**1.24**	0.00	2				3	
IPI00215983	CA1	CARBONIC ANHYDRASE 1.	**1.59**	0.24	5	0.191	**5.30**	0.76	7	0.189	**2.33**	0.19	7	0.371
IPI00216008;IPI00289800;IPI00645745;IPI00853547;IPI00884082	G6PD	ISOFORM LONG OF GLUCOSE-6-PHOSPHATE 1-DEHYDROGENASE.;ISOFORM SHORT OF GLUCOSE-6-PHOSPHATE 1-DEHYDROGENASE.;37 KDA PROTEIN.;ISOFORM 3 OF GLUCOSE-6-PHOSPHATE 1-DEHYDROGENASE.;GLUCOSE-6-PHOSPHATE 1-DEHYDROGENASE.	**1.78**	0.00	1		**2.96**	0.00	1		**1.91**	0.00	1	
IPI00216049;IPI00216746;IPI00514561;IPI00807545;IPI00910458	HNRNPK	ISOFORM 1 OF HETEROGENEOUS NUCLEAR RIBONUCLEOPROTEIN K.;ISOFORM 2 OF HETEROGENEOUS NUCLEAR RIBONUCLEOPROTEIN K.;HETEROGENEOUS NUCLEAR RIBONUCLEOPROTEIN K.;ISOFORM 3 OF HETEROGENEOUS NUCLEAR RIBONUCLEOPROTEIN K.;"CDNA FLJ54552, HIGHLY SIMILAR TO HETEROGENEOUS NUCLEAR RIBONUCLEOPROTEIN K."	**1.17**	0.10	12	0.318	**1.13**	0.10	14	0.578	**0.85**	0.10	13	0.424
IPI00216106;IPI00290416;IPI00908669;IPI00916410;IPI00916847	OLA1	ISOFORM 3 OF OBG-LIKE ATPASE 1.;ISOFORM 1 OF OBG-LIKE ATPASE 1.;"CDNA FLJ58890, HIGHLY SIMILAR TO GTP-BINDING PROTEIN PTD004.";PROTEIN.;47 KDA PROTEIN.	**0.67**	0.05	2		**0.52**	0.00	2		**0.38**	0.00	2	
IPI00216138	TAGLN	TRANSGELIN.	**3.19**	0.45	7	0.269	**4.95**	0.31	8	0.167	**7.14**	0.37	7	0.169
IPI00216293;IPI00878140	TST	THIOSULFATE SULFURTRANSFERASE.;"CDNA FLJ57870, HIGHLY SIMILAR TO THIOSULFATE SULFURTRANSFERASE."	**1.14**	0.00	3	0.369	**0.59**	0.07	3	0.325	**0.56**	0.00	3	
IPI00216298;IPI00552768	TXN	THIOREDOXIN.;"THIOREDOXIN, ISOFORM CRA_B."	**1.04**	0.00	3		**0.95**	0.00	1		**0.86**	0.00	1	
IPI00216308;IPI00790304;IPI00910830	VDAC1	VOLTAGE-DEPENDENT ANION-SELECTIVE CHANNEL PROTEIN 1.;20 KDA PROTEIN.;"CDNA FLJ57715, HIGHLY SIMILAR TO VOLTAGE-DEPENDENT ANION-SELECTIVE CHANNEL PROTEIN 1."	**1.00**	0.00	1		**0.90**	0.00	1				1	
IPI00216318;IPI00759832	YWHAB	ISOFORM LONG OF 14-3-3 PROTEIN BETA/ALPHA.;ISOFORM SHORT OF 14-3-3 PROTEIN BETA/ALPHA.	**1.03**	0.10	10	0.346	**1.14**	0.12	11	0.610	**1.52**	0.18	12	0.268
IPI00216319	YWHAH	14-3-3 PROTEIN ETA.	**0.98**	0.15	5		**1.37**	0.38	6	0.448	**1.49**	0.09	8	0.398
IPI00216348;IPI00302712;IPI00744015;IPI00827813;IPI00827859;IPI00915993	DYNC1I2	ISOFORM 2C OF CYTOPLASMIC DYNEIN 1 INTERMEDIATE CHAIN 2.;ISOFORM 2B OF CYTOPLASMIC DYNEIN 1 INTERMEDIATE CHAIN 2.;ISOFORM 2A OF CYTOPLASMIC DYNEIN 1 INTERMEDIATE CHAIN 2.;ISOFORM 2F OF CYTOPLASMIC DYNEIN 1 INTERMEDIATE CHAIN 2.;ISOFORM 2E OF CYTOPLASMIC DYNEIN 1 INTERMEDIATE CHAIN 2.;"CDNA, FLJ79018, HIGHLY SIMILAR TO CYTOPLASMIC DYNEIN 1 INTERMEDIATE CHAIN 2."			1		**1.11**	0.00	1				1	
IPI00216457;IPI00339274	HIST2H2AA3;HIST2H2AA4;HIST2H2AC	HISTONE H2A TYPE 2-A.;HISTONE H2A TYPE 2-C.	**1.15**	0.08	9	0.321	**0.94**	0.32	9	0.463	**0.70**	0.22	9	0.524
IPI00216587;IPI00645201	RPS8	40S RIBOSOMAL PROTEIN S8.;RIBOSOMAL PROTEIN S8.	**0.81**	0.06	3	0.177	**0.60**	0.08	3	0.197	**0.34**	0.01	3	0.125
IPI00216592;IPI00477313;IPI00759596;IPI00868835;IPI00909232;IPI00910666;IPI00910718	HNRNPC	ISOFORM C1 OF HETEROGENEOUS NUCLEAR RIBONUCLEOPROTEINS C1/C2.;ISOFORM C2 OF HETEROGENEOUS NUCLEAR RIBONUCLEOPROTEINS C1/C2.;ISOFORM 4 OF HETEROGENEOUS NUCLEAR RIBONUCLEOPROTEINS C1/C2.;SIMILAR TO HETEROGENEOUS NUCLEAR RIBONUCLEOPROTEINS C1/C2 (HNRNP C1 / HNRNP C2). ISOFORM 4.;"CDNA FLJ53542, HIGHLY SIMILAR TO HETEROGENEOUS NUCLEAR RIBONUCLEOPROTEINS C.";"CDNA FLJ52993, HIGHLY SIMILAR TO HETEROGENEOUS NUCLEAR RIBONUCLEOPROTEINS C.";"CDNA FLJ52975, HIGHLY SIMILAR TO HETEROGENEOUS NUCLEAR RIBONUCLEOPROTEINS C."	**1.13**	0.03	5		**1.35**	0.14	3	0.610	**0.85**	0.15	4	0.591
IPI00216654;IPI00292387;IPI00908873	NOLC1	ISOFORM BETA OF NUCLEOLAR PHOSPHOPROTEIN P130.;ISOFORM ALPHA OF NUCLEOLAR PHOSPHOPROTEIN P130.;ISOFORM 3 OF NUCLEOLAR PHOSPHOPROTEIN P130.	**1.15**	0.02	2		**1.19**	0.00	2		**0.78**	0.00	2	
IPI00216691	PFN1	PROFILIN-1.	**1.38**	0.14	6	0.244	**1.81**	0.04	7	0.302	**2.13**	0.13	7	0.060
IPI00216694;IPI00947227	PLS3	PLASTIN-3.;"CDNA FLJ59681, HIGHLY SIMILAR TO PLASTIN-3."					**1.62**	0.00	3					

IPI00216697;IPI00297532;IPI00374973;IPI00646300;IPI00654646;IPI00744408;IPI00744846;IPI00744895;IPI00744986;IPI00745348;IPI00747536;IPI00747552;IPI00748074;IPI00748330;IPI00748439;IPI00748895;IPI00749301	ANK1	ISOFORM ER1 OF ANKYRIN-1.;ISOFORM ER2 OF ANKYRIN-1.;ISOFORM ER3 OF ANKYRIN-1.;ISOFORM ER16 OF ANKYRIN-1.;ISOFORM ER5 OF ANKYRIN-1.;ISOFORM ER7 OF ANKYRIN-1.;ISOFORM ER10 OF ANKYRIN-1.;ISOFORM ER6 OF ANKYRIN-1.;ISOFORM ER9 OF ANKYRIN-1.;ISOFORM ER4 OF ANKYRIN-1.;ISOFORM ER11 OF ANKYRIN-1.;ISOFORM ER8 OF ANKYRIN-1.;ISOFORM ER15 OF ANKYRIN-1.;ISOFORM BR21 OF ANKYRIN-1.;ISOFORM ER14 OF ANKYRIN-1.;ISOFORM ER13 OF ANKYRIN-1.;ISOFORM ER12 OF ANKYRIN-1.							1					
IPI00216699;IPI00397834	FERMT3	ISOFORM 2 OF FERMITIN FAMILY HOMOLOG 3.;ISOFORM 1 OF FERMITIN FAMILY HOMOLOG 3.			1				1				1	
IPI00216704;IPI00237806;IPI00384172;IPI00783228;IPI00914949	SPTB	ISOFORM 2 OF SPECTRIN BETA CHAIN, ERYTHROCYTE.;"ISOFORM 3 OF SPECTRIN BETA CHAIN, ERYTHROCYTE.";BETA I SPECTRIN FORM BETAI SIGMA3.;"ISOFORM 1 OF SPECTRIN BETA CHAIN, ERYTHROCYTE.";"SPECTRIN, BETA, ERYTHROCYTIC (INCLUDES SPHEROCYTOSIS, CLINICAL TYPE I) VARIANT (FRAGMENT)."	**1.75**	0.00	2		**3.21**	0.00	3		**1.49**	0.00	2	
IPI00216773	ALB	ALB PROTEIN.	**1.74**	0.62	32	0.369	**3.22**	0.89	30	0.008	**4.18**	0.91	31	0.014
IPI00216951	DARS	ASPARTYL-TRNA SYNTHETASE, CYTOPLASMIC.	**1.09**	0.01	2	0.474	**1.05**	0.02	2	0.597	**0.57**	0.00	2	
IPI00216952;IPI00644087	LMNA	ISOFORM C OF LAMIN-A/C.;PROGERIN.	**2.09**	0.33	30	0.000	**2.89**	0.47	28	0.001	**1.92**	0.33	30	0.039
IPI00217030	RPS4X	40S RIBOSOMAL PROTEIN S4, X ISOFORM.	**0.66**	0.06	9	0.028	**0.48**	0.10	10	0.090	**0.30**	0.08	9	0.118
IPI00217053;IPI00386206;IPI00744319;IPI00868973	PRRC1	ISOFORM 3 OF PROTEIN PRRC1.;PUTATIVE UNCHARACTERIZED PROTEIN PRRC1 (FRAGMENT).;ISOFORM 2 OF PROTEIN PRRC1.;ISOFORM 1 OF PROTEIN PRRC1.			2		**0.49**	0.00	2				2	
IPI00217143;IPI00305166	SDHA	57 KDA PROTEIN.;"CDNA FLJ61478, HIGHLY SIMILAR TO SUCCINATE DEHYDROGENASE (UBIQUINONE) FLAVOPROTEIN SUBUNIT, MITOCHONDRIAL."	**1.20**	0.07	3		**0.79**	0.13	3		**0.29**	0.00	3	
IPI00217465;IPI00217466;IPI00217467	HIST1H1C;HIST1H1D;HIST1H1E	HISTONE H1.2.;HISTONE H1.3.;HISTONE H1.4.	**1.16**	0.09	9	0.368	**1.31**	0.19	9	0.603	**0.90**	0.22	8	0.550
IPI00217468	HIST1H1B	HISTONE H1.5.	**1.32**	0.00	3		**1.30**	0.00	4		**2.22**	1.18	4	
IPI00217561;IPI00217562;IPI00217563;IPI00293305;IPI00549336	ITGB1	ISOFORM BETA-1C OF INTEGRIN BETA-1.;ISOFORM BETA-1C-2 OF INTEGRIN BETA-1.;ISOFORM BETA-1A OF INTEGRIN BETA-1.;ISOFORM BETA-1B OF INTEGRIN BETA-1.;ISOFORM BETA-1D OF INTEGRIN BETA-1.	**1.67**	0.00	2		**2.14**	0.00	2		**1.89**	0.00	2	
IPI00217683;IPI00237884;IPI00867627;IPI00940222	AKAP12	A KINASE (PRKA) ANCHOR PROTEIN 12 ISOFORM 2.;ISOFORM 1 OF A-KINASE ANCHOR PROTEIN 12.;ISOFORM 2 OF A-KINASE ANCHOR PROTEIN 12.;ISOFORM 3 OF A-KINASE ANCHOR PROTEIN 12.			1				2				2	
IPI00217906;IPI00748145;IPI00794986;IPI00924427;IPI00925504;IPI00925691;IPI00926935	GNAI2	ISOFORM 2 OF GUANINE NUCLEOTIDE-BINDING PROTEIN G(I), ALPHA-2 SUBUNIT.;"ISOFORM 1 OF GUANINE NUCLEOTIDE-BINDING PROTEIN G(I), ALPHA-2 SUBUNIT.";WUGSC:H_LUCA15.1 PROTEIN.;"CDNA FLJ61561, HIGHLY SIMILAR TO GUANINE NUCLEOTIDE-BINDING PROTEIN G(I), ALPHA-2 SUBUNIT.";"CDNA FLJ39276 FIS, CLONE OCBBF2010843, HIGHLY SIMILAR TO GUANINE NUCLEOTIDE-BINDING PROTEIN G(I), ALPHA-2 SUBUNIT.";"CDNA FLJ61190, HIGHLY SIMILAR TO GUANINE NUCLEOTIDE-BINDING PROTEIN G(I), ALPHA-2 SUBUNIT.";GALPHAI2 PROTEIN.							1				1	
IPI00217920;IPI00910572	ALDH16A1	ISOFORM 1 OF ALDEHYDE DEHYDROGENASE FAMILY 16 MEMBER A1.;"ALDEHYDE DEHYDROGENASE 16 FAMILY, MEMBER A1 ISOFORM 2."	**1.13**	0.02	2		**0.65**	0.00	2		**0.43**	0.00	2	
IPI00217952;IPI00299506	GFPT1	ISOFORM 1 OF GLUCOSAMINE--FRUCTOSE-6-PHOSPHATE AMINOTRANSFERASE [ISOMERIZING] 1.;ISOFORM 2 OF GLUCOSAMINE--FRUCTOSE-6-PHOSPHATE AMINOTRANSFERASE [ISOMERIZING] 1.	**0.74**	0.07	9	0.075	**0.68**	0.15	9				9	
IPI00217963;IPI00384444;IPI00654614;IPI00792454;IPI00794731	KRT16;KRT14;KRT13	KERATIN, TYPE I CYTOSKELETAL 16.;"KERATIN, TYPE I CYTOSKELETAL 14.";EPIDERMAL TYPE I KERATIN (FRAGMENT).;30 KDA PROTEIN.;15 KDA PROTEIN.									**4.92**	0.00	3	
IPI00217966;IPI00910754;IPI00947127	LDHA	ISOFORM 1 OF L-LACTATE DEHYDROGENASE A CHAIN.;LACTATE DEHYDROGENASE A ISOFORM 2.;LACTATE DEHYDROGENASE A ISOFORM 3.	**1.77**	0.08	6		**2.34**	0.04	5		**4.72**	0.33	7	
IPI00217975	LMNB1	LAMIN-B1.	**1.38**	0.16	6	0.306	**1.67**	0.32	5	0.493	**1.57**	0.24	5	0.425
IPI00218192;IPI00556036;IPI00791097;IPI00896413;IPI00896419;IPI00922043;IPI00944960	ITIH4	ISOFORM 2 OF INTER-ALPHA-TRYPSIN INHIBITOR HEAVY CHAIN H4.;16 KDA PROTEIN.;"CDNA FLJ53871, HIGHLY SIMILAR TO INTER-ALPHA-TRYPSIN INHIBITOR HEAVY CHAIN H4.";100 KDA PROTEIN.;ISOFORM 1 OF INTER-ALPHA-TRYPSIN INHIBITOR HEAVY CHAIN H4.;PROTEIN.;ITIH4 PROTEIN.			1				2				2	
IPI00218288;IPI00747830	SEC24D	ISOFORM 2 OF PROTEIN TRANSPORT PROTEIN SEC24D.;ISOFORM 1 OF PROTEIN TRANSPORT PROTEIN SEC24D.	**1.01**	0.00	1				1				1	
IPI00218319;IPI00218320;IPI00382894;IPI00477649;IPI00479185;IPI00642042;IPI00854592;IPI00909961	TPM3	ISOFORM 2 OF TROPOMYOSIN ALPHA-3 CHAIN.;ISOFORM 3 OF TROPOMYOSIN ALPHA-3 CHAIN.;"TROPOMYOSIN 3, ISOFORM CRA_B.";TROPOMYOSIN 3 ISOFORM 5.;TROPOMYOSIN 3 ISOFORM 4.;PUTATIVE UNCHARACTERIZED PROTEIN DKFZP686J1372.;"CDNA FLJ35393 FIS, CLONE SKNSH2000971, HIGHLY SIMILAR TO TROPOMYOSIN, CYTOSKELETAL TYPE.";"CDNA FLJ50720, HIGHLY SIMILAR TO HOMO SAPIENS TROPOMYOSIN 3 (TPM3), TRANSCRIPT VARIANT 2, MRNA."	**1.46**	0.20	5		**2.02**	0.40	6		**1.81**	0.36	5	
IPI00218342;IPI00794900	MTHFD1	C-1-TETRAHYDROFOLATE SYNTHASE, CYTOPLASMIC.;"CDNA FLJ56016, HIGHLY SIMILAR TO C-1-TETRAHYDROFOLATE SYNTHASE, CYTOPLASMIC."	**0.76**	0.00	2				2		**0.65**	0.00	2	
IPI00218414	CA2	CARBONIC ANHYDRASE 2.	**1.18**	0.06	4	0.469	**1.56**	0.07	4	0.518	**0.77**	0.00	3	0.394
IPI00218466;IPI00604476;IPI00792005;IPI00941172	SEC61A1	CDNA FLJ59739, HIGHLY SIMILAR TO PROTEIN TRANSPORT PROTEIN SEC61 SUBUNIT ALPHA ISOFORM 1.;ISOFORM 3 OF PROTEIN TRANSPORT PROTEIN SEC61 SUBUNIT ALPHA ISOFORM 1.;"CDNA FLJ14546 FIS, CLONE NT2RM2001613, HIGHLY SIMILAR TO PROTEIN TRANSPORT PROTEIN SEC61 SUBUNIT ALPHA ISOFORM 1.";ISOFORM 1 OF PROTEIN TRANSPORT PROTEIN SEC61 SUBUNIT ALPHA ISOFORM 1.	**0.84**	0.04	2		**0.92**	0.03	2				2	
IPI00218539;IPI00218540;IPI00295575;IPI00844199	COL11A1	ISOFORM B OF COLLAGEN ALPHA-1(XI) CHAIN.;ISOFORM C OF COLLAGEN ALPHA-1(XI) CHAIN.;ISOFORM A OF COLLAGEN ALPHA-1(XI) CHAIN.;"CDNA FLJ57066, HIGHLY SIMILAR TO COLLAGEN ALPHA-1(XI) CHAIN."			1				1				1	
IPI00218606;IPI00793332	RPS23	40S RIBOSOMAL PROTEIN S23.;16 KDA PROTEIN.	**0.75**	0.00	1				1		**0.36**	0.00	1	
IPI00218693;IPI00642457	APRT	ADENINE PHOSPHORIBOSYLTRANSFERASE.;ADENINE PHOSPHORIBOSYLTRANSFERASE ISOFORM B.	**0.93**	0.04	2	0.262	**0.82**	0.15	2	0.545	**0.49**	0.02	2	0.300
IPI00218725;IPI00479834;IPI00844276;IPI00873889;IPI00940615;IPI00942809	LAMA2	LAMININ ALPHA 2 SUBUNIT ISOFORM B PRECURSOR.;PUTATIVE UNCHARACTERIZED PROTEIN LAMA2.;LAMININ SUBUNIT ALPHA-2.;344 KDA PROTEIN.;343 KDA PROTEIN.			1				1				1	
IPI00218733	SOD1	SUPEROXIDE DISMUTASE [CU-ZN].	**1.04**	0.16	3	0.399	**1.49**	0.02	3		**1.35**	0.02	3	
IPI00218803;IPI00296534	FBLN1	ISOFORM B OF FIBULIN-1.;ISOFORM D OF FIBULIN-1.	**1.63**	0.25	4		**3.84**	0.12	6		**4.78**	0.43	6	
IPI00218820;IPI00220709;IPI00646748;IPI00926427	TPM2	ISOFORM 3 OF TROPOMYOSIN BETA CHAIN.;ISOFORM 2 OF TROPOMYOSIN BETA CHAIN.;TROPOMYOSIN 2.;TROPOMYOSIN 2 (BETA) ISOFORM 3.	**2.45**	0.53	10		**3.51**	0.71	8		**3.85**	0.45	6	
IPI00218847;IPI00219861;IPI00410615;IPI00935343;IPI00936586;IPI00952677	ACP1;LOC100288820;LOC100291559;LOC100290103	ISOFORM 2 OF LOW MOLECULAR WEIGHT PHOSPHOTYROSINE PROTEIN PHOSPHATASE.;ISOFORM 1 OF LOW MOLECULAR WEIGHT PHOSPHOTYROSINE PROTEIN PHOSPHATASE.;ISOFORM 3 OF LOW MOLECULAR WEIGHT PHOSPHOTYROSINE PROTEIN PHOSPHATASE.;HYPOTHETICAL PROTEIN XP_002342217.;HYPOTHETICAL PROTEIN XP_002347914.	**0.92**	0.00	1		**0.96**	0.00	1				1	
IPI00218850	SCAMP2	SECRETORY CARRIER-ASSOCIATED MEMBRANE PROTEIN 2.											1	
IPI00218896;IPI00473031;IPI00872991	ADH1A;ADH1B	ALCOHOL DEHYDROGENASE 1A.;ALCOHOL DEHYDROGENASE 1B.;"CDNA FLJ51682, HIGHLY SIMILAR TO ALCOHOL DEHYDROGENASE 1B."	**1.60**	0.11	5	0.089	**2.09**	0.12	7	0.147	**0.80**	0.07	6	0.685
IPI00218914	ALDH1A1	RETINAL DEHYDROGENASE 1.	**0.91**	0.07	20	0.073	**0.82**	0.11	19	0.038	**0.29**	0.09	19	0.005
IPI00218918	ANXA1	ANNEXIN A1.	**2.03**	0.26	7	0.092	**3.79**	0.41	6	0.064	**4.36**	0.31	11	0.043
IPI00219005	FKBP4	FK506-BINDING PROTEIN 4.			1				1				1	
IPI00219018;IPI00789134	GAPDH	GLYCERALDEHYDE-3-PHOSPHATE DEHYDROGENASE.;GLYCERALDEHYDE 3-PHOSPHATE DEHYDROGENASE.	**1.28**	0.10	9	0.186	**1.40**	0.04	9	0.379	**1.14**	0.09	10	0.526
IPI00219029;IPI00922421	GOT1	ASPARTATE AMINOTRANSFERASE, CYTOPLASMIC.;ASPARTATE AMINOTRANSFERASE.											1	
IPI00219067;IPI00639805;IPI00645360;IPI00940377	GSTM2	GLUTATHIONE S-TRANSFERASE MU 2.;26 KDA PROTEIN.;"CDNA FLJ60842, HIGHLY SIMILAR TO GLUTATHIONE S-TRANSFERASE MU 2.";GLUTATHIONE TRANSFERASE.			3		**1.63**	0.00	2		**1.04**	0.00	3	
IPI00219103;IPI00219344;IPI00395510	HPCA;HPCAL1;NCALD	NEURON-SPECIFIC CALCIUM-BINDING PROTEIN HIPPOCALCIN.;HIPPOCALCIN-LIKE PROTEIN 1.;NEUROCALCIN-DELTA.	**0.90**	0.00	1		**0.94**	0.00	1		**0.69**	0.00	1	
IPI00219111;IPI00790653;IPI00797490	TRAM1	TRANSLOCATING CHAIN-ASSOCIATED MEMBRANE PROTEIN 1.;TRAM1 PROTEIN (FRAGMENT).;33 KDA PROTEIN.	**1.01**	0.00	1				1				1	
IPI00219153;IPI00878392;IPI00879364	RPL22	60S RIBOSOMAL PROTEIN L22.;11 KDA PROTEIN.;9 KDA PROTEIN.	**0.85**	0.00	1		**0.44**	0.00	1		**0.34**	0.00	1	
IPI00219155	RPL27	60S RIBOSOMAL PROTEIN L27.	**0.49**	0.03	4	0.063	**0.57**	0.13	4		**0.20**	0.00	4	
IPI00219160	RPL34	60S RIBOSOMAL PROTEIN L34.			1				1				1	
IPI00219217;IPI00788938;IPI00789173	LDHB	L-LACTATE DEHYDROGENASE B CHAIN.;L-LACTATE DEHYDROGENASE.;15 KDA PROTEIN.	**1.05**	0.04	6	0.477	**0.60**	0.05	6		**0.35**	0.05	8	0.156
IPI00219219	LGALS1	GALECTIN-1.	**2.99**	0.00	1		**6.17**	0.00	2		**6.83**	0.00	3	
IPI00219365;IPI00872814	MSN	MOESIN.;PUTATIVE UNCHARACTERIZED PROTEIN MSN (FRAGMENT).	**1.31**	0.08	6	0.272	**1.67**	0.08	8	0.323	**1.67**	0.04	8	0.336
IPI00219436;IPI00643825;IPI00646627	SEC11C	SIGNAL PEPTIDASE COMPLEX CATALYTIC SUBUNIT SEC11C.;16 KDA PROTEIN.;"SEC11-LIKE 3 (S. CEREVISIAE), ISOFORM CRA_A."	**0.75**	0.16	2	0.247	**0.59**	0.07	2		**0.16**	0.00	2	
IPI00219446	PEBP1	PHOSPHATIDYLETHANOLAMINE-BINDING PROTEIN 1.	**0.79**	0.08	9	0.028	**0.67**	0.09	9	0.042	**0.18**	0.01	9	0.005
IPI00219525	PGD	6-PHOSPHOGLUCONATE DEHYDROGENASE, DECARBOXYLATING.	**0.98**	0.00	4		**1.15**	0.14	4		**1.09**	0.06	3	
IPI00219526	PGM1	ISOFORM 1 OF PHOSPHOGLUCOMUTASE-1.	**0.75**	0.09	7	0.075	**0.97**	0.09	7	0.410			6	

IPI00219622;IPI00927830	PSMA2	PROTEASOME SUBUNIT ALPHA TYPE-2.;19 KDA PROTEIN.	**0.91**	0.00	1		**0.79**	0.00	1		**0.69**	0.00	1	
IPI00219678	EIF2S1	EUKARYOTIC TRANSLATION INITIATION FACTOR 2 SUBUNIT 1.	**1.08**	0.00	1		**0.77**	0.00	1		**0.52**	0.00	1	
IPI00219685;IPI00909424;IPI00909465;IPI00942935;IPI00946421	YJEFN3;NDUFA13	26 KDA PROTEIN.;"CDNA FLJ57958, HIGHLY SIMILAR TO NADH DEHYDROGENASE (UBIQUINONE) 1 ALPHA SUBCOMPLEX SUBUNIT 13.";"CDNA FLJ58045, HIGHLY SIMILAR TO NADH DEHYDROGENASE (UBIQUINONE) 1 ALPHA SUBCOMPLEX SUBUNIT 13.";"CDNA FLJ59191, HIGHLY SIMILAR TO NADH DEHYDROGENASE (UBIQUINONE) 1 ALPHA SUBCOMPLEX SUBUNIT 13.";NADH DEHYDROGENASE [UBIQUINONE] 1 ALPHA SUBCOMPLEX SUBUNIT 13.											1	
IPI00219718;IPI00791403;IPI00940513;IPI00953392	RBP1	RETINOL BINDING PROTEIN 1, CELLULAR ISOFORM A.;"RETINOL BINDING PROTEIN 1, CELLULAR ISOFORM C.";RETINOL-BINDING PROTEIN 1.	**1.48**	0.11	3	0.374	**1.31**	0.14	3	0.566	**0.73**	0.00	3	
IPI00219757	GSTP1	GLUTATHIONE S-TRANSFERASE P.	**1.04**	0.06	3	0.502	**0.82**	0.05	3		**1.00**	0.08	4	0.646
IPI00219910;IPI00783862	BLVRB	22 KDA PROTEIN.;FLAVIN REDUCTASE.	**1.32**	0.05	2		**1.89**	0.15	2		**0.97**	0.09	2	
IPI00219953;IPI00908752;IPI00909419	CMPK1	UMP-CMP KINASE 1 ISOFORM A.;"CDNA FLJ53966, MODERATELY SIMILAR TO HOMO SAPIENS CYTIDYLATE KINASE (CMPK), MRNA.";UMP-CMP KINASE 1 ISOFORM B.	**0.70**	0.08	2		**0.68**	0.00	2		**0.58**	0.00	2	
IPI00220059;IPI00790949;IPI00815735	NDUFB4	NADH DEHYDROGENASE [UBIQUINONE] 1 BETA SUBCOMPLEX SUBUNIT 4.;8 KDA PROTEIN.;"NADH DEHYDROGENASE (UBIQUINONE) 1 BETA SUBCOMPLEX, 4, 15KDA."			1				1				1	
IPI00220113;IPI00333281;IPI00396171;IPI00411375;IPI00743873;IPI00878314;IPI00878651;IPI00888475;IPI00910596;IPI00924603;IPI00927173;IPI00927819	MAP4	ISOFORM 2 OF MICROTUBULE-ASSOCIATED PROTEIN 4.;MICROTUBULE-ASSOCIATED PROTEIN (FRAGMENT).;ISOFORM 1 OF MICROTUBULE-ASSOCIATED PROTEIN 4.;MICROTUBULE-ASSOCIATED PROTEIN 4 ISOFORM 2.;PUTATIVE UNCHARACTERIZED PROTEIN MAP4.;110 KDA PROTEIN.;33 KDA PROTEIN.;ISOFORM 6 OF MICROTUBULE-ASSOCIATED PROTEIN 4.;MICROTUBULE-ASSOCIATED PROTEIN.;47 KDA PROTEIN.;ISOFORM 5 OF MICROTUBULE-ASSOCIATED PROTEIN 4.	**1.36**	0.00	1		**1.89**	0.00	1		**1.76**	0.00	1	
IPI00220194;IPI00872375;IPI00909237	SLC2A1	SOLUTE CARRIER FAMILY 2, FACILITATED GLUCOSE TRANSPORTER MEMBER 1.;PUTATIVE UNCHARACTERIZED PROTEIN SLC2A1 (FRAGMENT).;"CDNA FLJ55703, HIGHLY SIMILAR TO SOLUTE CARRIER FAMILY 2, FACILITATED GLUCOSE TRANSPORTER MEMBER 1."	**1.33**	0.00	2		**1.77**	0.00	2		**1.09**	0.00	2	
IPI00220219	COPB2	COATOMER SUBUNIT BETA'.	**0.89**	0.00	3		**0.93**	0.00	3		**0.31**	0.00	3	
IPI00220271	AKR1A1	ALCOHOL DEHYDROGENASE [NADP+].	**0.80**	0.04	7	0.185	**0.67**	0.12	6	0.129	**0.47**	0.03	6	0.080
IPI00220301;IPI00910553	PRDX6	PEROXIREDOXIN-6.;"CDNA FLJ51310, MODERATELY SIMILAR TO PEROXIREDOXIN-6."	**0.82**	0.01	2	0.202	**0.69**	0.00	2	0.316	**0.35**	0.01	2	0.104
IPI00220327	KRT1	KERATIN, TYPE II CYTOSKELETAL 1.	**1.41**	0.22	11	0.367	**2.03**	0.32	12	0.159	**1.61**	0.33	11	0.578
IPI00220362;IPI00916763	HSPE1	10 KDA HEAT SHOCK PROTEIN, MITOCHONDRIAL.;PUTATIVE UNCHARACTERIZED PROTEIN HSPE1.	**1.72**	0.00	1	0.506	**1.07**	0.00	1		**1.26**	0.00	1	0.641
IPI00220365;IPI00384463;IPI00386533;IPI00479262;IPI00552639;IPI00759560;IPI00798400;IPI00924828;IPI00925057;IPI00925413;IPI00925434;IPI00926013;IPI00926411;IPI00926917;IPI00927510;IPI00927765	EIF4G1	87 KDA PROTEIN.;96 KDA PROTEIN.;"EUKARYOTIC TRANSLATION INITIATION FACTOR 4 GAMMA, 1 ISOFORM 4.";ISOFORM B OF EUKARYOTIC TRANSLATION INITIATION FACTOR 4 GAMMA 1.;ISOFORM 1 OF EUKARYOTIC TRANSLATION INITIATION FACTOR 4 GAMMA 1.;EIF4G1 PROTEIN.;"EUKARYOTIC TRANSLATION INITIATION FACTOR 4 GAMMA, 1 ISOFORM 3.";172 KDA PROTEIN.;89 KDA PROTEIN.;93 KDA PROTEIN.;98 KDA PROTEIN.;"EUKARYOTIC TRANSLATION INITIATION FACTOR 4 GAMMA, 1 ISOFORM 2.";82 KDA PROTEIN.;ISOFORM D OF EUKARYOTIC TRANSLATION INITIATION FACTOR 4 GAMMA 1.;ISOFORM E OF EUKARYOTIC TRANSLATION INITIATION FACTOR 4 GAMMA 1.	**0.71**	0.00	1		**0.58**	0.00	1		**0.38**	0.00	1	
IPI00220503;IPI00789792	DCTN2	DYNACTIN 2.;"DYNACTIN 2 (P50), ISOFORM CRA_B."	**1.12**	0.00	1		**1.26**	0.00	1		**0.57**	0.00	1	
IPI00220637;IPI00514587	SARS	SERYL-TRNA SYNTHETASE, CYTOPLASMIC.;SERYL-TRNA SYNTHETASE.	**1.22**	0.07	2		**1.56**	0.00	2				2	
IPI00220642;IPI00910779	YWHAG	14-3-3 PROTEIN GAMMA.;"CDNA FLJ52141, HIGHLY SIMILAR TO 14-3-3 PROTEIN GAMMA."				0.441	**0.82**	0.04	5	0.448	**1.36**	0.02	6	0.552
IPI00220644;IPI00941899	PKM2	ISOFORM M1 OF PYRUVATE KINASE ISOZYMES M1/M2.;66 KDA PROTEIN.				0.221				0.082	**2.18**	0.45	16	0.072
IPI00220706;IPI00554676;IPI00744503;IPI00749035;IPI00939160;IPI00939544	HBG2;HBG1;HBG2;HBG1	HEMOGLOBIN SUBUNIT GAMMA-1.;HEMOGLOBIN SUBUNIT GAMMA-2.;17 KDA PROTEIN.;GAMMA-G GLOBIN.;HEMOGLOBIN GAMMA-G (FRAGMENT).	**0.76**	0.04	3		**2.85**	1.64	5	0.578	**0.78**	0.18	3	0.680
IPI00220739	PGRMC1	MEMBRANE-ASSOCIATED PROGESTERONE RECEPTOR COMPONENT 1.			1				1					
IPI00220740;IPI00549248;IPI00658013	NPM1	ISOFORM 2 OF NUCLEOPHOSMIN.;ISOFORM 1 OF NUCLEOPHOSMIN.;NUCLEOPHOSMIN 1 ISOFORM 3.	**1.36**	0.14	5	0.210	**0.87**	0.01	6		**1.15**	0.06	6	
IPI00220741;IPI00641363	SPTA1	ISOFORM 1 OF SPECTRIN ALPHA CHAIN, ERYTHROCYTE.;"ISOFORM 2 OF SPECTRIN ALPHA CHAIN, ERYTHROCYTE."							1					
IPI00220766	GLO1	LACTOYLGLUTATHIONE LYASE.	**0.64**	0.00	1		**0.93**	0.00	1				1	
IPI00220834	XRCC5	ATP-DEPENDENT DNA HELICASE 2 SUBUNIT 2.			3		**0.59**	0.00	2				2	
IPI00220835;IPI00935805	SEC61B;LOC100287189;LOC100292167;LOC100290169	PROTEIN TRANSPORT PROTEIN SEC61 SUBUNIT BETA.;HYPOTHETICAL PROTEIN XP_002343101.	**0.90**	0.00	2		**0.90**	0.00	2		**0.34**	0.00	2	
IPI00220857;IPI00220859;IPI00302047;IPI00305750;IPI00413492;IPI00760715;IPI00760909;IPI00761035;IPI00761069;IPI00761140;IPI00761160;IPI00939354	CAST	ISOFORM 2 OF CALPASTATIN.;CALPASTATIN ISOFORM L.;"CDNA FLJ77737, HIGHLY SIMILAR TO HOMO SAPIENS CALPASTATIN (CAST), TRANSCRIPT VARIANT 3, MRNA.";ISOFORM 1 OF CALPASTATIN.;CALPASTATIN ISOFORM B.;ISOFORM 5 OF CALPASTATIN.;ISOFORM 4 OF CALPASTATIN.;ISOFORM 7 OF CALPASTATIN.;CALPASTATIN ISOFORM F.;CALPASTATIN ISOFORM H.;ISOFORM 6 OF CALPASTATIN.;CALPASTATIN ISOFORM C.							1				2	
IPI00221088	RPS9	40S RIBOSOMAL PROTEIN S9.	**0.72**	0.06	5	0.028	**0.59**	0.10	5	0.071	**0.39**	0.15	5	0.075
IPI00221089	RPS13	40S RIBOSOMAL PROTEIN S13.									**0.55**	0.00	1	
IPI00221092;IPI00397701	RPS16	40S RIBOSOMAL PROTEIN S16.;"CDNA FLJ56786, MODERATELY SIMILAR TO 40S RIBOSOMAL PROTEIN S16."	**0.84**	0.07	3	0.108	**0.53**	0.08	3		**0.28**	0.00	3	0.092
IPI00221093;IPI00797968	RPS17	40S RIBOSOMAL PROTEIN S17.;SIMILAR TO 40S RIBOSOMAL PROTEIN S17.	**1.04**	0.00	2				2				2	
IPI00221101	SI	SUCRASE-ISOMALTASE, INTESTINAL.			1									
IPI00221178;IPI00306825;IPI00399265;IPI00399266;IPI00399267;IPI00399268;IPI00743469	TPD52L2	ISOFORM 2 OF TUMOR PROTEIN D54.;ISOFORM 1 OF TUMOR PROTEIN D54.;TUMOR PROTEIN D52-LIKE 2 ISOFORM A.;ISOFORM 3 OF TUMOR PROTEIN D54.;TUMOR PROTEIN D52-LIKE 2 ISOFORM C.;TUMOR PROTEIN D52-LIKE 2 ISOFORM D.;PUTATIVE UNCHARACTERIZED PROTEIN TPD52L2 (FRAGMENT).	**0.56**	0.00	1				1		**0.98**	0.00	1	
IPI00221224	ANPEP	AMINOPEPTIDASE N.			1				1				1	
IPI00221226	ANXA6	ANNEXIN A6.	**1.37**	0.11	9	0.028	**1.97**	0.18	9	0.003	**1.28**	0.09	11	0.300
IPI00221232	GNG12	GUANINE NUCLEOTIDE-BINDING PROTEIN G(I)/G(S)/G(O) SUBUNIT GAMMA-12.							1					
IPI00221255;IPI00221259;IPI00336081;IPI00376019;IPI00550936;IPI00736561;IPI00790383;IPI00791032;IPI00791924;IPI00855853;IPI00943107	MYLK	ISOFORM 2 OF MYOSIN LIGHT CHAIN KINASE, SMOOTH MUSCLE.;"ISOFORM DEL-1790 OF MYOSIN LIGHT CHAIN KINASE, SMOOTH MUSCLE.";"ISOFORM 1 OF MYOSIN LIGHT CHAIN KINASE, SMOOTH MUSCLE.";"ISOFORM 3B OF MYOSIN LIGHT CHAIN KINASE, SMOOTH MUSCLE.";MYOSIN LIGHT CHAIN KINASE ISOFORM 7.;"ISOFORM 6 OF MYOSIN LIGHT CHAIN KINASE, SMOOTH MUSCLE.";203 KDA PROTEIN.;"ISOFORM 5 OF MYOSIN LIGHT CHAIN KINASE, SMOOTH MUSCLE.";"ISOFORM 4 OF MYOSIN LIGHT CHAIN KINASE, SMOOTH MUSCLE.";"ISOFORM 3A OF MYOSIN LIGHT CHAIN KINASE, SMOOTH MUSCLE.";210 KDA PROTEIN.			1								2	
IPI00240675;IPI00290110;IPI00940614	PDCD4	PROGRAMMED CELL DEATH 4 ISOFORM 2.;PROGRAMMED CELL DEATH PROTEIN 4.;PUTATIVE UNCHARACTERIZED PROTEIN PDCD4 (FRAGMENT).	**1.39**	0.03	2	0.281	**0.90**	0.00	2				2	
IPI00246058;IPI00938079	PDCD6IP	PROGRAMMED CELL DEATH 6-INTERACTING PROTEIN.;PROGRAMMED CELL DEATH 6 INTERACTING PROTEIN ISOFORM 2.	**0.98**	0.26	5	0.352	**1.81**	0.00	5		**1.19**	0.16	5	
IPI00246975;IPI00845493	GSTM3	GLUTATHIONE S-TRANSFERASE MU 3.;BRAIN TYPE MU-GLUTATHIONE S-TRANSFERASE.	**0.95**	0.17	4		**1.21**	0.16	5		**0.94**	0.00	5	
IPI00247583;IPI00397713;IPI00845507;IPI00879165;IPI00940766	RPL21;RPL21P19	60S RIBOSOMAL PROTEIN L21.;19 KDA PROTEIN.;SIMILAR TO 60S RIBOSOMAL PROTEIN L21.;PROTEIN.	**0.97**	0.00	1				1				1	
IPI00249672;IPI00642709;IPI00719783	CUZD1	ISOFORM 1 OF CUB AND ZONA PELLUCIDA-LIKE DOMAIN-CONTAINING PROTEIN 1.;ISOFORM 3 OF CUB AND ZONA PELLUCIDA-LIKE DOMAIN-CONTAINING PROTEIN 1.;ISOFORM 2 OF CUB AND ZONA PELLUCIDA-LIKE DOMAIN-CONTAINING PROTEIN 1.			1		**0.44**	0.00	1				1	
IPI00253036;IPI00642948	CD99	ISOFORM I OF CD99 ANTIGEN.;CD99 ANTIGEN ISOFORM B PRECURSOR.	**1.05**	0.00	1		**1.96**	0.00	1		**0.94**	0.00	1	
IPI00257508	DPYSL2	DIHYDROPYRIMIDINASE-RELATED PROTEIN 2.	**1.70**	0.06	5	0.186	**2.09**	0.21	6	0.186	**1.53**	0.20	5	0.253
IPI00257882;IPI00909399;IPI00910514	PEPD	XAA-PRO DIPEPTIDASE.;PROLIDASE ISOFORM 3.;PROLIDASE ISOFORM 2.	**0.91**	0.00	1		**1.15**	0.00	1		**0.51**	0.00	1	
IPI00260769;IPI00376503;IPI00550882;IPI00793698;IPI00902651;IPI00922289;IPI00930393;IPI00941557	SIRT7;PYCR1	PUTATIVE UNCHARACTERIZED PROTEIN.;PYRROLINE-5-CARBOXYLATE REDUCTASE 1 ISOFORM 2.;PYRROLINE-5-CARBOXYLATE REDUCTASE.;30 KDA PROTEIN.;28 KDA PROTEIN.;"CDNA FLJ56284, MODERATELY SIMILAR TO PYRROLINE-5-CARBOXYLATE REDUCTASE 1.";23 KDA PROTEIN.;"PYRROLINE-5-CARBOXYLATE REDUCTASE 1, MITOCHONDRIAL."			1				1				1	
IPI00289275;IPI00791803	CILP	CARTILAGE INTERMEDIATE LAYER PROTEIN 1.;SIMILAR TO CARTILAGE INTERMEDIATE LAYER PROTEIN.											1	
IPI00289334;IPI00382697;IPI00477536;IPI00900293;IPI00943563;IPI00953109	FLNB	ISOFORM 1 OF FILAMIN-B.;ISOFORM 3 OF FILAMIN-B.;FILAMIN B ISOFORM 3.;FILAMIN B ISOFORM 1.;FILAMIN B ISOFORM 4.	**1.18**	0.13	10	0.355	**1.17**	0.12	13	0.231	**2.46**	0.65	28	0.150

IPI00289499;IPI00925601;IPI00925871	ATIC	BIFUNCTIONAL PURINE BIOSYNTHESIS PROTEIN PURH.;"5-AMINOIMIDAZOLE-4-CARBOXAMIDE RIBONUCLEOTIDE FORMYLTRANSFERASE/IMP CYCLOHYDROLASE, ISOFORM CRA_G.";19 KDA PROTEIN.	**0.69**	0.00	1		**1.23**	0.00	2				2	
IPI00289501	VGF	NEUROSECRETORY PROTEIN VGF.			2				3					
IPI00289758;IPI00910745	CAPN2	CALPAIN-2 CATALYTIC SUBUNIT.;"CDNA FLJ58224, HIGHLY SIMILAR TO CALPAIN-2 CATALYTIC SUBUNIT."	**1.21**	0.00	3		**1.73**	0.00	2		**1.50**	0.00	3	
IPI00289862;IPI00657698;IPI00909070;IPI00923582	SCRN1	SECERNIN-1.;SECERNIN 1 ISOFORM C.;"CDNA FLJ51971, HIGHLY SIMILAR TO SECERNIN-1.";SECERNIN 1 ISOFORM B.	**0.84**	0.00	1				1				1	
IPI00290198	IL18	INTERLEUKIN-18.											1	
IPI00290315	CHGA	CHROMOGRANIN-A.			2		**2.64**	0.39	4	0.296			2	
IPI00290460	EIF3G	EUKARYOTIC TRANSLATION INITIATION FACTOR 3 SUBUNIT G.	**0.81**	0.00	1				1		**0.49**	0.00	1	
IPI00290566	TCP1	T-COMPLEX PROTEIN 1 SUBUNIT ALPHA.	**1.05**	0.00	1		**0.62**	0.00	1		**0.56**	0.00	1	
IPI00290928;IPI00922608	GNA13	GUANINE NUCLEOTIDE-BINDING PROTEIN SUBUNIT ALPHA-13.;"CDNA FLJ53108, HIGHLY SIMILAR TO GUANINE NUCLEOTIDE-BINDING PROTEIN ALPHA-13 SUBUNIT."	**1.04**	0.00	1		**1.47**	0.00	1		**0.96**	0.00	1	
IPI00291006;IPI00924593	MDH2	MALATE DEHYDROGENASE, MITOCHONDRIAL.;"CDNA FLJ52880, HIGHLY SIMILAR TO MALATE DEHYDROGENASE, MITOCHONDRIAL."	**0.74**	0.04	5		**0.62**	0.05	5	0.185	**0.50**	0.06	5	
IPI00291136	COL6A1	COLLAGEN ALPHA-1(VI) CHAIN.	**3.98**	0.66	13	0.000	**6.71**	0.67	16	0.001	**4.55**	1.07	15	0.001
IPI00291175;IPI00307162	VCL	ISOFORM 1 OF VINCULIN.;ISOFORM 2 OF VINCULIN.	**1.69**	0.22	13	0.025	**2.08**	0.21	15	0.030	**1.89**	0.26	16	0.087
IPI00291262;IPI00400826;IPI00795633	CLU	ISOFORM 1 OF CLUSTERIN.;ISOFORM 2 OF CLUSTERIN.;CLU.	**1.34**	0.27	4	0.246	**3.39**	0.24	4	0.037	**1.35**	0.06	4	0.519
IPI00291510;IPI00791628;IPI00795859;IPI00924999;IPI00925196	IMPDH2	INOSINE-5'-MONOPHOSPHATE DEHYDROGENASE 2.;INOSINE MONOPHOSPHATE DEHYDROGENASE TYPE II.;IMP DEHYDROGENASE 2.;31 KDA PROTEIN.;PROTEIN.							1					
IPI00291866;IPI00556459;IPI00879931	SERPING1	PLASMA PROTEASE C1 INHIBITOR.;SERINE/CYSTEINE PROTEINASE INHIBITOR CLADE G MEMBER 1 SPLICE VARIANT 2 (FRAGMENT).;"CDNA FLJ58826, HIGHLY SIMILAR TO PLASMA PROTEASE C1 INHIBITOR."	**3.34**	0.00	1		**2.64**	0.00	1		**3.87**	0.00	2	
IPI00292020	SRM	SPERMIDINE SYNTHASE.			1				1				1	
IPI00292071	SCG3	SECRETOGRANIN-3.			1				1					
IPI00292130	DPT	DERMATOPONTIN.	**3.01**	0.00	1		**6.08**	0.00	1		**4.45**	0.00	1	
IPI00292530;IPI00945924	ITIH1	INTER-ALPHA-TRYPSIN INHIBITOR HEAVY CHAIN H1.;23 KDA PROTEIN.	**1.95**	0.00	2		**3.21**	0.00	4		**7.71**	0.00	2	
IPI00292532	CAMP	CATHELICIDIN ANTIMICROBIAL PEPTIDE PRECURSOR.	**0.47**	0.00	1		**1.73**	0.00	1		**0.94**	0.00	1	
IPI00292695	ACADL	LONG-CHAIN SPECIFIC ACYL-COA DEHYDROGENASE, MITOCHONDRIAL.			1									
IPI00292858;IPI00852987;IPI00853163	TYMP	THYMIDINE PHOSPHORYLASE.;"CDNA, FLJ95575, HIGHLY SIMILAR TO HOMO SAPIENS ENDOTHELIAL CELL GROWTH FACTOR 1 (PLATELET-DERIVED) (ECGF1), MRNA.";46 KDA PROTEIN.	**1.28**	0.00	2		**2.80**	0.20	2		**2.82**	0.94	3	
IPI00293088;IPI00883772	GAA	LYSOSOMAL ALPHA-GLUCOSIDASE.;ACID ALPHA-GLUCOSIDASE PREPROPROTEIN.			2				1				1	
IPI00293303;IPI00384155;IPI00384156	LGMN	LEGUMAIN.;FULL-LENGTH CDNA CLONE CS0DI002YH20 OF PLACENTA OF HOMO SAPIENS.;FULL-LENGTH CDNA CLONE CS0DB001YK19 OF NEUROBLASTOMA OF HOMO SAPIENS.							1				1	
IPI00293616;IPI00908506	DDX3Y	ATP-DEPENDENT RNA HELICASE DDX3Y.;"CDNA FLJ53122, HIGHLY SIMILAR TO ATP-DEPENDENT RNA HELICASE DDX3Y."			2		**1.34**	0.06	2				2	
IPI00293655	DDX1	ATP-DEPENDENT RNA HELICASE DDX1.	**0.73**	0.00	1		**1.04**	0.00	1				1	
IPI00293721	AKR7A3	AFLATOXIN B1 ALDEHYDE REDUCTASE MEMBER 3.	**0.47**	0.00	1				1		**0.22**	0.00	1	
IPI00293867;IPI00878984	DDT	D-DOPACHROME DECARBOXYLASE.;14 KDA PROTEIN.	**0.49**	0.01	2	0.176	**0.44**	0.01	2	0.148	**0.19**	0.01	2	0.200
IPI00294380;IPI00797038	PCK2	PHOSPHOENOLPYRUVATE CARBOXYKINASE [GTP], MITOCHONDRIAL.;MITOCHONDRIAL PHOSPHOENOLPYRUVATE CARBOXYKINASE 2 ISOFORM 1 PRECURSOR.	**0.98**	0.11	6	0.349	**0.93**	0.15	6	0.450	**0.71**	0.18	6	
IPI00294395	C8B	COMPLEMENT COMPONENT C8 BETA CHAIN.							1				1	
IPI00294398;IPI00298406;IPI00902588	HADH	ISOFORM 1 OF HYDROXYACYL-COENZYME A DEHYDROGENASE, MITOCHONDRIAL.;"ISOFORM 2 OF HYDROXYACYL-COENZYME A DEHYDROGENASE, MITOCHONDRIAL.";"CDNA FLJ38699 FIS, CLONE KIDNE2002168, HIGHLY SIMILAR TO SHORT CHAIN 3-HYDROXYACYL-COA DEHYDROGENASE, MITOCHONDRIAL."	**0.86**	0.00	1		**0.57**	0.00	1		**0.38**	0.00	1	
IPI00294578	TGM2	ISOFORM 1 OF PROTEIN-GLUTAMINE GAMMA-GLUTAMYLTRANSFERASE 2.	**2.37**	0.36	6	0.064	**4.51**	0.31	12	0.203	**4.36**	0.28	12	0.029
IPI00294739;IPI00943982	SAMHD1	ISOFORM 1 OF SAM DOMAIN AND HD DOMAIN-CONTAINING PROTEIN 1.;ISOFORM 2 OF SAM DOMAIN AND HD DOMAIN-CONTAINING PROTEIN 1.	**0.63**	0.00	2		**0.83**	0.00	3		**0.68**	0.00	3	
IPI00294834;IPI00944640	ASPH	ASPARTYL/ASPARAGINYL BETA-HYDROXYLASE.;ASPARTATE BETA-HYDROXYLASE ISOFORM F.											4	
IPI00294911	SDHB	SUCCINATE DEHYDROGENASE [UBIQUINONE] IRON-SULFUR SUBUNIT, MITOCHONDRIAL.									**0.74**	0.00	1	
IPI00295098	SRPRB	SIGNAL RECOGNITION PARTICLE RECEPTOR SUBUNIT BETA.	**0.76**	0.09	3	0.185	**0.76**	0.00	3				3	
IPI00295386	CBR1	CARBONYL REDUCTASE [NADPH] 1.	**1.76**	0.14	5	0.267	**1.47**	0.06	4	0.413	**1.16**	0.04	4	0.681
IPI00295400;IPI00412737	WARS	ISOFORM 1 OF TRYPTOPHANYL-TRNA SYNTHETASE, CYTOPLASMIC.;TRYPTOPHANYL-TRNA SYNTHETASE ISOFORM B.	**0.80**	0.00	1		**0.75**	0.00	1		**0.67**	0.00	1	
IPI00295414	COL15A1	COLLAGEN ALPHA-1(XV) CHAIN.	**1.49**	0.09	3		**2.65**	0.26	4		**1.45**	0.02	4	
IPI00295542;IPI00892540;IPI00892657;IPI00893190	NUCB1	NUCLEOBINDIN-1.;PROTEIN.;"CDNA FLJ52898, HIGHLY SIMILAR TO NUCLEOBINDIN-1."			1				1				1	
IPI00295663	CELA3A	CHYMOTRYPSIN-LIKE ELASTASE FAMILY MEMBER 3A.	**0.63**	0.12	14	0.031	**0.28**	0.09	14	0.049	**0.04**	0.01	14	0.282
IPI00295741;IPI00788996;IPI00909303	CTSB	CATHEPSIN B.;20 KDA PROTEIN.;"CDNA FLJ58073, MODERATELY SIMILAR TO CATHEPSIN B."	**1.26**	0.00	1		**2.19**	0.00	1		**3.33**	0.00	1	
IPI00295857;IPI00646493	COPA	ISOFORM 1 OF COATOMER SUBUNIT ALPHA.;ISOFORM 2 OF COATOMER SUBUNIT ALPHA.	**0.94**	0.07	5	0.338	**0.94**	0.03	5	0.461	**0.45**	0.04	5	0.270
IPI00295992;IPI00455487;IPI00513768;IPI00643435;IPI00646144;IPI00872864	ATAD3A;ATAD3C	ISOFORM 2 OF ATPASE FAMILY AAA DOMAIN-CONTAINING PROTEIN 3A.;"ATPASE FAMILY, AAA DOMAIN CONTAINING 3A.";ATPASE FAMILY AAA DOMAIN-CONTAINING PROTEIN 3C.;ISOFORM 1 OF ATPASE FAMILY AAA DOMAIN-CONTAINING PROTEIN 3A.;"CDNA FLJ31537 FIS, CLONE NT2RI2000704, HIGHLY SIMILAR TO ATPASE FAMILY AAA DOMAIN-CONTAINING PROTEIN 3A.";PUTATIVE UNCHARACTERIZED PROTEIN ENSP00000383631.	**0.58**	0.00	2		**0.41**	0.00	1				1	
IPI00296053;IPI00759715	FH	ISOFORM MITOCHONDRIAL OF FUMARATE HYDRATASE, MITOCHONDRIAL.;"ISOFORM CYTOPLASMIC OF FUMARATE HYDRATASE, MITOCHONDRIAL."	**1.08**	0.06	2		**0.99**	0.00	2		**0.33**	0.00	2	
IPI00296099	THBS1	THROMBOSPONDIN-1.			1				1				4	
IPI00296147;IPI00909406	TMEM119	TRANSMEMBRANE PROTEIN 119.;CDNA FLJ54181.							2				2	
IPI00296190;IPI00646289;IPI00646889;IPI00939991	C10orf58;C10orf57	UNCHARACTERIZED PROTEIN C10ORF58.;25 KDA PROTEIN.;PUTATIVE UNCHARACTERIZED PROTEIN C10ORF58.;"CDNA, FLJ96497."	**1.41**	0.00	1				1		**0.51**	0.00	1	
IPI00296337;IPI00376215;IPI00786995	PRKDC;LOC731751	ISOFORM 1 OF DNA-DEPENDENT PROTEIN KINASE CATALYTIC SUBUNIT.;ISOFORM 2 OF DNA-DEPENDENT PROTEIN KINASE CATALYTIC SUBUNIT.;"SIMILAR TO PROTEIN KINASE, DNA-ACTIVATED, CATALYTIC POLYPEPTIDE."	**1.46**	0.07	3		**1.27**	0.17	2		**1.29**	0.00	1	
IPI00296526;IPI00939575	NAGK	N-ACETYLGLUCOSAMINE KINASE.;N-ACETYL-D-GLUCOSAMINE KINASE.	**1.48**	0.00	1				1		**1.02**	0.00	1	
IPI00296537;IPI00889740	FBLN1	ISOFORM C OF FIBULIN-1.;FIBULIN 1.	**2.44**	0.00	3		**4.29**	0.00	4		**4.57**	0.00	5	
IPI00296909	PARP4	POLY [ADP-RIBOSE] POLYMERASE 4.											1	
IPI00296922;IPI00743203	LAMB2	LAMININ SUBUNIT BETA-2.;SIMILAR TO S-LAMININ.	**1.32**	0.14	6	0.202	**1.55**	0.18	7	0.308	**1.16**	0.04	5	0.666
IPI00296930;IPI00941312	CPA2	CARBOXYPEPTIDASE A2 (PANCREATIC) PRECURSOR.;CARBOXYPEPTIDASE A2.	**0.73**	0.10	19	0.028	**0.27**	0.04	17	0.003	**0.04**	0.00	17	
IPI00297084;IPI00910419	DDOST	DOLICHYL-DIPHOSPHOOLIGOSACCHARIDE--PROTEIN GLYCOSYLTRANSFERASE 48 KDA SUBUNIT.;"CDNA FLJ52929, HIGHLY SIMILAR TO DOLICHYL-DIPHOSPHOOLIGOSACCHARIDE--PROTEINGLYCOSYLTRANSFERASE 48 KDA SUBUNIT."	**0.75**	0.04	3		**0.35**	0.00	2		**0.16**	0.00	2	
IPI00297160;IPI00305064;IPI00418465;IPI00419219;IPI00827555;IPI00827650;IPI00827658;IPI00827795;IPI00827893;IPI00827937;IPI00827982;IPI00828056;IPI00828064;IPI00828108;IPI00828117;IPI00828192;IPI00854669;IPI00918020;IPI00922478	CD44	ISOFORM 12 OF CD44 ANTIGEN.;ISOFORM CD44 OF CD44 ANTIGEN.;ISOFORM 4 OF CD44 ANTIGEN.;ISOFORM 10 OF CD44 ANTIGEN.;ISOFORM 9 OF CD44 ANTIGEN.;ISOFORM 3 OF CD44 ANTIGEN.;ISOFORM 7 OF CD44 ANTIGEN.;ISOFORM 8 OF CD44 ANTIGEN.;ISOFORM 11 OF CD44 ANTIGEN.;ISOFORM 14 OF CD44 ANTIGEN.;ISOFORM 13 OF CD44 ANTIGEN.;ISOFORM 16 OF CD44 ANTIGEN.;ISOFORM 6 OF CD44 ANTIGEN.;ISOFORM 15 OF CD44 ANTIGEN.;ISOFORM 5 OF CD44 ANTIGEN.;ISOFORM 17 OF CD44 ANTIGEN.;PUTATIVE UNCHARACTERIZED PROTEIN CD44.;"CDNA FLJ52702, HIGHLY SIMILAR TO HOMO SAPIENS CD44 ANTIGEN (HOMING FUNCTION AND INDIAN BLOOD GROUP SYSTEM) (CD44), TRANSCRIPT VARIANT 4, MRNA.";CD44.	**1.03**	0.00	1		**1.25**	0.00	1		**1.06**	0.00	1	
IPI00297492	STT3A	DOLICHYL-DIPHOSPHOOLIGOSACCHARIDE--PROTEIN GLYCOSYLTRANSFERASE SUBUNIT STT3A.	**0.78**	0.03	2	0.105	**0.66**	0.00	2	0.090	**0.29**	0.00	2	
IPI00297646	COL1A1	COLLAGEN ALPHA-1(I) CHAIN.	**6.85**	1.26	15	0.025	**13.48**	2.76	15	0.087	**9.54**	2.53	15	0.036
IPI00297779	CCT2	T-COMPLEX PROTEIN 1 SUBUNIT BETA.	**1.22**	0.10	3	0.506	**0.96**	0.00	3		**0.49**	0.00	3	0.340
IPI00297910	TACSTD2	TUMOR-ASSOCIATED CALCIUM SIGNAL TRANSDUCER 2.											1	
IPI00297982	EIF2S3	EUKARYOTIC TRANSLATION INITIATION FACTOR 2 SUBUNIT 3.			1				1				1	

IPI00298057	PPL	PERIPLAKIN.											2	
IPI00298237;IPI00554538;IPI00554617;IPI00909516;IPI00922058;IPI00939667	TPP1	CDNA FLJ56402, HIGHLY SIMILAR TO TRIPEPTIDYL-PEPTIDASE 1.;60 KDA PROTEIN.;"CDNA FLJ57277, HIGHLY SIMILAR TO TRIPEPTIDYL-PEPTIDASE 1.";"CDNA FLJ58558, HIGHLY SIMILAR TO TRIPEPTIDYL-PEPTIDASE 1.";"CDNA FLJ59472, HIGHLY SIMILAR TO TRIPEPTIDYL-PEPTIDASE 1.";ISOFORM 1 OF TRIPEPTIDYL-PEPTIDASE 1.	**1.51**	0.00	3		**1.62**	0.00	3		**0.88**	0.00	3	
IPI00298281	LAMC1	LAMININ SUBUNIT GAMMA-1.	**1.75**	0.15	7	0.013	**2.04**	0.18	8	0.046	**1.03**	0.06	7	0.623
IPI00298308	ALDH1L2	ISOFORM 1 OF PROBABLE 10-FORMYLTETRAHYDROFOLATE DEHYDROGENASE ALDH1L2.	**0.70**	0.04	7	0.129	**0.87**	0.12	7		**0.19**	0.00	7	
IPI00298497	FGB	FIBRINOGEN BETA CHAIN.	**1.63**	0.29	13	0.337	**2.77**	0.45	14	0.155	**2.32**	0.41	13	0.102
IPI00298520;IPI00514053	ARCN1	COATOMER SUBUNIT DELTA VARIANT 2.;COATOMER SUBUNIT DELTA.	**0.98**	0.04	3	0.347	**0.82**	0.08	3	0.312	**0.58**	0.07	3	0.452
IPI00298547	PARK7	PROTEIN DJ-1.	**1.10**	0.07	6	0.426	**0.98**	0.05	6	0.333	**0.45**	0.00	6	0.150
IPI00298828;IPI00910625	APOH	BETA-2-GLYCOPROTEIN 1.;"CDNA FLJ51265, MODERATELY SIMILAR TO BETA-2-GLYCOPROTEIN 1."	**3.17**	0.00	1		**3.88**	0.00	1		**2.80**	0.00	1	
IPI00298860;IPI00789477;IPI00790675;IPI00903112;IPI00908908;IPI00925299;IPI00925519;IPI00925547	LTF	CDNA FLJ78440, HIGHLY SIMILAR TO HUMAN LACTOFERRIN.;"CDNA FLJ58679, HIGHLY SIMILAR TO LACTOTRANSFERRIN.";LACTOFERRIN (FRAGMENT).;"CDNA FLJ36533 FIS, CLONE TRACH2004428, HIGHLY SIMILAR TO LACTOTRANSFERRIN (FRAGMENT).";"CDNA FLJ59602, HIGHLY SIMILAR TO LACTOTRANSFERRIN.";20 KDA PROTEIN.;13 KDA PROTEIN.;LACTOFERRIN.	**0.72**	0.00	2		**4.80**	1.35	5		**0.75**	0.00	5	
IPI00298961;IPI00893013	XPO1	EXPORTIN-1.;"CDNA FLJ55480, HIGHLY SIMILAR TO EXPORTIN-1."			1		**2.12**	0.00	1				1	
IPI00298971	VTN	VITRONECTIN.			1				2				1	
IPI00298994	TLN1	TALIN-1.	**1.67**	0.32	16	0.025	**2.69**	0.56	19	0.017	**1.82**	0.36	18	0.014
IPI00299000;IPI00794875;IPI00807557	PA2G4	PROLIFERATION-ASSOCIATED PROTEIN 2G4.;41 KDA PROTEIN.;PA2G4 PROTEIN (FRAGMENT).	**1.04**	0.03	2		**0.83**	0.00	2		**0.57**	0.00	2	
IPI00299145;IPI00300725;IPI00909059;IPI00930073;IPI00942257	KRT6C;KRT6A	KERATIN, TYPE II CYTOSKELETAL 6C.;"KERATIN, TYPE II CYTOSKELETAL 6A.";"CDNA FLJ53910, HIGHLY SIMILAR TO KERATIN, TYPE II CYTOSKELETAL 6A.";"CDNA, FLJ93744, HIGHLY SIMILAR TO HOMO SAPIENS KERATIN 6E (KRT6E), MRNA.";58 KDA PROTEIN.				0.516				0.463	**1.22**	0.24	8	0.638
IPI00299155;IPI00789638;IPI00790207;IPI00791083;IPI00795606	PSMA4	PROTEASOME SUBUNIT ALPHA TYPE-4.;PROTEASOME SUBUNIT BETA TYPE.;27 KDA PROTEIN.;20 KDA PROTEIN.;26 KDA PROTEIN.	**0.97**	0.00	1		**1.30**	0.00	1		**0.92**	0.00	1	
IPI00299301;IPI00299302;IPI00921956;IPI00952899	SYNM	ISOFORM 1 OF SYNEMIN.;ISOFORM 2 OF SYNEMIN.;ISOFORM 3 OF SYNEMIN.			2								2	
IPI00299429;IPI00470823;IPI00914914;IPI00914943	GP2	CDNA FLJ56017, HIGHLY SIMILAR TO PANCREATIC SECRETORY GRANULE MEMBRANE MAJOR GLYCOPROTEIN GP2.;ISOFORM 1 OF PANCREATIC SECRETORY GRANULE MEMBRANE MAJOR GLYCOPROTEIN GP2.;ISOFORM 2 OF PANCREATIC SECRETORY GRANULE MEMBRANE MAJOR GLYCOPROTEIN GP2.;ISOFORM ALPHA OF PANCREATIC SECRETORY GRANULE MEMBRANE MAJOR GLYCOPROTEIN GP2.	**0.46**	0.09	8		**0.16**	0.08	8	0.022	**0.04**	0.00	8	
IPI00299547;IPI00643623	LCN2	NEUTROPHIL GELATINASE-ASSOCIATED LIPOCALIN.;PUTATIVE UNCHARACTERIZED PROTEIN LCN2.											2	
IPI00299571;IPI00644989	PDIA6	ISOFORM 2 OF PROTEIN DISULFIDE-ISOMERASE A6.;ISOFORM 1 OF PROTEIN DISULFIDE-ISOMERASE A6.	**0.88**	0.13	21	0.031	**0.45**	0.10	22	0.004	**0.20**	0.05	21	0.008
IPI00299573;IPI00397676	RPL7A	60S RIBOSOMAL PROTEIN L7A.;PUTATIVE UNCHARACTERIZED PROTEIN ENSP00000372524.	**0.74**	0.09	7	0.253	**0.67**	0.07	7	0.206	**0.27**	0.02	7	
IPI00299738;IPI00908730	PCOLCE	PROCOLLAGEN C-ENDOPEPTIDASE ENHANCER 1.;"CDNA FLJ55126, HIGHLY SIMILAR TO PROCOLLAGEN C-ENDOPEPTIDASE ENHANCER 1."							2					
IPI00300026;IPI00300027;IPI00640414;IPI00647709;IPI00782941	SULT1A1;SULT1A2	SULFOTRANSFERASE 1A1.;SULFOTRANSFERASE 1A2.;29 KDA PROTEIN.;24 KDA PROTEIN.;"SULFOTRANSFERASE FAMILY, CYTOSOLIC, 1A, PHENOL-PREFERRING, MEMBER 2."	**1.34**	0.00	1		**2.30**	0.00	1		**0.80**	0.00	1	
IPI00300074	FARSB	PHENYLALANYL-TRNA SYNTHETASE BETA CHAIN.	**0.86**	0.00	2	0.246	**0.71**	0.00	1	0.148			1	
IPI00300371	SF3B3	ISOFORM 1 OF SPLICING FACTOR 3B SUBUNIT 3.							1					
IPI00300567;IPI00398758	DCI	ISOFORM 1 OF 3,2-TRANS-ENOYL-COA ISOMERASE, MITOCHONDRIAL.;"ISOFORM 2 OF 3,2-TRANS-ENOYL-COA ISOMERASE, MITOCHONDRIAL."	**0.81**	0.00	1				1		**0.59**	0.00	1	
IPI00301021;IPI00449669;IPI00945633;IPI00945795;IPI00946676;IPI00946839	SSR1	ISOFORM 1 OF TRANSLOCON-ASSOCIATED PROTEIN SUBUNIT ALPHA.;ISOFORM 2 OF TRANSLOCON-ASSOCIATED PROTEIN SUBUNIT ALPHA.;34 KDA PROTEIN.;30 KDA PROTEIN.;"CDNA, FLJ93042, HIGHLY SIMILAR TO HOMO SAPIENS SIGNAL SEQUENCE RECEPTOR, ALPHA (TRANSLOCON-ASSOCIATED PROTEIN ALPHA) (SSR1), MRNA."	**0.76**	0.00	2	0.324	**0.34**	0.00	2		**0.15**	0.00	2	
IPI00301058	VASP	VASODILATOR-STIMULATED PHOSPHOPROTEIN.	**0.92**	0.00	1				1				1	
IPI00301109;IPI00413014;IPI00470502;IPI00470503;IPI00654717;IPI00749469;IPI00910509	PPA2	ISOFORM 1 OF INORGANIC PYROPHOSPHATASE 2, MITOCHONDRIAL.;INORGANIC PYROPHOSPHATASE 2 ISOFORM 3 PRECURSOR.;"ISOFORM 2 OF INORGANIC PYROPHOSPHATASE 2, MITOCHONDRIAL.";"ISOFORM 3 OF INORGANIC PYROPHOSPHATASE 2, MITOCHONDRIAL.";INORGANIC PYROPHOSPHATASE 2 ISOFORM 5 PRECURSOR.;25 KDA PROTEIN.;"CDNA FLJ59863, HIGHLY SIMILAR TO INORGANIC PYROPHOSPHATASE 2, MITOCHONDRIAL."			1				1				1	
IPI00301185;IPI00306719;IPI00940998	CHID1	ISOFORM 2 OF CHITINASE DOMAIN-CONTAINING PROTEIN 1.;CDNA FLJ55809.;ISOFORM 1 OF CHITINASE DOMAIN-CONTAINING PROTEIN 1.	**1.01**	0.09	3		**0.37**	0.00	3				3	
IPI00301280;IPI00383085;IPI00926007	TMEM43	TRANSMEMBRANE PROTEIN 43.;23 KDA PROTEIN.;FLJ00144 PROTEIN.							1					
IPI00301364	SKP1	ISOFORM 1 OF S-PHASE KINASE-ASSOCIATED PROTEIN 1.	**1.18**	0.00	1		**0.97**	0.00	1				1	
IPI00301503;IPI00472633;IPI00555647;IPI00793887;IPI00927262;IPI00927271;IPI00927500	TRA2B	ISOFORM 1 OF TRANSFORMER-2 PROTEIN HOMOLOG BETA.;"CDNA FLJ40872 FIS, CLONE TUTER2000283, HIGHLY SIMILAR TO HOMO SAPIENS TRANSFORMER-2-BETA (SFRS10) GENE.";"SPLICING FACTOR, ARGININE/SERINE-RICH 10 (TRANSFORMER 2 HOMOLOG, DROSOPHILA) VARIANT.";13 KDA PROTEIN.;PROTEIN.;ISOFORM 3 OF TRANSFORMER-2 PROTEIN HOMOLOG BETA.	**1.14**	0.00	1		**0.98**	0.00	1		**0.97**	0.00	1	
IPI00301936;IPI00940851	ELAVL1	CDNA FLJ60076, HIGHLY SIMILAR TO ELAV-LIKE PROTEIN 1.;ELAV-LIKE PROTEIN 1.			1				1				1	
IPI00301961	PCSK1	NEUROENDOCRINE CONVERTASE 1.							1					
IPI00302592;IPI00333541	FLNA	ISOFORM 2 OF FILAMIN-A.;ISOFORM 1 OF FILAMIN-A.	**2.65**	0.45	53	0.000	**3.87**	0.99	53	0.000	**3.91**	1.14	70	0.000
IPI00302850	SNRPD1	SMALL NUCLEAR RIBONUCLEOPROTEIN SM D1.	**1.29**	0.00	1				1				1	
IPI00302925;IPI00784090	CCT8	59 KDA PROTEIN.;T-COMPLEX PROTEIN 1 SUBUNIT THETA.	**1.22**	0.19	4		**0.94**	0.00	4		**1.06**	0.00	4	
IPI00302927;IPI00893358;IPI00921414	ILK-2;CCT4	T-COMPLEX PROTEIN 1 SUBUNIT DELTA.;"T-COMPLEX PROTEIN 1, DELTA SUBUNIT.";CHAPERONIN CPN60/TCP-1 FAMILY PROTEIN.			1		**1.02**	0.00	1		**0.62**	0.00	1	
IPI00302944;IPI00329573	COL12A1	ISOFORM 4 OF COLLAGEN ALPHA-1(XII) CHAIN.;ISOFORM 1 OF COLLAGEN ALPHA-1(XII) CHAIN.							4				29	
IPI00303158;IPI00479958	CMAS	ISOFORM 1 OF N-ACYLNEURAMINATE CYTIDYLYLTRANSFERASE.;ISOFORM 2 OF N-ACYLNEURAMINATE CYTIDYLYLTRANSFERASE.	**0.74**	0.00	1				1				1	
IPI00303207	ABCE1	ATP-BINDING CASSETTE SUB-FAMILY E MEMBER 1.	**1.23**	0.00	1				1				1	
IPI00303258;IPI00910942;IPI00926221;IPI00926626;IPI00927828	LMCD1	LIM AND CYSTEINE-RICH DOMAINS PROTEIN 1.;"CDNA FLJ52480, HIGHLY SIMILAR TO LIM AND CYSTEINE-RICH DOMAINS PROTEIN 1.";"CDNA FLJ55018, HIGHLY SIMILAR TO LIM AND CYSTEINE RICH DOMAINS PROTEIN 1.";26 KDA PROTEIN.;23 KDA PROTEIN.											1	
IPI00303476	ATP5B	ATP SYNTHASE SUBUNIT BETA, MITOCHONDRIAL.	**1.02**	0.10	18	0.452	**0.78**	0.10	18	0.088	**0.45**	0.05	16	0.033
IPI00303568;IPI00395565;IPI00514138;IPI00947368	PTGES2	PROSTAGLANDIN E SYNTHASE 2.;PUTATIVE UNCHARACTERIZED PROTEIN PTGES2.;29 KDA PROTEIN.;PROTEIN.											1	
IPI00304273;IPI00847179	APOA4	APOLIPOPROTEIN A-IV.;APOLIPOPROTEIN A-IV PRECURSOR.			2		**1.01**	0.00	6		**0.90**	0.00	6	
IPI00304612;IPI00398949;IPI00398983;IPI00432865;IPI00936705	RPL13A;LOC100287887;RPL13AP25;RPL13AP3;LOC100293339;LOC100293761	60S RIBOSOMAL PROTEIN L13A.;23 KDA PROTEIN.;NOVEL PROTEIN SIMILAR TO RIBOSOMAL PROTEIN L13A RPL13A.;PUTATIVE 60S RIBOSOMAL PROTEIN L13A-LIKE MGC87657.;SIMILAR TO RIBOSOMAL PROTEIN L13A.	**0.69**	0.00	2		**0.32**	0.00	1		**0.18**	0.00	1	
IPI00304840;IPI00828201	COL6A2	ISOFORM 2C2 OF COLLAGEN ALPHA-2(VI) CHAIN.;COL6A2 PROTEIN.	**6.18**	1.17	7		**7.23**	0.63	6		**6.01**	0.33	7	
IPI00304925;IPI00845339;IPI00911039	HSPA1B;HSPA1A	HEAT SHOCK 70 KDA PROTEIN 1.;"CDNA FLJ54392, HIGHLY SIMILAR TO HEAT SHOCK 70 KDA PROTEIN 1.";"CDNA FLJ54408, HIGHLY SIMILAR TO HEAT SHOCK 70 KDA PROTEIN 1."	**1.34**	0.14	15		**1.34**	0.24	12		**1.02**	0.24	12	
IPI00304962	COL1A2	COLLAGEN ALPHA-2(I) CHAIN.	**5.43**	2.55	13	0.177	**8.26**	5.25	12	0.482	**6.64**	2.70	15	0.033
IPI00305092;IPI00790634;IPI00914992	WIBG	ISOFORM 1 OF PARTNER OF Y14 AND MAGO.;ISOFORM 2 OF PARTNER OF Y14 AND MAGO.;23 KDA PROTEIN.			1				1				1	
IPI00305152;IPI00445429;IPI00515103;IPI00746182;IPI00795507;IPI00852741;IPI00853220;IPI00853290;IPI00953506	SEC31A	ISOFORM 3 OF PROTEIN TRANSPORT PROTEIN SEC31A.;ISOFORM 7 OF PROTEIN TRANSPORT PROTEIN SEC31A.;ISOFORM 4 OF PROTEIN TRANSPORT PROTEIN SEC31A.;ISOFORM 1 OF PROTEIN TRANSPORT PROTEIN SEC31A.;ISOFORM 2 OF PROTEIN TRANSPORT PROTEIN SEC31A.;ISOFORM 8 OF PROTEIN TRANSPORT PROTEIN SEC31A.;ISOFORM 6 OF PROTEIN TRANSPORT PROTEIN SEC31A.;ISOFORM 5 OF PROTEIN TRANSPORT PROTEIN SEC31A.	**1.56**	0.00	4		**1.04**	0.00	3		**0.66**	0.00	3	
IPI00305281	TTYH2	PROTEIN TWEETY HOMOLOG 2.	**1.32**	0.00	1		**1.94**	0.00	1		**2.06**	0.00	2	
IPI00305383	UQCRC2	CYTOCHROME B-C1 COMPLEX SUBUNIT 2, MITOCHONDRIAL.			2				2				1	
IPI00305461;IPI00645038	ITIH2	INTER-ALPHA-TRYPSIN INHIBITOR HEAVY CHAIN H2.;INTER-ALPHA (GLOBULIN) INHIBITOR H2.			3				3				6	

IPI00305978;IPI00940335;IPI00946527	AKR7A2	AFLATOXIN B1 ALDEHYDE REDUCTASE MEMBER 2.;32 KDA PROTEIN.;35 KDA PROTEIN.	**1.62**	0.08	2		**1.41**	0.06	2		**0.72**	0.00	2	
IPI00306140;IPI00744153	GCG	GLUCAGON.;PUTATIVE UNCHARACTERIZED PROTEIN GCG.	**2.04**	0.45	13	0.356	**3.05**	0.73	14	0.158	**0.51**	0.12	13	0.242
IPI00306322;IPI00936857;IPI00940293	COL4A2	COLLAGEN ALPHA-2(IV) CHAIN.;168 KDA PROTEIN.;"CDNA FLJ56433, HIGHLY SIMILAR TO COLLAGEN ALPHA-2(IV) CHAIN."	**1.65**	0.25	3	0.213	**2.13**	0.40	4	0.198	**0.85**	0.30	3	0.716
IPI00306332;IPI00791426;IPI00793696;IPI00946221	RPL24	60S RIBOSOMAL PROTEIN L24.;14 KDA PROTEIN.;19 KDA PROTEIN.;18 KDA PROTEIN.	**0.68**	0.16	2		**0.42**	0.00	2		**0.14**	0.00	2	
IPI00306959	KRT7	KERATIN, TYPE II CYTOSKELETAL 7.	**1.69**	0.39	14	0.092	**1.86**	0.49	15	0.082	**3.28**	1.22	26	0.160
IPI00306960	NARS	ASPARAGINYL-TRNA SYNTHETASE, CYTOPLASMIC.	**0.87**	0.09	3	0.233	**0.63**	0.05	3	0.161	**0.32**	0.05	3	0.158
IPI00307545;IPI00925950;IPI00926129	TNS1	TENSIN-1.;TNS1 PROTEIN.			2				2				2	
IPI00328113	FBN1	FIBRILLIN-1.	**3.23**	0.42	5	0.166	**7.89**	1.12	6	0.122	**9.70**	0.57	6	0.160
IPI00328170;IPI00916474	MOGS	MANNOSYL-OLIGOSACCHARIDE GLUCOSIDASE.;MANNOSYL-OLIGOSACCHARIDE GLUCOSIDASE ISOFORM 2.	**1.05**	0.07	4	0.379	**0.55**	0.04	4	0.067	**0.24**	0.01	4	0.103
IPI00328243	PLD3	PHOSPHOLIPASE D3.			1									
IPI00328257;IPI00384489;IPI00413947;IPI00940292;IPI00946351;IPI00953658	AP1B1	ISOFORM A OF AP-1 COMPLEX SUBUNIT BETA-1.;ADAPTOR-RELATED PROTEIN COMPLEX 1 BETA 1 SUBUNIT ISOFORM C.;ISOFORM B OF AP-1 COMPLEX SUBUNIT BETA-1.;ADAPTOR-RELATED PROTEIN COMPLEX 1 BETA 1 SUBUNIT ISOFORM B.;65 KDA PROTEIN.	**0.68**	0.00	2		**0.78**	0.00	2		**0.68**	0.00	2	
IPI00328350;IPI00872650	FAM129A	PROTEIN NIBAN.;102 KDA PROTEIN.	**0.66**	0.13	4		**0.47**	0.08	4		**0.12**	0.00	4	
IPI00328415;IPI00446235;IPI00871319	CYB5R3	CDNA FLJ56301, HIGHLY SIMILAR TO NADH-CYTOCHROME B5 REDUCTASE.;ISOFORM 2 OF NADH-CYTOCHROME B5 REDUCTASE 3.;34 KDA PROTEIN.	**1.33**	0.04	4	0.285	**1.97**	0.16	5	0.234	**1.67**	0.19	4	0.410
IPI00328715	MTDH	PROTEIN LYRIC.	**1.51**	0.00	1		**1.39**	0.00	1		**1.06**	0.00	1	
IPI00328748;IPI00924819;IPI00927651	MANF	MESENCEPHALIC ASTROCYTE-DERIVED NEUROTROPHIC FACTOR.;PROTEIN.	**1.74**	0.00	1		**0.73**	0.00	1				1	
IPI00329352;IPI00333985;IPI00413732;IPI00465432;IPI00872411;IPI00941810	NOMO1;NOMO3;NOMO2	NODAL MODULATOR 1.;ISOFORM 2 OF NODAL MODULATOR 2.;139 KDA PROTEIN.;ISOFORM 1 OF NODAL MODULATOR 2.;NODAL MODULATOR 3.	**1.09**	0.10	2		**0.88**	0.00	2				2	
IPI00329389;IPI00790342;IPI00867533	RPL6	60S RIBOSOMAL PROTEIN L6.	**0.75**	0.05	11	0.044	**0.59**	0.10	11	0.087	**0.35**	0.08	11	0.114
IPI00329482;IPI00735310	LAMA4	ISOFORM 1 OF LAMININ SUBUNIT ALPHA-4.;ISOFORM 2 OF LAMININ SUBUNIT ALPHA-4.							2				2	
IPI00329598	HSD17B11	ESTRADIOL 17-BETA-DEHYDROGENASE 11.			1				1				1	
IPI00329600;IPI00641681	SCCPDH	PROBABLE SACCHAROPINE DEHYDROGENASE.;SACCHAROPINE DEHYDROGENASE.											1	
IPI00329633;IPI00908949;IPI00910719	TARS	THREONYL-TRNA SYNTHETASE, CYTOPLASMIC.;"CDNA FLJ53464, HIGHLY SIMILAR TO THREONYL TRNA SYNTHETASE, CYTOPLASMIC.";"CDNA FLJ55705, HIGHLY SIMILAR TO THREONYL-TRNA SYNTHETASE, CYTOPLASMIC."	**0.82**	0.10	5	0.264	**0.75**	0.06	5	0.485	**0.36**	0.03	5	
IPI00329801;IPI00872379	ANXA5	ANNEXIN A5.;PUTATIVE UNCHARACTERIZED PROTEIN ANXA5 (FRAGMENT).	**1.39**	0.22	17	0.028	**1.36**	0.15	18	0.148	**1.06**	0.08	16	0.713
IPI00332371;IPI00925520	PFKL	ISOFORM 1 OF 6-PHOSPHOFRUCTOKINASE, LIVER TYPE.;"6-PHOSPHOFRUCTOKINASE, LIVER TYPE (EC 2.7.1.11) (PHOSPHOFRUCTOKINASE 1) (PHOSPHOHEXOKINASE) (PHOSPHOFRUCTO-1-KINASE ISOZYME B) (PFK-B). ISOFORM 2."	**1.34**	0.00	1		**1.49**	0.00	1				1	
IPI00332493;IPI00791133;IPI00793168	PLEKHO2	ISOFORM 1 OF PLECKSTRIN HOMOLOGY DOMAIN-CONTAINING FAMILY O MEMBER 2.;ISOFORM 2 OF PLECKSTRIN HOMOLOGY DOMAIN-CONTAINING FAMILY O MEMBER 2.;25 KDA PROTEIN.							1				1	
IPI00334159	VBP1	VON HIPPEL-LINDAU BINDING PROTEIN 1.											1	
IPI00334190;IPI00908723	STOML2	STOMATIN-LIKE PROTEIN 2.;"CDNA FLJ61039, HIGHLY SIMILAR TO STOMATIN-LIKE PROTEIN 2."	**1.04**	0.09	4		**0.60**	0.00	3		**0.54**	0.08	3	
IPI00334587;IPI00334713;IPI00742926	HNRNPAB	ISOFORM 2 OF HETEROGENEOUS NUCLEAR RIBONUCLEOPROTEIN A/B.;ISOFORM 3 OF HETEROGENEOUS NUCLEAR RIBONUCLEOPROTEIN A/B.;PUTATIVE UNCHARACTERIZED PROTEIN HNRNPAB.	**2.26**	0.00	1	0.206	**1.76**	0.00	1	0.416	**1.64**	0.00	1	
IPI00334627	ANXA2P2	PUTATIVE ANNEXIN A2-LIKE PROTEIN.	**1.86**	0.32	13	0.034	**3.06**	0.70	17	0.004	**3.21**	0.75	20	0.004
IPI00335168;IPI00413922;IPI00789605;IPI00795576;IPI00796366;IPI00930599	MYL6B;MYL6;MYL6B;MYL6	ISOFORM NON-MUSCLE OF MYOSIN LIGHT POLYPEPTIDE 6.;11 KDA PROTEIN.;ISOFORM SMOOTH MUSCLE OF MYOSIN LIGHT POLYPEPTIDE 6.;13 KDA PROTEIN.;"CDNA FLJ56329, HIGHLY SIMILAR TO MYOSIN LIGHT POLYPEPTIDE 6.";16 KDA PROTEIN.	**1.65**	0.21	7	0.202	**2.66**	0.09	7	0.082	**2.34**	0.09	5	0.103
IPI00337335;IPI00607818;IPI00926581;IPI00941217	MYH14	ISOFORM 1 OF MYOSIN-14.;"MYOSIN, HEAVY CHAIN 14 ISOFORM 1.";"MYOSIN, HEAVY CHAIN 14 ISOFORM 3.";ISOFORM 2 OF MYOSIN-14.				0.250				0.231	**2.27**	0.01	8	
IPI00337541	NNT	NAD(P) TRANSHYDROGENASE, MITOCHONDRIAL.			1				1				1	
IPI00339224	FN1	FIBRONECTIN 1 ISOFORM 4 PREPROPROTEIN.				0.177	**5.08**	0.89	27	0.003	**5.75**	1.10	28	0.039
IPI00339226	FN1	ISOFORM 6 OF FIBRONECTIN.				0.262	**4.78**	0.82	26	0.007	**5.24**	0.85	27	0.064
IPI00374563	AGRN	AGRIN.	**0.88**	0.00	1	0.277	**0.71**	0.00	1	0.345	**0.49**	0.00	1	0.601
IPI00375462	SFRS12	ISOFORM 2 OF SPLICING FACTOR, ARGININE/SERINE-RICH 12.			1		**1.61**	0.00	1				1	
IPI00375676	FTL	FERRITIN.	**3.02**	0.05	5		**4.09**	0.57	6		**4.79**	0.03	7	
IPI00376005;IPI00411704	EIF5A	ISOFORM 2 OF EUKARYOTIC TRANSLATION INITIATION FACTOR 5A-1.;ISOFORM 1 OF EUKARYOTIC TRANSLATION INITIATION FACTOR 5A-1.	**0.80**	0.11	4	0.094	**0.68**	0.12	4	0.159	**0.41**	0.02	4	
IPI00376121;IPI00401819;IPI00479543;IPI00655650;IPI00847149	RPS26	13 KDA PROTEIN.;SIMILAR TO 40S RIBOSOMAL PROTEIN S26.;31 KDA PROTEIN.;40S RIBOSOMAL PROTEIN S26.	**0.84**	0.00	1		**1.17**	0.00	1		**0.47**	0.00	1	
IPI00376798;IPI00746438	RPL11	ISOFORM 1 OF 60S RIBOSOMAL PROTEIN L11.;ISOFORM 2 OF 60S RIBOSOMAL PROTEIN L11.	**0.77**	0.06	2		**0.61**	0.00	3		**0.30**	0.00	2	
IPI00377161;IPI00419802	HIBCH	ISOFORM 2 OF 3-HYDROXYISOBUTYRYL-COA HYDROLASE, MITOCHONDRIAL.;"ISOFORM 1 OF 3-HYDROXYISOBUTYRYL-COA HYDROLASE, MITOCHONDRIAL."	**0.71**	0.03	3		**0.61**	0.00	3		**0.12**	0.00	3	
IPI00382470;IPI00784295	HSP90AA1	ISOFORM 2 OF HEAT SHOCK PROTEIN HSP 90-ALPHA.;ISOFORM 1 OF HEAT SHOCK PROTEIN HSP 90-ALPHA.	**1.20**	0.11	13	0.281	**1.36**	0.25	12	0.392	**1.35**	0.35	13	0.486
IPI00382698	FLNB	ISOFORM 4 OF FILAMIN-B.				0.301				0.190	**2.76**	0.93	23	0.150
IPI00382699	FLNB	ISOFORM 5 OF FILAMIN-B.				0.301				0.190	**2.76**	0.93	23	0.150
IPI00383645;IPI00855763;IPI00856116	PALLD	ISOFORM 4 OF PALLADIN.;ISOFORM 3 OF PALLADIN.;ISOFORM 1 OF PALLADIN.											2	
IPI00384016;IPI00420108;IPI00954003	DLSTP;DLST	FULL-LENGTH CDNA 5-PRIME END OF CLONE CS0DJ009YL13 OF T CELLS (JURKAT CELL LINE) OF HOMO SAPIENS (FRAGMENT).;"DIHYDROLIPOYLLYSINE-RESIDUE SUCCINYLTRANSFERASE COMPONENT OF 2- OXOGLUTARATE DEHYDROGENASE COMPLEX, MITOCHONDRIAL."	**1.33**	0.00	1	0.474	**1.19**	0.00	1	0.400	**0.86**	0.00	1	0.473
IPI00384051;IPI00746205;IPI00943181	PSME2	PUTATIVE UNCHARACTERIZED PROTEIN PSME2.;PROTEASOME ACTIVATOR SUBUNIT 2.;29 KDA PROTEIN.	**0.94**	0.06	6	0.404	**0.79**	0.19	6	0.408	**0.99**	0.22	6	0.534
IPI00384697;IPI00953582	ALB	ISOFORM 2 OF SERUM ALBUMIN.	**1.87**	0.78	32	0.358	**3.53**	0.97	30	0.003	**4.39**	0.99	32	0.021
IPI00384938;IPI00423463;IPI00423464;IPI00423466;IPI00448925;IPI00645363;IPI00784817;IPI00784842;IPI00785084;IPI00816314;IPI00876888;IPI00930124	LOC100294459;LOC100290320;IGHG1;IGH@;IGHV4-31	PUTATIVE UNCHARACTERIZED PROTEIN DKFZP686N02209.;PUTATIVE UNCHARACTERIZED PROTEIN DKFZP686O01196.;PUTATIVE UNCHARACTERIZED PROTEIN DKFZP686K03196.;PUTATIVE UNCHARACTERIZED PROTEIN DKFZP686H20196.;44 KDA PROTEIN.;PUTATIVE UNCHARACTERIZED PROTEIN DKFZP686P15220.;ANTI-RHD MONOCLONAL T125 GAMMA1 HEAVY CHAIN.;PUTATIVE UNCHARACTERIZED PROTEIN DKFZP686G11190.;IGH@ PROTEIN.;PUTATIVE UNCHARACTERIZED PROTEIN DKFZP686I15196.;CDNA FLJ78387.;PUTATIVE UNCHARACTERIZED PROTEIN DKFZP686C11235.				0.112	**6.63**	0.34	8	0.037	**7.75**	0.86	10	0.282
IPI00385264;IPI00479708;IPI00892870;IPI00896380;IPI00939620;IPI00940716;IPI00940918;IPI00941355;IPI00941961;IPI00946337;IPI00952640	IGHM	IG MU HEAVY CHAIN DISEASE PROTEIN.;FULL-LENGTH CDNA CLONE CS0DD006YL02 OF NEUROBLASTOMA OF HOMO SAPIENS.;PROTEIN.;ISOFORM 2 OF IG MU CHAIN C REGION.;54 KDA PROTEIN.;52 KDA PROTEIN.;53 KDA PROTEIN.;66 KDA PROTEIN.	**1.21**	0.00	3		**2.21**	0.32	5		**2.10**	0.27	5	
IPI00385495;IPI00852693;IPI00854764	LMF2	ISOFORM 1 OF LIPASE MATURATION FACTOR 2.;ISOFORM 2 OF LIPASE MATURATION FACTOR 2.;ISOFORM 3 OF LIPASE MATURATION FACTOR 2.			1									
IPI00386524;IPI00423462;IPI00647704	IGHA1	CDNA FLJ25298 FIS, CLONE STM07683, HIGHLY SIMILAR TO PROTEIN TRO ALPHA1 H,MYELOMA.;PUTATIVE UNCHARACTERIZED PROTEIN DKFZP686K18196 (FRAGMENT).;"CDNA FLJ41552 FIS, CLONE COLON2004478, HIGHLY SIMILAR TO PROTEIN TRO ALPHA1 H,MYELOMA."	**2.91**	0.53	8	0.205	**3.64**	1.42	10	0.070				0.090
IPI00386755;IPI00872773	ERO1L	ERO1-LIKE PROTEIN ALPHA.;PUTATIVE UNCHARACTERIZED PROTEIN ERO1L.			1				1				1	
IPI00394820	OLFML1	OLFACTOMEDIN-LIKE PROTEIN 1.							1				1	
IPI00396321	LRRC59	LEUCINE-RICH REPEAT-CONTAINING PROTEIN 59.	**0.67**	0.12	4	0.079	**0.65**	0.10	3		**0.23**	0.00	3	
IPI00396370;IPI00719752	EIF3B	ISOFORM 1 OF EUKARYOTIC TRANSLATION INITIATION FACTOR 3 SUBUNIT B.;ISOFORM 2 OF EUKARYOTIC TRANSLATION INITIATION FACTOR 3 SUBUNIT B.			2				2		**0.64**	0.00	2	
IPI00396378;IPI00414696;IPI00916517	HNRNPA2B1	ISOFORM B1 OF HETEROGENEOUS NUCLEAR RIBONUCLEOPROTEINS A2/B1.;ISOFORM A2 OF HETEROGENEOUS NUCLEAR RIBONUCLEOPROTEINS A2/B1.;PUTATIVE UNCHARACTERIZED PROTEIN HNRNPA2B1.	**1.13**	0.11	9	0.469	**1.15**	0.23	12	0.578	**0.93**	0.17	12	0.605
IPI00396383	VWA1	ISOFORM 1 OF VON WILLEBRAND FACTOR A DOMAIN-CONTAINING PROTEIN 1.							2					

IPI00397526;IPI00479307;IPI00790503	MYH10	ISOFORM 1 OF MYOSIN-10.;ISOFORM 2 OF MYOSIN-10.;ISOFORM 3 OF MYOSIN-10.	**1.73**	0.24	12	0.180	**2.14**	0.38	13	0.071	**2.50**	0.13	13	0.102
IPI00398002	PLEC1	ISOFORM 3 OF PLECTIN-1.				0.025				0.003	**3.07**	0.34	45	0.001
IPI00398779	PLEC1	ISOFORM 4 OF PLECTIN-1.	**1.46**	0.18	22	0.025	**2.22**	0.35	28	0.003	**3.07**	0.34	45	0.001
IPI00399007;IPI00426051;IPI00930072;IPI00930442	IGHG2;IGHG4	PUTATIVE UNCHARACTERIZED PROTEIN DKFZP686I04196 (FRAGMENT).;PUTATIVE UNCHARACTERIZED PROTEIN DKFZP686C15213.;PUTATIVE UNCHARACTERIZED PROTEIN DKFZP686E23209.;PUTATIVE UNCHARACTERIZED PROTEIN DKFZP686M24218.			4				1				5	
IPI00399318;IPI00465132	COPE	EPSILON SUBUNIT OF COATOMER PROTEIN COMPLEX ISOFORM B.;COATOMER SUBUNIT EPSILON.	**1.16**	0.18	2	0.349	**1.32**	0.21	2		**0.57**	0.00	2	
IPI00401264	ERP44	ENDOPLASMIC RETICULUM RESIDENT PROTEIN ERP44.			1		**1.02**	0.00	1				2	
IPI00402215	COL14A1	ISOFORM 3 OF COLLAGEN ALPHA-1(XIV) CHAIN.	**5.88**	1.00	26	0.177	**14.18**	2.77	26	0.082	**10.06**	1.65	28	0.274
IPI00410693;IPI00412714;IPI00470497;IPI00470498	SERBP1	ISOFORM 1 OF PLASMINOGEN ACTIVATOR INHIBITOR 1 RNA-BINDING PROTEIN.;ISOFORM 4 OF PLASMINOGEN ACTIVATOR INHIBITOR 1 RNA-BINDING PROTEIN.;ISOFORM 2 OF PLASMINOGEN ACTIVATOR INHIBITOR 1 RNA-BINDING PROTEIN.;ISOFORM 3 OF PLASMINOGEN ACTIVATOR INHIBITOR 1 RNA-BINDING PROTEIN.	**1.02**	0.04	4		**0.56**	0.00	4		**0.41**	0.06	4	
IPI00410714	HBA2;HBA1	HEMOGLOBIN SUBUNIT ALPHA.	**1.38**	0.24	14	0.252	**3.28**	0.82	15	0.269	**1.27**	0.22	15	0.437
IPI00411706;IPI00641040	ESD	S-FORMYLGLUTATHIONE HYDROLASE.;28 KDA PROTEIN.	**0.80**	0.00	1		**1.17**	0.00	1		**0.67**	0.00	1	
IPI00412545;IPI00554681;IPI00946670	NDUFA5	PUTATIVE UNCHARACTERIZED PROTEIN DKFZP781K1356.;NADH DEHYDROGENASE [UBIQUINONE] 1 ALPHA SUBCOMPLEX SUBUNIT 5.;PROTEIN.	**1.09**	0.07	2	0.446	**0.56**	0.03	2		**0.66**	0.00	2	
IPI00412579	RPL10A	60S RIBOSOMAL PROTEIN L10A.	**0.61**	0.00	2		**0.35**	0.00	2		**0.21**	0.00	2	
IPI00413324;IPI00478208;IPI00644171;IPI00874214	RPL17	60S RIBOSOMAL PROTEIN L17.;21 KDA PROTEIN.;PUTATIVE UNCHARACTERIZED PROTEIN RPL17P34.;11 KDA PROTEIN.			2				2				2	
IPI00413451;IPI00749398	SERPINB6	PUTATIVE UNCHARACTERIZED PROTEIN DKFZP686I04222.;SERPIN B6.	**0.94**	0.06	4	0.474	**0.60**	0.03	4	0.173	**0.48**	0.00	4	
IPI00413587;IPI00420084;IPI00472003;IPI00943215	BID	ISOFORM 1 OF BH3-INTERACTING DOMAIN DEATH AGONIST.;ISOFORM 2 OF BH3-INTERACTING DOMAIN DEATH AGONIST.;ISOFORM 4 OF BH3-INTERACTING DOMAIN DEATH AGONIST.;22 KDA PROTEIN.							1				1	
IPI00414320;IPI00647738;IPI00909703	ANXA11	CDNA FLJ55482, HIGHLY SIMILAR TO ANNEXIN A11.;ANNEXIN A11.;"CDNA FLJ51518, HIGHLY SIMILAR TO ANNEXIN A11."							1				1	
IPI00414442	CDK5RAP3	CDNA FLJ56403, HIGHLY SIMILAR TO CDK5 REGULATORY SUBUNIT-ASSOCIATED PROTEIN 3.	**0.83**	0.03	4	0.294	**0.74**	0.05	4				4	
IPI00414676	HSP90AB1	HEAT SHOCK PROTEIN HSP 90-BETA.	**1.11**	0.14	15	0.452	**1.15**	0.30	15	0.578	**1.15**	0.40	15	0.715
IPI00414836	OSTF1	OSTEOCLAST-STIMULATING FACTOR 1.			1				1				1	
IPI00418169;IPI00455315	ANXA2	ISOFORM 2 OF ANNEXIN A2.;ISOFORM 1 OF ANNEXIN A2.	**1.96**	0.29	19	0.007	**3.09**	0.60	24	0.001	**3.28**	0.66	27	0.001
IPI00418262;IPI00789171;IPI00792375	ALDOC	FRUCTOSE-BISPHOSPHATE ALDOLASE.;16 KDA PROTEIN.	**1.31**	0.00	1		**1.23**	0.00	2		**1.54**	0.00	1	
IPI00418431;IPI00647693	ASPN	ASPN PROTEIN.;"ASPORIN (LRR CLASS 1), ISOFORM CRA_B."			1				3				3	
IPI00418471	VIM	VIMENTIN.	**2.37**	0.32	42	0.000	**4.19**	0.70	45	0.000	**2.78**	0.50	42	0.000
IPI00419100;IPI00471914;IPI00942099	FGD6	ISOFORM 1 OF FYVE, RHOGEF AND PH DOMAIN-CONTAINING PROTEIN 6.;"ISOFORM 2 OF FYVE, RHOGEF AND PH DOMAIN-CONTAINING PROTEIN 6.";FGD6 PROTEIN.							1				1	
IPI00419235	GSTM5	GLUTATHIONE S-TRANSFERASE MU 5.					**1.75**	0.05	2					
IPI00419237;IPI00789806	LAP3	ISOFORM 1 OF CYTOSOL AMINOPEPTIDASE.;ISOFORM 2 OF CYTOSOL AMINOPEPTIDASE.	**1.31**	0.00	2		**1.67**	0.00	2		**1.54**	0.00	1	
IPI00419373;IPI00455134;IPI00909208;IPI00927407;IPI00927677;IPI00939124	HNRNPA3	ISOFORM 1 OF HETEROGENEOUS NUCLEAR RIBONUCLEOPROTEIN A3.;ISOFORM 2 OF HETEROGENEOUS NUCLEAR RIBONUCLEOPROTEIN A3.;"CDNA FLJ58832, HIGHLY SIMILAR TO HETEROGENEOUS NUCLEAR RIBONUCLEOPROTEIN A3.";"CDNA FLJ59080, HIGHLY SIMILAR TO HETEROGENEOUS NUCLEAR RIBONUCLEOPROTEIN A3.";34 KDA PROTEIN.;37 KDA PROTEIN.	**1.27**	0.10	3		**1.55**	0.13	3	0.191	**1.27**	0.11	3	0.348
IPI00419585;IPI00925411;IPI00925747	PPIA	PEPTIDYL-PROLYL CIS-TRANS ISOMERASE A.;13 KDA PROTEIN.	**1.43**	0.23	3									
IPI00419880;IPI00472119	RPS3A	40S RIBOSOMAL PROTEIN S3A.;30 KDA PROTEIN.	**1.14**	0.09	3		**0.47**	0.00	3		**0.30**	0.00	3	
IPI00419986	RBP2	RETINOL-BINDING PROTEIN 2.			1									
IPI00420117;IPI00746004	RPS27L	11 KDA PROTEIN.;40S RIBOSOMAL PROTEIN S27-LIKE PROTEIN.	**1.10**	0.00	2		**1.03**	0.00	2				2	
IPI00423461;IPI00641229;IPI00642017;IPI00784758;IPI00784830;IPI00784950;IPI00785067;IPI00893853;IPI00894384;IPI00940245	IGHA2;LOC100126583	PUTATIVE UNCHARACTERIZED PROTEIN DKFZP686C02220 (FRAGMENT).;IG ALPHA-2 CHAIN C REGION.;PUTATIVE UNCHARACTERIZED PROTEIN DKFZP686C02218 (FRAGMENT).;PUTATIVE UNCHARACTERIZED PROTEIN DKFZP686M08189.;"CDNA FLJ41981 FIS, CLONE SMINT2011888, HIGHLY SIMILAR TO PROTEIN TRO ALPHA1 H,MYELOMA.";PUTATIVE UNCHARACTERIZED PROTEIN DKFZP686L19235.;IGH@ PROTEIN.;PROTEIN.;PUTATIVE UNCHARACTERIZED PROTEIN DKFZP686O16217 (FRAGMENT).;IMMUNOGLOBULIN HEAVY CHAIN VARIANT (FRAGMENT).	**2.65**	0.44	5		**3.18**	1.34	7		**3.69**	1.01	6	
IPI00429633;IPI00643134	ERO1LB	ERO1-LIKE PROTEIN BETA.;ERO1-LIKE BETA.	**0.93**	0.00	2		**0.94**	0.00	2				2	
IPI00431405;IPI00654793;IPI00845432	C5orf33	ISOFORM 2 OF UPF0465 PROTEIN C5ORF33.;ISOFORM 3 OF UPF0465 PROTEIN C5ORF33.;ISOFORM 1 OF UPF0465 PROTEIN C5ORF33.	**1.45**	0.00	1						**0.59**	0.00	1	
IPI00431645;IPI00478493;IPI00641737;IPI00942787	HP	HP PROTEIN.;HAPTOGLOBIN ISOFORM 2 PREPROPROTEIN.;HAPTOGLOBIN.;42 KDA PROTEIN.	**0.72**	0.08	2		**1.82**	0.00	3		**6.54**	2.48	3	
IPI00440493	ATP5A1	ATP SYNTHASE SUBUNIT ALPHA, MITOCHONDRIAL.	**0.97**	0.11	16	0.354	**0.78**	0.10	16	0.161	**0.48**	0.10	16	0.075
IPI00442073	CSRP1	CYSTEINE AND GLYCINE-RICH PROTEIN 1.	**1.32**	0.00	3		**1.93**	0.00	3		**2.25**	0.00	3	
IPI00443909	CNPY2	ISOFORM 1 OF PROTEIN CANOPY HOMOLOG 2.			1				1				1	
IPI00444262;IPI00604620	NCL	CDNA FLJ45706 FIS, CLONE FEBRA2028457, HIGHLY SIMILAR TO NUCLEOLIN.;NUCLEOLIN.	**1.14**	0.11	12	0.480	**0.95**	0.08	11	0.450	**0.79**	0.06	12	0.565
IPI00446769;IPI00607799	BDH2	ISOFORM 2 OF 3-HYDROXYBUTYRATE DEHYDROGENASE TYPE 2.;ISOFORM 1 OF 3-HYDROXYBUTYRATE DEHYDROGENASE TYPE 2.	**1.46**	0.00	1		**1.22**	0.00	1		**1.18**	0.00	1	
IPI00449049	PARP1	POLY [ADP-RIBOSE] POLYMERASE 1.			2		**0.43**	0.00	1				1	
IPI00452747;IPI00472939	LOC653566;SPCS2	SIMILAR TO SIGNAL PEPTIDASE COMPLEX SUBUNIT 2.;SIGNAL PEPTIDASE COMPLEX SUBUNIT 2.			1				1				1	
IPI00453473	HIST1H4E;HIST4H4;HIST2H4A;HIST1H4F;HIST1H4L;HIST1H4H;HIST1H4C;HIST1H4K;HIST2H4B;HIST1H4I;HIST1H4B;HIST1H4A;HIST1H4J;HIST1H4D	HISTONE H4.	**1.72**	0.14	10	0.031	**1.32**	0.14	10	0.493	**0.84**	0.16	10	0.598
IPI00453476;IPI00549725	PGAM1	29 KDA PROTEIN.;PHOSPHOGLYCERATE MUTASE 1.	**1.03**	0.08	5	0.336	**1.33**	0.16	5	0.345	**1.06**	0.14	3	0.352
IPI00456758	RPL27A	60S RIBOSOMAL PROTEIN L27A.	**0.95**	0.00	2				2				2	
IPI00456969	DYNC1H1	CYTOPLASMIC DYNEIN 1 HEAVY CHAIN 1.	**1.39**	0.19	4	0.354	**1.14**	0.22	4		**0.87**	0.17	4	
IPI00464952;IPI00641287;IPI00641957;IPI00908907;IPI00910208	SFRS11	SPLICING FACTOR, ARGININE/SERINE-RICH 11.;"SPLICING FACTOR, ARGININE/SERINE-RICH 11, ISOFORM CRA_B.";SFRS11 PROTEIN.;"CDNA FLJ52287, HIGHLY SIMILAR TO SPLICING FACTOR ARGININE/SERINE-RICH 11.";"CDNA FLJ58939, HIGHLY SIMILAR TO SPLICING FACTOR ARGININE/SERINE-RICH 11."	**1.04**	0.00	1		**1.17**	0.00	1		**0.80**	0.00	1	
IPI00465028;IPI00797270	TPI1;RCTPI1	TRIOSEPHOSPHATE ISOMERASE 1 ISOFORM 2.;ISOFORM 1 OF TRIOSEPHOSPHATE ISOMERASE.	**1.24**	0.15	12	0.177	**1.26**	0.16	13	0.500	**0.99**	0.14	13	0.563
IPI00465084	DES	DESMIN.	**2.67**	0.26	24	0.074	**3.48**	0.65	16	0.098	**2.99**	0.52	24	0.075
IPI00465085;IPI00880164	NAPRT1	ISOFORM 1 OF NICOTINATE PHOSPHORIBOSYLTRANSFERASE.;ISOFORM 3 OF NICOTINATE PHOSPHORIBOSYLTRANSFERASE.							1				1	
IPI00465233;IPI00745266;IPI00879876;IPI00910633	EIF3L	EUKARYOTIC TRANSLATION INITIATION FACTOR 3, SUBUNIT E INTERACTING PROTEIN.;EUKARYOTIC TRANSLATION INITIATION FACTOR 3 SUBUNIT L.;"CDNA FLJ59381, HIGHLY SIMILAR TO EUKARYOTIC TRANSLATION INITIATION FACTOR 3 SUBUNIT 6-INTERACTING PROTEIN."	**0.89**	0.06	3		**0.84**	0.05	3		**0.70**	0.00	3	
IPI00465248	ENO1	ISOFORM ALPHA-ENOLASE OF ALPHA-ENOLASE.	**1.17**	0.11	13	0.133	**1.62**	0.24	14	0.082	**1.38**	0.21	15	0.117
IPI00465361	RPL13	60S RIBOSOMAL PROTEIN L13.	**0.74**	0.06	7	0.112	**0.57**	0.15	6	0.076	**0.19**	0.04	6	0.060
IPI00465431	LGALS3	GALECTIN-3.											1	
IPI00465436	CAT	CATALASE.	**1.88**	0.14	4	0.094	**3.08**	0.36	4	0.318	**1.77**	0.22	4	
IPI00465439;IPI00796333	ALDOA	FRUCTOSE-BISPHOSPHATE ALDOLASE A.;45 KDA PROTEIN.	**1.09**	0.04	5	0.399	**0.38**	0.27	5	0.610	**1.24**	0.40	6	0.604
IPI00470559;IPI00760928;IPI00760962	KANK2	ISOFORM 1 OF KN MOTIF AND ANKYRIN REPEAT DOMAIN-CONTAINING PROTEIN 2.;ISOFORM 3 OF KN MOTIF AND ANKYRIN REPEAT DOMAIN-CONTAINING PROTEIN 2.;ISOFORM 2 OF KN MOTIF AND ANKYRIN REPEAT DOMAIN-CONTAINING PROTEIN 2.			1				1				1	
IPI00470649;IPI00607732	NCLN	ISOFORM 1 OF NICALIN.;ISOFORM 2 OF NICALIN.			1				1				1	
IPI00477118;IPI00843881;IPI00909345	TMEM214	TRANSMEMBRANE PROTEIN 214.;TRANSMEMBRANE PROTEIN 214 ISOFORM 2.;CDNA FLJ58194.			1		**0.95**	0.00	1				1	

IPI00477992;IPI00643948	C1QB	COMPLEMENT COMPONENT 1, Q SUBCOMPONENT, B CHAIN PRECURSOR.;"COMPLEMENT COMPONENT 1, Q SUBCOMPONENT, B CHAIN."											1	
IPI00478003	A2M	ALPHA-2-MACROGLOBULIN.	**0.90**	0.13	12		**1.08**	0.27	12		**0.98**	0.20	13	
IPI00479145	KRT19	KERATIN, TYPE I CYTOSKELETAL 19.	**1.54**	0.25	21	0.028	**2.42**	0.49	23	0.046	**4.06**	1.51	32	0.028
IPI00479186;IPI00847989;IPI00910979	PKM2	ISOFORM M2 OF PYRUVATE KINASE ISOZYMES M1/M2.;PYRUVATE KINASE.	**1.22**	0.11	15		**1.45**	0.22	13		**2.52**	0.54	13	
IPI00479217;IPI00644079;IPI00644224;IPI00883857	HNRNPU	ISOFORM SHORT OF HETEROGENEOUS NUCLEAR RIBONUCLEOPROTEIN U.;HNRPU PROTEIN.;"CDNA FLJ54020, HIGHLY SIMILAR TO HETEROGENEOUS NUCLEAR RIBONUCLEOPROTEIN U.";ISOFORM LONG OF HETEROGENEOUS NUCLEAR RIBONUCLEOPROTEIN U.	**0.88**	0.09	3	0.506	**0.86**	0.18	4	0.611	**0.59**	0.12	3	0.591
IPI00479306;IPI00910237	PSMB5	PROTEASOME SUBUNIT BETA TYPE-5.;PROTEASOME BETA 5 SUBUNIT ISOFORM 2.							1					
IPI00479722	PSME1	PROTEASOME ACTIVATOR COMPLEX SUBUNIT 1.	**1.34**	0.08	3	0.143	**1.17**	0.01	4	0.610	**1.31**	0.10	5	0.486
IPI00479723	FN1	FN1 PROTEIN.				0.177	**5.08**	0.89	27	0.003	**5.75**	1.10	28	0.039
IPI00479786;IPI00855957;IPI00909344	KHSRP	KH-TYPE SPLICING REGULATORY PROTEIN.;ISOFORM 2 OF FAR UPSTREAM ELEMENT-BINDING PROTEIN 2.;"CDNA FLJ51330, HIGHLY SIMILAR TO FAR UPSTREAM ELEMENT-BINDING PROTEIN 2."	**1.28**	0.00	2		**1.03**	0.00	1				1	
IPI00479877	ALDH9A1	ALDEHYDE DEHYDROGENASE 9A1.	**1.02**	0.00	2	0.396	**1.08**	0.00	2	0.500	**0.39**	0.00	2	
IPI00514204	LMNA	LAMIN A/C.	**2.05**	0.33	26	0.001	**2.89**	0.47	24	0.002	**1.92**	0.33	26	0.045
IPI00514310;IPI00607735;IPI00644969;IPI00645501	TSTD1	ISOFORM 4 OF PUTATIVE THIOSULFATE SULFURTRANSFERASE KAT.;ISOFORM 2 OF PUTATIVE THIOSULFATE SULFURTRANSFERASE KAT.;ISOFORM 1 OF PUTATIVE THIOSULFATE SULFURTRANSFERASE KAT.;ISOFORM 3 OF PUTATIVE THIOSULFATE SULFURTRANSFERASE KAT.	**0.72**	0.00	1		**0.70**	0.00	1				1	
IPI00514399;IPI00879531	RPS27	RIBOSOMAL PROTEIN S27.;9 KDA PROTEIN.	**1.49**	0.00	2		**1.34**	0.00	2		**0.81**	0.00	2	
IPI00514893	DAAM2	DISHEVELED-ASSOCIATED ACTIVATOR OF MORPHOGENESIS 2.							1					
IPI00515087;IPI00742763	CTRB2	CHYMOTRYPSIN B2 PRECURSOR.;CHYMOTRYPSINOGEN B2.	**0.83**	0.11	7	0.230	**0.53**	0.09	7	0.155	**0.04**	0.00	7	
IPI00549236;IPI00854851	CCDC90B	ISOFORM 1 OF COILED-COIL DOMAIN-CONTAINING PROTEIN 90B, MITOCHONDRIAL.;"ISOFORM 2 OF COILED-COIL DOMAIN-CONTAINING PROTEIN 90B, MITOCHONDRIAL."			1				1				1	
IPI00550020;IPI00815656;IPI00830138	PTMS	PARATHYMOSIN.;PARATHYMOSIN (FRAGMENT).;PTMS PROTEIN (FRAGMENT).			2				2		**1.62**	0.01	2	
IPI00550069	RNH1	RIBONUCLEASE INHIBITOR.	**1.94**	0.00	1		**2.59**	0.00	1		**1.75**	0.00	1	
IPI00550239	H1F0	HISTONE H1.0.	**1.61**	0.14	5		**1.30**	0.16	5	0.456	**1.48**	0.39	5	
IPI00550363;IPI00644531;IPI00647915	TAGLN2	TRANSGELIN-2.;21 KDA PROTEIN.;24 KDA PROTEIN.	**1.55**	0.04	3	0.177	**1.84**	0.13	4	0.323	**1.69**	0.20	5	0.282
IPI00550644;IPI00877947;IPI00910722	TTC38	TETRATRICOPEPTIDE REPEAT PROTEIN 38.;PROTEIN.;CDNA FLJ58627.	**0.74**	0.00	1		**0.66**	0.00	1				1	
IPI00550731		PUTATIVE UNCHARACTERIZED PROTEIN.	**4.41**	0.00	5		**5.44**	0.00	5		**5.69**	0.00	7	
IPI00550991;IPI00847635	SERPINA3	CDNA FLJ35730 FIS, CLONE TESTI2003131, HIGHLY SIMILAR TO ALPHA-1-ANTICHYMOTRYPSIN.;ISOFORM 1 OF ALPHA-1-ANTICHYMOTRYPSIN.	**1.88**	0.11	2	0.264	**3.12**	0.00	1	0.238	**4.62**	0.03	9	0.545
IPI00551024	DAK	BIFUNCTIONAL ATP-DEPENDENT DIHYDROXYACETONE KINASE/FAD-AMP LYASE (CYCLIZING).	**1.36**	0.00	1				1		**0.81**	0.00	1	
IPI00552419;IPI00744115;IPI00895869;IPI00952649	PCCA	CDNA FLJ56469, HIGHLY SIMILAR TO PROPIONYL-COA CARBOXYLASE ALPHA CHAIN, MITOCHONDRIAL.;"PROPIONYL-COENZYME A CARBOXYLASE, ALPHA POLYPEPTIDE ISOFORM A PRECURSOR.";"PROPIONYL-COENZYME A CARBOXYLASE, ALPHA POLYPEPTIDE ISOFORM B."			1				1				1	
IPI00552578	SAA1;SAA2	SERUM AMYLOID A PROTEIN.							1				4	
IPI00553177	SERPINA1	ISOFORM 1 OF ALPHA-1-ANTITRYPSIN.	**1.25**	0.21	15	0.367	**2.42**	0.47	18	0.190	**3.99**	0.75	20	0.199
IPI00554474;IPI00946928	SSC5D	SCAVENGER RECEPTOR CYSTEINE-RICH DOMAIN-CONTAINING PROTEIN LOC284297.;SCAVENGER RECEPTOR CYSTEINE-RICH GLYCOPROTEIN.							2					
IPI00554648	KRT8	KERATIN, TYPE II CYTOSKELETAL 8.	**1.13**	0.18	30	0.440	**0.89**	0.15	29	0.310	**0.97**	0.22	32	0.477
IPI00554742;IPI00555572;IPI00555743;IPI00855856;IPI00908668	API5	ISOFORM 2 OF APOPTOSIS INHIBITOR 5.;ISOFORM 1 OF APOPTOSIS INHIBITOR 5.;ISOFORM 3 OF APOPTOSIS INHIBITOR 5.;APOPTOSIS INHIBITOR 5 ISOFORM A.;APOPTOSIS INHIBITOR 5 ISOFORM C.			1									
IPI00554777;IPI00924906;IPI00925572	ASNS	ASPARAGINE SYNTHETASE [GLUTAMINE-HYDROLYZING].;55 KDA PROTEIN.;"CDNA FLJ51387, HIGHLY SIMILAR TO ASPARAGINE SYNTHETASE."	**0.40**	0.00	1		**1.12**	0.00	1				1	
IPI00554788	KRT18	KERATIN, TYPE I CYTOSKELETAL 18.	**1.06**	0.20	25	0.126	**0.67**	0.14	22	0.045	**0.48**	0.15	24	0.311
IPI00554811;IPI00790262;IPI00925052;IPI00925334;IPI00926232	TTLL3;ARPC4;TTLL3	ACTIN-RELATED PROTEIN 2/3 COMPLEX SUBUNIT 4.;SIMILAR TO ACTIN-RELATED PROTEIN 2/3 COMPLEX SUBUNIT 4.;22 KDA PROTEIN.;21 KDA PROTEIN.;13 KDA PROTEIN.	**1.35**	0.14	2	0.334	**1.80**	0.13	2	0.269	**1.95**	0.09	2	0.155
IPI00555812;IPI00954102	GC	VITAMIN D-BINDING PROTEIN.	**0.96**	0.00	2		**2.21**	0.00	1		**2.56**	0.00	3	
IPI00556589;IPI00719622	RPS28	4 KDA PROTEIN.;40S RIBOSOMAL PROTEIN S28.	**0.79**	0.00	2		**0.65**	0.16	2		**0.19**	0.00	2	
IPI00604607	HSP90AA1	HSP89-ALPHA-DELTA-N.				0.369	**1.39**	0.27	9	0.373	**1.32**	0.36	10	0.552
IPI00604664;IPI00925023;IPI00925853;IPI00927225;IPI00927308;IPI00940744	NDUFS1	NADH-UBIQUINONE OXIDOREDUCTASE 75 KDA SUBUNIT.;"CDNA FLJ54000, HIGHLY SIMILAR TO NADH-UBIQUINONE OXIDOREDUCTASE 75 KDA SUBUNIT, MITOCHONDRIAL.";"CDNA FLJ60586, HIGHLY SIMILAR TO NADH-UBIQUINONE OXIDOREDUCTASE 75 KDA SUBUNIT, MITOCHONDRIAL.";"CDNA FLJ55879, HIGHLY SIMILAR TO NADH-UBIQUINONE OXIDOREDUCTASE 75 KDA SUBUNIT, MITOCHONDRIAL.";75 KDA PROTEIN.;"NADH-UBIQUINONE OXIDOREDUCTASE 75 KDA SUBUNIT, MITOCHONDRIAL."			2								1	
IPI00619951;IPI00619958;IPI00796527;IPI00873344;IPI00896474;IPI00941154	TPD52	TUMOR PROTEIN D52 ISOFORM 2.;ISOFORM 1 OF TUMOR PROTEIN D52.;12 KDA PROTEIN.;ISOFORM 3 OF TUMOR PROTEIN D52.;ISOFORM 2 OF TUMOR PROTEIN D52.;PUTATIVE UNCHARACTERIZED PROTEIN TPD52.	**0.74**	0.03	2	0.193			2		**0.46**	0.00	2	
IPI00639945;IPI00647280	PRDX4	PROTEIN.;PUTATIVE UNCHARACTERIZED PROTEIN PRDX4.	**0.76**	0.10	13	0.044	**0.49**	0.09	13	0.019	**0.21**	0.07	11	0.090
IPI00640417;IPI00642238;IPI00645339;IPI00646486	HP1BP3	HETEROCHROMATIN PROTEIN 1, BINDING PROTEIN 3.;ISOFORM 1 OF HETEROCHROMATIN PROTEIN 1-BINDING PROTEIN 3.;39 KDA PROTEIN.;ISOFORM 2 OF HETEROCHROMATIN PROTEIN 1-BINDING PROTEIN 3.	**1.40**	0.15	2	0.349	**1.82**	0.13	2	0.323			2	
IPI00641829;IPI00792043;IPI00848161;IPI00893580	BAT1	ISOFORM 2 OF SPLICEOSOME RNA HELICASE BAT1.;"HCG2005638, ISOFORM CRA_C.";ISOFORM 1 OF SPLICEOSOME RNA HELICASE BAT1.;HLA-B ASSOCIATED TRANSCRIPT 1.	**1.50**	0.11	3	0.185	**1.51**	0.22	3		**0.87**	0.13	3	
IPI00642211	RNPEP	AMINOPEPTIDASE B.	**1.83**	0.07	2		**1.05**	0.10	2		**0.89**	0.25	2	
IPI00643041;IPI00792352;IPI00795671;IPI00796462	RANP1;RAN	GTP-BINDING NUCLEAR PROTEIN RAN.;26 KDA PROTEIN.;"RAN, MEMBER RAS ONCOGENE FAMILY, ISOFORM CRA_C.";27 KDA PROTEIN.	**1.59**	0.22	2		**0.94**	0.10	2		**1.05**	0.12	3	
IPI00643920;IPI00792641;IPI00940673;IPI00942979	TKT	CDNA FLJ54957, HIGHLY SIMILAR TO TRANSKETOLASE.;TRANSKETOLASE ISOFORM 2.;"CDNA FLJ53217, HIGHLY SIMILAR TO TRANSKETOLASE.";TRANSKETOLASE.	**0.90**	0.10	13	0.237	**0.78**	0.08	13	0.025	**0.39**	0.02	13	0.022
IPI00644127;IPI00910980	IARS	ISOLEUCYL-TRNA SYNTHETASE, CYTOPLASMIC.;IARS PROTEIN.			1				1				1	
IPI00644712;IPI00893062;IPI00893179	XRCC6	ATP-DEPENDENT DNA HELICASE 2 SUBUNIT 1.;X-RAY REPAIR COMPLEMENTING DEFECTIVE REPAIR IN CHINESE HAMSTER CELLS 6.	**1.15**	0.07	5	0.392	**1.12**	0.13	4	0.609	**0.88**	0.09	4	0.563
IPI00645078	UBA1	UBIQUITIN-LIKE MODIFIER-ACTIVATING ENZYME 1.	**1.16**	0.05	9	0.193	**1.48**	0.18	9	0.350	**0.95**	0.08	9	0.512
IPI00645425;IPI00874075	CRYZ	CRYSTALLIN, ZETA ISOFORM C.;PUTATIVE UNCHARACTERIZED PROTEIN CRYZ (FRAGMENT).	**1.11**	0.13	3	0.459	**0.87**	0.00	3				3	
IPI00645452;IPI00908770;IPI00909140	TUBB	TUBULIN, BETA.;"CDNA FLJ53063, HIGHLY SIMILAR TO TUBULIN BETA-7 CHAIN.";"CDNA FLJ56903, HIGHLY SIMILAR TO TUBULIN BETA-7 CHAIN."	**1.23**	0.13	12		**2.06**	0.35	14		**1.88**	0.39	11	
IPI00645898;IPI00793375	XPNPEP1	X-PROLYL AMINOPEPTIDASE (AMINOPEPTIDASE P) 1, SOLUBLE.;XAA-PRO AMINOPEPTIDASE 1.	**1.08**	0.00	3		**0.87**	0.00	3				3	
IPI00646304	PPIB	PEPTIDYL-PROLYL CIS-TRANS ISOMERASE B.	**0.74**	0.09	9	0.096	**0.57**	0.09	9	0.069	**0.33**	0.05	9	0.043
IPI00646689;IPI00789285;IPI00797323	TXNDC17	THIOREDOXIN DOMAIN-CONTAINING PROTEIN 17.;11 KDA PROTEIN.;9 KDA PROTEIN.	**1.19**	0.00	1		**0.74**	0.00	1		**0.69**	0.00	1	
IPI00647650;IPI00789224;IPI00792326;IPI00793882;IPI00941359	EIF3H	EUKARYOTIC TRANSLATION INITIATION FACTOR 3, SUBUNIT 3 GAMMA, 40KDA, ISOFORM CRA_B.;6 KDA PROTEIN.;5 KDA PROTEIN.;11 KDA PROTEIN.;EUKARYOTIC TRANSLATION INITIATION FACTOR 3 SUBUNIT H.			1				1				1	
IPI00647896	TUBB	TUBULIN, BETA.	**1.21**	0.11	14	0.126	**1.41**	0.22	16	0.348	**1.22**	0.20	14	0.671
IPI00651653;IPI00651677;IPI00879638;IPI00889541;IPI00940000;IPI00947363;IPI00952778	DDX17	ISOFORM 3 OF PROBABLE ATP-DEPENDENT RNA HELICASE DDX17.;ISOFORM 2 OF PROBABLE ATP-DEPENDENT RNA HELICASE DDX17.;"CDNA FLJ58652, HIGHLY SIMILAR TO PROBABLE ATP-DEPENDENT RNA HELICASE DDX17.";ISOFORM 4 OF PROBABLE ATP-DEPENDENT RNA HELICASE DDX17.;DEAD BOX POLYPEPTIDE 17 ISOFORM 3.;73 KDA PROTEIN.	**1.24**	0.06	2	0.441			2		**1.24**	0.00	2	
IPI00654755	HBB	HEMOGLOBIN SUBUNIT BETA.	**1.43**	0.23	18	0.110	**2.77**	0.70	19	0.090	**1.36**	0.38	21	0.473
IPI00654777;IPI00941255	EIF3F	HCG1784554, ISOFORM CRA_A.;EUKARYOTIC TRANSLATION INITIATION FACTOR 3 SUBUNIT F.	**1.19**	0.00	1		**0.97**	0.00	2		**0.44**	0.00	1	
IPI00655812	LMNA	RHABDOMYOSARCOMA ANTIGEN MU-RMS-40.12.	**2.09**	0.33	27	0.000	**2.89**	0.47	25	0.002	**1.92**	0.33	27	0.045
IPI00657644	PDIA2	PROTEIN DISULFIDE ISOMERASE FAMILY A, MEMBER 2.	**0.77**	0.13	13	0.064	**0.42**	0.13	13	0.097	**0.07**	0.00	14	
IPI00740336;IPI00889693	FMN1	FORMIN 1.;ISOFORM 3 OF FORMIN-1.	**0.20**	0.00	2		**0.25**	0.00	2				2	
IPI00742682	TPR	NUCLEOPROTEIN TPR.	**1.18**	0.00	1		**1.05**	0.00	1		**0.48**	0.00	1	
IPI00743696;IPI00873684	COL4A1	PUTATIVE UNCHARACTERIZED PROTEIN COL4A1.;ISOFORM 2 OF COLLAGEN ALPHA-1(IV) CHAIN.	**0.95**	0.18	3		**1.98**	0.24	3		**1.12**	0.00	2	

IPI00744692	TALDO1	TRANSALDOLASE.	**0.76**	0.13	2	0.426	**1.28**	0.06	2		**1.08**	0.08	2	
IPI00745313;IPI00894097	AEBP1	ISOFORM 1 OF ADIPOCYTE ENHANCER-BINDING PROTEIN 1.;ISOFORM 2 OF ADIPOCYTE ENHANCER-BINDING PROTEIN 1.											1	
IPI00745872;IPI00878282	ALB	ISOFORM 1 OF SERUM ALBUMIN.;23 KDA PROTEIN.	**1.95**	0.72	18		**3.58**	0.85	17		**4.16**	0.71	17	
IPI00746165;IPI00873622;IPI00908482	WDR1	ISOFORM 1 OF WD REPEAT-CONTAINING PROTEIN 1.;PUTATIVE UNCHARACTERIZED PROTEIN WDR1.;"CDNA FLJ57081, MODERATELY SIMILAR TO WD REPEAT PROTEIN 1."											1	
IPI00783097;IPI00915808	GARS	GLYCYL-TRNA SYNTHETASE.;PUTATIVE UNCHARACTERIZED PROTEIN GARS.	**0.78**	0.05	4	0.157	**0.78**	0.00	3		**0.19**	0.00	3	
IPI00783271	LRPPRC	LEUCINE-RICH PPR MOTIF-CONTAINING PROTEIN, MITOCHONDRIAL.	**0.67**	0.13	3		**0.82**	0.00	3		**0.33**	0.00	3	
IPI00783665	LAMA5	LAMININ SUBUNIT ALPHA-5.	**1.64**	0.35	12	0.447	**2.06**	0.41	11	0.277	**0.96**	0.24	9	0.685
IPI00783982	COPG	COATOMER SUBUNIT GAMMA.	**1.00**	0.13	4	0.384	**0.95**	0.13	4		**0.48**	0.05	5	
IPI00783987	C3	COMPLEMENT C3 (FRAGMENT).	**2.36**	0.36	18	0.076	**3.54**	0.40	21	0.028	**4.63**	0.78	23	0.015
IPI00784044;IPI00789428	MCCC2	ISOFORM 1 OF METHYLCROTONOYL-COA CARBOXYLASE BETA CHAIN, MITOCHONDRIAL.;"ISOFORM 2 OF METHYLCROTONOYL-COA CARBOXYLASE BETA CHAIN, MITOCHONDRIAL."	**1.22**	0.00	1		**1.36**	0.00	1				1	
IPI00784154;IPI00917575	HSPD1	60 KDA HEAT SHOCK PROTEIN, MITOCHONDRIAL.;"CDNA FLJ51046, HIGHLY SIMILAR TO 60 KDA HEAT SHOCK PROTEIN, MITOCHONDRIAL."	**1.07**	0.16	9		**0.96**	0.26	9	0.384	**0.45**	0.04	10	0.318
IPI00784156;IPI00784366;IPI00790702	AP2B1	ISOFORM 1 OF AP-2 COMPLEX SUBUNIT BETA.;ISOFORM 2 OF AP-2 COMPLEX SUBUNIT BETA.;"ADAPTOR-RELATED PROTEIN COMPLEX 2, BETA 1 SUBUNIT, ISOFORM CRA_F."			2								2	
IPI00789094	PDIA2	CDNA FLJ42941 FIS, CLONE BRSTN2000872, HIGHLY SIMILAR TO PROTEIN DISULFIDE-ISOMERASE A2.	**0.68**	0.14	18	0.003				0.055				
IPI00791350;IPI00792115	CLEC3B	11 KDA PROTEIN.;PUTATIVE UNCHARACTERIZED PROTEIN DKFZP686H17246.							1				1	
IPI00791534;IPI00926256	SLC4A1	104 KDA PROTEIN.;BAND 3 ANION TRANSPORT PROTEIN.	**2.21**	0.07	5	0.193	**3.52**	0.24	8	0.515	**1.04**	0.07	4	0.708
IPI00793199;IPI00872780	ANXA4	ANNEXIN IV.;ANNEXIN A4.	**1.14**	0.11	20	0.374	**0.97**	0.14	16	0.483	**0.38**	0.06	18	0.028
IPI00793874;IPI00871988;IPI00942178	SFXN3	SIDEROFLEXIN 3.;"CDNA FLJ58980, HIGHLY SIMILAR TO SIDEROFLEXIN-3."											2	
IPI00794644	KRT19	21 KDA PROTEIN.				0.075	**2.68**	0.59	9	0.143	**4.79**	2.00	15	0.102
IPI00797556	ANXA2	24 KDA PROTEIN.	**2.11**	0.16	18	0.001	**3.39**	0.36	23	0.000	**3.59**	0.47	23	0.000
IPI00798401;IPI00867509;IPI00943173	CORO1C	CDNA FLJ50992, HIGHLY SIMILAR TO CORONIN-1C.;CORONIN-1C_I3 PROTEIN.;CORONIN-1C.											1	
IPI00843765;IPI00844215;IPI00871535;IPI00879810	SPTAN1	ISOFORM 3 OF SPECTRIN ALPHA CHAIN, BRAIN.;"ISOFORM 1 OF SPECTRIN ALPHA CHAIN, BRAIN.";"ISOFORM 2 OF SPECTRIN ALPHA CHAIN, BRAIN.";PUTATIVE UNCHARACTERIZED PROTEIN SPTAN1.	**1.39**	0.16	12	0.007	**1.51**	0.21	12	0.022	**1.13**	0.15	12	0.531
IPI00843975;IPI00872684	EZR	EZRIN.;69 KDA PROTEIN.	**1.27**	0.10	3	0.439	**1.50**	0.15	5	0.485	**1.85**	0.26	7	0.400
IPI00844000	KIAA0776	UPF0555 PROTEIN KIAA0776.			1				1				1	
IPI00844578	DHX9	ATP-DEPENDENT RNA HELICASE A.	**1.16**	0.00	3				2				1	
IPI00848226	GNB2L1	GUANINE NUCLEOTIDE-BINDING PROTEIN SUBUNIT BETA-2-LIKE 1.	**0.84**	0.03	3	0.147	**0.41**	0.01	3	0.062	**0.18**	0.00	3	
IPI00871809	IQGAP2	ISOFORM 2 OF RAS GTPASE-ACTIVATING-LIKE PROTEIN IQGAP2.	**1.37**	0.16	4				3				3	
IPI00872508;IPI00876962;IPI00878985;IPI00895800	INF2	PUTATIVE UNCHARACTERIZED PROTEIN INF2.;ISOFORM 2 OF INVERTED FORMIN-2.;ISOFORM 1 OF INVERTED FORMIN-2.											1	
IPI00877852;IPI00953675	ITIH1	52 KDA PROTEIN.											4	
IPI00878517	ALB	PUTATIVE UNCHARACTERIZED PROTEIN ALB.	**1.85**	0.72	37	0.355	**3.82**	1.06	35	0.001	**4.52**	0.98	37	0.015
IPI00884105	LAMP1	LYSOSOME-ASSOCIATED MEMBRANE GLYCOPROTEIN 1.	**1.17**	0.17	3	0.507	**1.18**	0.06	3	0.626	**0.91**	0.08	3	0.704
IPI00887169	LOC100293440;IGLC3;IGLC2;IGL@;LOC100293277;LOC100290557;IGLC1;LOC100290481;IGLV2-14;IGLV1-40;IGLV1-44;IGLV3-21;IGLV2-11	PUTATIVE UNCHARACTERIZED PROTEIN.	**2.08**	0.06	4	0.262	**4.00**	0.54	5		**4.77**	0.19	5	0.197
IPI00888126	LOC652797	SIMILAR TO PYRUVATE KINASE, MUSCLE.				0.252				0.092	**2.00**	0.44	11	0.109
IPI00903334	ANXA2	CDNA FLJ34687 FIS, CLONE MESAN2000620, HIGHLY SIMILAR TO ANNEXIN A2.				0.202	**2.97**	0.89	11	0.067	**3.26**	1.00	11	0.078
IPI00908876	ALB	CDNA FLJ50830, HIGHLY SIMILAR TO SERUM ALBUMIN.	**1.91**	0.72	39	0.324	**3.65**	1.03	37	0.001	**4.37**	0.95	38	0.010
IPI00909509		CDNA FLJ59138, HIGHLY SIMILAR TO ANNEXIN A2.	**2.14**	0.19	14	0.008	**3.54**	0.40	15	0.001	**3.73**	0.51	18	0.001
IPI00911047		CDNA FLJ58131, HIGHLY SIMILAR TO SECRETOGRANIN-1.					**1.25**	0.00	2					
IPI00915869;IPI00916111;IPI00916861	MDH1	MALATE DEHYDROGENASE.;PUTATIVE UNCHARACTERIZED PROTEIN MDH1.	**1.07**	0.00	1	0.376	**1.00**	0.00	1	0.604	**0.57**	0.00	1	0.353
IPI00916345;IPI00935211;IPI00935986;IPI00938042	HSPE1;LOC100292829;LOC100287159;LOC100290437;LOC100292290	PUTATIVE UNCHARACTERIZED PROTEIN HSPE1.;HYPOTHETICAL PROTEIN XP_002342349.;HYPOTHETICAL PROTEIN XP_002346492.;SIMILAR TO HEAT SHOCK 10KDA PROTEIN 1.	**0.43**	0.00	2		**0.57**	0.00	2		**0.46**	0.00	2	
IPI00922127	HYOU1	CDNA FLJ54708, HIGHLY SIMILAR TO 150 KDA OXYGEN-REGULATED PROTEIN.	**1.13**	0.13	13	0.417	**0.70**	0.11	13	0.125	**0.22**	0.03	13	0.114
IPI00922744;IPI00935601	C4B;LOC100293534	COMPLEMENT PROTEIN C4B FRAMESHIFT MUTANT (FRAGMENT).;"SIMILAR TO COMPLEMENT COMPONENT 4B (CHILDO BLOOD GROUP), PARTIAL."								0.175			1	0.179
IPI00925804;IPI00953925	AIP	NON-FUNCTIONAL ARYL HYDROCARBON RECEPTOR INTERACTING PROTEIN (FRAGMENT).							1				1	
IPI00936387	RPN2	RIBOPHORIN II, ISOFORM CRA_D.	**0.67**	0.11	8	0.177	**0.48**	0.09	8	0.076	**0.21**	0.03	8	0.153
IPI00939159	CAP1	ADENYLYL CYCLASE-ASSOCIATED PROTEIN.	**1.27**	0.00	3		**2.96**	0.27	3	0.208	**1.89**	0.43	3	0.150
IPI00940084	TPM1	37 KDA PROTEIN.				0.034				0.087	**4.62**	1.22	9	0.080
IPI00941465	COL6A3	325 KDA PROTEIN.				0.000	**4.82**	0.75	78	0.000	**3.82**	0.83	74	0.000
IPI00941764	RPN2	RIBOPHORIN II.	**0.67**	0.11	8	0.177	**0.48**	0.09	8	0.076	**0.21**	0.03	8	0.153
