# Supplementary material for: Proteomics Portrait of Archival Lesions of Chronic Pancreatitis
Source: PLoS One. 2011 Nov 23;6(11):e27574. doi: 10.1371/journal.pone.0027574 (PMC3223181; doi:10.1371/journal.pone.0027574)
Supplement: Table S3 — The proteins with ≥2-fold change in abundance in mild chronic pancreatitis (MCP), severe chronic pancreatitis (SCP) and pancreatic ductal adenocarcinoma (PDAC) compared to normal pancreas (NL). (PDF) [file pone.0027574.s005.pdf]

Supplemental table 3. The proteins with ≥2-fold change in abundance in mild chronic pancreatitis (MCP), severe chronic pancreatitis (SCP) and pancreatic ductal adenocarcinoma (PDAC) compared to normal pancreas (NL)

* protein groups with multiple identifications - the first identification is listed

IPI number	gene symbol	protein	MCP/NL ratio	MCP/NL stdev	SCP/NL ratio	SCP/NL stdev	PDAC/NL ratio	PDAC/NL stdev	Glyco-protein	Concurrent with our previous studies in cancer or PanIN? (ref 8, 17, 22)	Concurrent with our previous studies in CP? (Ref 8, 22)
IPI00009532	ABAT	CDNA FLJ56034, HIGHLY SIMILAR TO 4-AMINOBUTYRATE AMINOTRANSFERASE, MITOCHONDRIAL.	**0.74**	0.13	**0.41**	0.11	**0.09**	0.00		down in cancer (iTRAQ)	down (iTRAQ)
*IPI00005040	ACADM	MEDIUM-CHAIN SPECIFIC ACYL-COA DEHYDROGENASE, MITOCHONDRIAL.	**0.78**	0.07	**0.73**	0.05	**0.35**	0.00			
*IPI00028031	ACADVL	CDNA FLJ56425, HIGHLY SIMILAR TO VERY-LONG-CHAIN SPECIFIC ACYL-COADEHYDROGENASE, MITOCHONDRIAL.	**1.05**	0.12	**0.85**	0.06	**0.42**	0.04			
IPI00030363	ACAT1	ACETYL-COA ACETYLTRANSFERASE, MITOCHONDRIAL.	**0.84**	0.10	**0.53**	0.08	**0.21**	0.05		down in cancer (iTRAQ)	down (iTRAQ)
IPI00008485	ACO1	CYTOPLASMIC ACONITATE HYDRATASE.	**0.81**	0.06	**0.79**	0.07	**0.23**	0.00		down in PanIN/cancer (iTRAQ)	
*IPI00017855	ACO2	ACONITASE 2, MITOCHONDRIAL.	**0.91**	0.05	**1.04**	0.20	**0.44**	0.03		down in PanIN/cancer (iTRAQ)	
*IPI00021439	*ACTB	ACTIN, CYTOPLASMIC 1.	**1.83**	0.24	**2.40**	0.29	**2.42**	0.35		up in cancer (iTRAQ/ICAT)	
*IPI00013508	ACTN1	ALPHA-ACTININ-1.	**1.69**	0.38	**1.66**	0.44	**2.05**	0.57		up in cancer (iTRAQ)	
*IPI00218896	*ADH1A	ALCOHOL DEHYDROGENASE 1A.	**1.60**	0.11	**2.09**	0.12	**0.80**	0.07			up (iTRAQ)
*IPI00007427	AGR2	ANTERIOR GRADIENT PROTEIN 2.	**3.94**	1.17	**1.24**	0.00	**4.04**	0.31		up in cancer (iTRAQ)	
IPI00374563	AGRN	AGRIN.	**0.88**	0.00	**0.71**	0.00	**0.49**	0.00	Yes		
IPI00021812	AHNAK	NEUROBLAST DIFFERENTIATION-ASSOCIATED PROTEIN AHNAK.	**2.67**	0.38	**4.39**	0.65	**2.82**	0.37		up in PanIN/cancer (iTRAQ)	up (iTRAQ)
IPI00030706	AHSA1	ACTIVATOR OF 90 KDA HEAT SHOCK PROTEIN ATPASE HOMOLOG 1.	**0.28**	0.00	**0.32**	0.00	**0.26**	0.00			
*IPI00011916	*AIMP2	AMINOACYL TRNA SYNTHETASE COMPLEX-INTERACTING MULTIFUNCTIONAL PROTEIN 2.	**1.34**	0.00	**0.96**	0.00	**0.24**	0.00			
*IPI00215901	AK2	ISOFORM 1 OF ADENYLATE KINASE 2, MITOCHONDRIAL.	**1.12**	0.00	**1.05**	0.00	**0.45**	0.00			
IPI00220271	AKR1A1	ALCOHOL DEHYDROGENASE [NADP+].	**0.80**	0.04	**0.67**	0.12	**0.47**	0.03			
IPI00293721	AKR7A3	AFLATOXIN B1 ALDEHYDE REDUCTASE MEMBER 3.	**0.47**	0.00			**0.22**	0.00			
IPI00216773	ALB	ALB PROTEIN.	**1.74**	0.62	**3.22**	0.89	**4.18**	0.91	Yes	up in cancer (iTRAQ/ICAT)	up (iTRAQ/ICAT)
*IPI00217920	ALDH16A1	ISOFORM 1 OF ALDEHYDE DEHYDROGENASE FAMILY 16 MEMBER A1.	**1.13**	0.02	**0.65**	0.00	**0.43**	0.00			
*IPI00008982	ALDH18A1	ISOFORM LONG OF DELTA-1-PYRROLINE-5-CARBOXYLATE SYNTHETASE.	**2.05**	0.00			**0.76**	0.00			up (iTRAQ)
IPI00218914	ALDH1A1	RETINAL DEHYDROGENASE 1.	**0.91**	0.07	**0.82**	0.11	**0.29**	0.09		down in PanIN/cancer (iTRAQ/ICAT)	
IPI00103467	ALDH1B1	ALDEHYDE DEHYDROGENASE X, MITOCHONDRIAL.	**2.18**	0.00	**1.04**	0.00	**0.52**	0.00			
IPI00298308	ALDH1L2	ISOFORM 1 OF PROBABLE 10-FORMYLTETRAHYDROFOLATE DEHYDROGENASE ALDH1L2.	**0.70**	0.04	**0.87**	0.12	**0.19**	0.00			
IPI00024990	ALDH6A1	METHYLMALONATE-SEMIALDEHYDE DEHYDROGENASE [ACYLATING], MITOCHONDRIAL.	**1.11**	0.11	**0.37**	0.03				down in PanIN/cancer (iTRAQ)	down (iTRAQ)
IPI00479877	ALDH9A1	ALDEHYDE DEHYDROGENASE 9A1.	**1.02**	0.00	**1.08**	0.00	**0.39**	0.00		down in cancer (iTRAQ)	
*IPI00465439	ALDOA	FRUCTOSE-BISPHOSPHATE ALDOLASE A.	**1.09**	0.04	**0.38**	0.27	**1.24**	0.40			
IPI00025476	*AMY2A	PANCREATIC ALPHA-AMYLASE.	**0.86**	0.15	**0.32**	0.12	**0.02**	0.00	Yes		
IPI00021447	AMY2B	ALPHA-AMYLASE 2B.	**0.90**	0.20	**0.38**	0.14	**0.03**	0.01			down (iTRAQ)
IPI00218918	ANXA1	ANNEXIN A1.	**2.03**	0.26	**3.79**	0.41	**4.36**	0.31		up in cancer (iTRAQ/ICAT)	
*IPI00418169	ANXA2	ISOFORM 2 OF ANNEXIN A2.	**1.96**	0.29	**3.09**	0.60	**3.28**	0.66		up in cancer (iTRAQ/ICAT)	

IPI00334627	ANXA2P2	PUTATIVE ANNEXIN A2-LIKE PROTEIN.	**1.86**	0.32	**3.06**	0.70	**3.21**	0.75			
*IPI00793199	ANXA4	ANNEXIN A4.	**1.14**	0.11	**0.97**	0.14	**0.38**	0.06			
IPI00022391	APCS	SERUM AMYLOID P-COMPONENT.	**1.68**	0.06	**3.40**	0.82	**1.60**	0.21	Yes		
IPI00021841	APOA1	APOLIPOPROTEIN A-I.	**1.22**	0.20	**2.70**	0.68	**1.38**	0.42	Yes		up (iTRAQ)
IPI00021854	APOA2	APOLIPOPROTEIN A-II.	**0.89**	0.10	**5.08**	0.00	**4.93**	0.51			up (iTRAQ/ICAT)
*IPI00298828	APOH	BETA-2-GLYCOPROTEIN 1.	**3.17**	0.00	**3.88**	0.00	**2.80**	0.00	Yes	up in cancer (iTRAQ/ICAT)	up (iTRAQ/ICAT)
*IPI00218693	APRT	ADENINE PHOSPHORIBOSYLTRANSFERASE.	**0.93**	0.04	**0.82**	0.15	**0.49**	0.02			
*IPI00024689	*AQP1	AQUAPORIN-1.	**0.90**	0.00	**0.41**	0.00			Yes		
*IPI00003817	ARHGDIB	RHO GDP-DISSOCIATION INHIBITOR 2.	**1.29**	0.00			**2.20**	0.00			
*IPI00554777	ASNS	ASPARAGINE SYNTHETASE [GLUTAMINE-HYDROLYZING].	**0.40**	0.00	**1.12**	0.00					
*IPI00295992	*ATAD3A	ATPASE FAMILY, AAA DOMAIN CONTAINING 3A.	**0.58**	0.00	**0.41**	0.00					
*IPI00177817	ATP2A2	ISOFORM SERCA2A OF SARCOPLASMIC/ENDOPLASMIC RETICULUM CALCIUM ATPASE 2.	**1.03**	0.17	**0.61**	0.00	**0.46**	0.00			
IPI00440493	ATP5A1	ATP SYNTHASE SUBUNIT ALPHA, MITOCHONDRIAL.	**0.97**	0.11	**0.78**	0.10	**0.48**	0.10			
IPI00303476	ATP5B	ATP SYNTHASE SUBUNIT BETA, MITOCHONDRIAL.	**1.02**	0.10	**0.78**	0.10	**0.45**	0.05		down in cancer (iTRAQ)	
IPI00007611	ATP5O	ATP SYNTHASE SUBUNIT O, MITOCHONDRIAL.	**1.10**	0.07	**0.80**	0.09	**0.40**	0.00			
*IPI00010790	BGN	BIGLYCAN.					**7.87**	3.39	Yes	up in cancer (iTRAQ/ICAT)	down (ICAT)
IPI00014230	C1QBP	COMPLEMENT COMPONENT 1 Q SUBCOMPONENT-BINDING PROTEIN, MITOCHONDRIAL.	**1.00**	0.02	**6.13**	0.00	**1.21**	0.00			
IPI00783987	C3	COMPLEMENT C3 (FRAGMENT).	**2.36**	0.36	**3.54**	0.40	**4.63**	0.78	Yes		up (ICAT)
*IPI00032258	*C4A	COMPLEMENT C4-A.	**1.54**	0.44	**3.36**	0.88	**3.40**	0.43	Yes	up in cancer (iTRAQ)	
IPI00007926	C6orf108	DEOXYRIBONUCLEOSIDE 5'-MONOPHOSPHATE N-GLYCOSIDASE.	**0.82**	0.00	**0.38**	0.00	**0.23**	0.00			
*IPI00001734	*C8orf62	PHOSPHOSERINE AMINOTRANSFERASE.	**0.73**	0.15	**0.67**	0.03	**0.27**	0.00		down in PanIN/cancer (iTRAQ)	
IPI00022395	C9	COMPLEMENT COMPONENT C9.	**1.18**	0.00	**2.41**	0.00	**1.48**	0.00	Yes		
IPI00215983	CA1	CARBONIC ANHYDRASE 1.	**1.59**	0.24	**5.30**	0.76	**2.33**	0.19			up (iTRAQ)
*IPI00014516	CALD1	ISOFORM 1 OF CALDESMON.	**2.73**	0.55	**4.73**	0.67	**6.99**	0.87		up in cancer (iTRAQ)	
*IPI00075248	*CALM3	CALMODULIN.	**2.14**	0.64	**1.61**	0.25	**1.82**	0.38			
IPI00020599	CALR	CALRETICULIN.	**0.98**	0.14	**0.84**	0.10	**0.46**	0.00	Yes	down in PanIN/cancer (iTRAQ)	
IPI00292532	CAMP	CATHELICIDIN ANTIMICROBIAL PEPTIDE PRECURSOR.	**0.47**	0.00	**1.73**	0.00	**0.94**	0.00			
IPI00939159	CAP1	ADENYLYL CYCLASE-ASSOCIATED PROTEIN.	**1.27**	0.00	**2.96**	0.27	**1.89**	0.43			
*IPI00027341	CAPG	MACROPHAGE-CAPPING PROTEIN.	**2.35**	0.00	**1.95**	0.40	**2.92**	1.38			
IPI00465436	CAT	CATALASE.	**1.88**	0.14	**3.08**	0.36	**1.77**	0.22			up (iCAT)
IPI00297779	CCT2	T-COMPLEX PROTEIN 1 SUBUNIT BETA.	**1.22**	0.10	**0.96**	0.00	**0.49**	0.00			
*IPI00010720	CCT5	T-COMPLEX PROTEIN 1 SUBUNIT EPSILON.	**1.34**	0.08	**1.22**	0.10	**0.49**	0.03			
*IPI00099670	CEL	CARBOXYL ESTER LIPASE PRECURSOR.	**0.67**	0.09	**0.35**	0.11	**0.03**	0.01	Yes	down in cancer (iTRAQ/ICAT)	
*IPI00027722	CELA2A	CHYMOTRYPSIN-LIKE ELASTASE FAMILY MEMBER 2A.	**0.44**	0.11	**0.33**	0.13	**0.16**	0.11		down in cancer (iTRAQ/ICAT)	
*IPI00027723	CELA2B	CHYMOTRYPSIN-LIKE ELASTASE FAMILY MEMBER 2B.	**0.74**	0.12	**0.61**	0.18	**0.16**	0.11		down in cancer (ICAT)	
IPI00295663	CELA3A	CHYMOTRYPSIN-LIKE ELASTASE FAMILY MEMBER 3A.	**0.63**	0.12	**0.28**	0.09	**0.04**	0.01	Yes	down in cancer (ICAT)	
IPI00290315	CHGA	CHROMOGRANIN-A.			**2.64**	0.39			Yes		
IPI00006601	CHGB	SECRETOGRANIN-1.	**2.88**	0.00	**2.60**	0.83			Yes		
*IPI00301185	CHID1	ISOFORM 2 OF CHITINASE DOMAIN-CONTAINING PROTEIN 1.	**1.01**	0.09	**0.37**	0.00					
*IPI00291262	CLU	ISOFORM 1 OF CLUSTERIN.	**1.34**	0.27	**3.39**	0.24	**1.35**	0.06	Yes		
IPI00021264	CNN1	CALPONIN-1.					**4.31**	0.00			
*IPI00176193	COL14A1	ISOFORM 1 OF COLLAGEN ALPHA-1(XIV) CHAIN.	**6.95**	1.32	**16.39**	3.13	**11.38**	1.87	Yes	up in cancer (iTRAQ/ICAT)	up (iTRAQ/ICAT)
IPI00295414	COL15A1	COLLAGEN ALPHA-1(XV) CHAIN.	**1.49**	0.09	**2.65**	0.26	**1.45**	0.02	Yes		
IPI00297646	COL1A1	COLLAGEN ALPHA-1(I) CHAIN.	**6.85**	1.26	**13.48**	2.76	**9.54**	2.53	Yes	up in cancer (iTRAQ)	
IPI00304962	COL1A2	COLLAGEN ALPHA-2(I) CHAIN.	**5.43**	2.55	**8.26**	5.25	**6.64**	2.70	Yes	up in cancer (iTRAQ)	

IPI00021033	COL3A1	ISOFORM 1 OF COLLAGEN ALPHA-1(III) CHAIN.	**8.17**	0.45	**14.99**	1.68	**11.44**	1.28	Yes		
*IPI00306322	COL4A2	COLLAGEN ALPHA-2(IV) CHAIN.	**1.65**	0.25	**2.13**	0.40	**0.85**	0.30	Yes		
IPI00291136	COL6A1	COLLAGEN ALPHA-1(VI) CHAIN.	**3.98**	0.66	**6.71**	0.67	**4.55**	1.07	Yes		
*IPI00304840	COL6A2	ISOFORM 2C2 OF COLLAGEN ALPHA-2(VI) CHAIN.	**3.60**	0.65	**5.06**	0.98	**4.16**	0.71	Yes		up (ICAT)
*IPI00022200	COL6A3	ISOFORM 1 OF COLLAGEN ALPHA-3(VI) CHAIN.	**4.04**	0.62	**4.82**	0.75	**3.82**	0.83	Yes	up in cancer (iTRAQ)	up (iTRAQ)
*IPI00295857	COPA	ISOFORM 1 OF COATOMER SUBUNIT ALPHA.	**0.94**	0.07	**0.94**	0.03	**0.45**	0.04			
IPI00220219	COPB2	COATOMER SUBUNIT BETA.	**0.89**	0.00	**0.93**	0.00	**0.31**	0.00			
IPI00783982	COPG	COATOMER SUBUNIT GAMMA.	**1.00**	0.13	**0.95**	0.13	**0.48**	0.05			
*IPI00010133	CORO1A	CORONIN-1A.			**2.71**	0.00	**1.65**	0.00			
IPI00017704	COTL1	COACTOSIN-LIKE PROTEIN.	**0.47**	0.00	**2.03**	0.00	**1.40**	0.00			
*IPI00006579	COX4I1	CYTOCHROME C OXIDASE SUBUNIT 4 ISOFORM 1, MITOCHONDRIAL.	**0.62**	0.00	**0.23**	0.00	**0.55**	0.00			
*IPI00026570	COX7A2	CYTOCHROME C OXIDASE POLYPEPTIDE 7A2, MITOCHONDRIAL.	**0.96**	0.00	**1.07**	0.00	**0.46**	0.00			
IPI00009823	CPA1	CARBOXYPEPTIDASE A1.	**0.58**	0.09	**0.19**	0.04	**0.04**	0.01		down in PanIN/cancer (iTRAQ/ICAT)	down (iTRAQ)
*IPI00296930	CPA2	CARBOXYPEPTIDASE A2 (PANCREATIC) PRECURSOR.	**0.73**	0.10	**0.27**	0.04	**0.04**	0.00		down in cancer (iTRAQ/ICAT)	down (iTRAQ)
IPI00009829	CPA3	MAST CELL CARBOXYPEPTIDASE A.	**0.89**	0.00	**2.58**	0.00					
IPI00009826	CPB1	CARBOXYPEPTIDASE B.	**0.61**	0.07	**0.35**	0.08	**0.04**	0.01		down in PanIN/cancer (iTRAQ/ICAT)	down (iTRAQ)
IPI00031121	CPE	CARBOXYPEPTIDASE E PRECURSOR.	**3.85**	0.00	**3.01**	0.00			Yes		
IPI00442073	CSRP1	CYSTEINE AND GLYCINE-RICH PROTEIN 1.	**1.32**	0.00	**1.93**	0.00	**2.25**	0.00		up in cancer (iTRAQ/ICAT)	
*IPI00515087	CTRB2	CHYMOTRYPSINOGEN B2.	**0.83**	0.11	**0.53**	0.09	**0.04**	0.00		down in cancer (iTRAQ)	
*IPI00018553	CTRC	CHYMOTRYPSIN-C.	**0.92**	0.23	**0.44**	0.09	**0.03**	0.00	Yes	down in cancer (iTRAQ)	down (iTRAQ)
*IPI00027947	CTRL	CHYMOTRYPSIN-LIKE PROTEASE CTRL-1.	**0.80**	0.10	**0.44**	0.01			Yes	down in cancer (ICAT)	
*IPI00295741	CTSB	CATHEPSIN B.	**1.26**	0.00	**2.19**	0.00	**3.33**	0.00	Yes	up in cancer (ICAT)	
IPI00028064	CTSG	CATHEPSIN G.	**1.85**	0.00			**2.35**	0.00	Yes		
*IPI00249672	CUZD1	ISOFORM 1 OF CUB AND ZONA PELLUCIDA-LIKE DOMAIN-CONTAINING PROTEIN 1.			**0.44**	0.00			Yes		
IPI00029264	CYC1	CYTOCHROME C1, HEME PROTEIN, MITOCHONDRIAL.	**0.78**	0.00	**0.56**	0.00	**0.31**	0.00			
IPI00009407	DAD1	DOLICHYL-DIPHOSPHOOLIGOSACCHARIDE--PROTEIN GLYCOSYLTRANSFERASE SUBUNIT DAD1.	**0.98**	0.00	**0.73**	0.00	**0.39**	0.00			
IPI00012119	DCN	ISOFORM A OF DECORIN.	**4.46**	0.23	**7.13**	0.17	**4.52**	0.33	Yes	up in cancer (iTRAQ)	up (iTRAQ)
IPI00297084	DDOST	DOLICHYL-DIPHOSPHOOLIGOSACCHARIDE--PROTEIN GLYCOSYLTRANSFERASE 48 KDA SUBUNIT.	**0.75**	0.04	**0.35**	0.00	**0.16**	0.00			
*IPI00028387	DDRGK1	ISOFORM 1 OF DDRGK DOMAIN-CONTAINING PROTEIN 1.	**0.85**	0.06	**0.60**	0.05	**0.15**	0.00		down in cancer (iTRAQ)	down (iTRAQ)
*IPI00293867	DDT	D-DOPACHROME DECARBOXYLASE.	**0.49**	0.01	**0.44**	0.01	**0.19**	0.01		down in PanIN/cancer (iTRAQ/ICAT)	
IPI00465084	DES	DESMIN.	**2.67**	0.26	**3.48**	0.65	**2.99**	0.52			
IPI00059476	DPEP1	DIPEPTIDASE 1.	**0.57**	0.08	**0.42**	0.05	**0.25**	0.06	Yes		
IPI00292130	DPT	DERMATOPONTIN.	**3.01**	0.00	**6.08**	0.00	**4.45**	0.00			
IPI00257508	DPYSL2	DIHYDROPYRIMIDINASE-RELATED PROTEIN 2.	**1.70**	0.06	**2.09**	0.21	**1.53**	0.20			
*IPI00029111	DPYSL3	DIHYDROPYRIMIDINASE-RELATED PROTEIN 3.	**1.31**	0.00	**3.86**	0.00	**2.82**	0.00			
*IPI00025447	EEF1A1	ELONGATION FACTOR 1-ALPHA.	**0.76**	0.12	**0.57**	0.10	**0.48**	0.07		down in PanIN/cancer (iTRAQ)	
IPI00178440	EEF1B2	ELONGATION FACTOR 1-BETA.	**0.93**	0.06	**0.55**	0.07	**0.28**	0.00			down (ICAT)
*IPI00000875	*EEF1G	ELONGATION FACTOR 1-GAMMA.	**1.05**	0.16	**0.61**	0.11	**0.42**	0.09		down in PanIN/cancer (iTRAQ/ICAT)	
IPI00186290	EEF2	ELONGATION FACTOR 2.	**0.64**	0.09	**0.61**	0.10	**0.26**	0.03		down in PanIN/cancer (iTRAQ)	

*IPI00021728	*EIF2S2	EUKARYOTIC TRANSLATION INITIATION FACTOR 2 SUBUNIT 2.	**0.94**	0.00			**0.44**	0.00			
*IPI00016910	*EIF3CL	EUKARYOTIC TRANSLATION INITIATION FACTOR 3 SUBUNIT C.	**1.43**	0.00	**0.43**	0.00	**0.83**	0.00			
*IPI00654777	EIF3F	EUKARYOTIC TRANSLATION INITIATION FACTOR 3 SUBUNIT F.	**1.19**	0.00	**0.97**	0.00	**0.44**	0.00			
IPI00290460	EIF3G	EUKARYOTIC TRANSLATION INITIATION FACTOR 3 SUBUNIT G.	**0.81**	0.00			**0.49**	0.00			
IPI00012795	EIF3I	EUKARYOTIC TRANSLATION INITIATION FACTOR 3 SUBUNIT I.	**0.70**	0.00	**0.75**	0.00	**0.48**	0.00			
*IPI00012079	EIF4B	EUKARYOTIC TRANSLATION INITIATION FACTOR 4B.	**1.42**	0.00			**0.30**	0.00			
*IPI00220365	EIF4G1	EUKARYOTIC TRANSLATION INITIATION FACTOR 4 GAMMA 1	**0.71**	0.00	**0.58**	0.00	**0.38**	0.00			
*IPI00376005	EIF5A	ISOFORM 2 OF EUKARYOTIC TRANSLATION INITIATION FACTOR 5A-1.	**0.80**	0.11	**0.68**	0.12	**0.41**	0.02			
*IPI00032003	EMD	EMERIN.	**1.34**	0.00			**2.12**	0.00			
IPI00013079	EMILIN1	EMILIN-1.	**3.41**	0.59	**5.71**	1.03	**4.39**	0.81	Yes		
*IPI00184311	ENPP1	ECTONUCLEOTIDE PYROPHOSPHATASE/PHOSPHODIESTERAS E FAMILY MEMBER 1.	**0.79**	0.00	**0.54**	0.00	**0.21**	0.00	Yes		
IPI00009896	EPHX1	EPOXIDE HYDROLASE 1.	**0.82**	0.10	**0.49**	0.10	**0.11**	0.01		down in PanIN/cancer (iTRAQ)	down (iTRAQ)
*IPI00165949	ERAP1	ISOFORM 2 OF ENDOPLASMIC RETICULUM AMINOPEPTIDASE 1.	**2.16**	0.00					Yes		
IPI00061005	ERP27	ENDOPLASMIC RETICULUM RESIDENT PROTEIN ERP27.	**0.92**	0.15	**0.49**	0.06				down in PanIN/cancer (iTRAQ)	down (iTRAQ)
IPI00024911	ERP29	ENDOPLASMIC RETICULUM PROTEIN ERP29.	**0.78**	0.00	**0.62**	0.03	**0.27**	0.00			
*IPI00022143	ESYT1	ISOFORM 1 OF EXTENDED SYNAPTOTAGMIN-1.	**2.11**	0.00	**1.62**	0.00	**1.34**	0.00			
*IPI00009841	EWSR1	EWING SARCOMA BREAKPOINT REGION 1 ISOFORM 1.	**2.12**	0.00							
IPI00215746	FABP4	FATTY ACID-BINDING PROTEIN, ADIPOCYTE.					**0.31**	0.00			
*IPI00328350	FAM129A	PROTEIN NIBAN.	**0.66**	0.13	**0.47**	0.08	**0.12**	0.00		down in cancer (iTRAQ)	down (iTRAQ)
*IPI00031820	FARSA	PHENYLALANYL-TRNA SYNTHETASE ALPHA CHAIN.	**0.73**	0.02	**0.48**	0.06	**0.23**	0.00			
IPI00019770	FAU	UBIQUITIN-LIKE PROTEIN FUBI AND RIBOSOMAL PROTEIN S30 PRECURSOR.	**0.61**	0.00	**0.31**	0.00	**0.19**	0.00			
*IPI00218803	FBLN1	ISOFORM B OF FIBULIN-1.	**1.63**	0.25	**3.84**	0.12	**4.78**	0.43	Yes	up in cancer (iTRAQ/ICAT)	
IPI00328113	FBN1	FIBRILLIN-1.	**3.23**	0.42	**7.89**	1.12	**9.70**	0.57	Yes	up in cancer (iTRAQ/ICAT)	up (iCAT)
*IPI00021885	FGA	ISOFORM 1 OF FIBRINOGEN ALPHA CHAIN.	**1.41**	0.41	**2.27**	0.55	**2.42**	0.47	Yes	up in cancer (iTRAQ)	
IPI00298497	FGB	FIBRINOGEN BETA CHAIN.	**1.63**	0.29	**2.77**	0.45	**2.32**	0.41	Yes	up in PanIN/cancer (iTRAQ/ICAT)	up (iTRAQ/ICAT)
*IPI00021891	FGG	ISOFORM GAMMA-B OF FIBRINOGEN GAMMA CHAIN.	**1.32**	0.35	**2.65**	0.81	**2.30**	0.36	Yes	up in cancer (ICAT)	up (iCAT)
*IPI00296053	FH	ISOFORM MITOCHONDRIAL OF FUMARATE HYDRATASE, MITOCHONDRIAL.	**1.08**	0.06	**0.99**	0.00	**0.33**	0.00			
*IPI00009885	FKBP11	FK506-BINDING PROTEIN 11.	**0.74**	0.09	**0.56**	0.01	**0.10**	0.00		down in cancer (iTRAQ/ICAT)	down (iTRAQ)
IPI00002535	FKBP2	FK506-BINDING PROTEIN 2.	**0.60**	0.00	**0.55**	0.00	**0.21**	0.00		down in cancer (iTRAQ/ICAT)	
*IPI00302592	FLNA	ISOFORM 2 OF FILAMIN-A.	**2.65**	0.45	**3.87**	0.99	**3.91**	1.14		up in cancer (iTRAQ/ICAT)	up (iTRAQ)
*IPI00289334	FLNB	ISOFORM 1 OF FILAMIN-B.	**1.18**	0.13	**1.17**	0.12	**2.46**	0.65		up in cancer (iTRAQ)	
*IPI00178352	FLNC	ISOFORM 1 OF FILAMIN-C.					**4.32**	0.66			
*IPI00740336	FMN1	FORMIN 1.	**0.20**	0.00	**0.25**	0.00					
*IPI00022418	FN1	ISOFORM 1 OF FIBRONECTIN.	**2.16**	0.26	**5.08**	0.89	**5.75**	1.10	Yes	up in cancer (iTRAQ/ICAT)	
IPI00375676	FTL	FERRITIN.	**3.02**	0.05	**4.09**	0.57	**4.79**	0.03			up (ICAT)
*IPI00216008	G6PD	ISOFORM LONG OF GLUCOSE-6-PHOSPHATE 1-DEHYDROGENASE.	**1.78**	0.00	**2.96**	0.00	**1.91**	0.00			up (ICAT)
*IPI00783097	GARS	GLYCYL-TRNA SYNTHETASE.	**0.78**	0.05	**0.78**	0.00	**0.19**	0.00			

*IPI00025273	GART	ISOFORM LONG OF TRIFUNCTIONAL PURINE BIOSYNTHETIC PROTEIN ADENOSINE-3.	**1.07**	0.00			**0.38**	0.00			
*IPI00032103	GATM	ISOFORM MITOCHONDRIAL OF GLYCINE AMIDINOTRANSFERASE, MITOCHONDRIAL.	**0.91**	0.12	**0.55**	0.18	**0.17**	0.07		down in cancer (iTRAQ/ICAT)	
*IPI00016077	GBAS	PROTEIN NIPSNAP HOMOLOG 2.	**0.84**	0.00	**0.72**	0.00	**0.33**	0.00			
*IPI00555812	GC	VITAMIN D-BINDING PROTEIN.	**0.96**	0.00	**2.21**	0.00	**2.56**	0.00	Yes	up in cancer (iTRAQ)	
*IPI00306140	GCG	GLUCAGON.	**2.04**	0.45	**3.05**	0.73	**0.51**	0.12		down in cancer (iTRAQ)	
*IPI00002243	GGT5	ISOFORM 1 OF GAMMA-GLUTAMYLTRANSFERASE 5.	**2.23**	0.00	**5.01**	0.00	**1.59**	0.00	Yes		up (ICAT)
IPI00848226	GNB2L1	GUANINE NUCLEOTIDE-BINDING PROTEIN SUBUNIT BETA-2-LIKE 1.	**0.84**	0.03	**0.41**	0.01	**0.18**	0.00		down in cancer (iTRAQ/ICAT)	
*IPI00007061	GOLT1B	VESICLE TRANSPORT PROTEIN GOT1B.	**0.74**	0.00	**0.51**	0.00	**0.40**	0.00			
*IPI00299429	GP2	ISOFORM 1 OF PANCREATIC SECRETORY GRANULE MEMBRANE MAJOR GLYCOPROTEIN GP2.	**0.46**	0.09	**0.16**	0.08	**0.04**	0.00	Yes	down in PanIN/CA (iTRAQ/ICAT)	down (iTRAQ)
*IPI00027497	GPI	GLUCOSE-6-PHOSPHATE ISOMERASE.	**1.57**	0.22	**2.08**	0.33	**1.51**	0.28			
*IPI00026314	GSN	ISOFORM 1 OF GELSOLIN.	**2.05**	0.09	**3.10**	0.41	**2.33**	0.58		up in cancer (ICAT)	up (iTRAQ)
IPI00019755	GSTO1	GLUTATHIONE S-TRANSFERASE OMEGA-1.	**2.02**	0.00	**2.22**	0.00	**1.33**	0.00			
*IPI00294398	HADH	ISOFORM 1 OF HYDROXYACYL-COENZYME A DEHYDROGENASE, MITOCHONDRIAL.	**0.86**	0.00	**0.57**	0.00	**0.38**	0.00			
IPI00410714	*HBA2	HEMOGLOBIN SUBUNIT ALPHA.	**1.38**	0.24	**3.28**	0.82	**1.27**	0.22	Yes	down in cancer (iTRAQ/ICAT)	up (ICAT)
IPI00654755	HBB	HEMOGLOBIN SUBUNIT BETA.	**1.43**	0.23	**2.77**	0.70	**1.36**	0.38	Yes	up in PanIN (iTRAQ)	up (iTRAQ/ICAT)
*IPI00220706	*HBG2	HEMOGLOBIN SUBUNIT GAMMA-1.	**0.76**	0.04	**2.85**	1.64	**0.78**	0.18			
*IPI00377161	HIBCH	ISOFORM 2 OF 3-HYDROXYISOBUTYRYL-COA HYDROLASE, MITOCHONDRIAL.	**0.71**	0.03	**0.61**	0.00	**0.12**	0.00		down in cancer (iTRAQ)	
IPI00217468	HIST1H1B	HISTONE H1.5.	**1.32**	0.00	**1.30**	0.00	**2.22**	1.18			
*IPI00018246	HK1	ISOFORM 1 OF HEXOKINASE-1.	**2.02**	0.00	**2.17**	0.00	**2.51**	0.00			
*IPI00018755	*HMGB1L1C	HIGH MOBILITY GROUP PROTEIN 1-LIKE 10.	**0.81**	0.00	**3.04**	0.00	**0.96**	0.00			
*IPI00334587	HNRNPAB	ISOFORM 2 OF HETEROGENEOUS NUCLEAR RIBONUCLEOPROTEIN A/B.	**2.26**	0.00	**1.76**	0.00	**1.64**	0.00			
*IPI00171903	HNRNPM	ISOFORM 1 OF HETEROGENEOUS NUCLEAR RIBONUCLEOPROTEIN M.	**1.25**	0.06	**3.19**	1.17	**1.12**	0.10			
*IPI00431645	HP	HAPTOGLOBIN.	**0.72**	0.08	**1.82**	0.00	**6.54**	2.48	Yes	up in cancer (iTRAQ)	
IPI00022371	HRG	HISTIDINE-RICH GLYCOPROTEIN.	**1.87**	0.25	**3.13**	0.75	**2.19**	0.39	Yes		up (iTRAQ/ICAT)
*IPI00031107	HSDL2	ISOFORM 2 OF HYDROXYSTEROID DEHYDROGENASE-LIKE PROTEIN 2.	**2.75**	0.00	**2.90**	0.00					
IPI00027230	HSP90B1	ENDOPLASMIN.	**0.87**	0.12	**0.51**	0.12	**0.26**	0.04	Yes	down in cancer (iTRAQ)	down (iTRAQ)
IPI00003362	HSPA5	HSPA5 PROTEIN.	**0.75**	0.10	**0.46**	0.10	**0.21**	0.03		down in cancer (iTRAQ/ICAT)	down (iTRAQ)
*IPI00022433	HSPB6	HEAT SHOCK PROTEIN BETA-6.	**1.67**	0.00	**2.65**	0.00	**1.28**	0.00			
*IPI00784154	HSPD1	60 KDA HEAT SHOCK PROTEIN, MITOCHONDRIAL.	**1.07**	0.16	**0.96**	0.26	**0.45**	0.04			
*IPI00916345	*HSPE1	PUTATIVE UNCHARACTERIZED PROTEIN HSPE1.	**0.43**	0.00	**0.57**	0.00	**0.46**	0.00		down in cancer (iTRAQ)	
IPI00024284	HSPG2	BASEMENT MEMBRANE-SPECIFIC HEPARAN SULFATE PROTEOGLYCAN CORE PROTEIN.	**1.77**	0.34	**2.46**	0.32	**1.21**	0.16	Yes		
*IPI00000877	HYOU1	HYPOXIA UP-REGULATED PROTEIN 1.	**1.13**	0.14	**0.73**	0.14	**0.22**	0.02	Yes	down in cancer (iTRAQ)	
*IPI00011107	IDH2	ISOCITRATE DEHYDROGENASE [NADP], MITOCHONDRIAL.	**0.86**	0.04	**0.75**	0.09	**0.27**	0.01		down in cancer (iTRAQ)	
*IPI00386524	IGHA1	CDNA FLJ25298 FIS, CLONE STM07683, HIGHLY SIMILAR TO PROTEIN TRO ALPHA1 H,MYELOMA.	**2.91**	0.53	**3.64**	1.42			Yes	up in cancer (iTRAQ)	
*IPI00423461	*IGHA2	IG ALPHA-2 CHAIN C REGION.	**2.65**	0.44	**3.18**	1.34	**3.69**	1.01	Yes	up in cancer (iTRAQ/ICAT)	
*IPI00385264	IGHM	IG MU HEAVY CHAIN DISEASE PROTEIN.	**1.21**	0.00	**2.21**	0.32	**2.10**	0.27	Yes	up in cancer (iTRAQ/ICAT)	
*IPI00030205	*IGKV3-20	IG KAPPA CHAIN V-III REGION HAH.	**3.06**	0.00						up in PanIN (iTRAQ)	up (iTRAQ)
IPI00001508	*INS	INSULIN.	**1.06**	0.09	**2.20**	0.38	**0.51**	0.03		down in cancer (iTRAQ)	
*IPI00217561	ITGB1	ISOFORM BETA-1C OF INTEGRIN BETA-1.	**1.67**	0.00	**2.14**	0.00	**1.89**	0.00	Yes		up (ICAT)

*IPI00292530	ITIH1	INTER-ALPHA-TRYPSIN INHIBITOR HEAVY CHAIN H1.	**1.95**	0.00	**3.21**	0.00	**7.71**	0.00	Yes		
IPI00060715	KCTD12	BTB/POZ DOMAIN-CONTAINING PROTEIN KCTD12.	**2.15**	0.20	**2.49**	0.13	**1.62**	0.03			
IPI00157790	KIAA0368	KIAA0368 PROTEIN.	**2.51**	0.00	**1.32**	0.00	**0.96**	0.00			
IPI00220327	KRT1	KERATIN, TYPE II CYTOSKELETAL 1.	**1.41**	0.22	**2.03**	0.32	**1.61**	0.33			
IPI00554788	KRT18	KERATIN, TYPE I CYTOSKELETAL 18.	**1.06**	0.20	**0.67**	0.14	**0.48**	0.15	Yes		
IPI00479145	KRT19	KERATIN, TYPE I CYTOSKELETAL 19.	**1.54**	0.25	**2.42**	0.49	**4.06**	1.51			
IPI00306959	KRT7	KERATIN, TYPE II CYTOSKELETAL 7.	**1.69**	0.39	**1.86**	0.49	**3.28**	1.22		up in cancer (iTRAQ)	
IPI00019359	KRT9	KERATIN, TYPE I CYTOSKELETAL 9.	**1.02**	0.18	**2.60**	0.00	**3.88**	0.00			
IPI00783665	LAMA5	LAMININ SUBUNIT ALPHA-5.	**1.64**	0.35	**2.06**	0.41	**0.96**	0.24	Yes		
IPI00298281	LAMC1	LAMININ SUBUNIT GAMMA-1.	**1.75**	0.15	**2.04**	0.18	**1.03**	0.06	Yes		
IPI00010471	LCP1	PLASTIN-2.	**0.98**	0.15	**2.18**	0.41	**1.48**	0.48			up (ICAT)
*IPI00217966	LDHA	ISOFORM 1 OF L-LACTATE DEHYDROGENASE A CHAIN.	**1.77**	0.08	**2.34**	0.04	**4.72**	0.33		up in cancer (iTRAQ/ICAT)	
*IPI00219217	LDHB	L-LACTATE DEHYDROGENASE B CHAIN.	**1.05**	0.04	**0.60**	0.05	**0.35**	0.05		down in cancer (iTRAQ)	
IPI00219219	LGALS1	GALECTIN-1.	**2.99**	0.00	**6.17**	0.00	**6.83**	0.00		up in cancer (iTRAQ/ICAT)	up (iTRAQ)
IPI00009750	LGALS4	GALECTIN-4.	**2.88**	0.00	**2.64**	0.00	**2.11**	0.00			
IPI00026530	LMAN1	PROTEIN ERGIC-53.	**0.93**	0.10	**0.47**	0.03	**0.26**	0.00		down in cancer (iTRAQ)	down (iTRAQ)
*IPI00655812	LMNA	RHABDOMYOSARCOMA ANTIGEN MU-RMS-40.12.	**2.09**	0.33	**2.89**	0.47	**1.92**	0.33		up in cancer (iTRAQ)	
IPI00005186	*LOC10013	HLA CLASS II HISTOCOMPATIBILITY ANTIGEN, DP(W4) BETA CHAIN.	**0.93**	0.00	**1.53**	0.00	**2.09**	0.00	Yes		
IPI00887169	*LOC10029	PUTATIVE UNCHARACTERIZED PROTEIN.	**2.08**	0.06	**4.00**	0.54	**4.77**	0.19		up in cancer (iTRAQ)	up (iTRAQ)
*IPI00384938	*LOC10029	PUTATIVE UNCHARACTERIZED PROTEIN DKFZP686N02209.			**6.63**	0.34	**7.75**	0.86	Yes		up (iTRAQ/ICAT)
IPI00888126	LOC652797	SIMILAR TO PYRUVATE KINASE, MUSCLE.					**2.00**	0.44			
*IPI00140827	*LOC72882	SMT3 SUPPRESSOR OF MIF TWO 3 HOMOLOG 2 ISOFORM B PRECURSOR.	**1.32**	0.00	**2.02**	0.00	**1.86**	0.00			
IPI00783271	LRPPRC	LEUCINE-RICH PPR MOTIF-CONTAINING PROTEIN, MITOCHONDRIAL.	**0.67**	0.13	**0.82**	0.00	**0.33**	0.00			
IPI00396321	LRRC59	LEUCINE-RICH REPEAT-CONTAINING PROTEIN 59.	**0.67**	0.12	**0.65**	0.10	**0.23**	0.00		down in PanIN/cancer (iTRAQ)	
*IPI00298860	LTF	CDNA FLJ78440, HIGHLY SIMILAR TO HUMAN LACTOFERRIN.	**0.72**	0.00	**4.80**	1.35	**0.75**	0.00	Yes		
IPI00020986	LUM	LUMICAN.	**6.29**	1.09	**12.82**	1.75	**13.05**	2.06	Yes	up in cancer (iTRAQ/ICAT)	up (iTRAQ)
IPI00106687	LXN	LATEXIN.	**4.63**	0.00	**8.46**	0.00	**7.26**	0.00			
IPI00019038	LYZ	LYSOZYME C.	**2.11**	0.00	**2.53**	0.00	**4.08**	0.00			
*IPI00291006	MDH2	MALATE DEHYDROGENASE, MITOCHONDRIAL.	**0.74**	0.04	**0.62**	0.05	**0.50**	0.06		down in cancer (iTRAQ)	
*IPI00022792	MFAP4	MICROFIBRIL-ASSOCIATED GLYCOPROTEIN 4.	**1.74**	0.00	**3.01**	0.00	**1.79**	0.00	Yes		
*IPI00029046	MLEC	MALECTIN.	**0.67**	0.00	**0.39**	0.00			Yes	down in PanIN/cancer (iTRAQ)	down (iTRAQ)
*IPI00328170	MOGS	MANNOSYL-OLIGOSACCHARIDE GLUCOSIDASE.	**1.05**	0.07	**0.55**	0.04	**0.24**	0.01	Yes	down in cancer (iTRAQ)	down (iTRAQ)
*IPI00007244	MPO	ISOFORM H17 OF MYELOPEROXIDASE.	**1.97**	0.25	**3.36**	0.39	**2.00**	0.27	Yes		
*IPI00011876	MTAP	S-METHYL-5'-THIOADENOSINE PHOSPHORYLASE.	**1.55**	0.18	**1.80**	0.00	**2.57**	0.00			
IPI00017510	*MT-CO2	CYTOCHROME C OXIDASE SUBUNIT 2.	**1.09**	0.00	**0.47**	0.00	**0.34**	0.00			
*IPI00000105	MVP	MAJOR VAULT PROTEIN.	**1.53**	0.52	**2.49**	0.75	**2.04**	0.63			
*IPI00397526	MYH10	ISOFORM 1 OF MYOSIN-10.	**1.73**	0.24	**2.14**	0.38	**2.50**	0.13			
*IPI00020501	MYH11	MYOSIN-11.	**2.63**	0.64	**2.98**	0.84	**3.16**	0.73			
*IPI00337335	MYH14	ISOFORM 1 OF MYOSIN-14.					**2.27**	0.01			
IPI00019502	MYH9	ISOFORM 1 OF MYOSIN-9.	**1.54**	0.17	**1.88**	0.27	**2.22**	0.35		up in cancer (ICAT)	
*IPI00335168	*MYL6B	ISOFORM NON-MUSCLE OF MYOSIN LIGHT POLYPEPTIDE 6.	**1.65**	0.21	**2.66**	0.09	**2.34**	0.09		up in PanIN/cancer (iTRAQ)	
*IPI00023748	NACA	NASCENT POLYPEPTIDE-ASSOCIATED COMPLEX SUBUNIT ALPHA.	**1.24**	0.00	**0.47**	0.17	**0.27**	0.00			
IPI00147874	NANS	SIALIC ACID SYNTHASE.	**0.83**	0.00	**0.56**	0.00	**0.37**	0.00			
*IPI00017763	NAP1L4	CDNA FLJ59403, HIGHLY SIMILAR TO NUCLEOSOME ASSEMBLY PROTEIN 1-LIKE 4.	**0.80**	0.00	**1.02**	0.00	**0.30**	0.00			
IPI00306960	NARS	ASPARAGINYL-TRNA SYNTHETASE, CYTOPLASMIC.	**0.87**	0.09	**0.63**	0.05	**0.32**	0.05			

*IPI00029561	NDUFA10	NADH DEHYDROGENASE [UBIQUINONE] 1 ALPHA SUBCOMPLEX SUBUNIT 10, MITOCHONDRIAL.	**1.02**	0.00	**0.90**	0.00	**0.31**	0.00			
IPI00003968	NDUFA9	NADH DEHYDROGENASE [UBIQUINONE] 1 ALPHA SUBCOMPLEX SUBUNIT 9, MITOCHONDRIAL.	**1.32**	0.10	**1.01**	0.00	**0.36**	0.00			
*IPI00028908	NID2	NIDOGEN-2.	**1.85**	0.00	**2.17**	0.00	**1.66**	0.00	Yes		
*IPI00009123	NUCB2	ISOFORM 1 OF NUCLEOBINDIN-2.	**0.52**	0.03	**0.31**	0.00	**0.10**	0.00			
*IPI00098902	OGDH	2-OXOGLUTARATE DEHYDROGENASE E1 COMPONENT, MITOCHONDRIAL.	**1.33**	0.03	**0.96**	0.04	**0.49**	0.07			
IPI00025465	OGN	CDNA FLJ59205, HIGHLY SIMILAR TO MIMECAN.	**6.08**	0.30	**6.41**	0.29	**3.16**	0.18	Yes		
*IPI00216106	OLA1	ISOFORM 1 OF OBG-LIKE ATPASE 1.	**0.67**	0.05	**0.52**	0.00	**0.38**	0.00			
*IPI00022429	ORM1	ALPHA-1-ACID GLYCOPROTEIN 1.	**1.79**	0.19	**4.74**	0.42	**6.19**	0.57	Yes		up (iTRAQ)
IPI00010796	P4HB	PROTEIN DISULFIDE-ISOMERASE.	**0.75**	0.13	**0.45**	0.11	**0.12**	0.02		down in cancer (iTRAQ)	
*IPI00012726	PABPC4	ISOFORM 1 OF POLYADENYLATE-BINDING PROTEIN 4.	**0.68**	0.02	**0.67**	0.06	**0.33**	0.01		down in cancer (ICAT)	
IPI00298547	PARK7	PROTEIN DJ-1.	**1.10**	0.07	**0.98**	0.05	**0.45**	0.00		down in PanIN/cancer (iTRAQ)	
IPI00449049	PARP1	POLY [ADP-RIBOSE] POLYMERASE 1.			**0.43**	0.00				down in cancer (iTRAQ)	
IPI00002280	PCSK1N	PROSAAS.	**2.59**	0.00	**5.94**	0.43	**2.45**	0.00	Yes		
*IPI00011571	PDIA2	PROTEIN DISULFIDE-ISOMERASE A2.	**0.74**	0.15	**0.45**	0.13	**0.07**	0.00	Yes	down in cancer (iTRAQ/ICAT)	
IPI00025252	PDIA3	PROTEIN DISULFIDE-ISOMERASE A3.	**0.98**	0.12	**0.76**	0.16	**0.45**	0.05		down in cancer (iTRAQ)	
IPI00009904	PDIA4	PROTEIN DISULFIDE-ISOMERASE A4.	**0.88**	0.13	**0.80**	0.10	**0.20**	0.03		down in cancer (iTRAQ)	down (iTRAQ)
*IPI00299571	PDIA6	ISOFORM 2 OF PROTEIN DISULFIDE-ISOMERASE A6.	**0.88**	0.13	**0.45**	0.10	**0.20**	0.05		down in PanIN/cancer (iTRAQ)	down (iTRAQ)
*IPI00007935	PDLIM5	PDZ AND LIM DOMAIN PROTEIN 5.	**1.24**	0.00	**2.10**	0.00	**1.75**	0.00			
IPI00219446	PEBP1	PHOSPHATIDYLETHANOLAMINE-BINDING PROTEIN 1.	**0.79**	0.08	**0.67**	0.09	**0.18**	0.01		down in PanIN/cancer (iTRAQ)	down (iTRAQ)
IPI00216691	PFN1	PROFILIN-1.	**1.38**	0.14	**1.81**	0.04	**2.13**	0.13		up in cancer (iTRAQ/ICAT)	
*IPI00022213	PGC	PROGASTRICSIN.	**3.14**	0.00							
IPI00011200	PHGDH	D-3-PHOSPHOGLYCERATE DEHYDROGENASE.	**0.69**	0.07	**0.34**	0.06	**0.16**	0.04		down in cancer (iTRAQ)	
*IPI00028786	PKD1	ISOFORM 3 OF POLYCYSTIN-1.	**1.36**	0.13	**2.14**	0.23	**1.31**	0.19	Yes		
*IPI00479186	PKM2	ISOFORM M2 OF PYRUVATE KINASE ISOZYMES M1/M2.	**1.22**	0.11	**1.45**	0.22	**2.52**	0.54		up in cancer (ICAT)	
*IPI00021792	PLA2G1B	PHOSPHOLIPASE A2.	**1.15**	0.11			**0.04**	0.00		down in PanIN (iTRAQ); down in cancer (iTRAQ/ICAT)	down (iTRAQ)
*IPI00398779	PLEC1	ISOFORM 3 OF PLECTIN-1.	**1.46**	0.18	**2.22**	0.35	**3.07**	0.34			
IPI00027720	PNLIP	PANCREATIC TRIACYLGLYCEROL LIPASE.	**0.71**	0.14	**0.35**	0.12	**0.02**	0.00	Yes	down in cancer (iTRAQ/ICAT)	
IPI00005924	PNLIPRP2	PANCREATIC LIPASE-RELATED PROTEIN 2.	**1.62**	0.22	**0.49**	0.11			Yes	down in PanIN/cancer (iTRAQ)	down (iTRAQ)
*IPI00007960	POSTN	ISOFORM 1 OF PERIOSTIN.	**1.21**	0.41	**2.38**	0.72	**3.26**	1.61	Yes	up in cancer (iTRAQ)	
IPI00646304	PPIB	PEPTIDYL-PROLYL CIS-TRANS ISOMERASE B.	**0.74**	0.09	**0.57**	0.09	**0.33**	0.05	Yes	down in cancer (iTRAQ)	
*IPI00000874	PRDX1	PEROXIREDOXIN-1.	**0.97**	0.11	**0.80**	0.17	**0.45**	0.13			
*IPI00024919	PRDX3	PEROXIREDOXIN 3 ISOFORM B.	**0.90**	0.02	**0.66**	0.06	**0.40**	0.00			
*IPI00639945	*PRDX4	PEROXIREDOXIN-4.	**0.76**	0.10	**0.49**	0.09	**0.21**	0.07		down in PanIN/cancer (iTRAQ)	down (iTRAQ)
*IPI00220301	PRDX6	PEROXIREDOXIN-6.	**0.82**	0.01	**0.69**	0.00	**0.35**	0.01		down in PanIN/cancer (iTRAQ)	
IPI00020987	PRELP	PROLARGIN.	**7.14**	0.00	**10.81**	0.00	**29.40**	0.00	Yes	up in PanIN/cancer (iTRAQ)	
*IPI00217053	PRRC1	ISOFORM 1 OF PROTEIN PRRC1.			**0.49**	0.00					
*IPI00011694	PRSS1	TRYPSIN-1.	**0.66**	0.12	**0.25**	0.06	**0.03**	0.00		down in PanIN/cancer (iTRAQ/ICAT)	down (iTRAQ)

*IPI00011695	*PRSS2	PROTEASE SERINE 2 ISOFORM B.	**0.72**	0.08	**0.32**	0.07	**0.06**	0.02		down in PanIN/cancer (iTRAQ/ICAT)	down (iTRAQ)
*IPI00020042	*PSMC4	SIMILAR TO PROTEASOME 26S ATPASE SUBUNIT 4.					**0.30**	0.00			
*IPI00012268	PSMD2	26S PROTEASOME NON-ATPASE REGULATORY SUBUNIT 2.	**2.29**	0.00							
*IPI00176903	PTRF	ISOFORM 1 OF POLYMERASE I AND TRANSCRIPT RELEASE FACTOR.	**2.39**	0.00	**2.91**	0.01	**1.39**	0.14			down (iTRAQ)
IPI00004358	PYGB	GLYCOGEN PHOSPHORYLASE, BRAIN FORM.	**1.59**	0.01	**1.33**	0.04	**2.15**	0.71		up in cancer (iTRAQ)	
*IPI00026665	QARS	CDNA FLJ75085, HIGHLY SIMILAR TO HOMO SAPIENS GLUTAMINYL-TRNA SYNTHETASE (QARS), MRNA.	**1.19**	0.00	**0.21**	0.00				down in PanIN/cancer (iTRAQ)	
IPI00009027	REG1A	LITHOSTATHINE-1-ALPHA.	**0.72**	0.02	**0.99**	0.10	**0.33**	0.00	Yes	down in cancer (iTRAQ/ICAT)	
IPI00550069	RNH1	RIBONUCLEASE INHIBITOR.	**1.94**	0.00	**2.59**	0.00	**1.75**	0.00			
IPI00412579	RPL10A	60S RIBOSOMAL PROTEIN L10A.	**0.61**	0.00	**0.35**	0.00	**0.21**	0.00		down in cancer (iTRAQ)	down (ICAT)
*IPI00376798	RPL11	ISOFORM 1 OF 60S RIBOSOMAL PROTEIN L11.	**0.77**	0.06	**0.61**	0.00	**0.30**	0.00			
IPI00465361	RPL13	60S RIBOSOMAL PROTEIN L13.	**0.74**	0.06	**0.57**	0.15	**0.19**	0.04			
*IPI00304612	*RPL13A	60S RIBOSOMAL PROTEIN L13A.	**0.69**	0.00	**0.32**	0.00	**0.18**	0.00		down in PanIN/cancer (iTRAQ)	down (iTRAQ)
*IPI00069693	RPL14	RIBOSOMAL PROTEIN L14 VARIANT.	**0.93**	0.15	**0.72**	0.13	**0.47**	0.10		down in PanIN/cancer (iTRAQ)	
IPI00215719	RPL18	60S RIBOSOMAL PROTEIN L18.	**0.67**	0.01	**0.36**	0.02	**0.23**	0.01		down in PanIN (iTRAQ)	
*IPI00219153	RPL22	60S RIBOSOMAL PROTEIN L22.	**0.85**	0.00	**0.44**	0.00	**0.34**	0.00		down in PanIN/cancer (iTRAQ/ICAT)	down (iTRAQ)
*IPI00306332	RPL24	60S RIBOSOMAL PROTEIN L24.	**0.68**	0.16	**0.42**	0.00	**0.14**	0.00		down in cancer (iTRAQ)	down (iTRAQ)
IPI00219155	RPL27	60S RIBOSOMAL PROTEIN L27.	**0.49**	0.03	**0.57**	0.13	**0.20**	0.00		down in cancer (iTRAQ)	
*IPI00173589	RPL29P4	60S RIBOSOMAL PROTEIN L29.	**0.74**	0.00	**0.48**	0.00	**0.22**	0.00			
IPI00003918	RPL4	60S RIBOSOMAL PROTEIN L4.	**0.67**	0.07	**0.53**	0.12	**0.23**	0.03		down in PanIN/cancer (iTRAQ)	
IPI00000494	RPL5	60S RIBOSOMAL PROTEIN L5.	**0.66**	0.10	**0.37**	0.00	**0.40**	0.00		down in cancer (iTRAQ)	down (iTRAQ)
*IPI00329389	RPL6	60S RIBOSOMAL PROTEIN L6.	**0.75**	0.05	**0.59**	0.10	**0.35**	0.08		down in PanIN/cancer (iTRAQ)	
*IPI00299573	RPL7A	60S RIBOSOMAL PROTEIN L7A.	**0.74**	0.09	**0.67**	0.07	**0.27**	0.02		down in cancer (iTRAQ)	
*IPI00030179	RPL7P32	60S RIBOSOMAL PROTEIN L7.	**0.79**	0.06	**0.71**	0.17	**0.25**	0.05		down in PanIN/cancer (iTRAQ)	
*IPI00012772	RPL8	60S RIBOSOMAL PROTEIN L8.	**0.86**	0.07	**0.48**	0.01	**0.28**	0.01		down in cancer (iTRAQ)	down (iCAT)
IPI00031691	RPL9	60S RIBOSOMAL PROTEIN L9.	**0.72**	0.13	**0.30**	0.00	**0.22**	0.00		down in PanIN/cancer (iTRAQ)	
*IPI00008530	RPLP0	60S ACIDIC RIBOSOMAL PROTEIN P0.	**0.75**	0.05	**0.55**	0.03	**0.31**	0.03		down in cancer (iTRAQ)	
IPI00008527	RPLP1	60S ACIDIC RIBOSOMAL PROTEIN P1.	**0.60**	0.05	**0.40**	0.00	**0.17**	0.00		down in cancer (iTRAQ)	
IPI00008529	RPLP2	60S ACIDIC RIBOSOMAL PROTEIN P2.	**0.81**	0.05	**0.71**	0.07	**0.36**	0.08		down in PanIN/cancer (iTRAQ)	
IPI00025874	RPN1	DOLICHYL-DIPHOSPHOOLIGOSACCHARIDE--PROTEIN GLYCOSYLTRANSFERASE SUBUNIT 1 PRECURSOR.	**0.77**	0.07	**0.56**	0.09	**0.23**	0.03	Yes	down in cancer (iTRAQ)	
IPI00936387	RPN2	RIBOPHORIN II, ISOFORM CRA_D.	**0.67**	0.11	**0.48**	0.09	**0.21**	0.03		down in cancer (iTRAQ)	
*IPI00008438	RPS10	40S RIBOSOMAL PROTEIN S10.	**0.73**	0.00	**0.38**	0.02	**0.27**	0.00		down in PanIN/cancer (iTRAQ)	down (iTRAQ)
*IPI00025091	RPS11	40S RIBOSOMAL PROTEIN S11.	**0.60**	0.00			**0.22**	0.00		down in cancer (iTRAQ/ICAT)	
IPI00026271	RPS14	40S RIBOSOMAL PROTEIN S14.	**0.72**	0.06	**0.27**	0.01	**0.20**	0.01			down (iTRAQ)

*IPI00156232	RPS15A	40S RIBOSOMAL PROTEIN S15A.	**0.82**	0.00	**0.50**	0.00	**0.25**	0.00			
*IPI00221092	RPS16	40S RIBOSOMAL PROTEIN S16.	**0.84**	0.07	**0.53**	0.08	**0.28**	0.00		down in cancer (iTRAQ)	
IPI00215780	RPS19	40S RIBOSOMAL PROTEIN S19.	**0.77**	0.13	**0.65**	0.17	**0.25**	0.02			
*IPI00012493	RPS20	40S RIBOSOMAL PROTEIN S20.			**0.52**	0.10	**0.21**	0.00		down in cancer (iTRAQ/ICAT)	
*IPI00218606	RPS23	40S RIBOSOMAL PROTEIN S23.	**0.75**	0.00			**0.36**	0.00			
*IPI00029750	RPS24	ISOFORM 1 OF 40S RIBOSOMAL PROTEIN S24.	**0.68**	0.00	**0.39**	0.00	**0.27**	0.00		down in PanIN/cancer (iTRAQ)	
*IPI00012750	RPS25	40S RIBOSOMAL PROTEIN S25.	**0.62**	0.00	**0.33**	0.00	**0.21**	0.00		down in PanIN/cancer (iTRAQ)	
*IPI00376121	RPS26	SIMILAR TO 40S RIBOSOMAL PROTEIN S26.	**0.84**	0.00	**1.17**	0.00	**0.47**	0.00		down in cancer (iTRAQ)	down (iTRAQ)
*IPI00556589	RPS28	40S RIBOSOMAL PROTEIN S28.	**0.79**	0.00	**0.65**	0.16	**0.19**	0.00		down in cancer (iTRAQ)	
IPI00011253	RPS3	40S RIBOSOMAL PROTEIN S3.	**0.87**	0.09	**0.54**	0.07	**0.27**	0.01		down in cancer (iTRAQ/ICAT)	
*IPI00419880	RPS3A	40S RIBOSOMAL PROTEIN S3A.	**1.14**	0.09	**0.47**	0.00	**0.30**	0.00		down in cancer (iTRAQ)	down (iTRAQ)
IPI00217030	RPS4X	40S RIBOSOMAL PROTEIN S4, X ISOFORM.	**0.66**	0.06	**0.48**	0.10	**0.30**	0.08		down in PanIN/cancer (iTRAQ/iCAT)	down (iTRAQ)
IPI00008433	RPS5	40S RIBOSOMAL PROTEIN S5.	**0.69**	0.00	**0.34**	0.00	**0.23**	0.00		down in cancer (iTRAQ)	
*IPI00013415	RPS7	40S RIBOSOMAL PROTEIN S7.	**0.78**	0.08	**0.56**	0.20	**0.26**	0.05		down in cancer (iTRAQ)	
*IPI00216587	RPS8	40S RIBOSOMAL PROTEIN S8.	**0.81**	0.06	**0.60**	0.08	**0.34**	0.01			
IPI00221088	RPS9	40S RIBOSOMAL PROTEIN S9.	**0.72**	0.06	**0.59**	0.10	**0.39**	0.15		down in PanIN/cancer (iTRAQ)	
*IPI00215743	RRBP1	ISOFORM 3 OF RIBOSOME-BINDING PROTEIN 1.	**0.79**	0.13	**0.54**	0.12	**0.19**	0.03		down in cancer (iTRAQ/ICAT)	
*IPI00021766	RTN4	ISOFORM 1 OF RETICULON-4.	**1.73**	0.00	**2.90**	0.00	**2.21**	0.00			
IPI00027463	S100A6	PROTEIN S100-A6.	**3.32**	0.00	**5.60**	0.00	**8.34**	0.00			
IPI00007047	S100A8	PROTEIN S100-A8.	**0.64**	0.00	**2.55**	0.00	**2.33**	0.00			
*IPI00027462	S100A9	PROTEIN S100-A9.	**1.07**	0.28	**3.34**	0.43	**2.80**	0.43		up in cancer (iTRAQ)	
*IPI00034308	SARDH	SARCOSINE DEHYDROGENASE, MITOCHONDRIAL.	**0.47**	0.05	**0.59**	0.07					
*IPI00008730	SCGN	SECRETAGOGIN.	**3.30**	0.00	**2.46**	0.00	**0.59**	0.00			
*IPI00217143	SDHA	"CDNA FLJ61478, HIGHLY SIMILAR TO SUCCINATE DEHYDROGENASE (UBIQUINONE) FLAVOPROTEIN SUBUNIT, MITOCHONDRIAL."	**1.20**	0.07	**0.79**	0.13	**0.29**	0.00		down in cancer (iTRAQ/ICAT)	
*IPI00219436	SEC11C	SIGNAL PEPTIDASE COMPLEX CATALYTIC SUBUNIT SEC11C.	**0.75**	0.16	**0.59**	0.07	**0.16**	0.00		down in cancer (iTRAQ)	
IPI00006865	SEC22B	VESICLE-TRAFFICKING PROTEIN SEC22B.	**1.01**	0.05	**0.89**	0.19	**0.19**	0.00			
IPI00024661	SEC24C	PROTEIN TRANSPORT PROTEIN SEC24C.			**2.52**	0.00					
*IPI00220835	*SEC61B	PROTEIN TRANSPORT PROTEIN SEC61 SUBUNIT BETA.	**0.90**	0.00	**0.90**	0.00	**0.34**	0.00			
*IPI00410693	SERBP1	ISOFORM 1 OF PLASMINOGEN ACTIVATOR INHIBITOR 1 RNA-BINDING PROTEIN.	**1.02**	0.04	**0.56**	0.00	**0.41**	0.06			
IPI00553177	SERPINA1	ISOFORM 1 OF ALPHA-1-ANTITRYPSIN.	**1.25**	0.21	**2.42**	0.47	**3.99**	0.75	Yes	up in cancer (iTRAQ)	up (iTRAQ)
*IPI00550991	SERPINA3	ISOFORM 1 OF ALPHA-1-ANTICHYMOTRYPSIN.	**1.88**	0.11	**3.12**	0.00	**4.62**	0.03	Yes		
*IPI00413451	SERPINB6	SERPIN B6.	**0.94**	0.06	**0.60**	0.03	**0.48**	0.00			
*IPI00032179	SERPINC1	ANTITHROMBIN-III.	**0.76**	0.00	**2.30**	0.00	**2.00**	0.00	Yes	up in cancer (iTRAQ)	up (ICAT)
*IPI00291866	SERPING1	PLASMA PROTEASE C1 INHIBITOR.	**3.34**	0.00	**2.64**	0.00	**3.87**	0.00	Yes	up in cancer (iTRAQ)	up (iTRAQ)
IPI00032140	SERPINH1	SERPIN H1.	**2.09**	0.33	**3.40**	0.76	**4.53**	1.13	Yes		
*IPI00010402	SH3BGRL3	SH3 DOMAIN BINDING GLUTAMIC ACID-RICH PROTEIN LIKE 3.	**1.61**	0.00	**0.86**	0.00	**2.24**	0.00			
*IPI00022202	SLC25A3	ISOFORM A OF PHOSPHATE CARRIER PROTEIN, MITOCHONDRIAL.	**1.02**	0.00	**0.82**	0.00	**0.44**	0.00			
*IPI00791534	SLC4A1	BAND 3 ANION TRANSPORT PROTEIN.	**2.21**	0.07	**3.52**	0.24	**1.04**	0.07	Yes		
*IPI00140420	SND1	STAPHYLOCOCCAL NUCLEASE DOMAIN-CONTAINING PROTEIN 1.	**0.71**	0.06	**0.67**	0.12	**0.25**	0.05		down in cancer (iTRAQ)	
*IPI00017469	SPR	SEPIAPTERIN REDUCTASE.	**0.77**	0.05	**0.69**	0.16	**0.36**	0.00			
*IPI00216704	SPTB	ISOFORM 1 OF SPECTRIN BETA CHAIN, ERYTHROCYTE.	**1.75**	0.00	**3.21**	0.00	**1.49**	0.00			

*IPI00009634	SQRDL	SULFIDE:QUINONE OXIDOREDUCTASE, MITOCHONDRIAL.			**1.71**	0.00	**2.51**	0.00			
*IPI00301021	SSR1	ISOFORM 1 OF TRANSLOCON-ASSOCIATED PROTEIN SUBUNIT ALPHA.	**0.76**	0.00	**0.34**	0.00	**0.15**	0.00	Yes		
*IPI00019385	SSR4	TRANSLOCON-ASSOCIATED PROTEIN SUBUNIT DELTA PRECURSOR.	**0.55**	0.03	**0.29**	0.04	**0.11**	0.00		down in PanIN/cancer (iTRAQ)	down (iTRAQ)
IPI00297492	STT3A	DOLICHYL-DIPHOSPHOOLIGOSACCHARIDE--PROTEIN GLYCOSYLTRANSFERASE SUBUNIT STT3A.	**0.78**	0.03	**0.66**	0.00	**0.29**	0.00	Yes	down in PanIN/cancer (iTRAQ)	
IPI00096066	SUCLG2	SUCCINYL-COA LIGASE [GDP-FORMING] SUBUNIT BETA, MITOCHONDRIAL.	**1.10**	0.13	**0.63**	0.09	**0.39**	0.06			
*IPI00300026	*SULT1A1	SULFOTRANSFERASE 1A1.	**1.34**	0.00	**2.30**	0.00	**0.80**	0.00			
*IPI00005737	SURF4	ISOFORM 1 OF SURFEIT LOCUS PROTEIN 4.	**0.76**	0.00	**0.64**	0.05	**0.25**	0.00		down in cancer (ICAT)	
*IPI00020194	*TAF15	ISOFORM SHORT OF TATA-BINDING PROTEIN-ASSOCIATED FACTOR 2N.	**1.55**	0.00	**2.22**	0.00	**1.34**	0.00			
IPI00216138	TAGLN	TRANSGELIN.	**3.19**	0.45	**4.95**	0.31	**7.14**	0.37		up in cancer (iTRAQ)	up (iTRAQ)
*IPI00329633	TARS	THREONYL-TRNA SYNTHETASE, CYTOPLASMIC.	**0.82**	0.10	**0.75**	0.06	**0.36**	0.03		down in cancer (iTRAQ)	
IPI00022463	TF	SEROTRANSFERRIN.	**2.86**	1.12	**4.13**	0.37	**4.27**	1.51	Yes	up in cancer (iTRAQ/ICAT)	up (iTRAQ/ICAT)
IPI00018219	TGFBI	TRANSFORMING GROWTH FACTOR-BETA-INDUCED PROTEIN IG-H3.	**2.94**	0.00	**5.63**	0.00	**7.15**	0.00			
IPI00294578	TGM2	ISOFORM 1 OF PROTEIN-GLUTAMINE GAMMA-GLUTAMYLTRANSFERASE 2.	**2.37**	0.36	**4.51**	0.31	**4.36**	0.28		up in cancer (ICAT)	
*IPI00022892	THY1	THY-1 MEMBRANE GLYCOPROTEIN.	**4.70**	0.00	**3.31**	0.00	**2.75**	0.00	Yes		
*IPI00005563	TINAGL1	ISOFORM 1 OF TUBULOINTERSTITIAL NEPHRITIS ANTIGEN-LIKE.	**2.22**	0.22	**2.28**	0.27	**0.97**	0.00	Yes		
*IPI00643920	TKT	CDNA FLJ54957, HIGHLY SIMILAR TO TRANSKETOLASE.	**0.90**	0.10	**0.78**	0.08	**0.39**	0.02		down in PanIN/cancer (iTRAQ)	
IPI00298994	TLN1	TALIN-1.	**1.67**	0.32	**2.69**	0.56	**1.82**	0.36			
*IPI00018415	TM9SF2	TRANSMEMBRANE 9 SUPERFAMILY MEMBER 2.	**0.78**	0.00	**0.20**	0.00	**0.30**	0.00			
*IPI00028055	TMED10	TRANSMEMBRANE EMP24 DOMAIN-CONTAINING PROTEIN 10.	**0.68**	0.00			**0.24**	0.00	Yes		down (iTRAQ)
IPI00063130	TMEM205	TRANSMEMBRANE PROTEIN 205.	**1.08**	0.40	**0.57**	0.00	**0.36**	0.00		down in cancer (iTRAQ)	
*IPI00619951	TPD52	TUMOR PROTEIN D52 ISOFORM 2.	**0.74**	0.03			**0.46**	0.00			
IPI00940084	TPM1	37 KDA PROTEIN.					**4.62**	1.22		up in cancer (iTRAQ)	up (iTRAQ)
*IPI00218820	TPM2	ISOFORM 2 OF TROPOMYOSIN BETA CHAIN.	**2.45**	0.53	**3.51**	0.71	**3.85**	0.45		up in cancer (iTRAQ/ICAT)	up (ICAT)
*IPI00218319	TPM3	ISOFORM 2 OF TROPOMYOSIN ALPHA-3 CHAIN.	**1.46**	0.20	**2.02**	0.40	**1.81**	0.36			up (iTRAQ)
*IPI00010779	TPM4	ISOFORM 1 OF TROPOMYOSIN ALPHA-4 CHAIN.	**2.22**	0.41	**3.47**	0.64	**4.06**	0.74		up in cancer (iTRAQ/ICAT)	up (iTRAQ)
IPI00742682	TPR	NUCLEOPROTEIN TPR.	**1.18**	0.00	**1.05**	0.00	**0.48**	0.00			
*IPI00010274	*TPSAB1	ISOFORM 1 OF TRYPTASE ALPHA-1.	**3.29**	0.31	**3.72**	0.17	**1.91**	0.38	Yes	up in cancer (ICAT)	up (ICAT)
*IPI00014361	TSTA3	GDP-L-FUCOSE SYNTHETASE.	**0.47**	0.00	**1.41**	0.00	**0.52**	0.00			
*IPI00022432	TTR	TRANSTHYRETIN.	**1.68**	0.24	**3.22**	0.53	**1.52**	0.28	Yes		
IPI00305281	TTYH2	PROTEIN TWEETY HOMOLOG 2.	**1.32**	0.00	**1.94**	0.00	**2.06**	0.00	Yes		
*IPI00645452	TUBB	TUBULIN, BETA.	**1.23**	0.13	**2.06**	0.35	**1.88**	0.39			
*IPI00007752	TUBB2C	TUBULIN BETA-2C CHAIN.	**0.87**	0.05	**0.64**	0.05	**0.42**	0.03			
IPI00027107	TUFM	TU TRANSLATION ELONGATION FACTOR, MITOCHONDRIAL PRECURSOR.	**1.09**	0.07	**0.70**	0.15	**0.48**	0.09		down in cancer (iTRAQ)	
*IPI00292858	TYMP	THYMIDINE PHOSPHORYLASE.	**1.28**	0.00	**2.80**	0.20	**2.82**	0.94		up in cancer (iTRAQ)	
*IPI00013847	UQCRC1	CYTOCHROME B-C1 COMPLEX SUBUNIT 1, MITOCHONDRIAL.	**1.06**	0.03	**0.92**	0.00	**0.43**	0.04		down in cancer (iTRAQ)	
IPI00009329	UTRN	UTROPHIN.	**3.20**	0.00	**2.44**	0.00					
IPI00156689	VAT1	SYNAPTIC VESICLE MEMBRANE PROTEIN VAT-1 HOMOLOG.	**1.97**	0.16	**2.59**	0.52	**0.96**	0.28			
*IPI00009802	VCAN	ISOFORM V0 OF VERSICAN CORE PROTEIN.			**8.20**	0.00	**20.75**	0.00	Yes	up in cancer (iTRAQ/ICAT)	
*IPI00291175	VCL	ISOFORM 1 OF VINCULIN.	**1.69**	0.22	**2.08**	0.21	**1.89**	0.26			
IPI00418471	VIM	VIMENTIN.	**2.37**	0.32	**4.19**	0.70	**2.78**	0.50		up in PanIN/cancer (iTRAQ/ICAT)	up (iTRAQ)
*IPI00298961	XPO1	EXPORTIN-1.			**2.12**	0.00					
IPI00007074	YARS	TYROSYL-TRNA SYNTHETASE, CYTOPLASMIC.	**0.89**	0.02	**0.62**	0.00	**0.37**	0.00		down in cancer (iTRAQ)	

IPI00021263	YWHAZ	14-3-3 PROTEIN ZETA/DELTA.	**1.29**	0.22	**1.28**	0.17	**2.02**	0.42
IPI00550731		PUTATIVE UNCHARACTERIZED PROTEIN.	**4.41**	0.00	**5.44**	0.00	**5.69**	0.00
IPI00909509		CDNA FLJ59138, HIGHLY SIMILAR TO ANNEXIN A2.	**2.14**	0.19	**3.54**	0.40	**3.73**	0.51
